# Rational plug–bridge design to enhance catalytic efficiency of sulfonic acid-functionalized plugged ethyl/imidazolium-based bifunctional periodic mesoporous organosilica

**DOI:** 10.1039/d5na01099c

**Published:** 2026-06-26

**Authors:** Hesamoddin Moradi, Yaser Maleki, Nasim Ganji, Omid Pourshiani, Babak Karimi, Hojatollah Vali, Piero Mastrorilli, Stefano Todisco

**Affiliations:** a Department of Chemistry, Institute for Advanced Studies in Basic Sciences (IASBS) Zanjan 45137-66731 Iran bkarimi48@gmail.com karimi@iasbs.ac.ir; b Research Center for Basic Sciences & Modern Technologies (RBST), Department of Chemistry, Institute for Advanced Studies in Basic Sciences (IASBS) Zanjan 45137-66731 Iran; c Department of Anatomy and Cell Biology, Facility for Electron Microscopy Research, McGill University Montreal Quebec H3A 2A7 Canada; d DICATECh, Politecnico di Bari Bari I-70125 Italy

## Abstract

A series of sulfonic acid-functionalized plugged and unplugged bifunctional periodic mesoporous organosilicas incorporating either ethyl-imidazolium or phenyl-imidazolium bridging units (P/U BFPMO-IL-PrSO_3_Hs, including Et-U-BFPMO-IL-PrSO_3_H (1a), Et-P-BFPMO-IL-PrSO_3_H (1b), Ph-U-BFPMO-IL-PrSO_3_H (1c) and Ph-P-BFPMO-IL-PrSO_3_H (1d)) were synthesized and systematically evaluated as solid acid catalysts in direct esterification and Biginelli reactions. The synthetic protocol involves a modified co-condensation method using organosilica and silica precursors in the presence of Pluronic P123 as a structural directing agent and KCl as an additive under acidic conditions, followed by grafting with mercaptopropyl groups and oxidation to create sulfonic acid-functionalized bi-functional PMO nanostructures. The key difference between the plugged and unplugged variants lies in the order of introducing the silica and organosilica precursors during synthesis. Among these materials, the catalyst 1b exhibited the highest catalytic performance, surpassing its unplugged analogs as well as reference catalysts such as mesoporous silica SBA-15-PrSO_3_H (2) and periodic mesoporous organosilica Et-PMO-Me(PrSO_3_H) (3). Considering a number of comparative studies, the superior activity of this catalyst may be largely attributed to its combination of organic bridges and channel plugs. These structural features enhance the accessibility and reactivity of sulfonic acid sites while prolonging the residence time of reactants near active centers and expelling out the by-produced water from the interior of the mesochannels. The 1b catalyst demonstrated excellent efficiency in the esterification of various alcohols and carboxylic acids and in the synthesis of dihydropyrimidinones *via* the Biginelli reaction under mild conditions. Moreover, it exhibited remarkable stability and reusability, retaining high catalytic performance over multiple reaction cycles without significant deactivation.

## Abstract 2

Sulfonic acid-functionalized plugged and unplugged bifunctional PMOs bearing ethyl- or phenyl-imidazolium bridges (1a–1d) were synthesized and investigated as efficient solid acid catalysts for esterification and Biginelli reactions.

## Graphical abstract

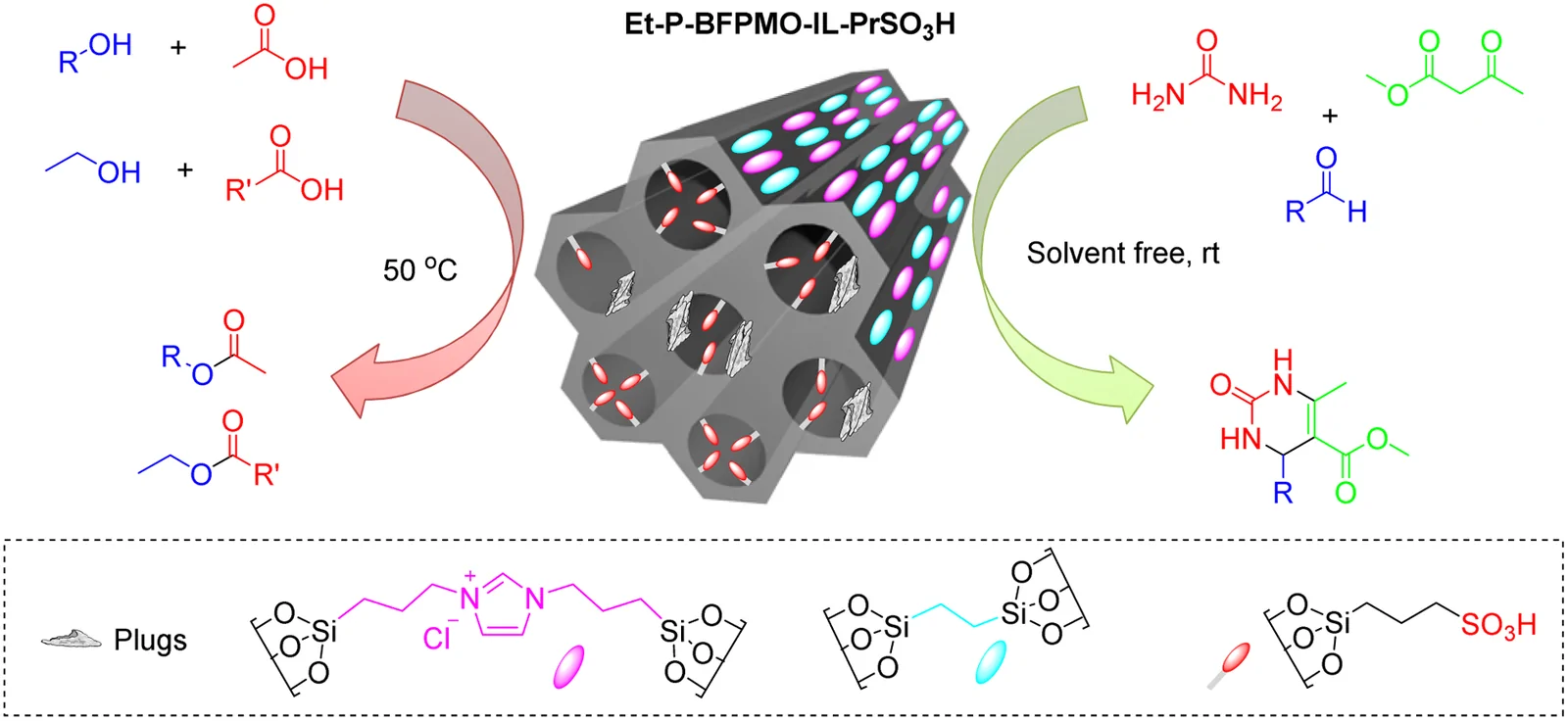

## Introduction

Acid-catalyzed reactions, such as esterification, aldol condensation, Biginelli condensation, acylation, hydrolysis, *etc.*, constitute a significant fraction of important organic transformations, utilized in various settings, ranging from chemistry laboratories to industrial applications.^1–4^ Nevertheless, many of these reactions continue to rely on traditional homogeneous and non-recyclable acid catalysts, which present challenges such as toxicity, corrosion, the need for large quantities of catalyst, difficulties in separation and waste neutralization, lack of reusability, and other issues. Consequently, considerable efforts have been focused on substituting these homogeneous acids with solid acids to tackle the environmental problems that have inadvertently persisted due to these catalytic systems over the past few decades.^1–10^ In recent years, there has been a proliferation of diverse heterogeneous acid catalysts incorporating supported sulfonic acid groups, which have proven to be recyclable solid acids capable of facilitating a broad spectrum of acid-catalyzed reactions with varying degrees of success. Along this line, various solid carriers, including carbon, metal–organic frameworks (MOFs), mesoporous silica, and periodic mesoporous organosilica (PMO) materials, have been employed, resulting in the development of versatile sulfonic acid-functionalized catalysts with broad applicability.^11–14^ A thorough examination of various types of solid sulfonic acids reveals that, beyond acidic strength and the loading of acidic sulfonic groups, a crucial factor in developing efficient catalysts with desirable selectivity, long-lasting activity, and stability is the adjustment of the hydrophobic–hydrophilic balance within the nanospaces of these materials.^11–19^ These nanospaces contain the active centres where reactions occur. Specifically, careful control of the hydrophobic–hydrophilic balance can significantly influence the mass transfer of reactants and products with different polarities, thereby facilitating reaction progress and providing greater control over reaction selectivity. Moreover, this rational design plays a crucial role in minimizing the poisoning of the acidic sites by water and/or other polar molecules, which may be involved in reactions as starting materials, by-products or solvents.^11–14,20^ In this context, organic–inorganic hybrid PMO materials have proven to be highly efficient carriers for preparing sulfonic acid-modified catalysts. These catalysts have found applications in a diverse range of acid-catalyzed transformations.^21–33^ PMO materials, in addition to deriving from notable characteristics like high surface area, adjustable pore sizes, and exceptional hydrothermal stability, present distinctive prospects for accurately adjusting the hydrophobic–hydrophilic environment encompassing acidic sites through the meticulous selection of the organic bridging group within the framework.^11–14^ In an illustrative instance, our research team discovered that a geminal methyl-propyl sulfonic acid-functionalized PMO material containing ethyl bridges (Et-PMO-Me-PrSO_3_H) exhibited enhanced catalytic performance in comparison to a PMO-based catalyst with identical sulfonic acid tagged groups but distinct (phenyl) bridges (Ph-PMO-Me-PrSO_3_H) in the esterification process of fatty acids with alcohols. This enhanced catalytic activity was clearly attributed to the well-balanced hydrophilic–hydrophobic properties of the Et-PMO catalyst through providing several compelling pieces of evidence.^26^ Furthermore, it has been demonstrated that the incorporation of imidazolium-based ionic liquid bridges into the structure of PMO materials (PMO-IL),^34–43^ in conjunction with the functionalized sulfonic acid groups within their mesospaces, has led to the development of bi-functional mesoporous solid acids. These materials have displayed remarkable catalytic efficacy in esterification^44^ and Biginelli reactions.^45^ This enhanced synergistic effect was attributed to successfully merging the benefits of the ordered mesoporous hybrid structure provided by PMOs with the amphiphilic ILs and acidic sulfonic acid groups that are closely located within the same nanospaces.

Another promising approach to achieving a compromise between the hydrophobic and hydrophilic properties within the mesochannels of PMO materials involves incorporating microporous silica nanocapsules, known as “plugs” in these areas. These plugs facilitate the creation of hydrophilic nanoregions enriched in silanol groups within the comparatively hydrophobic mesochannels of PMOs. In addition, the plug technology facilitates the construction of mesoporous materials possessing precise control over substrate/product mass transfer within their nanospaces. This allows for the modulation of selectivity in reaction outcomes by utilizing either a confinement effect mechanism and/or regulating the residence time of substrates and reaction intermediates within the nanospaces of the catalysts under the influence of plugs located near the catalytic centers. The control over the formation and the extension of these plugs within the mesochannels can be effectively achieved by adjusting various factors. These factors include changing the pH of the reaction mixture, aging temperature, co-solvent concentration, silica/surfactant molar ratios, and the order in which silica and organosilica precursors are introduced. These factors play a significant role in influencing the hydrolysis and co-condensation rate of silica and organosilica precursors.^46–50^ Partially plugged-PMOs featuring constricted mesochannels have demonstrated significant promise in certain applications, such as the preferential adsorption of proteins,^51^ the selective conversion of alcohols to either carbonyl compounds or carboxylic acids,^52,53^ and the effective removal of chlorophenols with different polarities from waste-waters.^54^ Nevertheless, the influence of channel plugging on the efficacy of PMO-based catalysts in a broader spectrum of chemical transformations, including those catalyzed by acids, still remains a topic that necessitates additional investigation and advancement.

In our current investigation, a series of sulfonic-acid functionalized bifunctional PMOs (plugged/unplugged) by incorporating either imidazolium/ethyl or imidazolium/phenyl bridging groups have been synthesized. These materials were then utilized in the direct esterification of various alcohols and carboxylic acids, as well as in the three-component condensation of various aldehydes with methyl acetoacetate and urea (Biginelli reactions), which are classified as two fundamental classes of acid-catalyzed reactions. The primary aim of this study is to explore the potential synergistic effect of the mesoporous structure, bridging groups, and channel plugging on enhancing the activity of anchored sulfonic acid functional groups in the aforementioned reactions. Our previous research studies clearly demonstrated the role of hydrophobic–hydrophilic balance of mesoporous channels in shaping the reactivity and selectivity of the catalysts.^26,28^ However, to the best of our knowledge, the utilization of plugged/unplugged technology in the design of novel acidic functionalized PMO-based catalysts, where the chemical functionality, hydrophobic–hydrophilic balance of the catalyst, and plug properties can be controlled, has not been explored in important catalytic applications such as esterification and Biginelli reactions.

## Experimental section

### Synthesis of the ionic liquid precursor, 1,3-bis(trimethoxysilylpropyl)imidazolium chloride (BTMSPCl)

The BTMSPCl ionic liquid was synthesized according to the established procedure outlined in our previous reports.^38,49^ All reaction steps were conducted under an argon atmosphere, and all solvents were dried using appropriate reagents and freshly distilled before use. In a typical experiment, imidazole was initially re-crystallized in distilled CH_2_Cl_2_ and then dried in a desiccator with P_2_O_5_ under vacuum for 3 days at room temperature. The dried imidazole (30 mmol, 2 g), NaH 95% (0.77 g) and anhydrous THF (60 mL) were added to a two-neck flask under an argon atmosphere. The reaction mixture was stirred for 1–2 h to obtain a suspension of sodium imidazolide salt in THF. Next, 3-chloropropyltrimethoxysilane (30 mmol, 5.4 mL) was added to the suspension, and the resultant mixture was refluxed for 24 h. Following this, the reaction mixture was cooled to room temperature, and the solvent was removed under reduced pressure conditions, leaving an oily mixture containing NaCl. In the second step, 3-chloropropyltrimethoxysilane (30 mmol, 5.4 mL) and anhydrous toluene (60 mL) were added to the oily mixture. The reaction mixture was refluxed for 48 h. Upon cooling to room temperature, a biphasic mixture consisting of the BTMSPCl ionic liquid and toluene was obtained. The toluene phase was separated, and then anhydrous CH_2_Cl_2_ (60 mL) was added to the mixture to eliminate the precipitated NaCl. The CH_2_Cl_2_ phase was subsequently transferred into a two-neck flask, and the solvent was evaporated under reduced pressure. Ultimately, the resulting ionic liquid was thoroughly washed with anhydrous toluene (4 × 50 mL) to obtain pure BTMSPCl: ^1^H NMR (400 MHz, CDCl_3_): *δ*_H_ (ppm) = 10.46 (s, 1H), 7.41 (s, 2H), 4.21 (br, 4H), 3.38 (s, 18H), 0.47 (br, 4H), 1.85 (m, 4H); ^13^C NMR (100 MHz, CDCl_3_): *δ*_C_ (ppm) = 137.1, 122.0, 51.5, 50.5, 24.0, 5.8.

### Synthesis of unplugged ethyl-periodic mesoporous organosilica-ionic liquid (Et-U-PMO-IL)

The Et-U-PMO-IL material was synthesized following the procedure previously reported by our research group.^49,52,53^ Typically, Pluronic P123 (1.67 g), HCl (2 M, 46.2 g), and distilled H_2_O (10.5 g) were added to a 250 mL flask, and the resulting mixture was stirred at 35 °C for 3 h. Subsequently, KCl (8.8 g) was added to the flask, and the mixture was stirred until a homogeneous solution was formed. In the next step, a pre-prepared solution of BTMSPCl (2.00 mmol, 0.86 g) and 1,2-bis(trimethoxysilyl)ethane (BTME, 3.00 mmol, 0.811 g) in anhydrous methanol (1 mL) was added to the homogeneous solution and the resulting mixture was stirred for 15 minutes. Following this, tetramethoxyorthosilicate (TMOS, 15 mmol, 2.28 g) was poured into the mixture while stirring was continued at 35 °C for 24 h. The mixture was then heated at 96 °C under static conditions for 72 h. The resulting white solid was separated by filtration and washed with deionized water. Finally, the removal of the P123 surfactant was accomplished using a Soxhlet apparatus with a solution of conc. HCl (3 mL) in ethanol (100 mL) for 48 h.

### Synthesis of plugged ethyl-periodic mesoporous organosilica-ionic liquid (Et-P-PMO-IL)

The Et-P-PMO-IL material was synthesized following the procedure previously reported by our research group.^49,52,53^ Typically, Pluronic P123 (1.67 g), HCl (2 M, 46.2 g), and distilled H_2_O (10.5 g) were added to a 250 mL flask, and the resulting mixture was stirred at 35 °C for 3 h. Subsequently, KCl (8.8 g) was added to the flask, and the mixture was stirred until a homogeneous solution was formed. In the next step, a pre-prepared solution of BTMSPCl (2.00 mmol, 0.86 g), BTME (3.00 mmol, 0.811 g) and TMOS (15 mmol, 2.28 g) in anhydrous methanol (1 mL) was added to the homogeneous solution and the resulting mixture was stirred at 35 °C for 24 h. The mixture was then heated at 96 °C under static conditions for 72 h. The resulting white solid was separated by filtration and washed with deionized water. Finally, the removal of the P123 surfactant was accomplished using a Soxhlet apparatus with a solution of conc. HCl (3 mL) in ethanol (100 mL) for 48 h.

### Synthesis of plugged/unplugged phenyl-periodic mesoporous organosilica-ionic liquid (Ph-P/U-PMO-IL)

The Ph-P-PMO-IL and Ph-U-PMO-IL materials were synthesized following procedures identical to those described above, with the only difference being the use of 1,4-bis(triethoxysilyl)benzene (BTEB, 3 mmol, 1.21 g) instead of BTME.

### General procedure for grafting sulfonic acid groups on the prepared PMO-based materials

The surface modification of the prepared PMO-based materials with sulfonic acid groups followed our previously established protocol.^42^ In a typical grafting procedure, the synthesized PMO material (1 g) and anhydrous toluene (50 mL) were added to a 100 mL flask under an argon atmosphere. The mixture was sonicated for 10 min to ensure the complete dispersion of the solid material. Subsequently, (3-mercaptopropyl)trimethoxysilane (1 mmol, 0.2 g) was added to the mixture, and the resulting suspension was refluxed for 24 h. The obtained material was separated by filtration, washed several times with anhydrous toluene and ethanol, and dried overnight at 80 °C to achieve the desired thiol-functionalized PMO material.

The transformation of thiol groups into sulfonic acid groups was accomplished through the treatment of the thiol-functionalized PMO material with H_2_O_2_ (30 wt%). 30 mL of H_2_O_2_ was poured into a flask containing the thiol-functionalized PMO material (1 g), and the resulting suspension was stirred at room temperature for 24 h. The material was then filtered, washed repeatedly with deionized water and ethanol. The obtained solid was subsequently acidified with an aqueous sulfuric acid solution (1 M, 50 mL) for 12 h. The resulting catalyst was then filtered, thoroughly washed with deionized water to reach a neutral pH of the filtrate, and finally dried at 80 °C. The obtained catalysts were denoted as Et-U-BFPMO-IL-PrSO_3_H (1a), Et-P-BFPMO-IL-PrSO_3_H (1b), Ph-U-BFPMO-IL-PrSO_3_H (1c), and Ph-P-BFPMO-IL-PrSO_3_H (1d).

### Proton capacity of the prepared sulfonic acid catalysts

The quantitative estimation of sulfonic acid groups anchored on the solid surface was conducted by an acid–base titration method. To do this, 100 mg of the synthesized catalyst was added to an aqueous solution of NaCl (1 M, 10 mL) which was used as a cation exchanger, and the resulting mixture was stirred at room temperature for 24 h. The mixture was then filtered, followed by several rinses with deionized water. To precisely determine the degree of functionalization, the acidity of the resulting solution was evaluated by a back titration procedure. In this process, the solution was allowed to react with an excess amount of a NaOH solution (0.01 M). Subsequently, the resulting solution was titrated using a HCl solution (0.01 M) to measure the amount of NaOH consumed by the sulfonic acid catalyst. The loading of sulfonic acid groups on the 1a, 1b, 1c, and 1d catalysts was determined to be 0.10, 0.12, 0.11, and 0.10 mmol g^−1^, respectively.

### General procedure for the solvent-free esterification of various alcohols with acetic acid using the Et-P-BFPMO-IL-PrSO_3_H (1b) catalyst

An alcohol (1 mmol), acetic acid (2 mmol), and 1b (0.5 mol%) were added to a 5 mL round-bottomed flask equipped with a magnetic stir bar and condenser. The reaction mixture was stirred at 50 °C, and the reaction progress was monitored by TLC and GC analysis using 1,3,5-trimethylbenzene (TMB) as an internal standard. No significant side reactions or side-products were detected within the sensitivity limits of the instrumentation. Upon completion, the mixture was filtered and washed with ethyl acetate. Subsequently, the filtrate was extracted with a saturated aqueous NaHCO_3_ solution and deionized water, dried over Na_2_SO_4_, and concentrated. The product was purified by chromatography on silica gel, if required.

For recycling experiments, the recovered catalyst was washed with ethyl acetate and *n*-heptane several times and dried overnight at 95 °C. The dried catalyst was then weighed, and the quantities of the reactants were recalculated based on its weight. The subsequent run of the esterification reaction was performed following the aforementioned procedure.

### General procedure for the solvent-free esterification of various carboxylic acids with ethanol using 1b

A carboxylic acid (1 mmol), an excess of ethanol (1–1.5 mL), and 1b (1 mol%) were added to a 5 mL round-bottomed flask equipped with a magnetic stir bar and condenser. The reaction mixture was stirred at 50 °C, and the reaction progress was monitored by TLC and GC analysis using 1,3,5-trimethylbenzene (TMB) as an internal standard. No significant side reactions or by-products were detected within the sensitivity limits of the instrumentation. Upon completion, the mixture was filtered and washed with ethyl acetate. Subsequently, the filtrate was extracted with a saturated aqueous NaHCO_3_ solution and deionized water, dried over Na_2_SO_4_, and concentrated. The product was purified by chromatography on silica gel, if required.

### General procedure for the solvent-free Biginelli reaction using 1b

Typically, an aldehyde (1 mmol), methyl acetoacetate (1 mmol), urea (1.2 mmol), and 1b (0.3 mol%) were added to a 5 mL round-bottomed flask. The resulting mixture was stirred at room temperature under solvent-free conditions until the reaction reached completion (monitored using TLC). The reaction mixture was filtered and rinsed with deionized water. Subsequently, the solid residue was dissolved in hot ethanol, and the catalyst was separated by filtration. The filtrate was then cooled in an ice bath to precipitate the desired dihydropyrimidinone product. The crude product was further purified by recrystallization with hot ethanol.

**Scheme 1:**
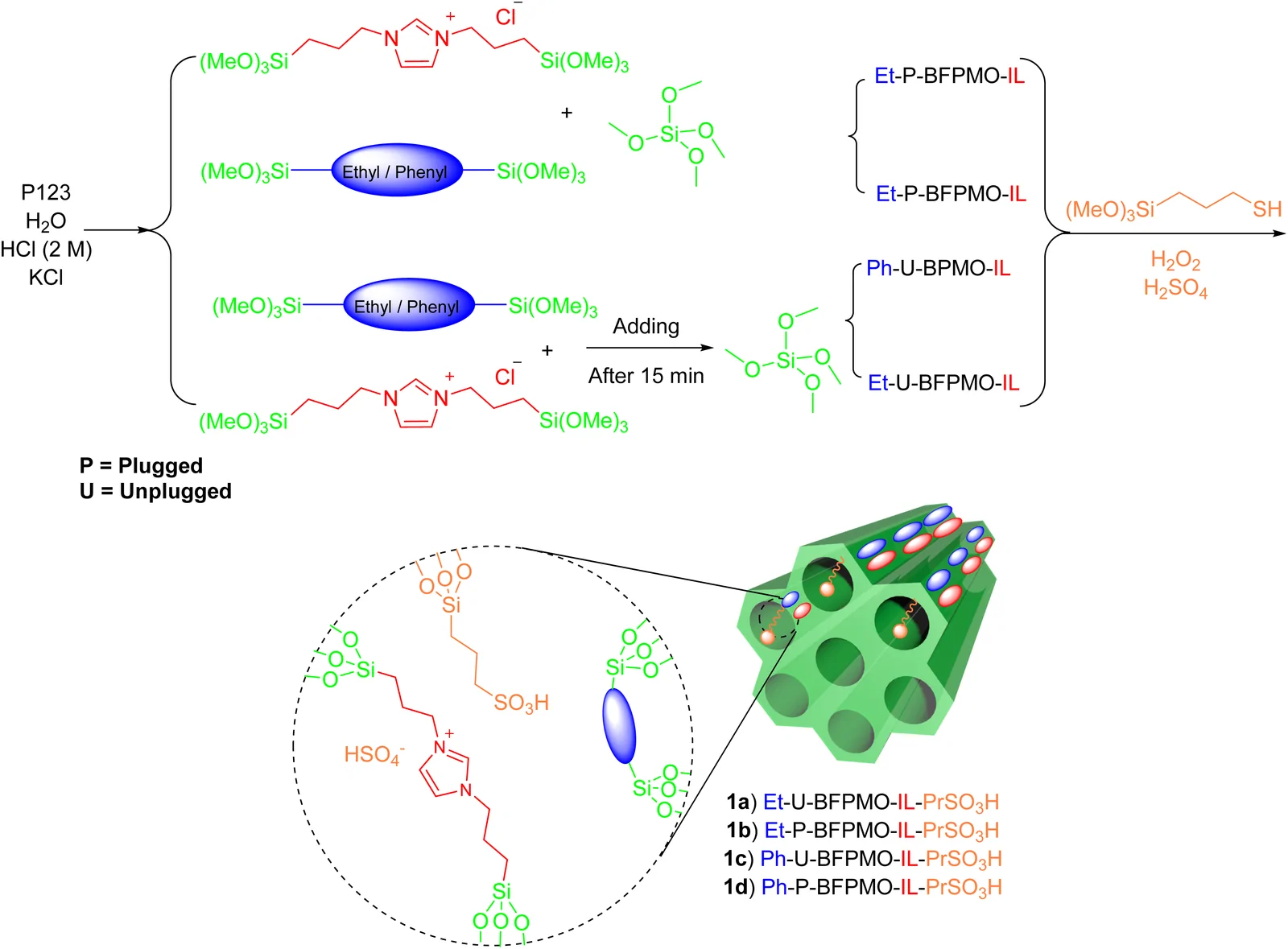
A schematic illustration outlining the synthetic steps toward the catalysts 1a–1d.

For recycling experiments, the recovered catalyst was washed with ethanol and deionized water and dried overnight at 95 °C. The dried catalyst was then weighed, and the quantities of the reactants were recalculated based on its weight. The subsequent run of the Biginelli reaction was performed following the aforementioned procedure (Scheme 1 and Fig. 5).

## Results and discussion

**Fig. 1:**
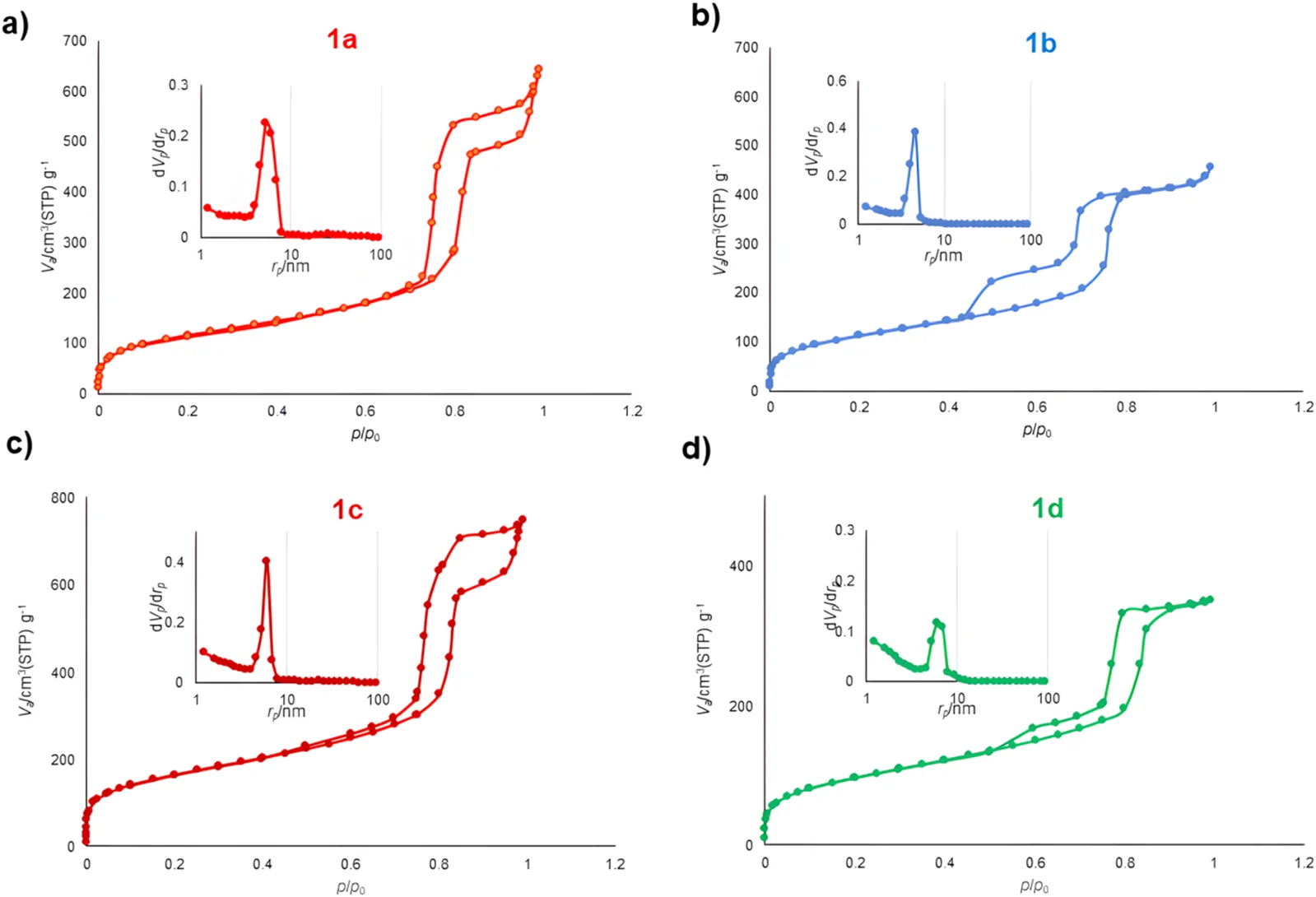
N_2_ adsorption–desorption isotherms and BJH pore size distribution curves (inset curves) for the synthesized BFPMO materials (1a–1d).

N_2_ adsorption–desorption analysis is a well-established technique for distinguishing between plugged and unplugged mesoporous materials.^46–50^Fig. 1 presents the N_2_ adsorption–desorption isotherms of all synthesized PMO materials, and their corresponding textural properties, such as specific surface area (*S*_BET_), pore diameter (*D*_BJH_), and total pore volume (*V*_t_), are collected in Table 1. The unplugged materials (1a and 1c) exhibit Type IV isotherms with a sharp, single-step capillary condensation at relative pressures (*P*/*P*_0_) between 0.7 and 0.9, consistent with abrupt N_2_ condensation upon reaching the critical pore-filling pressure (described by the Kelvin equation). The desorption branches of these unplugged materials likewise display a sharp, single-step capillary evaporation. The nearly parallel adsorption and desorption branches, accompanied by an H1-type hysteresis loop, are indicative of open cylindrical mesopores with uniform size (Fig. 1a and c).

**Table 1:** Textural parameters of the synthesized materials obtained from N_2_ adsorption–desorption analysis^a^

Catalyst	Acid capacity	BET surface area (m^2^ g^−1^)	Total pore volume (cm^3^ g^−1^)	*D*_BJH_ (nm)
Et-U-PMO-IL-SO_3_H (1a)	0.10	413	0.99	12.1
Ph-U-PMO-IL-SO_3_H (1c)	0.11	574	1.10	12.1
Et-P-PMO-IL-SO_3_H (1b)	0.12	402	0.71	9.2
Ph-P-PMO-IL-SO_3_H (1d)	0.10	343	0.54	12.1

a Determined by titration after ion-exchange (mmol H^+^ per g).

In contrast, materials 1b and 1d show Type IV isotherms, but with a less sharp capillary condensation step and broader hysteresis loop, suggesting restricted pore accessibility due to partial pore blockage, a hallmark of plugged mesoporous structures. The desorption branches of 1b and 1d reveal more distinctive features consistent with plugged mesostructures.^44,47^ Notably, these materials also display a two-step desorption branch, comprising a desorption step at high relative pressure, associated with N_2_ release from open mesopores, and a delayed desorption step at a relative pressure of approximately ∼0.45, attributed to N_2_ release from partially blocked pores (Fig. 1b and d). This delayed step is associated with the cavitation phenomenon, wherein liquid N_2_ trapped behind plugs requires a lower pressure to evaporate. Additionally, the presence of two distinct peaks in the Dollimore–Heal (DH) pore size distribution curves further confirms partial pore blockage in 1b and 1d (Fig. S1 and S2). Importantly, material 1d exhibits a smaller fraction of partially blocked mesopores compared to 1b, as evidenced by its isotherm and supported by DH curves. The acid capacities of the catalysts 1a–1d were determined to be 0.10, 0.12, 0.11, and 0.10 mmol g^−1^, respectively, indicating very similar values within experimental error (Table 1).

After verifying the presence or absence of plugs within the channels of all sulfonic acid-functionalized bi-functional PMO-IL materials, our subsequent objective was to evaluate these systems in esterification to identify the most efficient catalyst by highlighting the significance of plugs in the easiest and straightforward pathway possible. The rationale behind this hypothesis stems from the observation that the loading levels of the organic bridging groups, and particularly the grafted sulfonic acid functionalities, are closely comparable in these materials, with the primary distinguishing feature being the presence or absence of plugs within their respective mesoporous channels.

Esters are widely recognized for their versatile applications across various industries, serving as solvents, pharmaceuticals, plastics, lubricants, fragrances, flavouring agents, perfume additives, herbicides, and others.^55^ Extensive research in this field has shown that the effectiveness of solid acid catalysts in direct esterification reactions of alcohols with carboxylic acids (referred to as Fischer esterification) relies on three crucial factors: their water compatibility, recyclability, and mass transfer capacity.^26,56,57^ As previously outlined in this manuscript, both our group and other research groups have, over the past decade, reported extensive analytical and catalytic investigations, including water adsorption–desorption analyses and convincing comparative studies of functionalized and unfunctionalized mesoporous systems within organic and inorganic frameworks. These studies clearly establish that tuning the hydrophilic–hydrophobic balance of sulfonic acid-functionalized mesoporous materials plays a decisive role in controlling product selectivity and yield in important acid-catalyzed transformations.^15–19,21–33^ However, the primary aim of this study is to investigate the role of plugs while simultaneously assessing the impact of bridged groups on the hydrophobic–hydrophilic balance within the mesopores of sulfonic acid-functionalized mesoporous organosilicas during acid-catalyzed reactions.

**Fig. 2:**
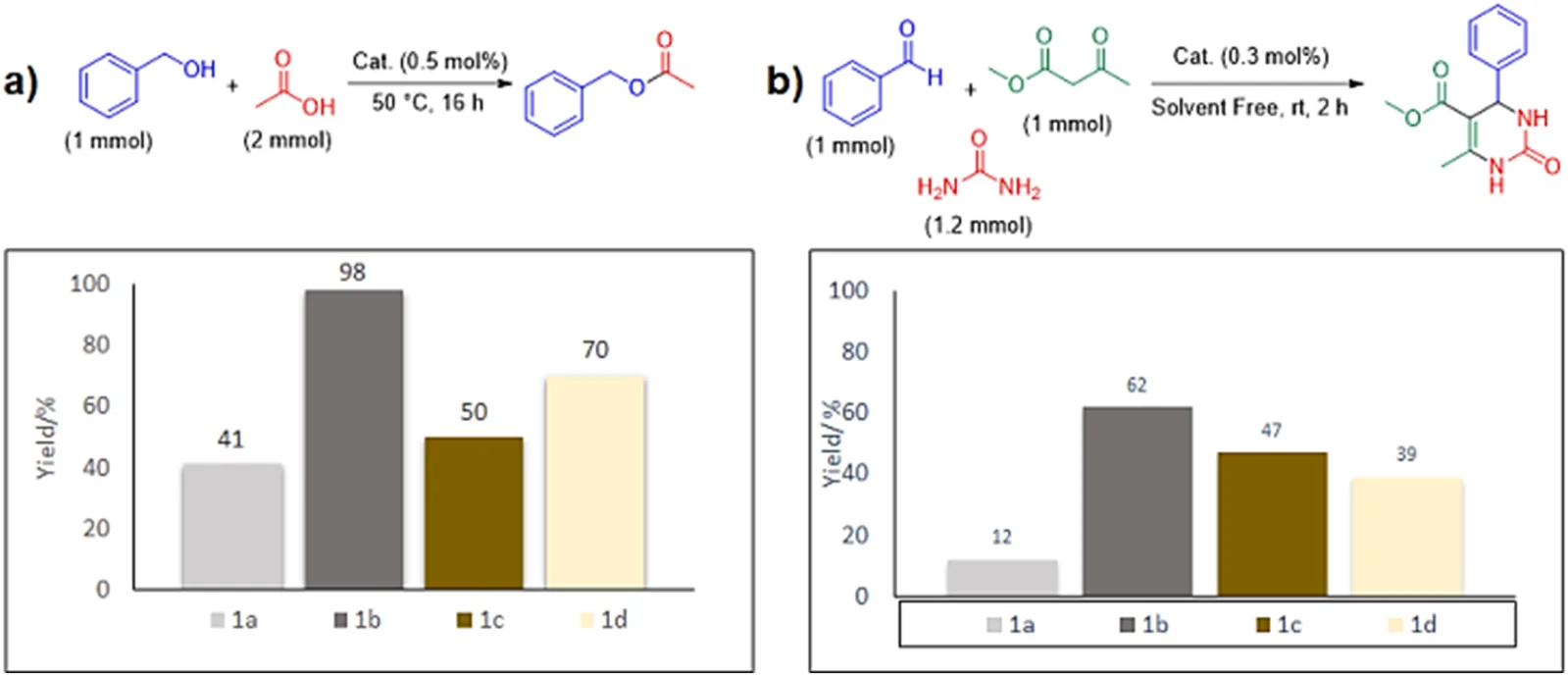
The catalytic performance of the synthesized PMO-based sulfonic acids 1a–1d in (a) esterification and (b) Biginelli reactions.

To commence our inquiry, we evaluated the catalytic performance of the synthesized plugged/unplugged sulfonic acid catalysts in a model esterification reaction involving acetic acid (2 mmol) and benzyl alcohol (1 mmol) using 0.5 mol% catalyst at 50 °C under solvent-free conditions for a fixed reaction time of 16 h. As shown in Fig. 2a, 1b exhibited superior reactivity compared to the other catalysts. Notably, although catalyst 1b has a much lower acid capacity compared to some reported organosilica-based sulfonic acid catalysts, it exhibits competitive or even superior activity relative to many existing systems (Table S1). This underscores that catalytic performance is not determined solely by acid site loading. This finding is consistent with previous studies,^15–19,26^ which found no direct correlation between acid site concentration and activity in PMO-based catalysts. Instead, catalytic efficiency is influenced by multiple factors, including the accessibility of acid sites, pore size of the catalyst, the local environment surrounding sulfonic acid groups, proximity effects, and the diffusion of reagents and products through the pores. In particular, the improved activity of our catalyst compared to Et-PMO-Me-PrSO_3_H^26^ should likely result from the additional synergistic cooperation between the imidazolium moieties, bridged organic functionalities, and the plugs present in our system.

Similarly, the catalytic performance of the synthesized BFPMO-based sulfonic acids 1a–1d was further investigated in the Biginelli reaction. This reaction particularly presents a challenging transformation as it shares similarities and competes with the Hantzsch reaction.^58^ Biginelli products, 3,4-dihydropyrimidin-2-ones/thiones (DHPMs), exhibit a wide array of biological and pharmacological properties, encompassing anticancer, antibacterial, antiviral, antihypertensive, HIV inhibition, and calcium channel-blocking activities.^59,60^ Previous studies have confirmed that the attainment of high yield and selectivity in this reaction, which encompasses both hydrophilic (urea or thiourea) and hydrophobic (an aldehyde and a β-keto ester) substrates, is significantly influenced by the hydrophobic/hydrophilic characteristics of the acid catalyst utilized.^28,61^ To investigate the potential synergistic effects of the plugs and functionalized organic bridges within the nanospaces of our catalyst systems on reaction selectivity, we conducted a study using a model reaction. This reaction involved benzaldehyde (1.0 mmol), methyl acetoacetate (1.0 mmol), and urea (1.2 mmol) in the presence of the synthesized sulfonic acid catalysts 1a–1d (0.3 mol%) at room temperature. Again, catalyst 1b exhibited superior activity compared to the other catalysts tested, yielding the desired DHPM product with a promising 62% yield within 2 hours (Fig. 2b). Considering that all solid acid catalysts tested (1a–1d) in both reactions possess nearly identical loadings of organic bridging functionalities, it is reasonable to assume that the hydrophilic–hydrophobic balance arising from these functional groups is also broadly comparable across the catalyst frameworks. Therefore, while the presence of organic (ethyl or phenyl) and imidazolium bridges is a fundamental prerequisite for achieving initial activity and selectivity in all examined catalyst systems (Table S1), the notably greater degree of plugs in the nanospaces of 1b appears to play a pivotal role in fulfilling the criteria for the highest activity observed with this catalyst in both the aforementioned esterification and Biginelli reactions. Further support for this interpretation is provided by the pore size distribution of catalysts 1a–1d. Catalyst 1b displays a reduced pore size relative to the others (Table 1), which can be attributed to the presence of a higher degree of microporous plugs within the mesoporous framework. Such structural confinement likely enhances catalytic performance by increasing the residence time of reactants in proximity to the sulfonic acid sites. This feature is corroborated by the distinct two-step desorption profile observed in the N_2_ adsorption–desorption isotherm (Fig. 1b).

**Fig. 3:**
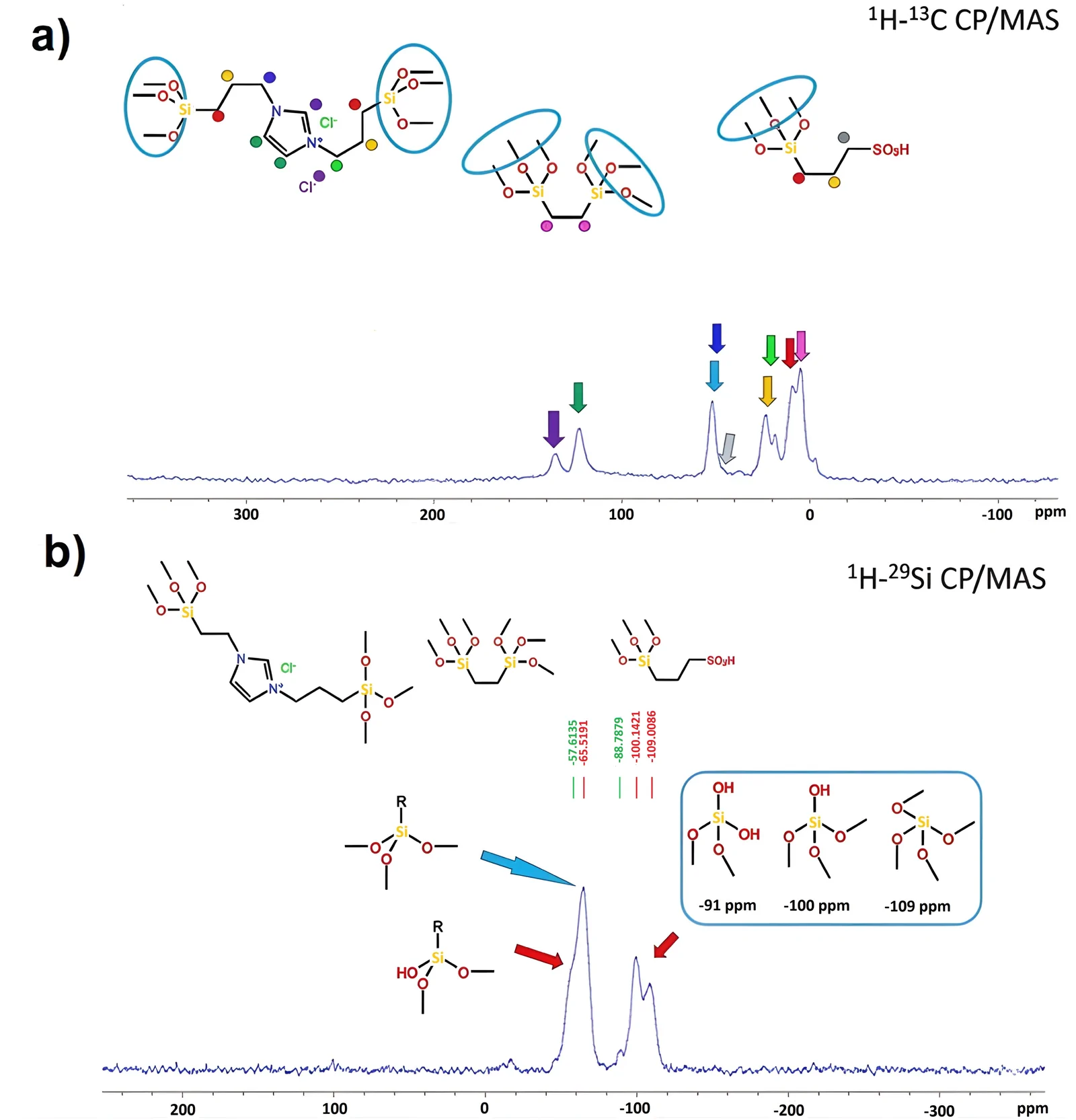
(a) ^13^C CP-MAS NMR spectrum and (b) ^29^Si CP-MAS NMR spectrum of the 1b catalyst.

After initially confirming the superior catalytic activity of 1b in the acid-catalyzed reactions mentioned above compared to the other catalysts examined, we proceeded to further characterize its structure using various analytical techniques. The FTIR spectrum of 1b was first examined to identify the functional groups existing in the catalyst structure (Fig. S3, SI). The distinctive sharp peaks at approximately 1200 and 960 cm^−1^ are associated with the asymmetric and symmetric stretching vibrations of Si–O–Si bonds, respectively. The band observed at 790 cm^−1^ is assigned to the Si–C bending vibrations.^25,42^ The peaks appearing in the 3160–2890 cm^−1^ range are attributed to stretching vibrations of C–H bonds of ethyl, propyl, and olefinic C–H of imidazole groups.^60^ The peaks appearing at 1570 and 1650 cm^−1^ correspond to C=C and C=N bonds, respectively, confirming the existence of the imidazolium ring within the structure.^43^ The broad peak at 3350 is mainly attributed to the stretching vibration of OH groups throughout the surface of the silica framework. The presence of sulfonic acid functional groups is verified by the peaks at 1290 and 1384 cm^−1^, which correspond to the symmetric and asymmetric stretching vibrations of S=O bonds.^25,61,62^ The successful integration of organic groups within the catalyst framework was further confirmed through ^13^C cross-polarization magic angle spinning (CP-MAS) NMR spectroscopy (Fig. 3a and S4, SI). The ^13^C CP-MAS NMR spectrum of 1b exhibits a peak at 3.8 ppm, which is associated with the carbons of the ethane bridges and –CH_2_–**CH**_**2**_–Si. The peak at 9.1 ppm corresponds to –**CH**_**2**_–CH_2_–Si carbons, respectively.

**Fig. 4:**
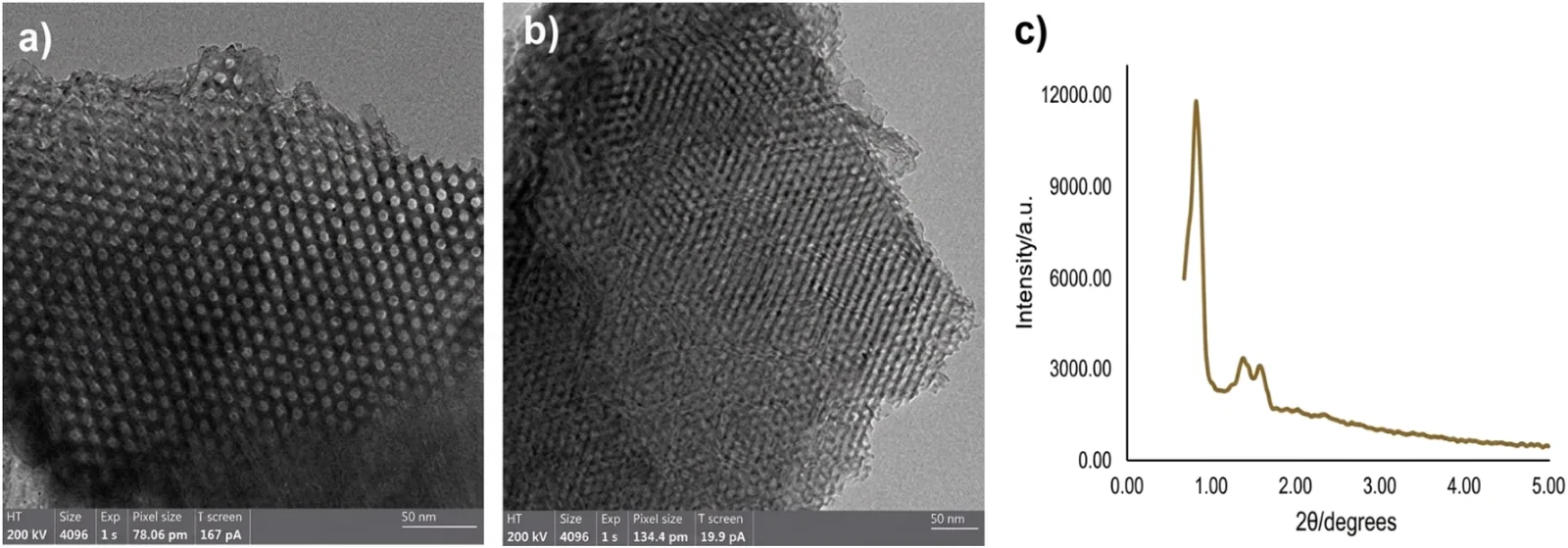
(a and b) TEM images (scale bar: 50 nm) and (c) PXRD pattern of catalyst 1b.

The peaks at 19, 22, and 51 ppm are assigned to the –N–CH_2_–**CH**_**2**_–CH_2_, –CH_2_–**CH**_**2**_–CH_2_–SO_3_H, and –N–**CH**_**2**_–CH_2_ (and also the residue of unhydrolyzed –Si(OMe) functionalities) carbons of the propyl groups within the catalyst framework, respectively. Additionally, the signal at 47 ppm is attributed to the –CH_2_–SO_3_H carbons. The signals at 122 and 137 ppm most likely originate from –**C**=**C**– and –N–**C**=N– carbons of the imidazolium ring, respectively. As anticipated, the ^29^Si CP-MAS NMR spectrum of 1b shows both T^*n*^ and Q^*n*^ sites. The peaks observed at −58 and −65 ppm can be unequivocally attributed to T^2^ [C–Si–(OSi)_2_(OH)] and T^3^ [C–Si–(OSi)_3_] sites within the framework, respectively. Furthermore, three peaks centered at −89, −100, and −109 ppm correspond to Q^2^ [Si–(OSi)_2_(OH)_2_], Q^3^ [Si–(OSi)_3_(OH)], and Q^4^ [Si–(OSi)_4_] units, respectively (Fig. 3b and S5, SI).^51^ The lack of any signal associated with T^1^-type Si species, coupled with the heightened signals originating from Q^3^ and Q^4^ silicon, clearly indicates the presence of densely condensed and cross-linked Si species in the materials, thereby suggesting the high stability of the framework in 1b. The loading capacity of ionic liquid bridges within the catalyst framework, as determined by elemental analysis, was estimated to be 0.89 mmol g^−1^. Transmission electron microscopy (TEM) images along and perpendicular to the mesochannels of the 1b catalyst are presented in Fig. 4a and b. These images clearly illustrate the uniform cylindrical structure of 1b with 2D hexagonal *P*6*mm* symmetry. The well-ordered structure of the catalyst was further validated through powder X-ray diffraction (PXRD) analysis. As shown in Fig. 4c, the low-angle PXRD diffraction pattern of 1b exhibits three distinct diffraction peaks indexed as *d*_100_, *d*_110_, and *d*_200_ reflections, which are indicative of materials with a well-organized 2D hexagonal structure and a *P*6*mm* space group. Both analyses are consistent with the findings from N_2_ adsorption–desorption analysis (Fig. 1). However, due to the low contrast TEM images, it was practically challenging to discern the presence of plugs in the channels even at high magnifications.

The thermal stability and organic functionalities content within the framework of 1b were assessed individually through thermogravimetric analysis (TGA) under both oxygen and nitrogen atmospheres (Fig. S6 and S7, SI). The TGA curve under an oxygen atmosphere exhibits three distinct weight losses in different temperature ranges. The initial weight loss of 5.5% below 125 °C is related to desorption of physiosorbed water and alcoholic solvents present on the surface of the material during the synthesis and purification processes. A subsequent weight loss of 2.7% between 125 °C and 300 °C is likely due to the removal of the surfactant residues and further condensation of silanol groups within the mesochannel wall. The primary weight loss of 23.3% occurring between 300 °C and 800 °C is linked to the decomposition of organic functional groups, such as ethyl, imidazolium, and propylsulfonic acid groups integrated into the material framework. These TGA results clearly underscore the exceptional thermal stability of 1b, even in the presence of an oxygen atmosphere.

After characterization of the 1b structure, the study proceeded to examine its efficiency in catalyzing the esterification of various alcohols and carboxylic acids. Optimization of the reaction parameters for the esterification of benzyl alcohol (1 mmol) with acetic acid (2 mmol) revealed specific reaction conditions: 1b (0.5 mol%), 50 °C, under solvent free conditions, for 16 h, yielding a 97% yield as determined by GC analysis (Table S2). The data presented in Table S2 demonstrated that altering the reaction parameters, such as reducing the catalyst amount, varying reaction temperature, or varying the reaction time, led to decreased reaction yields. It is also noteworthy that the esterification reaction conducted using equimolar amounts of benzyl alcohol and acetic acid did not reach completion even after extending the reaction time to 24 hours or increasing the reaction temperature to 80 °C (Table S2). It is crucial to highlight that the esterification reaction utilizing SBA-15-PrSO_3_H (2) and Et-PMO-Me-PrSO_3_H (3) exhibited significantly lower catalytic efficiency (Table S1). In both instances, a much higher molar ratio of acetic acid to benzyl alcohol (10 : 1), an increased catalyst loading of 5 mol% (almost tenfold compared to 1b in the present method), and elevated reaction temperature or prolonged reaction times were required to achieve satisfactory yields of the respective benzyl acetate ester.^24^ It is also noteworthy that under the optimized reaction conditions detailed in Table S2, both catalysts 2 and 3, as well as homogeneous H_2_SO_4_, produced less than 50% benzyl acetate ester even after 24 hours. Furthermore, catalyst 1b exhibits significantly superior catalytic efficiency compared to previous SBA-15-functionalized sulfonic acid catalyst systems, as demonstrated by the reduced catalyst amount, shorter reaction times, and improved recyclability.^63–65^ Given that the acid capacities of catalysts 1a–1d are essentially identical within experimental error, it can be concluded that the observed differences in catalytic activity do not arise from variations in acid capacity. We will return to this discussion later in the manuscript.

With the optimized reaction conditions established, we then explored the applicability of the catalyst in the esterification process involving various alcohols (4a–4m) with acetic acid to give the respective acetate esters (Table 2, 5a–5m). The results indicated that halo-substituted benzylic alcohols could be converted to the corresponding esters in excellent yields, without undergoing dehalogenation (Table 2, 5b–5d). Additionally, the catalytic system exhibited significant efficiency in the esterification of benzyl alcohol containing a strongly electron-withdrawing nitro group, leading to a good yield of the corresponding ester product (Table 2, 5e). Benzyl alcohol with an electron-donating methoxy group at the *para*-position also reacted efficiently under the present reaction conditions, giving rise to the respected acetate ester in excellent yields (Table 2, 5f). Notably, we found that using the present catalyst system, primary aliphatic alcohols were also capable of undergoing esterification, affording the corresponding esters in quantitative yields (Table 2, 5g–5k). Moreover, our catalytic system proved to be effective even in the esterification of secondary aliphatic alcohols, such as 2-octanol and cyclohexanol with acetic acid, albeit at an elevated temperature of 60 °C (Table 2, 5l, 5m). Motivated by the encouraging outcomes, we proceeded to further examine the efficacy of catalyst 1b in facilitating the esterification process of various carboxylic acids with ethanol. In this way, we explored the direct esterification of benzoic acid (6a, 1 mmol) with ethanol (1 mL) employing 1 mol% of 1b, which resulted in the formation of ethyl benzoate with a yield of 97% after 48 hours (Table 2, 7a). It is worth mentioning that while reactions utilizing 0.5 mol% of 1b exhibited good conversion rates (up 75%) within similar reaction times, they remained incomplete even after significantly prolonged reaction durations. This catalytic system proved to be effective for the esterification of 4-nitrobenzoic acid (6b) and 3-methoxybenzoic acid (6c) with ethanol, resulting in the corresponding ethyl esters in excellent yields after 24 hours (Table 2, 7b, and 7c). Notably, a lower catalyst amount (0.5 mol%) was sufficient for the completion of the 4-nitrobenzoic acid reaction, likely due to the strong electron-withdrawing effects of the nitro substituent, which activate the *para*-substituted carboxyl group. Furthermore, the system exhibited significant efficacy in the esterification of aliphatic carboxylic acids with ethanol (Table 2, 7d–7h). The ability of this system to tolerate long-chain aliphatic carboxylic acids, such as palmitic acid and stearic acid, may be attributed to the compromise between hydrophilic and hydrophobic nature within the mesochannels, as well as the capability of the catalyst's large pores to facilitate the mass transfer of long-chain molecules.

**Table 2:** Generality and scope of the esterification reaction catalyzed by Et-P-PMO-IL-SO_3_H (1b)^a^

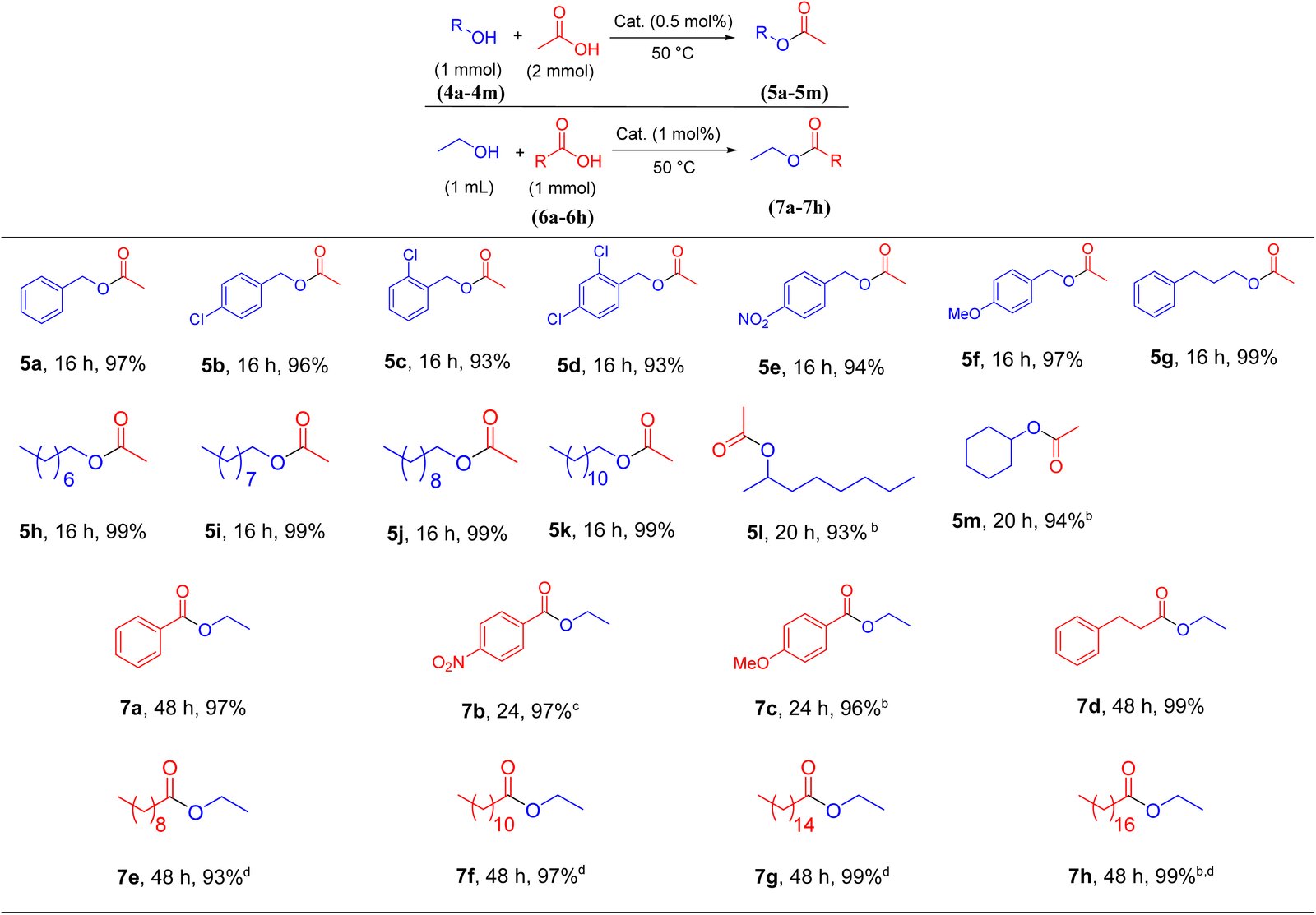

a Yields refer to GC yields.

b Reaction temperature = 60 °C.

c Catalyst amount = 0.5 mol%.

d Ethanol = 1.5 mL.

The outstanding performance of the 1b catalyst in the Biginelli reaction at a very low catalyst loading of 0.3 mol% (Fig. 2b) prompted us to make an assessment of the broad applicability and effectiveness of this approach in the synthesis of diverse DHPMs. This evaluation focused on the three-component reaction of a range of aldehyde substrates (8a–8p) in the presence of urea and methyl acetoacetate, as outlined in Table 3. The results demonstrated that aldehydes with electron-withdrawing groups at various positions on the benzene ring yielded the desired Biginelli adducts in high yields and selectivity while avoiding the formation of detectable levels of Hantzsch's ester by-products (Table 3, 9b–9g). A higher reaction temperature was required for 4-nitrobenzaldehyde, likely to enhance its solubility in the reaction environment (Table 3, 9f). Moreover, aldehydes with electron-donating substituents at *ortho*-, *meta*- or *para*-positions effectively underwent reaction with methyl acetoacetate and urea at 65 °C, resulting in the formation of the respective DHPMs with high isolated yields (Table 3, 9g–9l). The necessity for higher reaction temperatures in these instances can be ascribed to the reduced electrophilic properties of their carbonyl group in contrast to aldehydes containing electron-withdrawing groups. Notably, this procedure was suitable for the Biginelli reaction involving cinnamaldehyde, selected as an α,β-unsaturated substrate, in conjunction with methyl acetoacetate and urea under optimized reaction conditions, resulting in the corresponding product with high yield and selectivity (Table 3, 9m). The current system demonstrated an effective performance with 1-naphthaldehyde a relatively hindered aromatic aldehyde (Table 3, 9m). Additionally, DHPM adducts containing heterocyclic units were also produced with high selectivity and isolated yields when utilizing furan-2-carbaldehyde and 3-thiophene carbaldehyde (Table 3, 9o, 9p).

**Table 3:** Generality and scope of the Biginelli reaction catalyzed by Et-P-BFPMO-IL-PrSO_3_H (1b)^a^

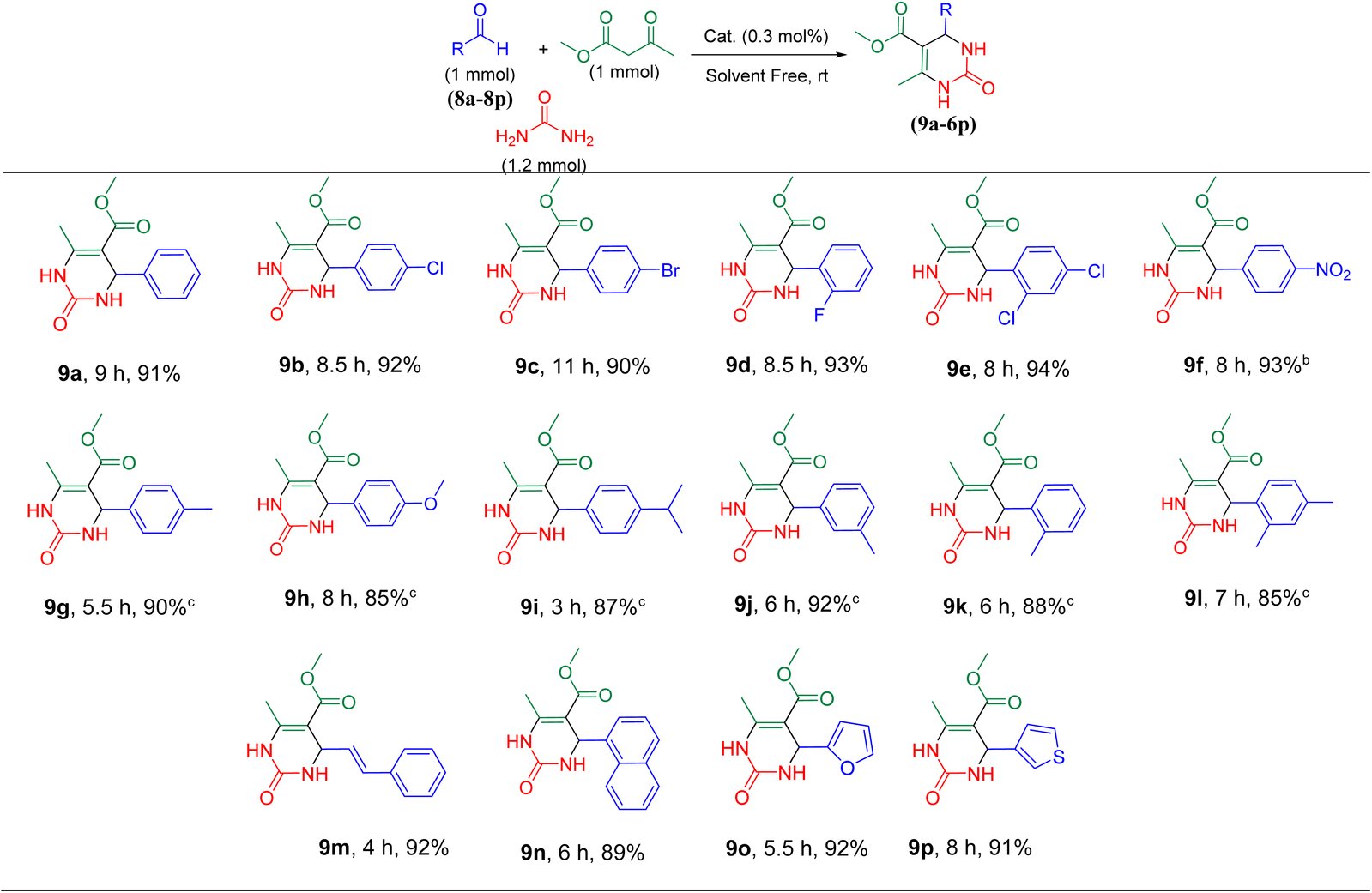

a Yields refer to the isolated yields.

b Reaction temperature = 80 °C.

c Reaction temperature = 65 °C.

**Scheme 2:**
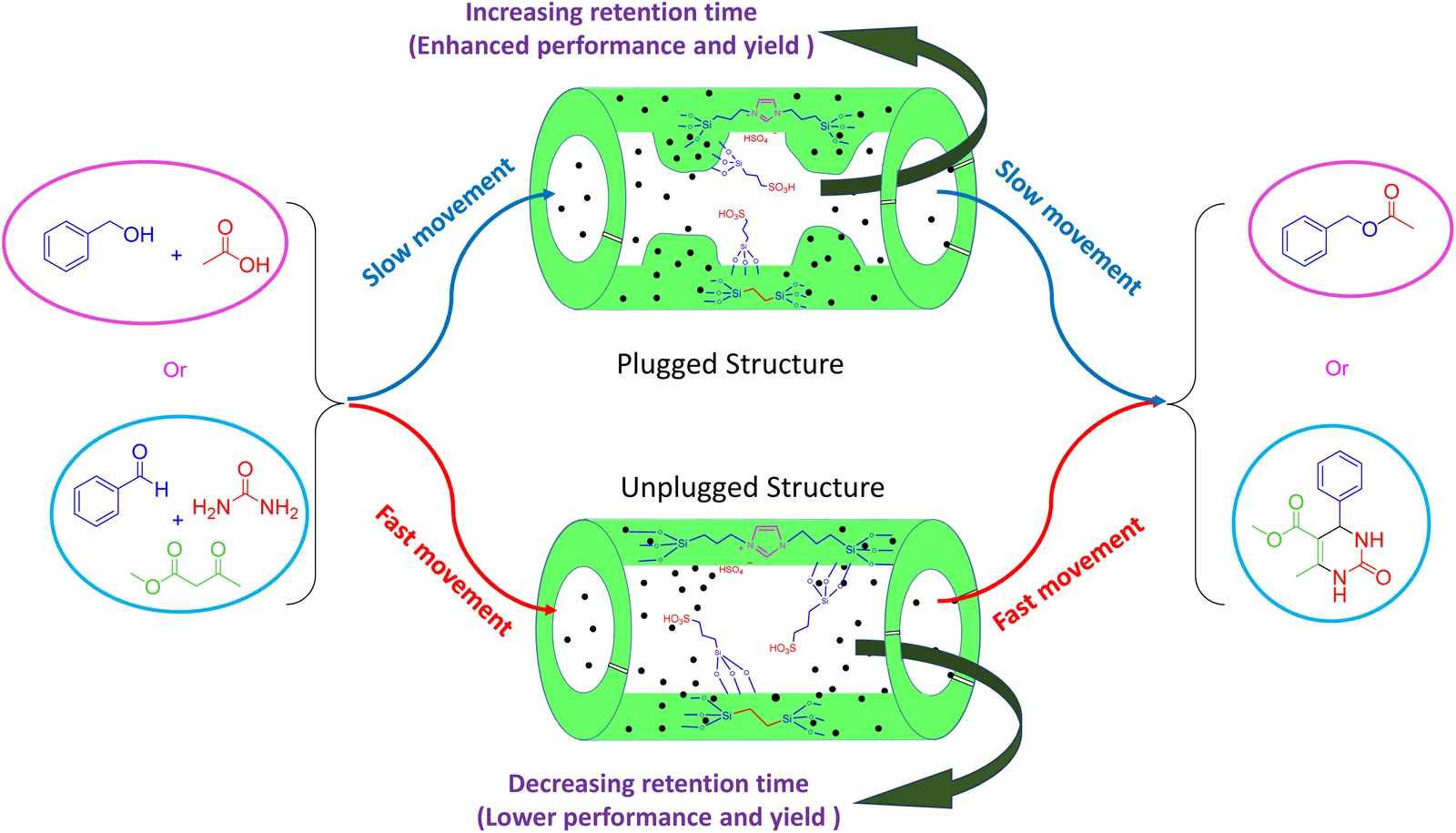
A proposed schematic representation of the reactions.

We proposed a schematic representation illustrating how plugs and organic bridges in the functionalized sulfonic acid mesoporous organosilica 1b synergistically contribute to its observed superior activity in both esterification and Biginelli reactions. It is suggested that the acidic site of catalyst 1b initially functions as a proton donor to the carboxylic acid group, thereby initiating the reaction pathway. Following the isomerization of the protonated acid, it becomes susceptible to nucleophilic attack by the alcohol. Subsequently, a hydrogen transfer process facilitates the removal of water, and the catalyst is regenerated *via* proton abstraction, ultimately resulting in the formation of the desired ester. Maintaining an optimal hydrophilic–hydrophobic balance within mesoporous catalysts is crucial, as it regulates the diffusion and transport of both polar and nonpolar reactants through their confined nanospaces. As illustrated in Fig. 2 and Table S3, comparison between the unplugged Et-U-BFPMO-IL-PrSO_3_H (1a) and the plugged Et-P-BFPMO-IL-PrSO_3_H (1b) systems clearly reveals that, while an appropriate concentration of ethylene bridges is essential for achieving acceptable conversion, the incorporation of plugs leads to a remarkable enhancement in catalytic efficiency. The key mechanistic question thus arises: how do the structural characteristics of these plugs and their interactions with the physicochemical environment inside the mesopores influence the observed reactivity? It is well established that plug formation typically occurs at high TEOS/template ratios during SBA-15 synthesis.^46,47,66,67^ Considering that we have previously and systematically investigated this concept using both plugged and unplugged bifunctional PMOs containing ethyl- or phenyl-imidazolium bridging units and demonstrated their effectiveness in adjusting the selectivity in alcohol oxidation reactions, it is highly plausible that a similar mechanism governs the present catalytic system as well.^49,52,53^ Hence, the plugs in 1b are plausibly composed of amorphous silica nanocapsules formed through the hydrolysis and condensation of excess TMOS. These nanocapsules, enriched with surface silanol groups, can create hydrophilic nano-domains embedded within the otherwise hydrophobic mesochannels of BFPMO-IL (Scheme 2). Such a dual nanoenvironment likely facilitates a complementary adsorption mechanism: hydrophilic regions attract polar reactants *via* hydrogen bonding, while the hydrophobic channel walls preferentially interact with nonpolar substrates. On the basis of our previous observations and analytical investigations of structurally related hybrid mesoporous organosilicas,^49,52,53^ we propose that this cooperative microenvironment, combined with the confinement effects arising from the plug-generated regions, promotes prolonged residence times of reactants near the acidic active sites, thereby facilitating reaction progression and enhancing overall yields. Conversely, the unplugged catalyst 1a, with its more open mesoporous architecture, enables faster reactant diffusion and weaker confinement effects, leading to less effective interaction with the active centers and consequently lower product yields under identical conditions (Scheme 2). Taken together, these observations highlight a synergistic interplay among the ethylene bridges (15%), imidazolium moieties, and optimally distributed plugs within the mesochannels, which likely creates a finely tuned microenvironment that maximizes catalytic turnover and selectivity through a cooperative mechanism. Nevertheless, comprehensive mechanistic investigations involving theoretical calculations and kinetic and additional surface analyses are currently in progress in our laboratories to gain deeper insight into the origin of the selectivity differences between the plugged and unplugged catalyst systems reported in this study.

**Fig. 5:**
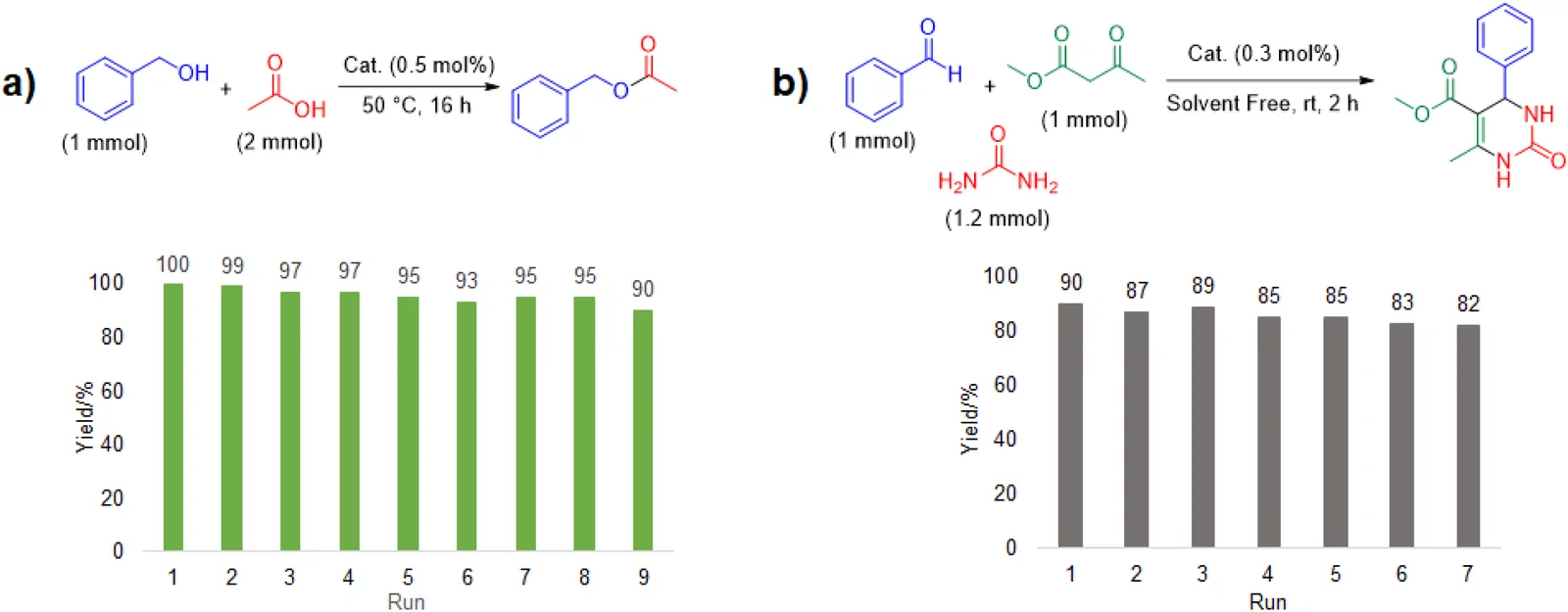
Reusability of the 1b catalyst in (a) the esterification and (b) Biginelli model reactions.

**Fig. 6:**
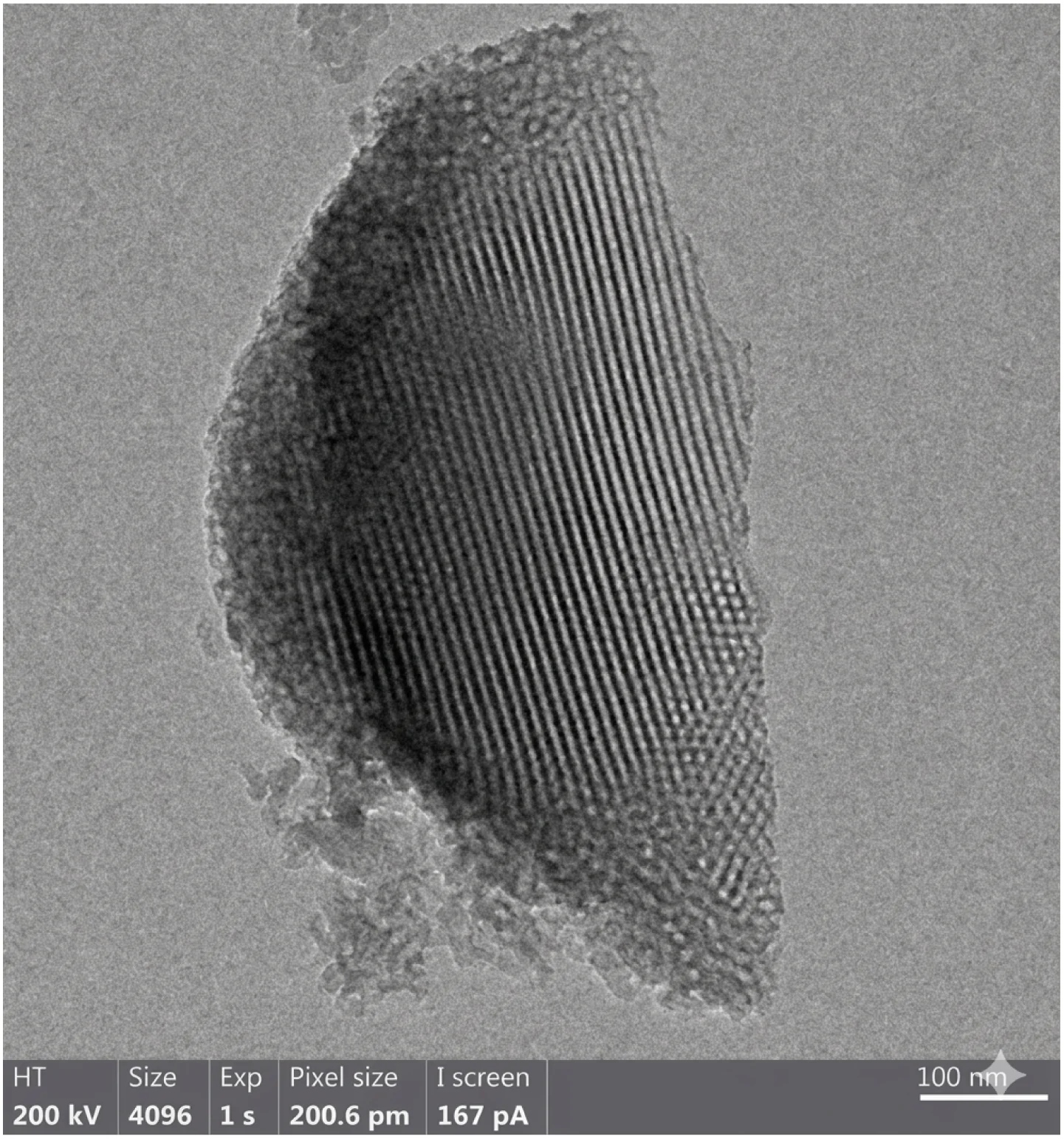
TEM image of the recovered 1b catalyst from the esterification reaction.

An essential consideration concerning heterogeneous catalysts is their reusability. The 1b catalyst's reusability was assessed by utilizing it in consecutive cycles for both the esterification and Biginelli model reactions, following the procedures outlined in the Experimental section (Fig. 5). Gratifyingly, the catalyst can be recovered and reused in multiple successive runs in both catalytic systems without a substantial decrease in activity. The recovered catalyst retained essentially the same acid capacity as the fresh catalyst, as confirmed by titration analysis. The outcome clearly shows the remarkable stability and endurance of the catalyst under the current reaction conditions. Moreover, the examination of the structure and morphology of the recovered catalyst was conducted through N_2_ sorption and TEM analyses. The N_2_ sorption isotherm of the specimen exhibited a Type IV profile with a two-step desorption branch, containing a desorption step at high relative pressure, which corresponds to the release of N_2_ from open mesopores, and a second delayed desorption step at a relative pressure of about ∼0.45, related to N_2_ release from partially blocked pores. This characteristic feature indicative of plugged mesoporous structures closely resembles that of the fresh catalyst, thereby validating the retention of the material's structural integrity (Fig. S8–S10). The specific surface area (*S*_BET_) and total pore volume (*V*_t_) of the sample were measured to be 335 m^2^ g^−1^ and 0.63 cm^3^ g^−1^, respectively. Furthermore, the DH pore size distribution curve of the reused catalyst revealed two distinct peaks (Fig. S10), which closely matched those of the initial catalyst. The TEM image of the specimen corroborated the preservation of the catalyst's structural integrity and order (Fig. 6), displaying a uniform cylindrical morphology with well-organized 2D hexagonal *P*6*mm* symmetry. However, due to the low contrast in the TEM image, detecting plugs in the channels was again challenging even at high magnifications.

## Conclusion

In summary, a series of acid-functionalized plugged/unplugged bifunctional periodic mesoporous organosilicas (P/U BFPMO-IL-PrSO_3_Hs, 1a–1d) incorporating ethyl/imidazolium or phenyl/imidazolium bridging groups were successfully synthesized. Controlled precursor addition was found to play a decisive role in the formation of plugged structures, thereby strongly influencing the textural and catalytic properties of the resulting materials. Among the prepared catalysts, the plugged ethyl-imidazolium-bridged material (Et-P-BFPMO-IL-PrSO_3_H, 1b) exhibited the highest catalytic performance in both the esterification of a broad range of benzylic and aliphatic alcohols/acids and the Biginelli condensation of aldehydes under mild and solvent-free conditions, affording the desired products in excellent yields and selectivities. Its superior performance is likely associated with the synergistic nanoenvironment generated by the combined presence of organic bridges and silica plugs within the mesochannels. The plugs create hydrophilic domains enriched with silanol groups, whereas the mesochannel walls remain relatively hydrophobic, thereby facilitating efficient interactions with both hydrophilic and hydrophobic substrates. In addition, the confinement effects induced by the plugs promote prolonged residence times of reactants near the acidic active sites, ultimately enhancing catalytic efficiency and selectivity. Furthermore, the catalyst demonstrated excellent recyclability and structural stability over multiple reaction cycles. Overall, this study highlights the critical role of controlled nanoscale architecture in the design of efficient, robust, and sustainable solid acid catalysts.

## Author contributions

Hesamoddin Moradi and Yaser Maleki synthesized and characterized the catalyst materials, performed the catalytic experiments, and carried out the catalytic performance evaluations. These authors contributed equally to this work. This research forms part of their M.Sc. dissertations at the Institute for Advanced Studies in Basic Sciences (IASBS). Nasim Ganji and Omid Pourshiani contributed to the experimental work and assisted in the preparation of the parent catalyst materials. Nasim Ganji and Hesamoddin Moradi prepared the initial draft of the manuscript. Babak Karimi conceived the original idea, designed and conceptualized the study, secured funding, supervised the project, interpreted the results, and prepared the final version of the manuscript. Hojatollah Vali provided financial support for the TEM and HRTEM analyses and contributed to the interpretation of the microscopy data. Piero Mastrorilli and Stefano Todisco provided financial support for the solid-state CP-MAS NMR analyses and contributed to the interpretation of the spectroscopic results. All authors discussed the results, contributed to the scientific interpretation of the data, partly reviewed the manuscript, and approved the final version.

## Conflicts of interest

There are no conflicts to declare.

## Supplementary Material

NA-008-D5NA01099C-s001

## Data Availability

The data supporting this article have been included as part of the supplementary information (SI). Supplementary information: NMR data, TGA, DH curves, optimization tables, *etc.* See DOI: https://doi.org/10.1039/d5na01099c.
