# Supplementary material for: Rational plug–bridge design to enhance catalytic efficiency of sulfonic acid-functionalized plugged ethyl/imidazolium-based bifunctional periodic mesoporous organosilica
Source: Nanoscale Adv. 2026 Jun 26;8(17):4921–34. doi: 10.1039/d5na01099c (PMC13445202; doi:10.1039/d5na01099c)
Supplement: Supplementary file 1 [file NA-008-D5NA01099C-s001.pdf]

Supplementary Information (SI) for Nanoscale Advances.
This journal is © The Royal Society of Chemistry 2026

Supporting Information

Rational Plug–Bridge Design to Enhance Catalytic Efficiency of Sulfonic Acid-Functionalized Plugged Ethyl/Imidazolium-based Bifunctional Periodic Mesoporous Organosilica (Et-P-BFPMO-IL-PrSO₃H)

Figure S1. Dollimore-Heal (DH) pore size distribution curves for **1a** (red) and **1b** (blue). 10
Figure S2. Dollimore-Heal (DH) pore size distribution curves for **1c** (red) and **1d** (green).P-Ph-PMO-IL-SO_3H and U-Ph-PMO-IL-SO_3H 10
Figure S3. FTIR spectrum of the **1b** catalyst 11
Figure S4. TGA curve of the **1b** catalyst under an oxygen atmosphere 12
Figure S5. TGA curve of the **1b** catalyst under a nitrogen atmosphere........................12
Figure S6. N_2 adsorption-desorption curve of the recovered **1b** catalyst 13
Figure S7. BJH pore size distribution curve of the recovered **1b** catalyst........................13
Figure S8. DH pore size distribution curve of the recovered **1b** catalyst 13
Figure S9. EDS spectrum of **1b** catalyst 14
Figure S10. TEM image of the **1b** catalyst, perpendicular to the mesochannels (scale bar: 50 nm)........14
Figure S11. TEM image of the **1b** catalyst, along to the mesochannels (scale bar: 50 nm)........................14
Figure S12. ^{1}H NMR spectrum of the ethyl benzoate (**5a**) product in $CDCl_3$........................15
Figure S13. ^{13}C NMR spectrum of the ethyl benzoate (**5a**) product in $CDCl_3$........................15
Figure S14. ^{1}H NMR spectrum of the 4-chlorobenzyl acetate (**5b**) product in $CDCl_3$........................16
Figure S15. ^{13}C NMR spectrum of the 4-chlorobenzyl acetate (**5b**) product in $CDCl_3$........................16
Figure S16. ^{1}H NMR spectrum of the 2-chlorobenzyl acetate (**5c**) product in $CDCl_3$........................17
Figure S17. ^{13}C NMR spectrum of the 2-chlorobenzyl acetate (**5c**) product in $CDCl_3$........................17
Figure S18. ^{1}H NMR spectrum of the 2,4-dichlorobenzyl acetate (**5d**) product in $CDCl_3$........................18
Figure S19. ^{13}C NMR spectrum of the 2,4-dichlorobenzyl acetate (**5d**) product in $CDCl_3$........................18
Figure S20. ^{1}H NMR spectrum of the 4-nitrobenzyl acetate (**5e**) product in $CDCl_3$........................19
Figure S21. ^{13}C NMR spectrum of the 4-nitrobenzyl acetate (**5e**) product in $CDCl_3$........................19
Figure S22. ^{1}H NMR spectrum of the 4-methoxybenzyl acetate (**5f**) product in $CDCl_3$........................20
Figure S23. ^{13}C NMR spectrum of the 4-methoxybenzyl acetate (**5f**) product in $CDCl_3$........................20
Figure S24. ^{1}H NMR spectrum of the 3-phenylpropyl acetate (**5g**) product in $CDCl_3$........................21
Figure S25. ^{13}C NMR spectrum of the 3-phenylpropyl acetate (**5g**) product in $CDCl_3$........................21
Figure S26. ^{1}H NMR spectrum of the octyl acetate (**5h**) product in $CDCl_3$........................22
Figure S27. ^{13}C NMR spectrum of the octyl acetate (**5h**) product in $CDCl_3$........................22
Figure S28. ^{1}H NMR spectrum of the nonyl acetate (**5i**) product in $CDCl_3$........................23
Figure S29. ^{13}C NMR spectrum of the nonyl acetate (**5i**) product in $CDCl_3$........................23
Figure S30. ^{1}H NMR spectrum of the decyl acetate (**5j**) product in $CDCl_3$........................24
Figure S31. ^{13}C NMR spectrum of the decyl acetate (**5j**) product in $CDCl_3$........................24
Figure S32. ^{1}H NMR spectrum of the dodecyl acetate (**5k**) product in $CDCl_3$........................25
Figure S33. ^{13}C NMR spectrum of the dodecyl acetate (**5k**) product in $CDCl_3$........................25
Figure S34. ^{1}H NMR spectrum of the 2-octyl acetate product (**5l**) in $CDCl_3$........................26
Figure S35. ^{13}C NMR spectrum of the 2-octyl acetate product (**5l**) in $CDCl_3$........................26
Figure S36. ^{1}H NMR spectrum of the cyclohexyl acetate (**5m**) product in $CDCl_3$........................27
Figure S37. ^{13}C NMR spectrum of the cyclohexyl acetate (**5m**) product in $CDCl_3$........................27
Figure S38. ^{1}H NMR spectrum of the ethyl benzoate (**7a**) product in $CDCl_3$........................28

Figure S39. ^{13}C NMR spectrum of the ethyl benzoate (**7a**) product in $CDCl_3$ 28
Figure S40. ^{1}H NMR spectrum of the ethyl 4-nitrobenzoate (**7b**) product in $CDCl_3$ 28
Figure S41. ^{13}C NMR spectrum of the ethyl 4-nitrobenzoate (**7b**) product in $CDCl_3$ 29
Figure S42. ^{1}H NMR spectrum of the ethyl 2-methoxybenzoate (**7c**) product in $CDCl_3$ 29
Figure S43. ^{13}C NMR spectrum of the ethyl 2-methoxybenzoate (**7c**) product in $CDCl_3$ 30
Figure S44. ^{1}H NMR spectrum of the ethyl 3-phenylpropanoate (**7d**) product in $CDCl_3$ 30
Figure S45. ^{13}C NMR spectrum of the ethyl 3-phenylpropanoate (**7d**) product in $CDCl_3$ 31
Figure S46. ^{1}H NMR spectrum of the ethyl decanoate (**7e**) product in $CDCl_3$ 31
Figure S47. ^{13}C NMR spectrum of the ethyl decanoate (**7e**) product in $CDCl_3$ 32
Figure S48. ^{1}H NMR spectrum of the ethyl dodecanoate (**7f**) product in $CDCl_3$ 32
Figure S49. ^{13}C NMR spectrum of the ethyl dodecanoate (**7f**) product in $CDCl_3$ 33
Figure S50. ^{1}H NMR spectrum of the ethyl hexadecanoate (**7g**) product in $CDCl_3$ 33
Figure S51. ^{13}C NMR spectrum of the ethyl hexadecanoate (**7g**) product in $CDCl_3$ 34
Figure S52. ^{1}H NMR spectrum of the ethyl octadecanoate (**7h**) product in $CDCl_3$ 34
Figure S53. ^{13}C NMR spectrum of the ethyl octadecanoate (**7h**) product in $CDCl_3$ 35
Figure S54. FTIR spectrum of the methyl 6-methyl-2-oxo-4-phenyl-1,2,3,4-tetrahydropyrimidine-5-carboxylate (**9a**) product 35
Figure S55. ^{1}H NMR spectrum of the methyl 6-methyl-2-oxo-4-phenyl-1,2,3,4-tetrahydropyrimidine-5-carboxylate (**9a**) product in DMSO 36
Figure S56. ^{13}C NMR spectrum of the methyl 6-methyl-2-oxo-4-phenyl-1,2,3,4-tetrahydropyrimidine-5-carboxylate (**9a**) product in DMSO 36
Figure S57. FTIR spectrum of the 4-(4-chlorophenyl)-6-methyl-2-oxo-1,2,3,4-tetrahydropyrimidine -5-carboxylate (**9b**) product 37
Figure S58. ^{1}H NMR spectrum of Methyl 4-(4-chlorophenyl)-6-methyl-2-oxo-1,2,3,4-tetrahydropyrimidine -5-carboxylate (**9b**) product in DMSO 37
Figure S59. ^{13}C NMR spectrum of Methyl 4-(4-chlorophenyl)-6-methyl-2-oxo-1,2,3,4-tetrahydropyrimidine-5-carboxylate (**9b**) product in DMSO 38
Figure S60. FTIR spectrum of the methyl 4-(4-boromophenyl)-6-methyl-2-oxo-1,2,3,4-tetrahydropyrimidine -5-carboxylate (**9c**) product 38
Figure S61. ^{1}H NMR spectrum of the methyl 4-(4-boromophenyl)-6-methyl-2-oxo-1,2,3,4-tetrahydropyrimidine -5-carboxylate (**9c**) product in DMSO 39
Figure S62. FTIR spectrum of the methyl 4-(2-fluorophenyl)-6-methyl-2-oxo-1,2,3,4 tetrahydropyrimidine-5-carboxylate (**9d**) product. 39
Figure S63. ^{1}H NMR spectrum of the methyl 4-(2-fluorophenyl)-6-methyl-2-oxo-1,2,3,4 tetrahydropyrimidine-5-carboxylate (**9d**) product in DMSO 40
Figure S64. ^{13}C NMR spectrum of the methyl 4-(2-fluorophenyl)-6-methyl-2-oxo-1,2,3,4 tetrahydropyrimidine-5-carboxylate (**9d**) product in DMSO 40
Figure S65. FTIR spectrum of the methyl 4-(2,4-dicholorophenyl)-6-methyl-2-oxo-1,2,3,4-tetrahydropyrimidine -5-carboxylate (**9e**) product 41
Figure S66. ^{1}H NMR spectrum of the methyl 4-(2,4-dicholorophenyl)-6-methyl-2-oxo-1,2,3,4-tetrahydropyrimidine -5-carboxylate (**9e**) product in DMSO 41
Figure S67. ^{13}C NMR spectrum of the methyl 4-(2,4-dicholorophenyl)-6-methyl-2-oxo-1,2,3,4 tetrahydropyrimidine-5-carboxylate (**9e**) product in DMSO 42
Figure S68. FTIR spectrum of the methyl 4-(4-nitrophenyl)-6-methyl-2-oxo-1,2,3,4-tetrahydropyrimidine-5-carboxylate (**9f**) product 42
Figure S69. ^{1}H-NMR spectrum of the methyl 4-(4-nitrophenyl)-6-methyl-2-oxo-1,2,3,4-tetrahydropyrimidine-5-carboxylate (**9f**) product in DMSO 43

Figure S70. ^{13}C NMR spectrum of the methyl 4-(4-nitrophenyl)-6-methyl-2-oxo-1,2,3,4-tetrahydropyrimidine-5-carboxylate (**9f**) product in DMSO 43
Figure S71. FTIR spectrum of the methyl 6-methyl-2-oxo-4-p-tolyl-1,2,3,4-tetrahydropyrimidine-5-carboxylate (**9g**) product 44
Figure S72. ^{1}H NMR spectrum of the methyl 6-methyl-2-oxo-4-p-tolyl-1,2,3,4-tetrahydropyrimidine-5-carboxylate (**9g**) product in DMSO 44
Figure S73. ^{13}C NMR spectrum of the methyl 6-methyl-2-oxo-4-p-tolyl-1,2,3,4-tetrahydropyrimidine-5-carboxylate (**9g**) product in DMSO 45
Figure S74. FTIR spectrum of the methyl 4-(4-methoxyphenyl)-6-methyl-2-oxo-1,2,3,4-tetrahydropyrimidine-5-carboxylate (**9h**) product 45
Figure S75. ^{1}H NMR spectrum of the methyl 4-(4-methoxyphenyl)-6-methyl-2-oxo-1,2,3,4-tetrahydropyrimidine-5-carboxylate (**9h**) product in DMSO 46
Figure S76. ^{13}C NMR spectrum of the methyl 4-(4-methoxyphenyl)-6-methyl-2-oxo-1,2,3,4-tetrahydropyrimidine-5-carboxylate (**9h**) product in DMSO 46
Figure S77. FTIR spectrum of the methyl 4-(4-isopropylphenyl)-6-methyl-2-oxo-1,2,3,4-tetrahydropyrimidine-5-carboxylate (**9i**) product 47
Figure S78. ^{1}H NMR spectrum of the methyl 4-(4-isopropylphenyl)-6-methyl-2-oxo-1,2,3,4-tetrahydropyrimidine-5-carboxylate (**9i**) product in DMSO 47
Figure S79. ^{13}C NMR spectrum of the methyl 4-(4-isopropylphenyl)-6-methyl-2-oxo-1,2,3,4-tetrahydropyrimidine-5-carboxylate (**9i**) product in DMSO 48
Figure S80. FTIR spectrum of the methyl 6-methyl-2-oxo-4-m-tolyl-1,2,3,4-tetrahydropyrimidine-5-carboxylate (**9j**) product 48
Figure S81. ^{1}H NMR spectrum of the methyl 6-methyl-2-oxo-4-m-tolyl-1,2,3,4-tetrahydropyrimidine-5-carboxylate (**9j**) product in DMSO 49
Figure S82. ^{13}C NMR spectrum of the methyl 6-methyl-2-oxo-4-m-tolyl-1,2,3,4-tetrahydropyrimidine-5-carboxylate (**9j**) product in DMSO 49
Figure S83. FTIR spectrum of the methyl 6-methyl-2-oxo-4-o-tolyl-1,2,3,4-tetrahydropyrimidine-5-carboxylate (**9k**) product. 50
Figure S84. ^{1}H NMR spectrum of the methyl 6-methyl-2-oxo-4-o-tolyl-1,2,3,4-tetrahydropyrimidine-5-carboxylate (**9k**) product in DMSO 50
Figure S85. ^{13}C NMR spectrum of the methyl 6-methyl-2-oxo-4-o-tolyl-1,2,3,4-tetrahydropyrimidine-5-carboxylate (**9k**) product in DMSO 51
Figure S86. FTIR spectrum of the methyl 4-(2,4-dimethylphenyl)-6-methyl-2-oxo-1,2,3,4-tetrahydropyrimidine-5-carboxylate (**9l**) product 51
Figure S87. ^{1}H NMR spectrum of the methyl 4-(2,4-dimethylphenyl)-6-methyl-2-oxo-1,2,3,4-tetrahydropyrimidine-5-carboxylate (**9l**) product in DMSO 52
Figure S88. ^{13}C NMR spectrum of the methyl 4-(2,4-dimethylphenyl)-6-methyl-2-oxo-1,2,3,4-tetrahydropyrimidine-5-carboxylate (**9l**) product in DMSO 52
Figure S89. FTIR spectrum of the methyl 6-methyl-2-oxo-4-styryl-1,2,3,4-tetrahydropyrimidine -5-carboxylate (**9m**) product 53
Figure S90. ^{1}H NMR spectrum of the methyl 6-methyl-2-oxo-4-styryl-1,2,3,4-tetrahydropyrimidine -5-carboxylate (**9m**) product in DMSO 53
Figure S91. ^{13}C NMR spectrum of the methyl 6-methyl-2-oxo-4-styryl-1,2,3,4-tetrahydropyrimidine -5-carboxylate (**9m**) product in DMSO 54
Figure S92. FTIR spectrum of the methyl 6-methyl-4-(naphthalene-1-yl)-2-oxo-1,2,3,4-tetrahydropyrimidine -5-carboxylate (**9n**) product 54

Figure S93. ^{1}H NMR spectrum of the methyl 6-methyl-4-(naphthalene-1-yl)-2-oxo-1,2,3,4-tetrahydropyrimidine -5-carboxylate (**9n**) product in DMSO 55
Figure S94. ^{13}C NMR spectrum of the methyl 6-methyl-4-(naphthalene-1-yl)-2-oxo-1,2,3,4-tetrahydropyrimidine -5-carboxylate (**9n**) product in DMSO 55
Figure S95. FTIR spectrum of the methyl 4-(4-furan-2-yl)-6-methyl-2-oxo-1,2,3,4-tetrahydropyrimidine-5-carboxylate (**9o**) product....................56
Figure S96. ^{1}H NMR spectrum of the methyl 4-(4-furan-2-yl)-6-methyl-2-oxo-1,2,3,4-tetrahydropyrimidine-5-carboxylate (**9o**) product in DMSO 56
Figure S97. ^{13}C NMR spectrum of the methyl 4-(4-furan-2-yl)-6-methyl-2-oxo-1,2,3,4-tetrahydropyrimidine-5-carboxylate (**9o**) product in DMSO 57
Figure S98. FTIR spectrum of the methyl 4-(4-thiol-3-yl)-6-methyl-2-oxo-1,2,3,4-tetrahydropyrimidine-5-carboxylate (**9p**) product 57
Figure S99. ^{1}H NMR spectrum of the methyl 4-(4-thiol-3-yl)-6-methyl-2-oxo-1,2,3,4-tetrahydropyrimidine -5-carboxylate (**9p**) product in DMSO 58
Figure S100. ^{13}C NMR spectrum of the methyl 4-(4-thiol-3-yl)-6-methyl-2-oxo-1,2,3,4-tetrahydropyrimidine -5-carboxylate (**9p**) product in DMSO 58
Figure S101. Gas chromatogram of the solution containing 0.25 mmol benzyl alcohol and 0.75 mmol TMB-first injection 59
Figure S102. Gas chromatogram of the solution containing 0.25 mmol benzyl alcohol and 0.75 mmol TMB-second injection 59
Figure S103. Gas chromatogram of the solution containing 0.5 mmol benzyl alcohol and 0.5 mmol TMB-first injection 60
Figure S104. Gas chromatogram of the solution containing 0.5 mmol benzyl alcohol and 0.5 mmol TMB-second injection 60
Figure S105. Gas chromatogram of the solution containing 0.75 mmol benzyl alcohol and 0.25 mmol TMB-first injection 61
Figure S106. Gas chromatogram of the solution containing 0.75 mmol benzyl alcohol and 0.25 mmol TMB-second injection 61
Figure S107. Gas chromatogram of the solution containing 0.25 mmol benzyl alcohol and 0.75 mmol benzyl acetate-first injection 62
Figure S108. Gas chromatogram of the solution containing 0.25 mmol benzyl alcohol and 0.75 mmol benzyl acetate-second injection 63
Figure S109. Gas chromatogram of the solution containing 0.5 mmol benzyl alcohol and 0.5 mmol benzyl acetate-first injection 63
Figure S110. Gas chromatogram of the solution containing 0.5 mmol benzyl alcohol and 0.5 mmol benzyl acetate-second injection 64
Figure S111. Gas chromatogram of the solution containing 0.75 mmol benzyl alcohol and 0.25 mmol benzyl acetate-first injection 64
Figure S112. Gas chromatogram of the solution containing 0.75 mmol benzyl alcohol and 0.25 mmol benzyl acetate-second injection 65
Figure S113. GC Results considering TMB and benzyl alcohol peaks after 4 h 66
Figure S114. GC Results considering benzyl acetate and benzyl alcohol peaks after 4 h 66
Figure S115. GC Results considering TMB, benzyl acetate and benzyl alcohol peaks after 4 h 67
Figure S116. GC Results considering TMB and benzyl alcohol peaks after 8 h 68
Figure S117. GC Results considering benzyl acetate and benzyl alcohol peaks after 8 h 68
Figure S118. GC Results considering TMB, benzyl acetate and benzyl alcohol peaks after 8 h 69
Figure S119. GC Results considering TMB and benzyl alcohol peaks after 12 h 69

Figure S120. GC Results considering benzyl acetate and benzyl alcohol peaks after 12 h........................70
Figure S121. GC Results considering TMB, benzyl acetate and benzyl alcohol peaks after 12 h............70

Experimental Sections

Chemicals and Instruments

All chemicals used in this project were purchased from Merck, Aldrich and Fluka Companies. The textural characteristics of the synthesized materials were determined by N_2 adsorption-desorption analysis at a temperature of 77 K, employing a Belsorp-max (BELMAX, Japan) analyzer. Before analysis, the samples were subjected to a 12-hour degassing process at 373 K. The specific surface areas were calculated using the Brunauer-Emmett-Teller (BET) equation. The pore size distribution curves were obtained using the Barrett-Joyner-Halenda (BJH) and Dollimore-Heal (DH) methods with data derived from the adsorption and desorption branches of the isotherms, respectively. Total pore volumes were estimated by evaluating the amount of nitrogen gas adsorbed at $P/P_0 \approx 0.95$. Transmission electron microscopic (TEM) images were taken by a Philips CM-200 electron microscope, operated at an accelerated voltage of 120 kV. Powder X-ray diffraction patterns were acquired by a Powder X-ray diffractometer (STOE StadiP) within a range spanning from 1° to 10°. Thermogravimetric analysis was performed using a NETZSCH STA 409 PC/PG instrument at a scan rate of 20 $K.min^{-1}$ under an oxygen flow. DRIFT spectra were recorded on a Bruker vector 22 spectrometer in the 400-4000 cm^{-1} region. Elemental analyses for C, N, S, and H were performed using an EL Vario CHNS analyzer. Progress of reactions was monitored using thin layer chromatography (TLC) on silica-gel polygram SIL UV 254 plates. Gas chromatography analyses were conducted using a Varian CP-3800 equipped with an S10 flame ionization detector (FID). Nuclear magnetic resonance (NMR) spectra were recorded using a Bruker instrument (^1H frequency: 400 MHz, ^{13}C frequency: 100 MHz).

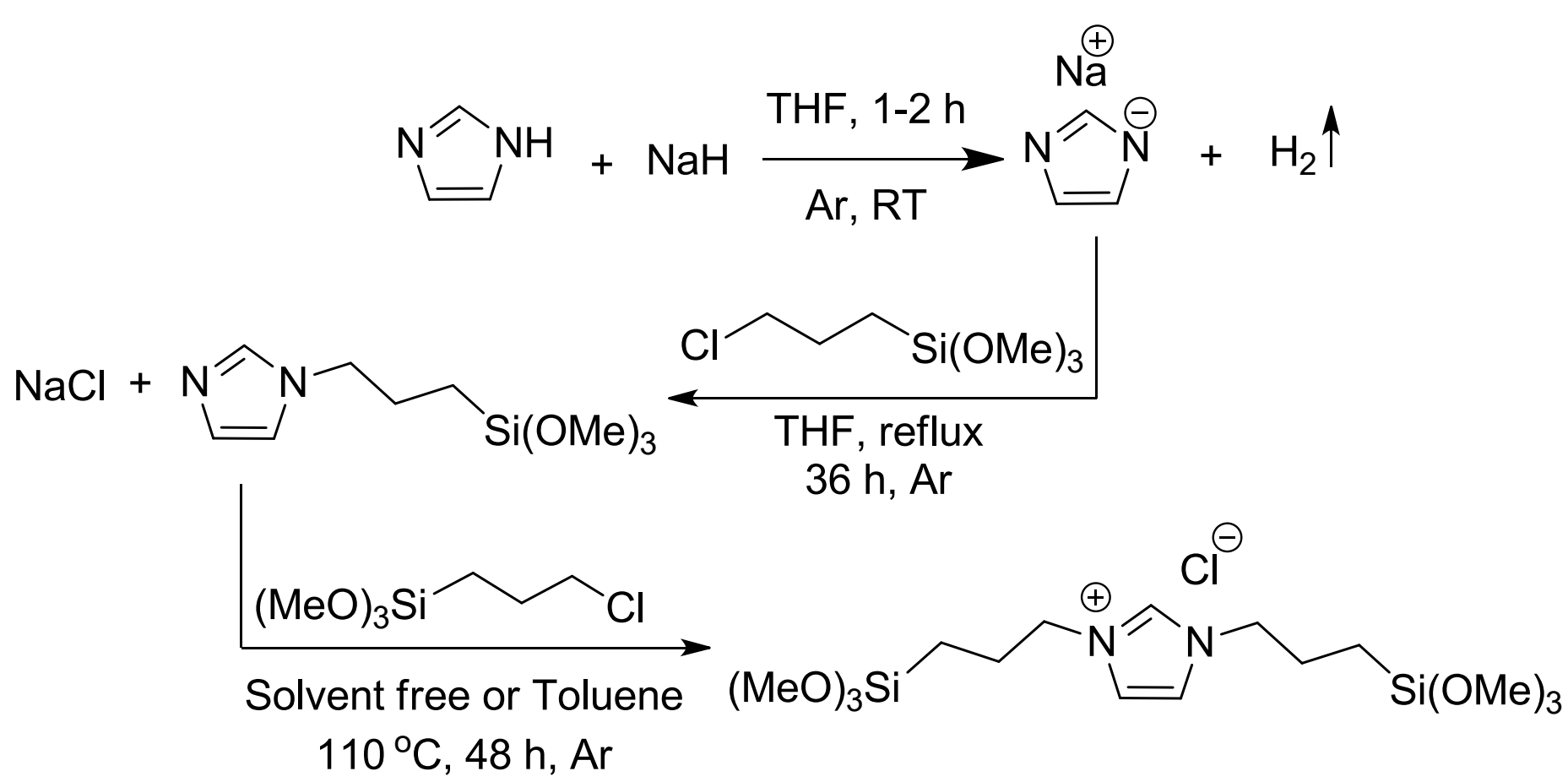

Scheme S1. Schematic synthesis of 1,3-bistrimethoxysilyl propyl imidazolium chloride

NMR data of esterification products:

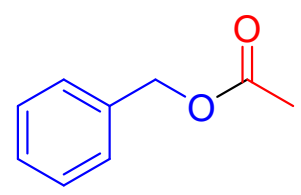

Benzyl acetate (5a).^{1}H NMR (400 MHz, $CDCl_3$): δ_H = 7.40 (m, 5H), 5.16 (s, 2H), 2.15 (s, 3H); ^{13}C NMR (101 MHz, $CDCl_3$): δ_C = 171.7, 136.8, 129.4, 129.1, 129.1, 67.2, 21.9.

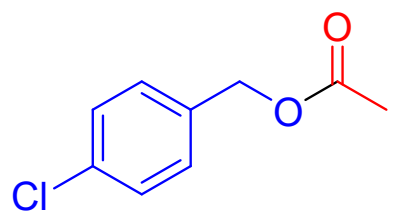

4-Chloro benzyl acetate (5b). ^{1}H NMR (400 MHz, $CDCl_3$): δ_H = 7.36 (d, J = 8.2 Hz, 2H), 7.23 (d, J = 8.2 Hz, 2H), 5.16 (s, 2H), 2.13 (s, 3H); ^{13}C NMR (101 MHz, $CDCl_3$): δ_C = 170.7, 134.5, 134.1, 129.6, 128.7, 65.4, 20.9.

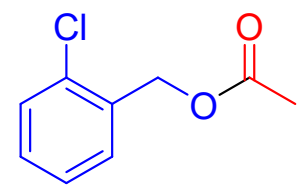

2-Chloro benzyl acetate (5c). ^{1}H NMR (400 MHz, $CDCl_3$): δ_H = 7.33 (m, 4H), 5.16 (s, 2H), 2.13 (s, 3H); ^{13}C NMR (101 MHz, $CDCl_3$): δ_C = 170.5, 134.6, 134.3, 132.3, 130.6, 129.3, 127.2, 62.9, 20.8.

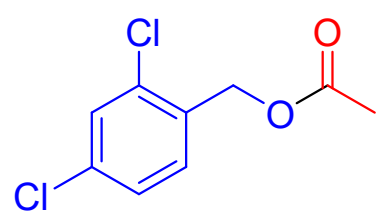

2,4-Dichloro benzyl acetate (5d). ^{1}H NMR (400 MHz, $CDCl_3$): δ_H = 7.43 (m, 1H), 7.29 (d, J = 5.9 Hz, 2H), 5.24 (s, 2H), 2.15 (s, 3H); ^{13}C NMR (101 MHz, $CDCl_3$): δ_C = 170.5, 134.6, 134.3, 132.3, 130.6, 129.3, 127.3, 62.9, 20.8.

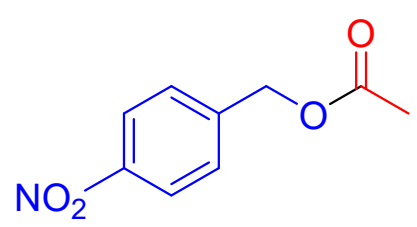

4-Nitrobenzyl acetate (5e). ^{1}H- NMR (400 MHz, $CDCl_3$): δ_H = 8.18 (d, J = 8.6 Hz, 2H), 7.51 (d, J = 8.6 Hz, 2H), 5.19 (s, 2H), 2.14 (s, 3H); ^{13}C NMR (101 MHz, $CDCl_3$): δ_C = 170.5, 147.6, 143.2, 128.3, 123.7, 64.7, 20.8.

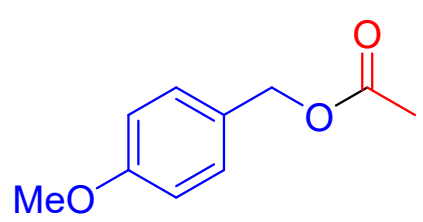

4-Methoxybenzyl acetate (5f). ^{1}H NMR (400 MHz, $CDCl_3$): δ_H = 7.34 (d, J = 8.7 Hz, 2H), 6.93 (d, J = 8.80 Hz, 2H), 5.08 (s, 2H), 3.84 (s, 3H), 2.11 (s, 3H); ^{13}C NMR (101 MHz, $CDCl_3$): δ_C = 171, 159.7, 130.1, 129.4, 114, 66.1, 55.3, 21.1.

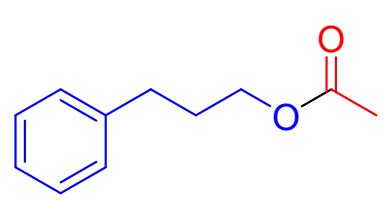

3-Phenylpropyl acetate (5g). ^{1}H NMR (400 MHz, $CDCl_3$): δ_H = 7.30 (m, 5H), 4.15 (t, J = 6.6 Hz, 2H), 2.75 (t, J = 8 Hz, 2H), 2.06 (s, 3H), 1.97 (t, J = 6.8 Hz, 2H); ^{13}C NMR (101 MHz, $CDCl_3$): δ_C = 171.1, 141.2, 128.4, 128.3, 126.0, 63.8, 32.2, 30.2, 20.9.

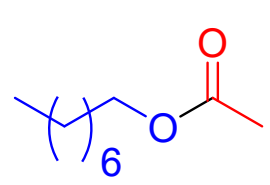

Octyl acetate (5h). ^{1}H NMR (400 MHz, $CDCl_3$): δ_H = 4.06 (t, J = 6.8 Hz, 2H), 2.05 (s, 3H), 1.6 (m, 2H), 1.3 (m, 10H), 0.8 (t, J = 7 Hz, 3H); ^{13}C NMR (101 MHz, $CDCl_3$): δ_C = 171.2, 64.6, 31.7, 29.2, 29.1, 28.6, 25.9, 22.6, 20.9, 14.0.

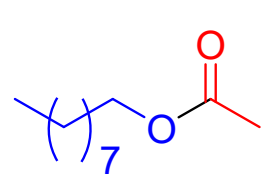

Nonyl acetate (5i). ^{1}H NMR (400 MHz, $CDCl_3$): δ_H = 4.02 (t, J = 6.5 Hz, 2H), 2.01 (s, 3H), 1.58 (m, 2H), 1.27 (m, 12H), 0.86 (t, J = 7 Hz, 3H); ^{13}C NMR (101 MHz, $CDCl_3$): δ_C = 171.0, 64.5, 31.8, 29.4, 29.2, 29.2, 28.6, 25.9, 22.6, 20.8, 14.0.

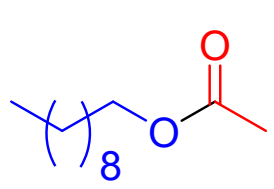

Decyl acetate (5j). ^{1}H NMR (400 MHz, $CDCl_3$): δ_H = 4.00 (t, J = 6.8 Hz, 2H), 1.98 (s, 3H), 1.57 (m, 2H), 1.27 (m, 14H), 0.84 (t, J = 7 Hz, 3H); ^{13}C NMR (101 MHz, $CDCl_3$): δ_C = 170.9, 64.5, 31.8, 29.5, 29.2, 28.5, 25.8, 22.6, 20.8, 14.0.

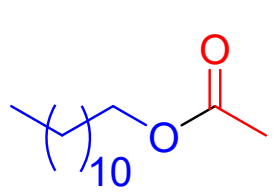

Dodecyl acetate (5k). ^{1}H NMR (400 MHz, $CDCl_3$): δ_H = 4.07 (t, J = 6.3 Hz, 2H), 2.06 (s, 3H), 1.63 (m, 2H), 1.33 (m, 18H), 0.90 (t, J = 7 Hz, 3H); ^{13}C NMR (101 MHz, $CDCl_3$): δ_C = 171.2, 64.6, 31.9, 29.6, 29.6, 29.5, 29.5, 29.3, 29.2, 28.6, 25.6, 22.6, 21.0, 14.1.

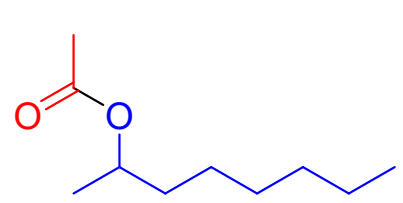

2-Octyl acetate (5l). ^{1}H NMR (400 MHz, $CDCl_3$): δ_H = 4.88 (q, J = 6.2 Hz, 1H), 2.01 (s, 3H), 1.50 (m, 2H), 1.26 (m, 11H), 0.85 (t, J = 7 Hz, 3H); ^{13}C NMR (101 MHz, $CDCl_3$): δ_C = 170.5, 70.9, 36.8, 31.7, 29.0, 25.3, 22.5, 21.2, 19.8, 13.9.

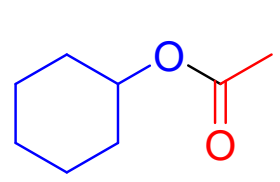

Cyclohexyl acetate (5m). ^{1}H NMR (400 MHz, $CDCl_3$): δ_H = 4.5 (m, 1H), 1.82 (s, 3H), 1.5-1.6 (m, 4H), 1.1-1.4 (m, 6H); ^{13}C NMR (101 MHz, $CDCl_3$): δ_c = 170.0, 72.2, 31.4, 25.2, 23.8, 21.0.

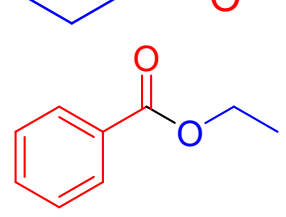

Ethyl benzoate (7a). ^{1}H NMR (400 MHz, $CDCl_3$): δ_H = 8.08 (d, J = 8.5 Hz, 2H), 7.58 (m, 1H), 7.00 (m, 2H), 4.41 (q, J = 7 Hz, 2H), 1.42 (t, J = 7 Hz, 3H); ^{13}C NMR (101 MHz, $CDCl_3$): δ_c = 166.6, 132.8, 130.5, 129.5, 129.3, 128.3, 60.9, 14.3.

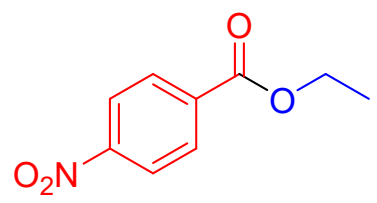

Ethyl-(4-Nitro benzoate) (7b). ^{1}H NMR (400 MHz, $CDCl_3$): δ_H = 8.19 (d, J = 7 Hz, 2H), 8.12 (d, J = 7 Hz, 2H), 4.36 (q, J = 7 Hz, 2H), 1.37 (t, J = 7 Hz, 3H); ^{13}C NMR (101 MHz, $CDCl_3$): δ_c = 164.5, 150.4, 135.8, 130.5, 123.4, 61.9, 14.1.

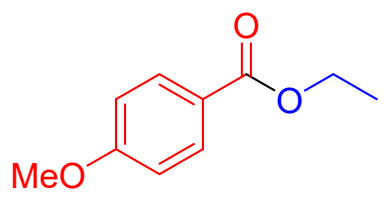

Ethyl-(3-Methoxy benzoate) (7c). ^{1}H NMR (400 MHz, $CDCl_3$): δ_H = 7.6 (m, 2H), 7.28 (s, 1H), 7.03 (m, 1H), 4.33 (q, J = 7 Hz, 2H), 3.78 (s, 3H), 1.35 (t, J = 7 Hz, 3H); ^{13}C NMR (101 MHz, $CDCl_3$): δ_c = 166.3, 159.5, 131.7, 129.3, 121.8, 119.1, 114.0, 60.9, 55.2, 14.2.

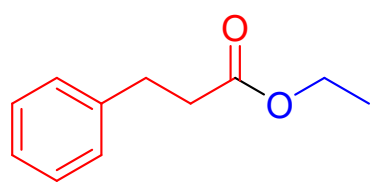

Ethyl-(3-Phenyl propionate) (7d). ^{1}H NMR (400 MHz, $CDCl_3$): δ_H = 7.3 (m, 5H), 4.17 (q, J = 7.2 Hz, 2H), 3.00 (t, J = 7.5 Hz, 2H), 2.66 (t, J = 7.5 Hz, 2H), 1.27 (t, J = 7.2 Hz, 3H); ^{13}C NMR (101 MHz, $CDCl_3$): δ_c = 172.9, 140.6, 128.5, 128.3, 126.2, 60.4, 35.9, 31.0, 14.2.

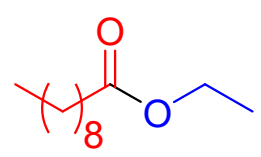

Ethyl decanoate (7e). ^{1}H NMR (400 MHz, $CDCl_3$): δ_H = 4.02 (q, J = 7.2 Hz, 2H), 2.18 (t, J = 7.5 Hz, 2H), 1.52 (m, 2H), 1.16 (m, 15H), 0.79 (t, J = 7.2 Hz, 3H); ^{13}C NMR (101 MHz, $CDCl_3$): δ_c = 173.4, 59.8, 34.1, 31.8, 29.3, 29.2, 29.1, 29.0, 24.8, 22.5, 14.0, 13.9.

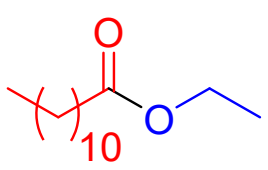

Ethyl laurate (7f). ^{1}H NMR (400 MHz, $CDCl_3$): δ_H = 4.04 (q, J = 7 Hz, 2H), 2.19 (t, J = 7.5 Hz, 2H), 1.54 (m, 2H), 1.18 (m, 19H), 0.80 (t, J = 7.5 Hz, 3H); ^{13}C NMR (101 MHz, $CDCl_3$): δ_c = 173.4, 59.8, 34.1, 31.8, 29.5, 29.4, 29.3, 29.2, 29.1, 24.9, 22.6, 14.0, 13.9.

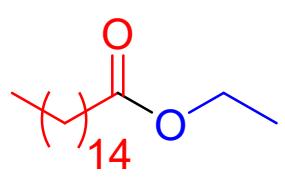

Ethyl palmitate (7g). ^{1}H NMR (400 MHz, $CDCl_3$): δ_H = 4.06 (q, J = 7 Hz, 2H), 2.22 (t, J = 7.8 Hz, 2H), 1.55 (m, 2H), 1.19 (m, 27H), 0.82 (t, J = 7 Hz, 3H); ^{13}C NMR (101 MHz, $CDCl_3$): δ_c = 173.5, 59.9, 34.2, 31.9, 29.6, 29.6, 29.3, 29.5, 29.4, 29.3, 29.2, 29.1, 24.9, 22.6, 14.1, 14.0.

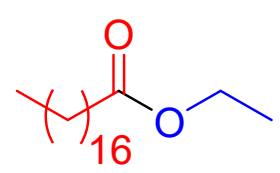

Ethyl stearate (7h). ^{1}H NMR (400 MHz, $CDCl_3$): δ_H = 4.15 (q, J = 7 Hz, 2H), 2.32 (t, J = 7.8 Hz, 2H), 1.64, (m, 2H), 1.29 (m, 32H), 0.91 (t, J = 7.8 Hz, 3H); ^{13}C NMR (101 MHz, $CDCl_3$): δ_c = 173.9, 60.1, 34.4, 31.9, 29.7, 29.4, 29.1, 25.0, 22.7, 14.1.

NMR and IR data of Biginelli products:

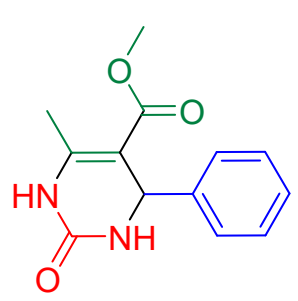

Methyl 6-methyl-2-oxo-4-phenyl-1,2,3,4-tetrahydropyrimidine-5-carboxylate (9a). ^{1}H NMR (400 MHz, DMSO): δ_H = 9.24 (s, 1H), 7.78 (s, 1H), 7.25-7.37 (m, 5H), 5.17 (d, J = 3.3 Hz, 1H), 3.55 (s, 3H), 2.28 (s, 3H); ^{13}C NMR (101 MHz, DMSO): δ_C = 166.3, 152.6, 149.2, 145.2, 129.0, 127.8, 126.7, 99.5, 54.3, 51.8, 18.3.

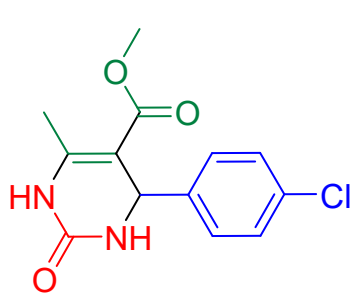

Methyl 4-(4-chlorophenyl)-6-methyl-2-oxo-1,2,3,4-tetrahydropyrimidine-5-carboxylate (9b). ^{1}H NMR (400 MHz, DMSO): δ_H = 9.31 (s, 1H), 7.83 (s, 1H), 7.39-7.42 (m, 2H), 7.26-7.28 (m, 2H), 5.16 (d, J = 3.4 Hz, 1H), 3.55 (s, 3H), 2.27 (s, 3H). ^{13}C NMR (101 MHz, DMSO): δ_C = 166.2, 152.5, 149.5, 144.1, 132.3, 128.9, 128.6, 99.1, 53.7, 51.3, 18.3.

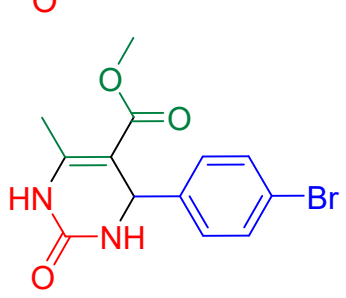

Methyl 4-(4-boromophenyl)-6-methyl-2-oxo-1,2,3,4-tetrahydropyrimidine-5-carboxylate (9c). ^{1}H NMR (400 MHz, DMSO): δ_H = 9.30 (s, 1H), 7.82 (s, 1H), 7.56-7.53 (m, 2H), 7.21-7.19 (m, 2H), 5.15 (d, J = 3.2 Hz, 1H), 3.56 (s, 3H), 2.27 (s, 3H). ^{13}C NMR (101 MHz, DMSO): δ_C = 166.2, 152.5, 149.5, 144.5, 131.8, 128.9, 120.8, 99.1, 53.8, 51.3, 18.3.

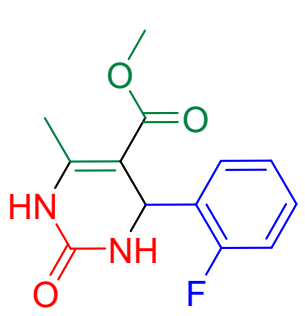

Methyl 4-(2-fluorophenyl)-6-methyl-2-oxo-1,2,3,4 tetrahydropyrimidine-5-carboxylate (9d). ^{1}H NMR (400 MHz, DMSO): δ_H = 9.33 (s, 1H), 7.76 (s, 1H), 7.30-7.34 (m, 2H), 7.25-7.30 (m, 2H), 5.47 (d, J = 3.1 Hz, 1H), 3.49 (s, 3H), 2.30 (s, 3H). ^{13}C NMR (101 MHz, DMSO): δ_C = 166.0, 161.1, 158.7, 152.1, 149.7, 131.9, 129.5, 125.0, 116.0, 97.7, 51.2, 49.1, 18.3.

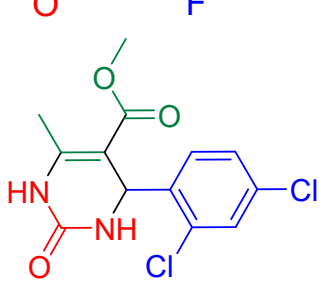

Methyl 4-(2,4-dicholorophenyl)-6-methyl-2-oxo-1,2,3,4-tetrahydropyrimidine-5-carboxylate (9e). ^{1}H NMR (400 MHz, DMSO): δ_H = 9.37 (s, 1H), 7.78 (s, 1H), 7.58 (d, J = 2.1 Hz, 1H), 7.43 (dd, J = 2.0, 2.0 Hz, 1H), 7.33 (d, J = 8.4 Hz, 1H), 5.60 (d, J = 2.9 Hz, 1H), 3.48 (s, 3H), 2.31 (s, 3H); ^{13}C NMR (101 MHz, DMSO): δ_C = 165.9, 156.3, 152.9, 150.1, 142.6, 110.8, 105.7, 97.0, 51.3, 49.1, 18.3.

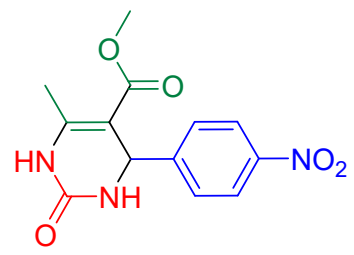

Methyl 4-(4-nitrophenyl)-6-methyl-2-oxo-1,2,3,4-tetrahydropyrimidine-5-carboxylate (9f). ^{1}H NMR (400 MHz, DMSO): δ_H = 9.07 (s, 1H), 7.54 (s, 1H), 7.30-7.26 (m, 2H), 7.20-7.15 (m, 2H), 4.13-4.09 (m, 1H), 3.58 (s, 3H), 2.19 (s, 3H); ^{13}C NMR (101 MHz, DMSO): δ_C = 166.3, 153.3, 149.1, 142.1, 128.7, 126.1, 99.5, 51.1, 50.4, 30.5, 18.2.

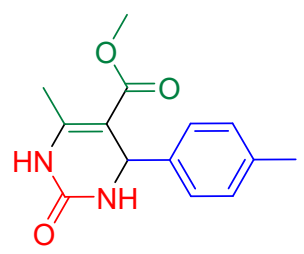

Methyl 6-methyl-2-oxo-4-p-tolyl-1,2,3,4-tetrahydropyrimidine-5-carboxylate (9g). ^{1}H NMR (400 MHz, DMSO) δ_H = 9.22 (d, *J* = 1.5 Hz, 1H), 7.74 (dd, *J* = 3.1, 2.1 Hz, 1H), 7.13 (s, 4H), 5.12 (d, *J* = 3.3 Hz, 1H), 3.54 (s, 3H), 2.27 (d, *J* = 4.8 Hz, 6H); ^{13}C NMR (101 MHz, DMSO): δ_C = 166.3, 152.7, 149.0, 142.2, 136.9, 129.4, 126.6, 99.6, 53.9, 51.2, 21.1, 18.2.

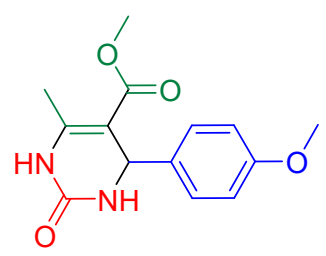

Methyl 4-(4-methoxyphenyl)-6-methyl-2-oxo-1,2,3,4-tetrahydropyrimidine-5-carboxylate (9h). ^{1}H NMR (400 MHz, DMSO): δ_H = 9.21 (s, 1H), 7.71 (s, 1H), 7.17-7.15 (m, 2H), 6.92-6.86 (m, 2H), 5.10 (d, *J* = 3.3 Hz, 1H), 3.74 (s, 3H), 3.54 (s, 3H), 2.53 (s, 3H); ^{13}C NMR (101 MHz, DMSO): δ_C = 166.3, 158.9, 152.7, 148.8, 137.3, 127.8, 114.2, 99.7, 55.5, 53.6, 51.3, 18.3.

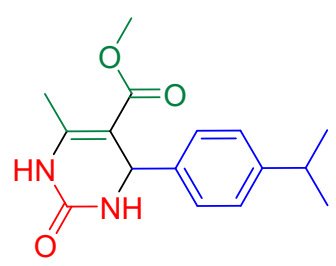

Methyl 4-(4-isopropylphenyl)-6-methyl-2-oxo-1,2,3,4-tetrahydropyrimidine-5-carboxylate (9i). ^{1}H NMR (400 MHz, DMSO): δ_H = 9.23 (s, 1H), 7.74 (s, 1H), 7.23-7.15 (m, 4H), 5.13 (d, *J* = 3.3 Hz, 1H), 3.55 (s, 3H), 2.85 (dq, *J* = 13.7, 6.9 Hz, 1H), 2.27 (s, 3H), 1.19 (d, *J* = 6.9 Hz, 6H); ^{13}C NMR (101 MHz, DMSO): $\delta_{C =}$ 166.3, 152.7, 149.0, 147.9, 142.6, 126.8, 126.6, 99.6, 53.9, 51.3, 33.6, 24.3, 18.

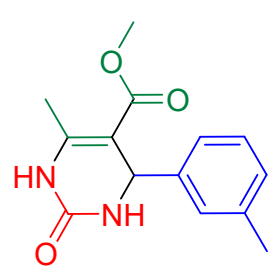

Methyl 6-methyl-2-oxo-4-m-tolyl-1,2,3,4-tetrahydropyrimidine-5-carboxylate (9j). ^{1}H NMR (400 MHz, DMSO): δ_H = 9.21 (s, 1H), 7.73 (t, *J* = 14.8 Hz, 1H), 7.21 (d, *J* = 8.0 Hz, 1H), 7.00-7.02(d, *J* = 8.0 Hz, 3H), 5.12-5.11(d, *J* = 3.8 Hz, 1H), 3.54 (s, 3H), 2.30 (s, 3H), 2.29 (s, 3H); ^{13}C NMR (101 MHz, DMSO) δ_C = 166.3, 152.7, 149.1, 145.1, 137.9, 128.8, 128.5, 127.1, 123.7, 99.5, 54.3, 51.3, 21.6, 18.3.

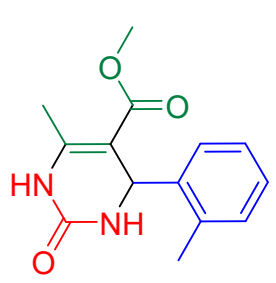

Methyl 6-methyl-2-oxo-4-o-tolyl-1,2,3,4-tetrahydropyrimidine-5-carboxylate (9k). ^{1}H NMR (400 MHz, DMSO): δ_H = 9.22 (s, 1H), 7.67 (s, 1H), 7.21-7.12 (m, 4H), 5.41 (d, *J* = 2.8 Hz, 1H), 3.47 (s, 3H), 2.43 (s, 3H), 2.31 (s, 3H); ^{13}C NMR (101 MHz, DMSO): δ_C = 166.2, 152.1, 149.1, 143.7, 135.1, 130.7, 127.7, 127.0, 127.0, 99.5, 51.2, 50.8, 19.1, 18.2.

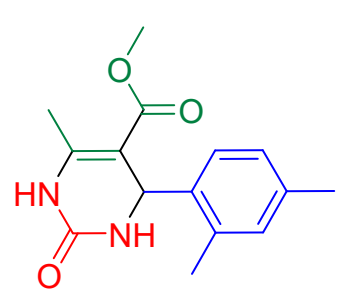

Methyl 4-(2,4-dimethylphenyl)-6-methyl-2-oxo-1,2,3,4-tetrahydropyrimidine-5-carboxylate (9l). ^{1}H NMR (400 MHz, DMSO): δ_H = 9.18 (s, 1H), 7.61 (s, 1H), 7.07 (d, *J* = 7.6 Hz, 1H), 6.95 (t, *J* = 8 Hz, 2H), 5.36 (d, *J* = 2.8 Hz, 1H), 3.49-3.43 (m, 3H), 2.37 (m, 3H), 2.30 (m, 3H), 2.23 (m, 3H); ^{13}C NMR (101 MHz, DMSO): δ_C = 166.2, 152.1, 148.9, 140.8, 136.7, 134.9, 131.3, 127.6, 126.9, 99.6, 51.2, 50.5, 21.1, 19.1, 18.2.

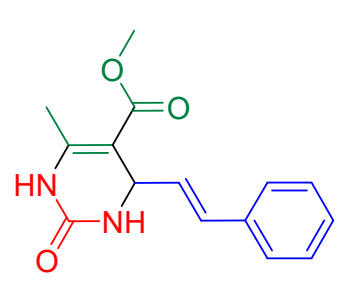

Methyl 6-methyl-2-oxo-4-styryl-1,2,3,4-tetrahydropyrimidine-5-carboxylate (9m). ^{1}H NMR (400 MHz, DMSO): δ_H = 9.19 (s, 1H), 7.58 (s, 1H), 7.42 (t, *J* = 2 Hz, 2H), 7.32 (t, *J* = 7.5 Hz, 2H), 7.26-7.23 (m, 1H), 6.38 (d, *J* = 16.0 Hz, 1H), 6.25 (dd, *J* = 5.6 Hz, *J* = 5.6 Hz, 1H), 4.74 (d, *J* = 8.0 Hz, 1H), 3.65 (s, 3H), 2.22 (s, 3H); ^{13}C NMR (101 MHz, DMSO): δ_C = 166.2, 153.2, 149.2, 136.7, 130.5, 129.1, 128.5, 128.1, 126.8, 98.2, 52.2, 51.4, 18.3.

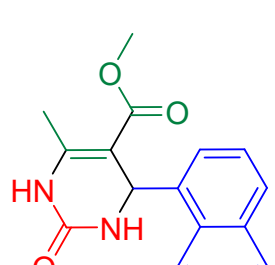

Methyl 6-methyl-4-(naphthalene-1-yl)-2-oxo-1,2,3,4-tetrahydropyrimidine-5-carboxylate (9n). ^{1}H NMR (400 MHz, DMSO): δ_H = 9.32 (s, 1H), 8.33 (d, *J* = 8.4 Hz, 1H), 7.97 (d, *J* = 8.2, Hz, 1H), 7.87 (d, *J* = 8.1 Hz, 1H), 7.79 (s, 1H), 7.64-7.54 (m, 2H), 7.53 (t, *J* = 7.0 Hz, 1H), 7.41 (d, *J* = 7.2, 1H), 6.07 (d, *J* = 3.4 Hz, 1H), 3.39 (s, 3H), 2.40 (s, 3H); ^{13}C NMR (101 MHz, DMSO): δ_C = 166.3, 152.3, 149.7, 140.3, 134.1, 130.5, 128.9, 128.5, 126.6, 126.2, 126.1, 124.4, 124.1, 99.2, 51.2, 50.1, 18.4.

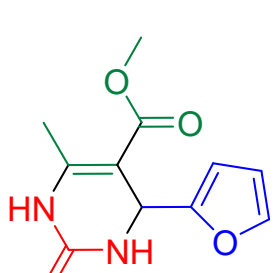

Methyl 4-(4-furan-2-yl)-6-methyl-2-oxo-1,2,3,4-tetrahydropyrimidine-5-carboxylate (9o). ^{1}H NMR (400 MHz, DMSO): δ_H = 9.30 (s, 1H), 7.81 (s, 1H), 7.577-7.571 (m, 1H), 6.37-6.36 (m, 1H), 6.12 – 6.11 (m, 1H), 5.22 (d, *J* = 3.5 Hz, 1H), 3.59 (s, 3H), 2.26 (s, 3H); ^{13}C NMR (101 MHz, DMSO): δ_C = 165.9, 156.3, 152.9, 150.2, 142.7, 110.8, 105.8, 97.0, 51.4, 48.1, 18.3.

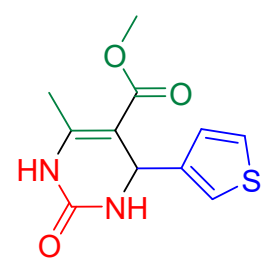

Methyl 4-(4-thiol-3-yl)-6-methyl-2-oxo-1,2,3,4-tetrahydropyrimidine-5-carboxylate (9p). ^{1}H NMR (400 MHz, DMSO): δ_H = 9.24 (s, 1H), 7.79 (s, 1H), 7.49-7.47 (m, 1H), 7.17-7.16 (m, 1H), 7.02-7.00 (m, 1H), 5.23 (d, *J* = 3.5 Hz, 1H), 3.61 (s, 3H), 2.25 (s, 3H); ^{13}C NMR (101 MHz, DMSO): δ_C = 166.8, 153.1, 149.2, 146.1, 127.2, 126.6, 121.2, 99.7, 51.3, 49.8, 18.3.

Table S1. Esterification of benzyl alcohol with acetic acid in the presence of different sulfonic acid catalysts

Entry	Catalyst (mol%)	Acid capacity[a]	Molar ratio alcohol:acid	T (°C)	Time (h)	Yield (%)	TOF (h^{-1})[d]	Ref.
1[b]	Et-PMO-Me-PrSO_3H (1)	0.5	1:5	60	24	99	4.12	1
2[b]	SBA-15 PrSO_3H (3)	1.2	1:3	60	24	72	1.00	-
3[b]	H_2SO_4 (3)	-	1:3	60	24	70	0.97	-
4[c]	SO_3H-ethane PMO-I (100 mg, 0.22)	1.2	1:1	120	6	80	60.60	2
5	PMO-IL-PrSO_3H (5)	0.22	1:1	70	18	95	1.05	3
6[d]	GPh- SO_3H (18)	0.9	10:1	70	6	90	0.08	4
**7**	**1b (0.5)**	**0.1**	**1:2**	**50**	**16**	**97**	**12.25**	**This work**

[a]Determined by titration after ion-exchange (mmol $H^+.g^{-1}$); [b]*Catal. Sci. Technol.* **2015**, *5*, 3624-3631, [c]The reaction were prformed under N_2 atmosphere, *J. Catal.* **2015**, 326, 139-148; [d]Sulfonic acid functionalized mesoporous benzene–silica with crystal-like pore wall structure. The reaction was performed using acetic acid and excess of EtOH. TOF = yield (%)/(Cat. (mol%) × Time (h)).

Table S2. Optimizing the reaction parameters for the esterification of benzyl alcohol with acetic acid

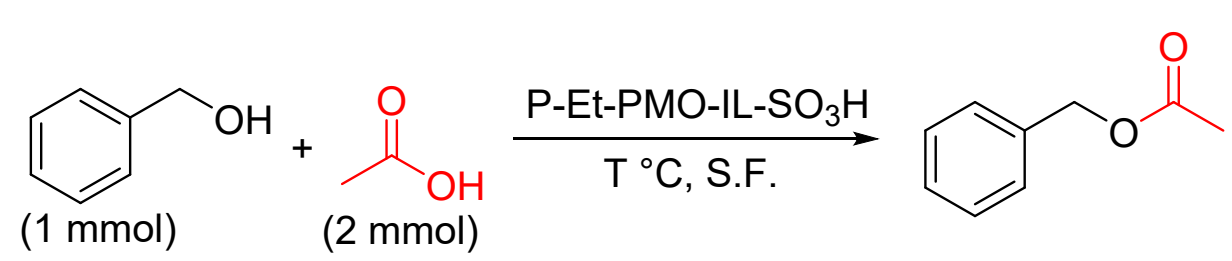

Entry	Catalyst (mol %)	T (°C)	Time (h)	GC Yield (%)
1	0.4	50	16	87
2	0.5	r.t.	16	64
3	0.5	r.t.	24	80
4	0.5	r.t.	48	85
5	1	r.t.	48	87
6	0.5	40	24	90
7	1	40	48	91
**8**	**0.5**	**50**	**16**	**97**
9	0.5	50	14	90
10[a]	0.5	50	24	80
11[a]	0.5	80	16	87

[a] benzyl alcohol: acetic acid (1:1)

Table S3. CHNS results

Material	C%	N%	H%	IL Loading (mmol/g)[a]
**1b**	14.09	2.494	3.324	0.891
**1a**	15.63	2.556	2.972	0.913
**1c**	19.15	2.681	3.515	0.957
**1d**	19.98	2.400	3.248	0.857

[a] Calculated according to the CHNS results

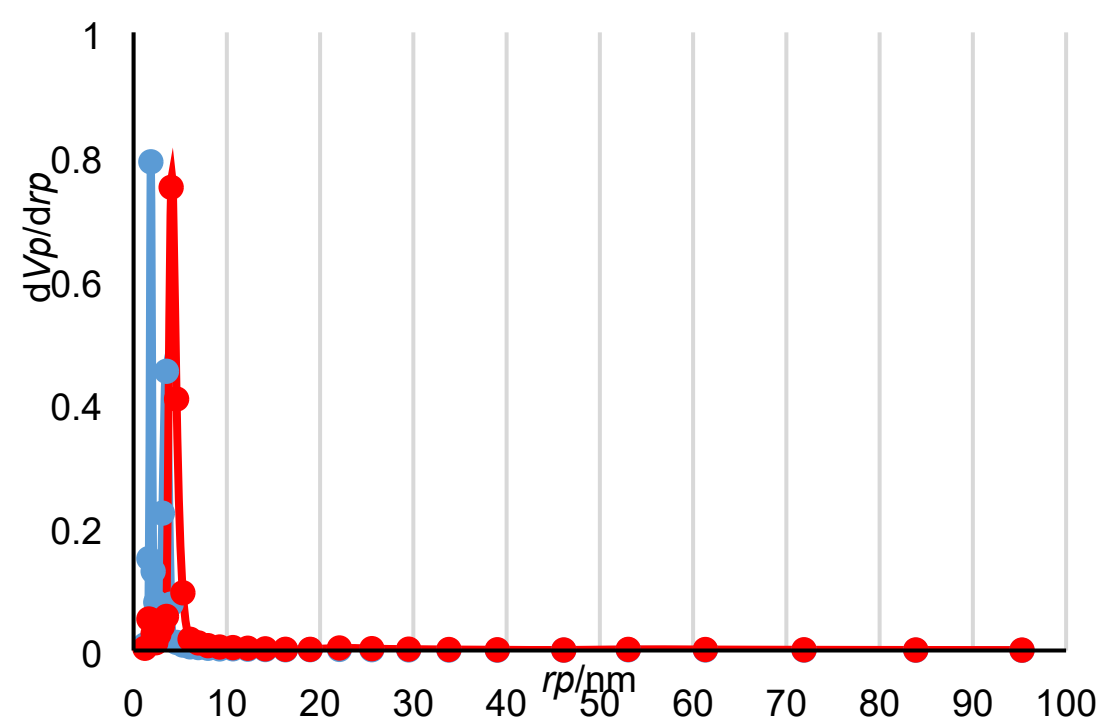

Figure S1. Dollimore-Heal (DH) pore size distribution curves for **1a** (red) and **1b** (blue).

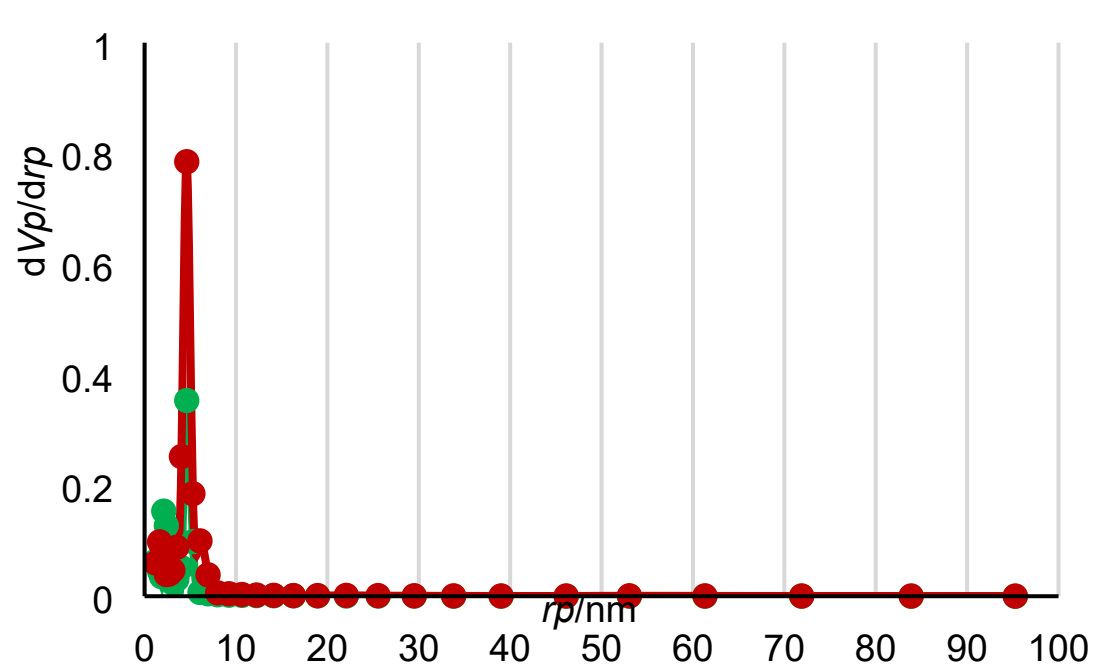

Figure S2. Dollimore-Heal (DH) pore size distribution curves for **1c** (red) and **1d** (green).P-Ph-PMO-IL-SO_3H and U-Ph-PMO-IL-SO_3H

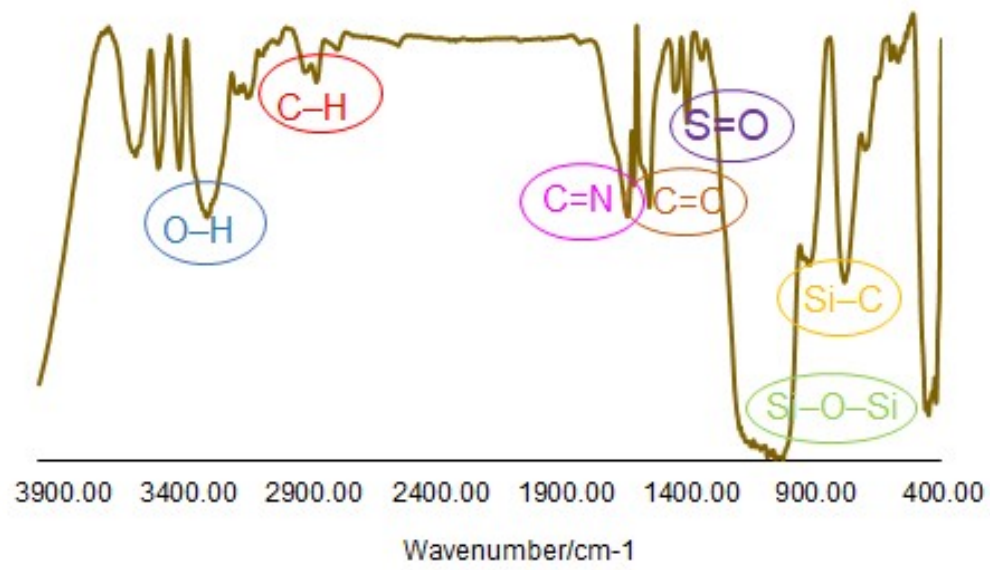

Figure S3. FTIR spectrum of the **1b** catalyst

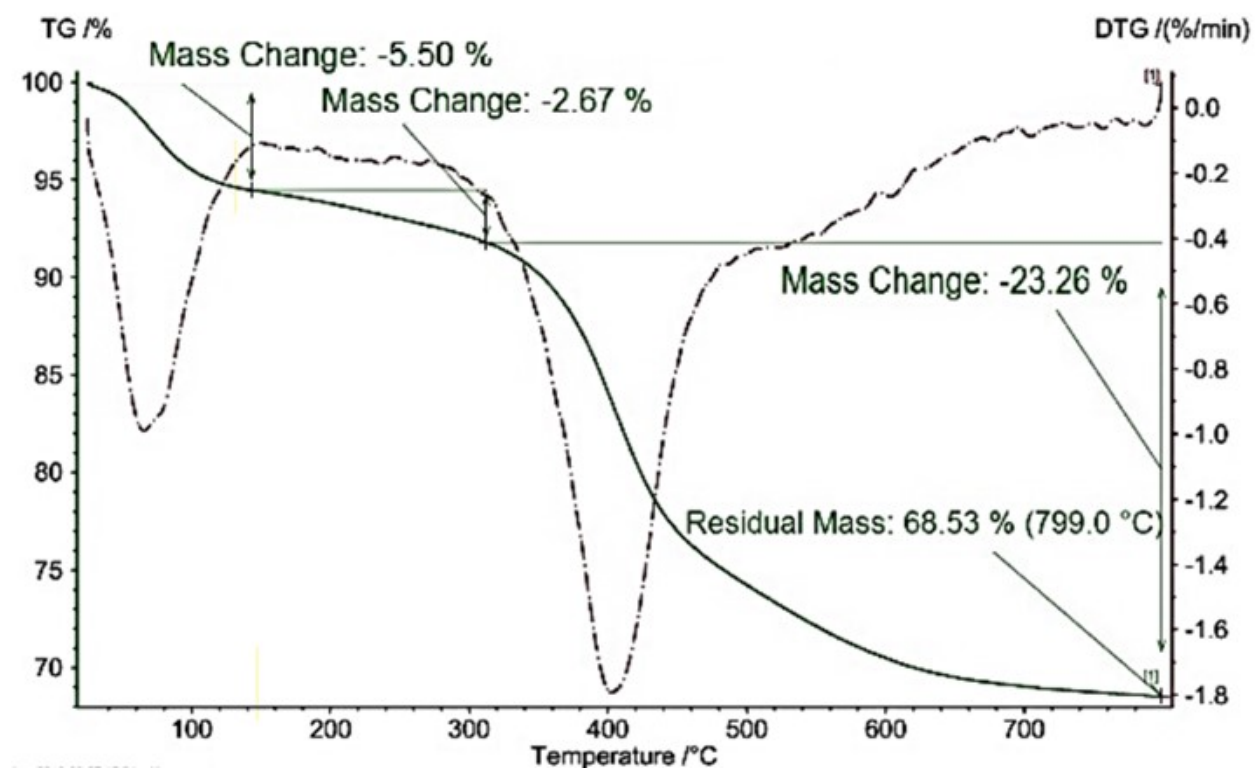

Figure S4. TGA curve of the **1b** catalyst under an oxygen atmosphere

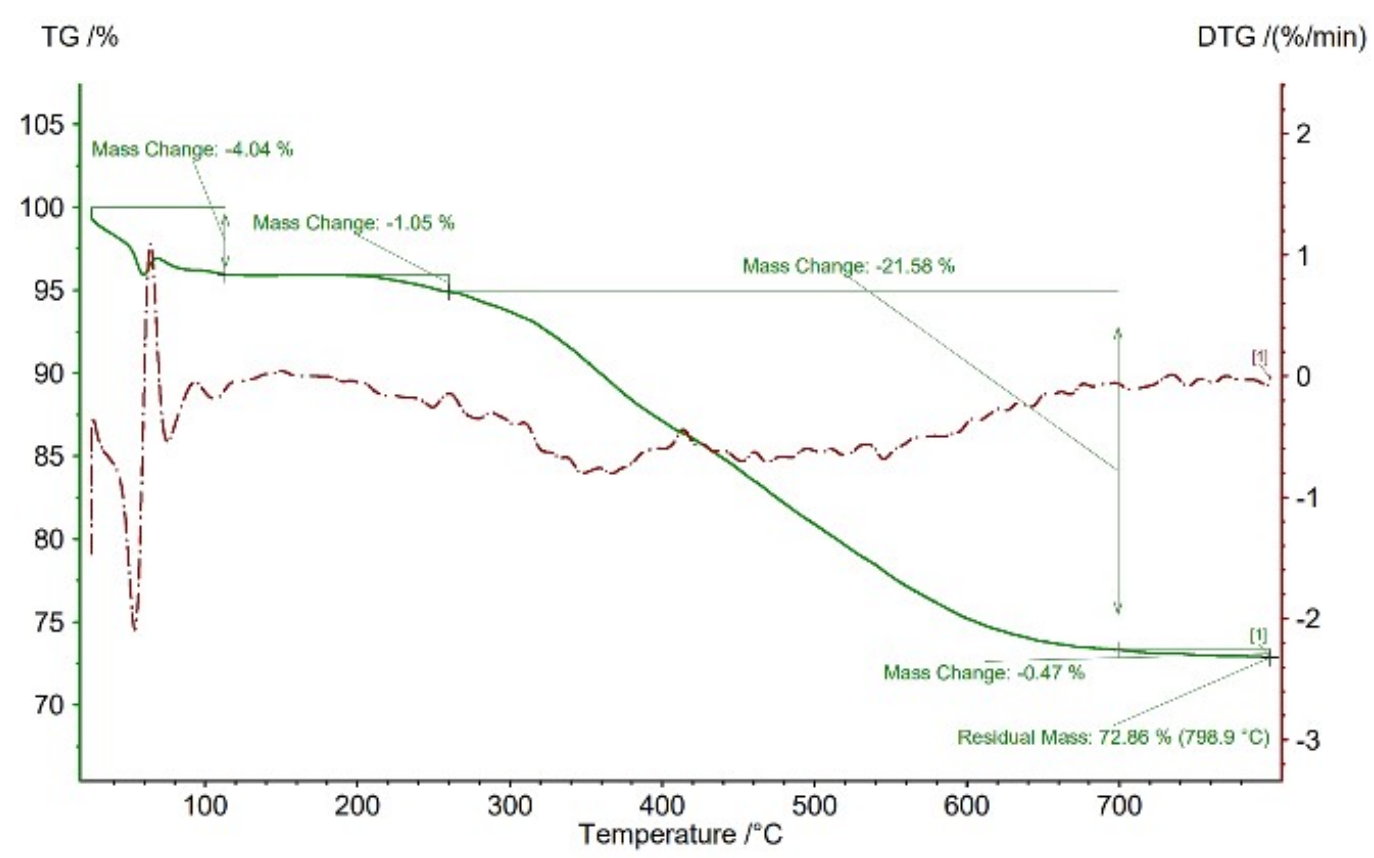

Figure S5. TGA curve of the **1b** catalyst under a nitrogen atmosphere

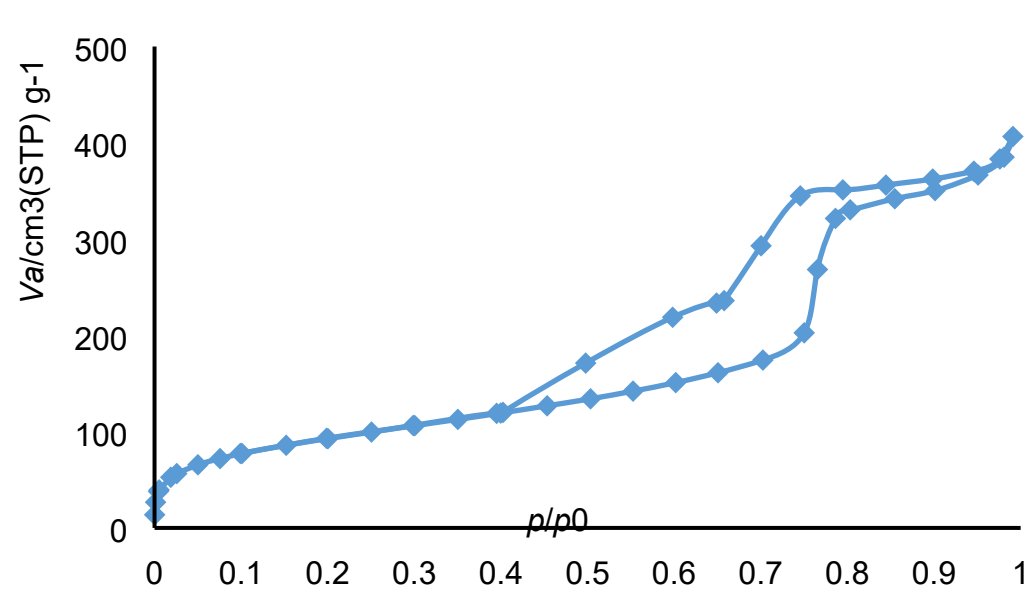

Figure S6. N_2 adsorption-desorption curve of the recovered **1b** catalyst

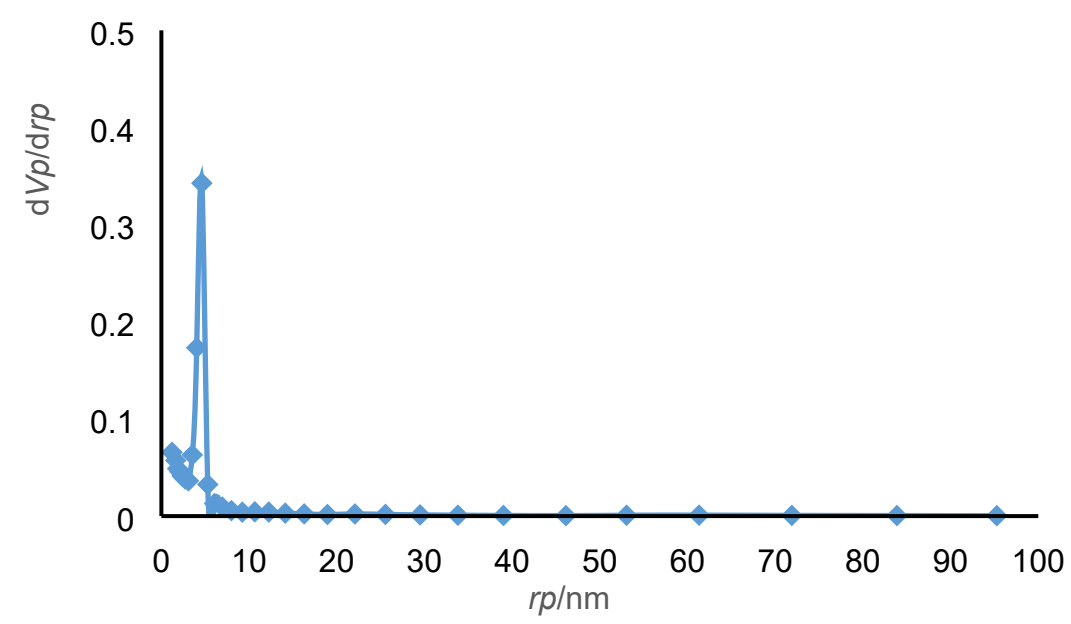

Figure S7. BJH pore size distribution curve of the recovered **1b** catalyst

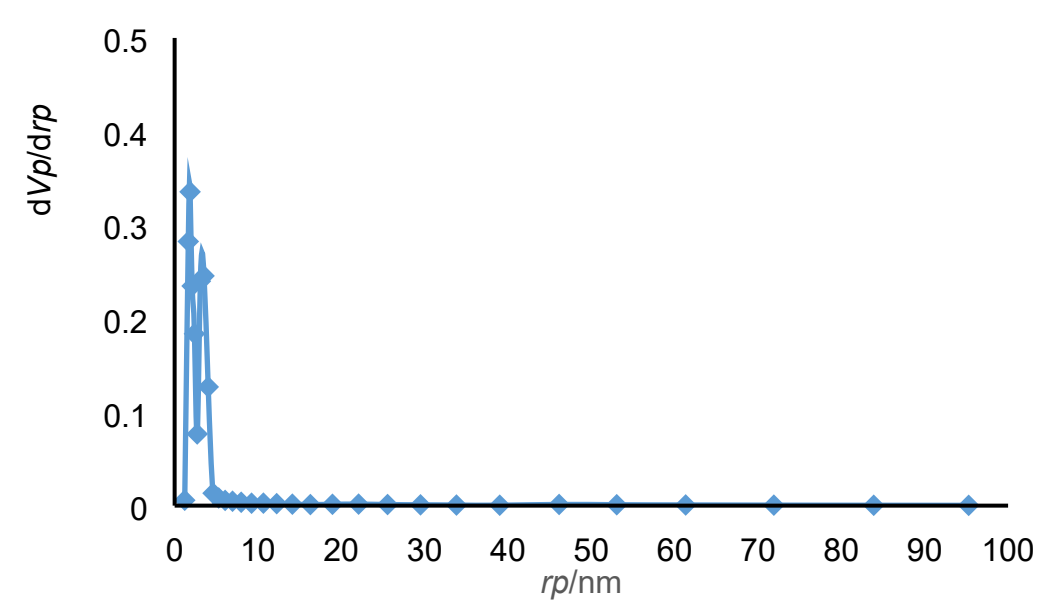

Figure S8. DH pore size distribution curve of the recovered **1b** catalyst

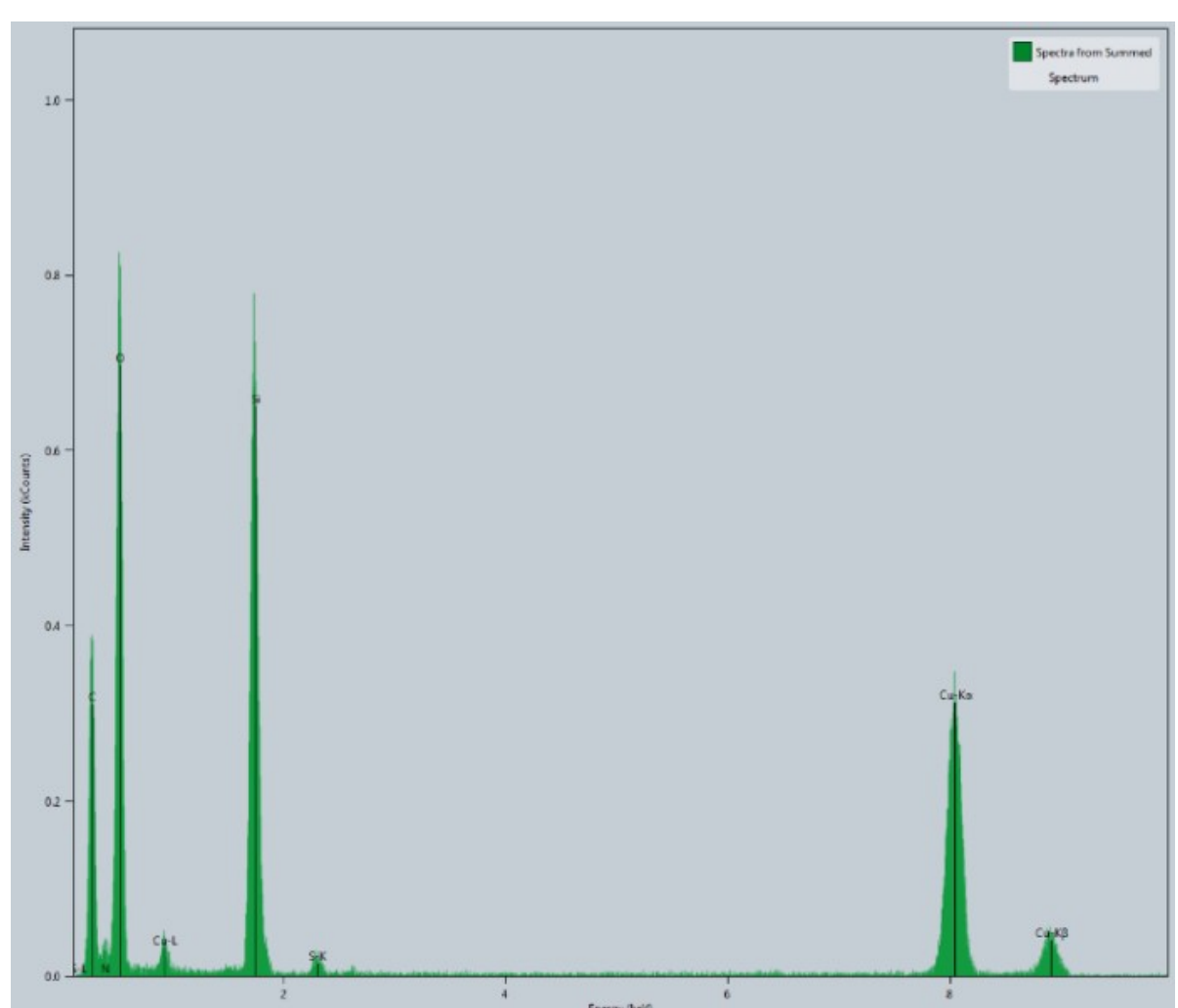

Figure S9. EDS spectrum of **1b** catalyst

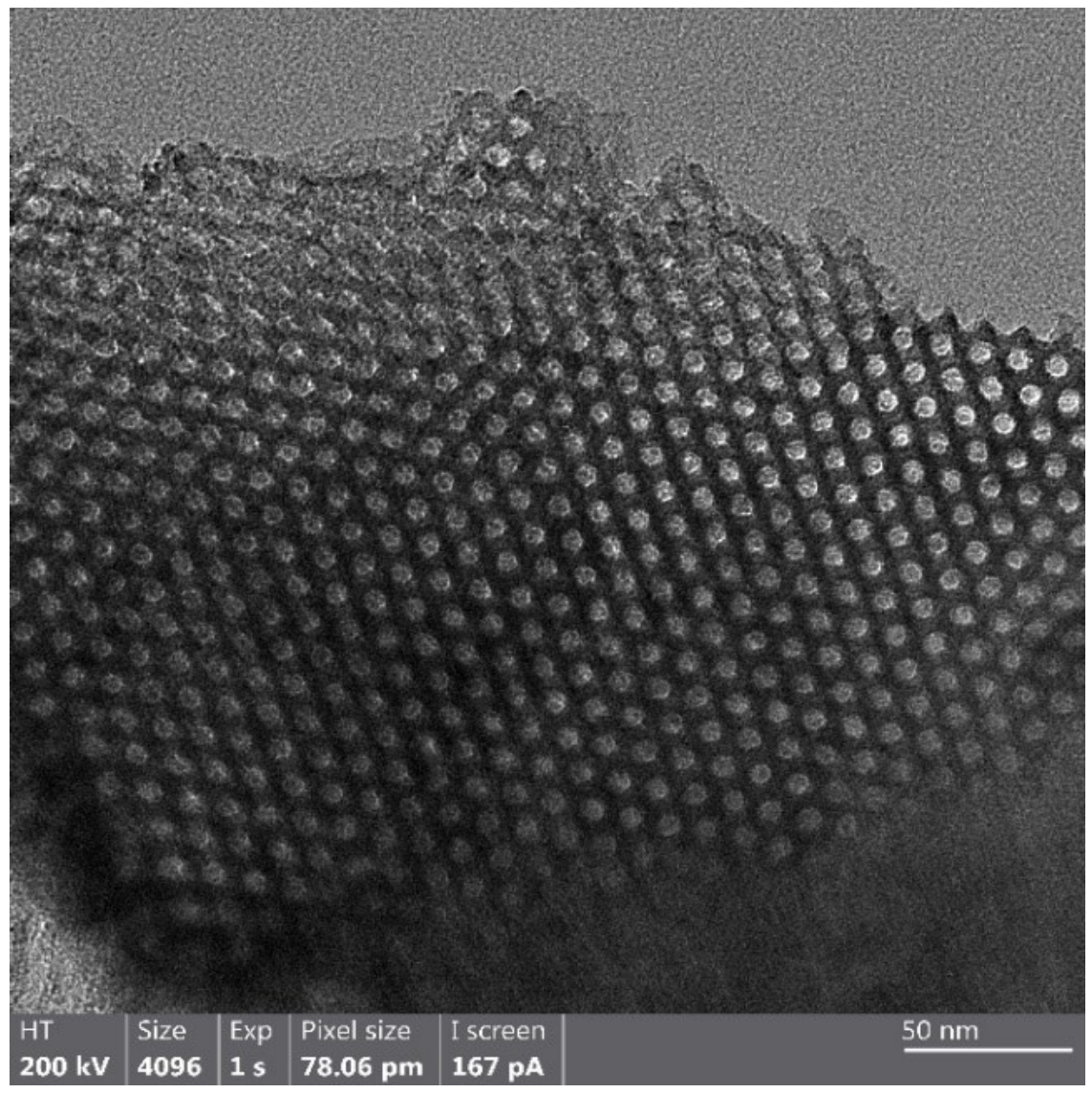

Figure S10. TEM image of the **1b** catalyst, perpendicular to the mesochannels (scale bar: 50 nm)

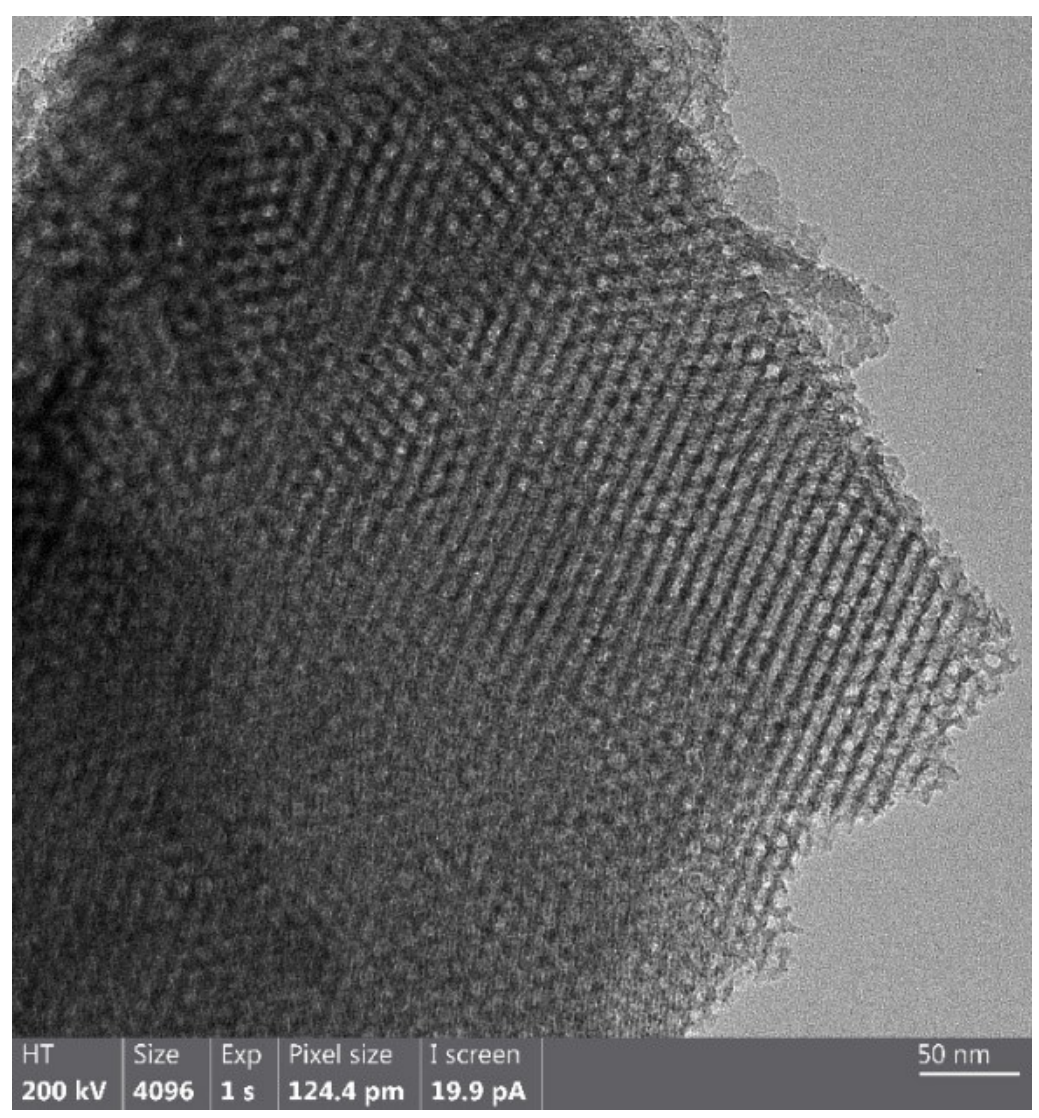

Figure S11. TEM image of the **1b** catalyst, along to the mesochannels (scale bar: 50 nm)

NMR Spectra of Ester Products

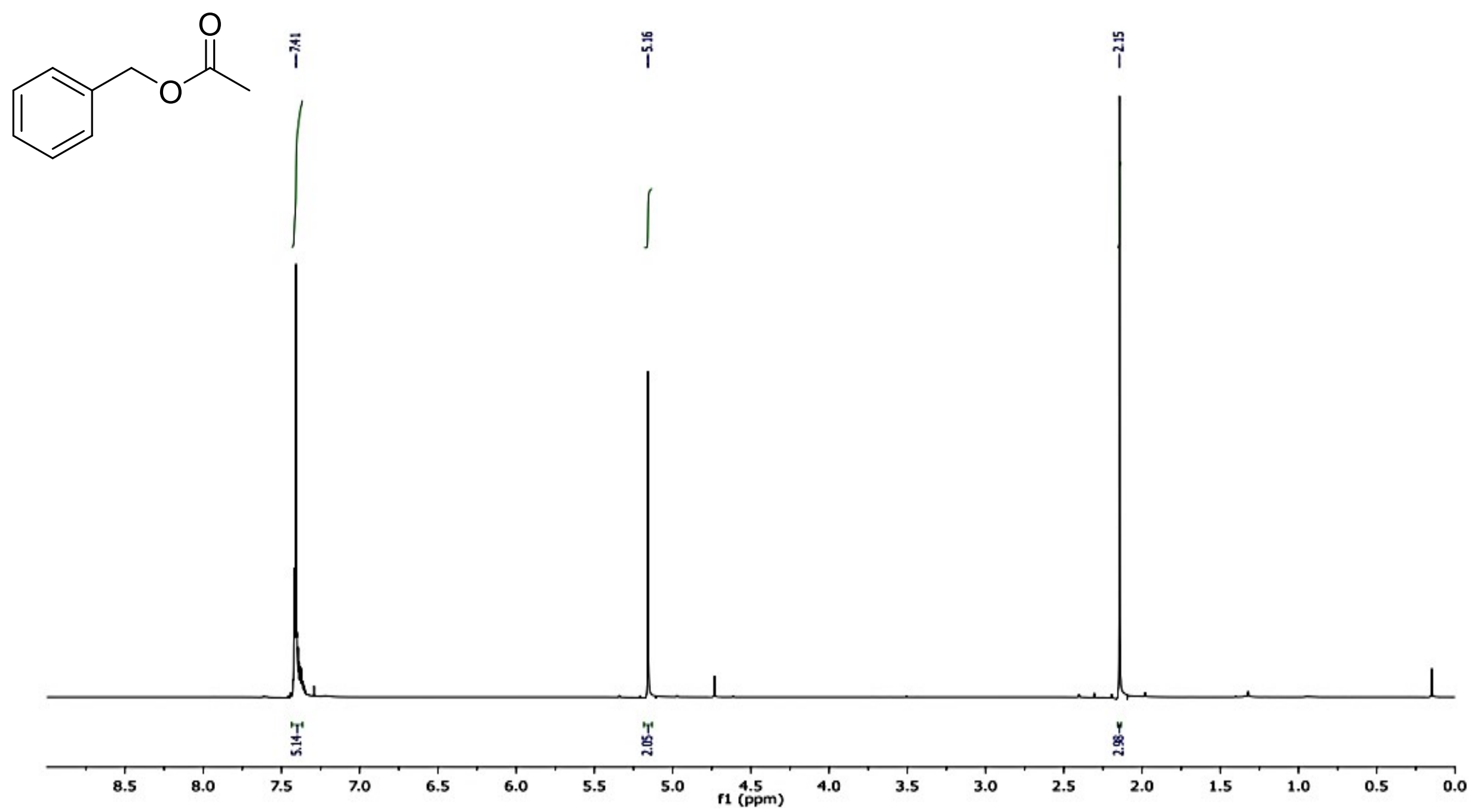

Figure S12. ^{1}H NMR spectrum of the ethyl benzoate (**5a**) product in $CDCl_3$

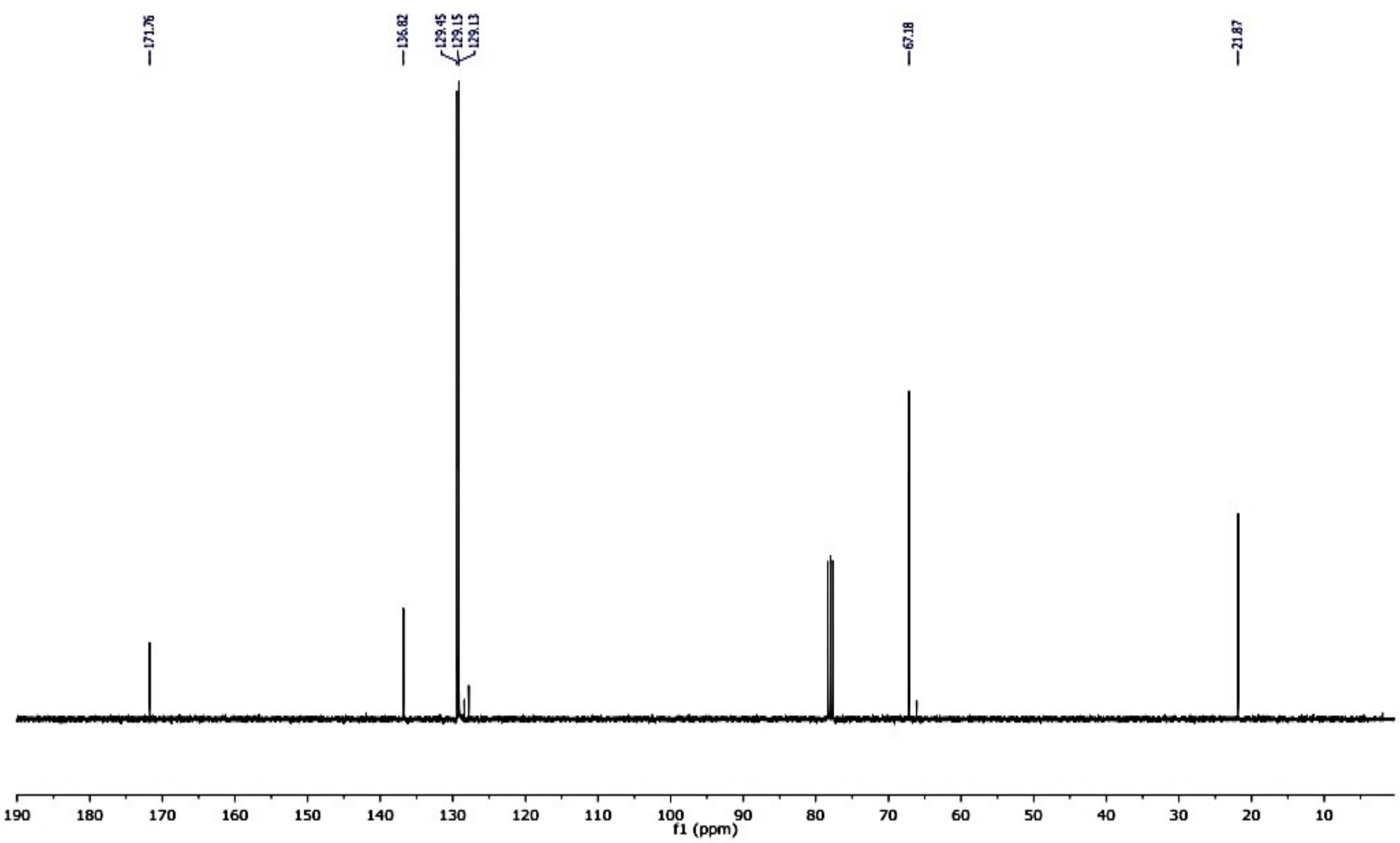

Figure S13. ^{13}C NMR spectrum of the ethyl benzoate (**5a**) product in $CDCl_3$

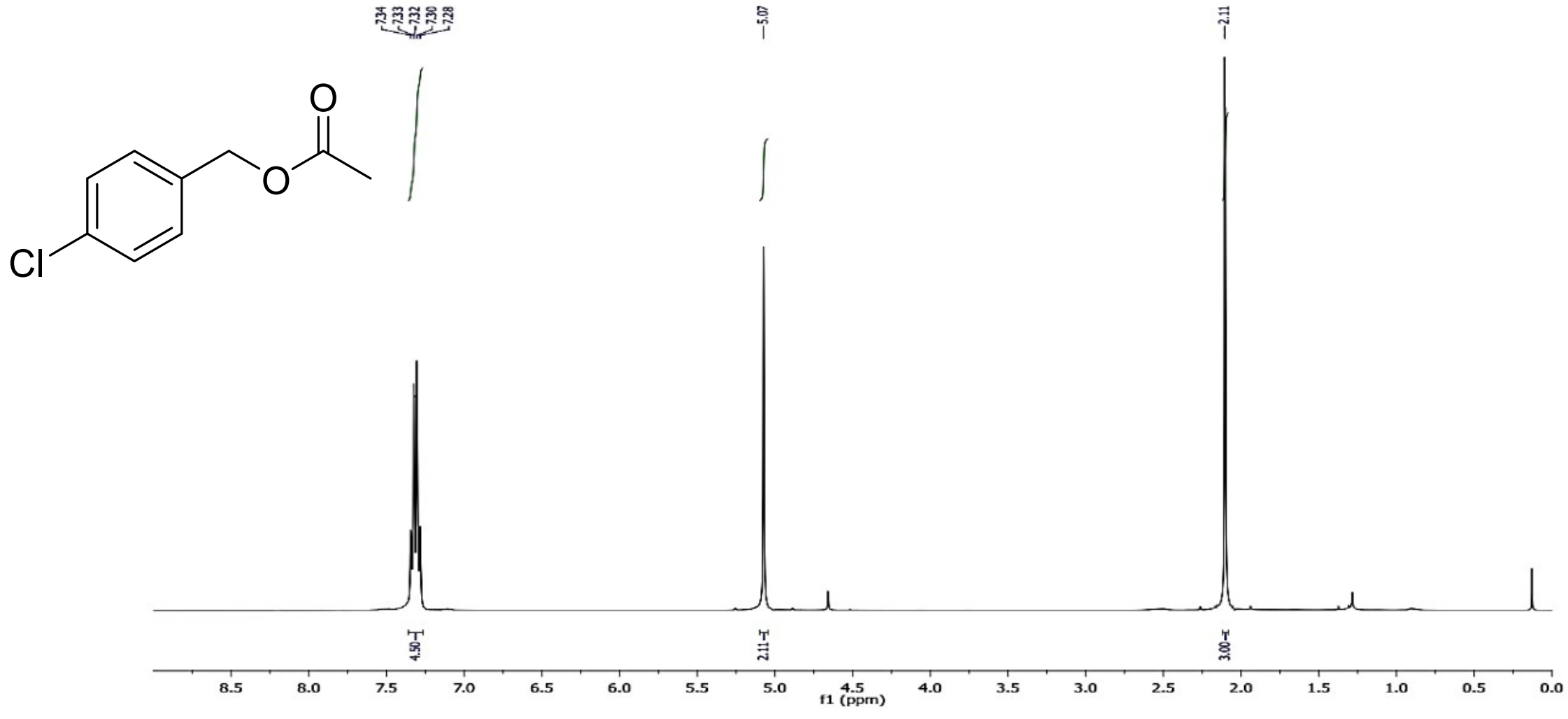

Figure S14. ^{1}H NMR spectrum of the 4-chlorobenzyl acetate (**5b**) product in $CDCl_3$

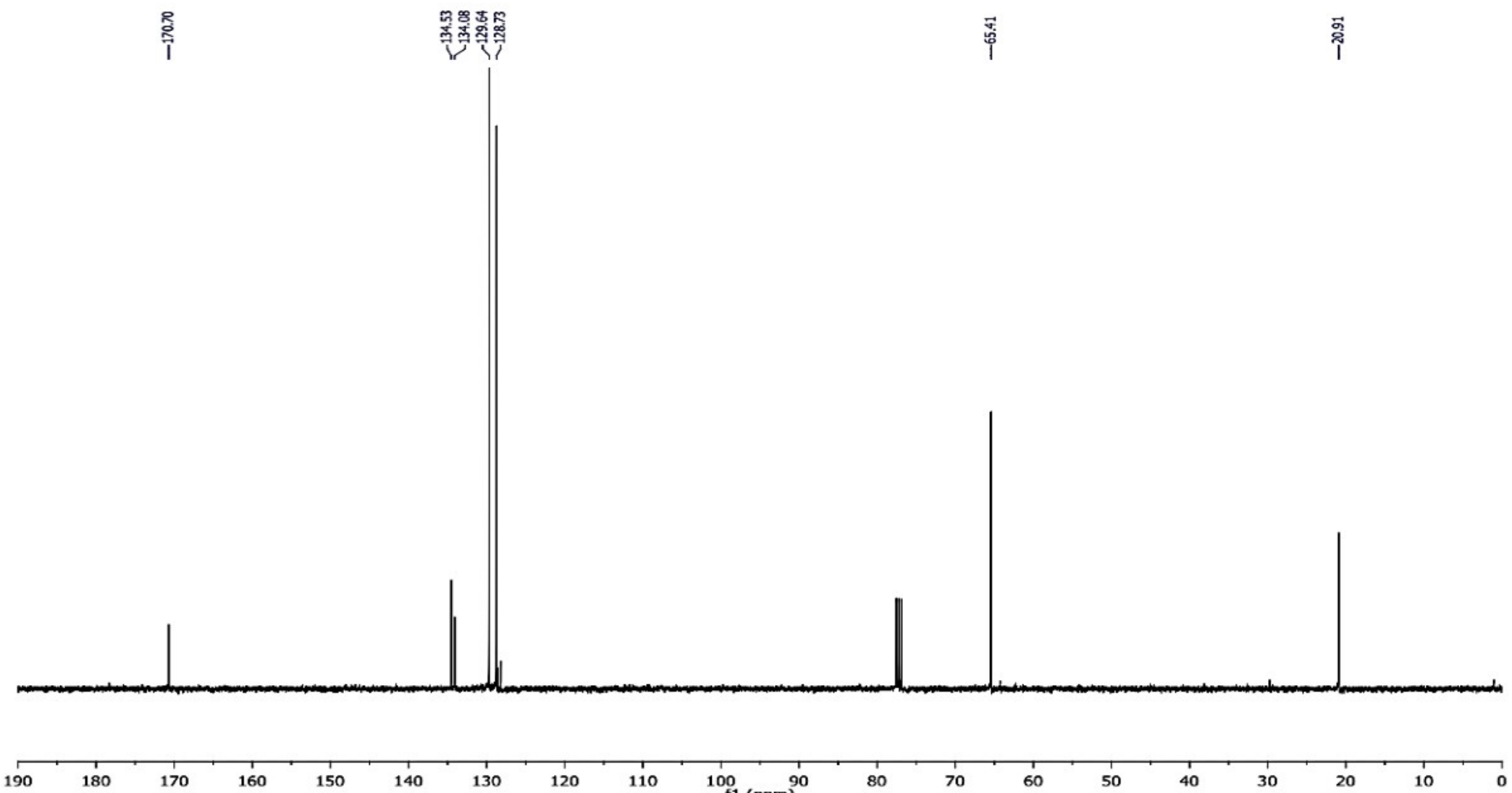

Figure 15. ^{13}C NMR spectrum of the 4-chlorobenzyl acetate (**5b**) product in $CDCl_3$

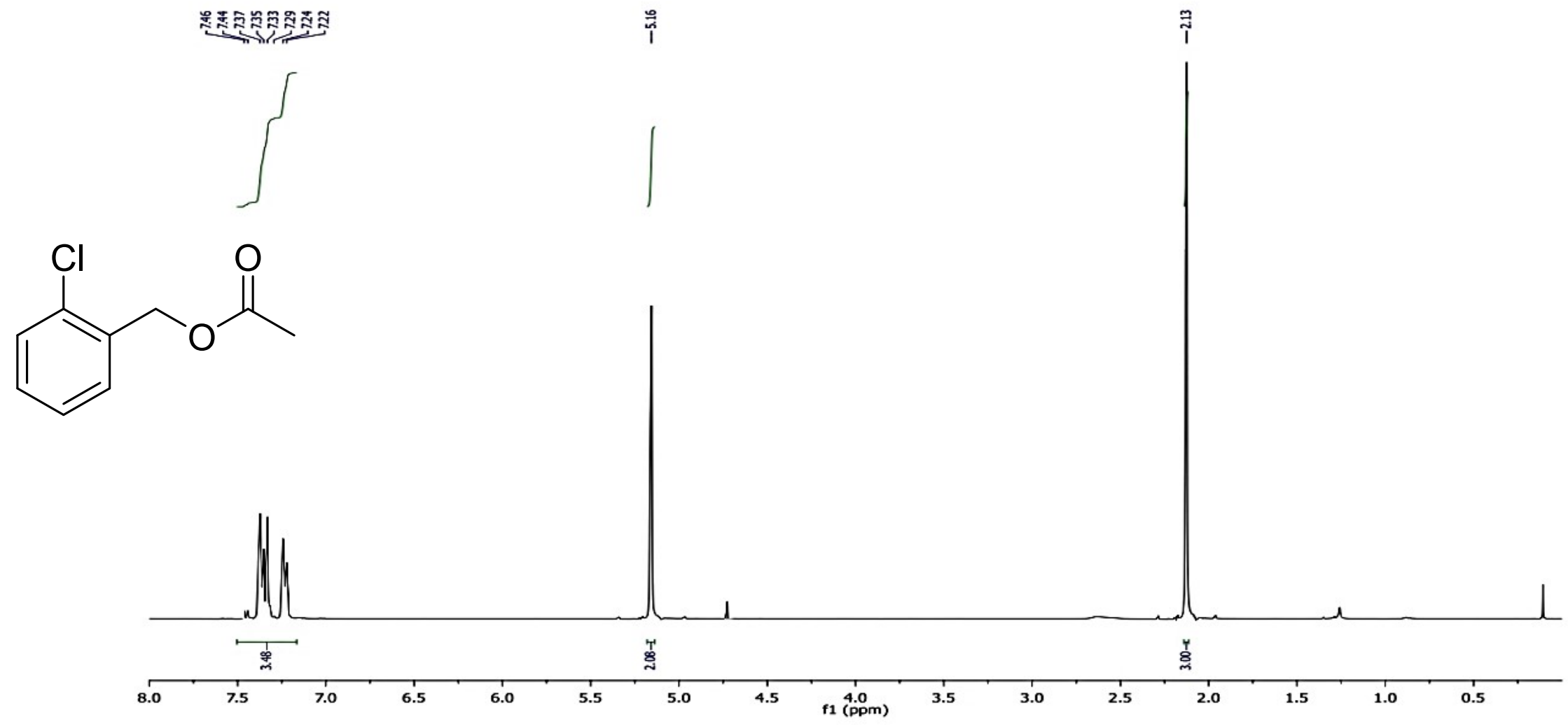

Figure S16. ^{1}H NMR spectrum of the 2-chlorobenzyl acetate (**5c**) product in $CDCl_3$

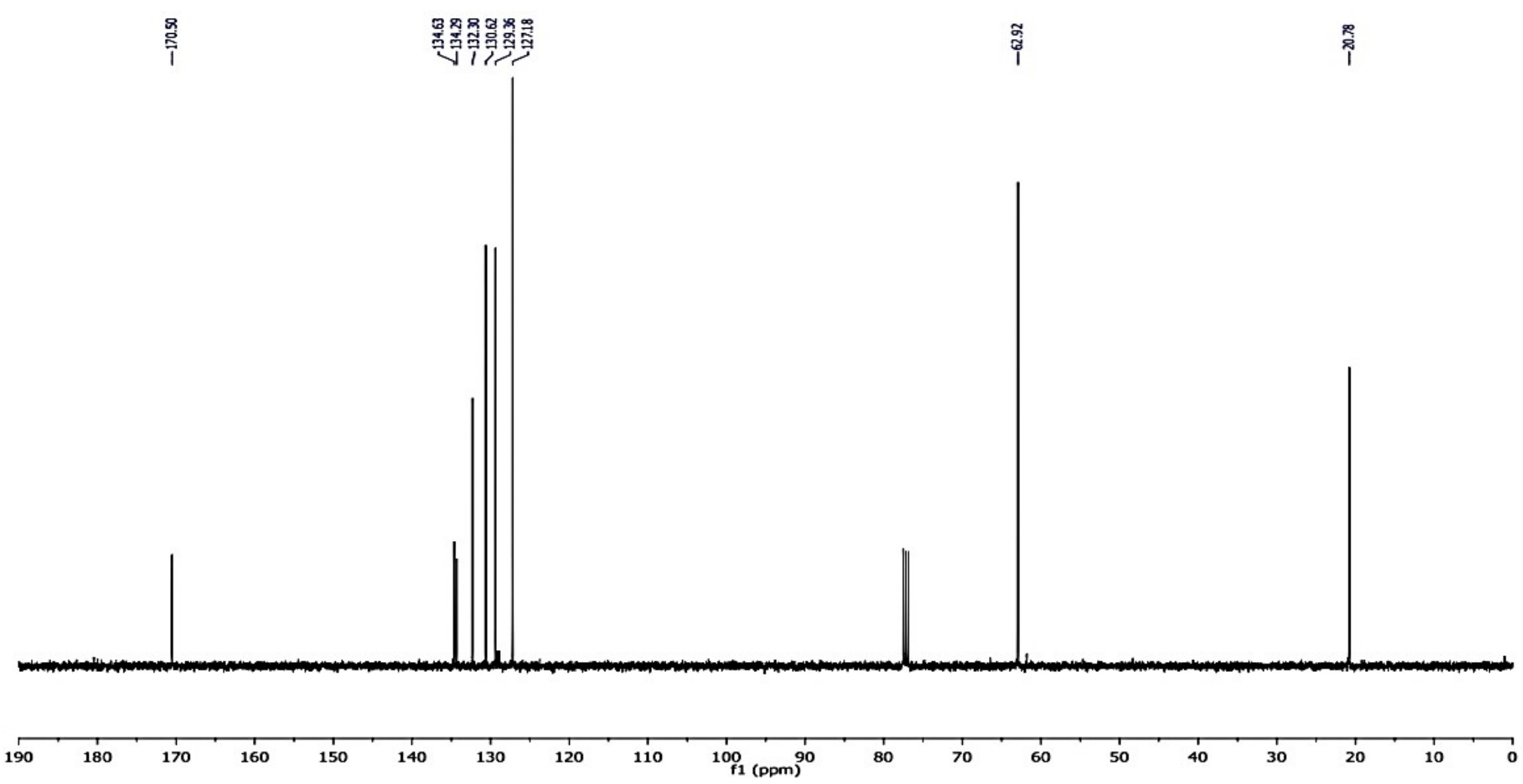

Figure S17. ^{13}C NMR spectrum of the 2-chlorobenzyl acetate (**5c**) product in $CDCl_3$

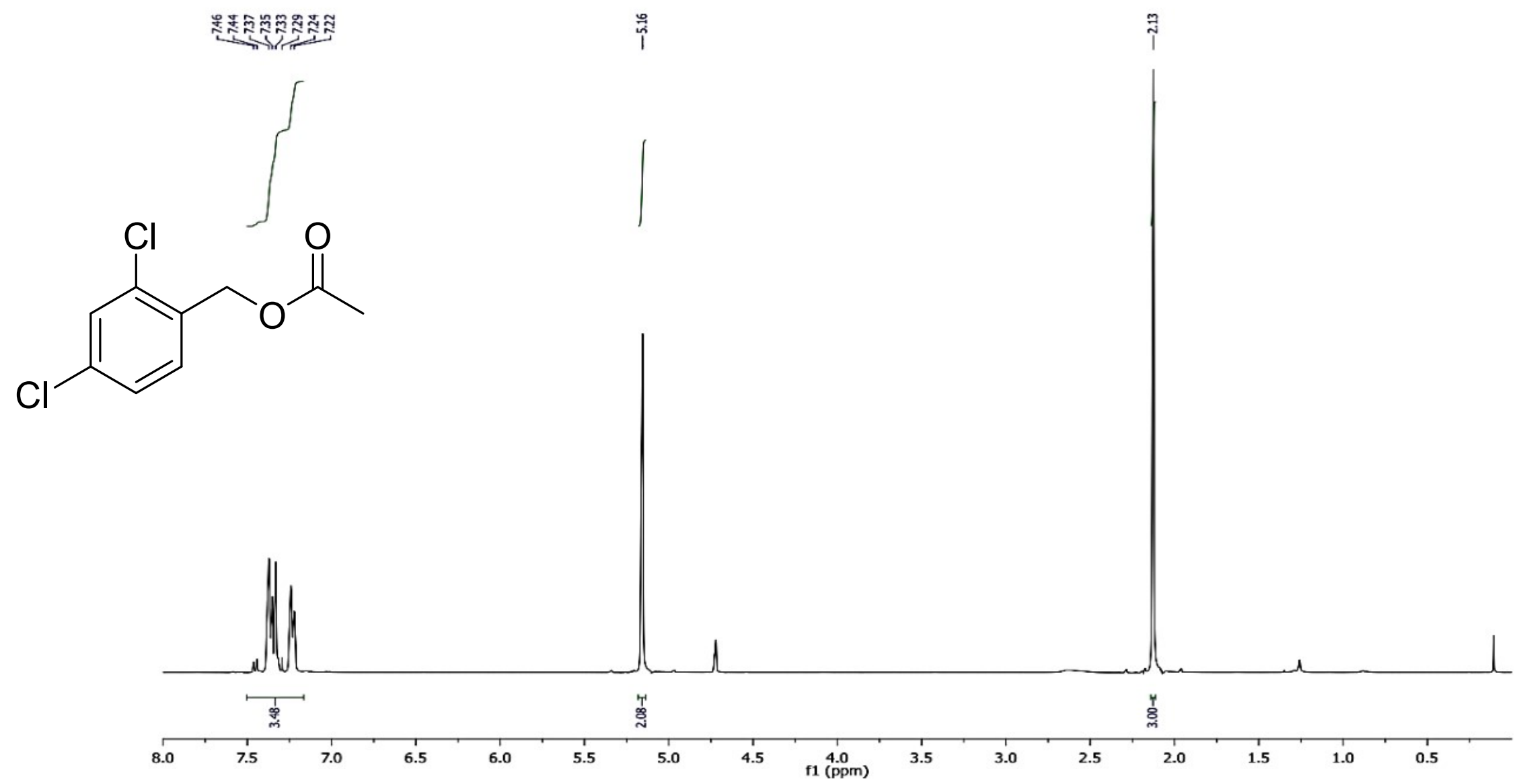

Figure S18. ^{1}H NMR spectrum of the 2,4-dichlorobenzyl acetate (**5d**) product in $CDCl_3$

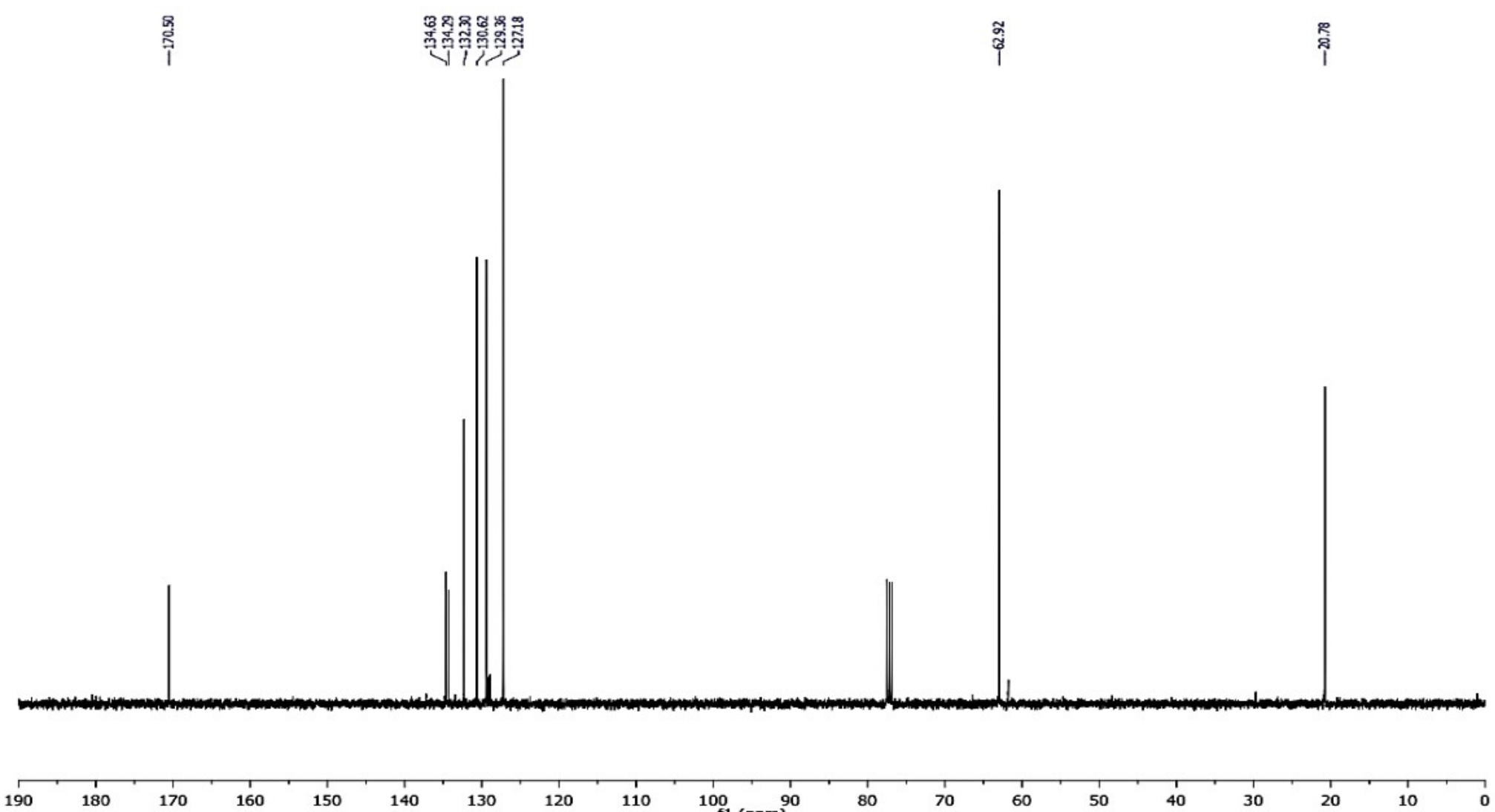

Figure S19. ^{13}C NMR spectrum of the 2,4-dichlorobenzyl acetate (**5d**) product in $CDCl_3$

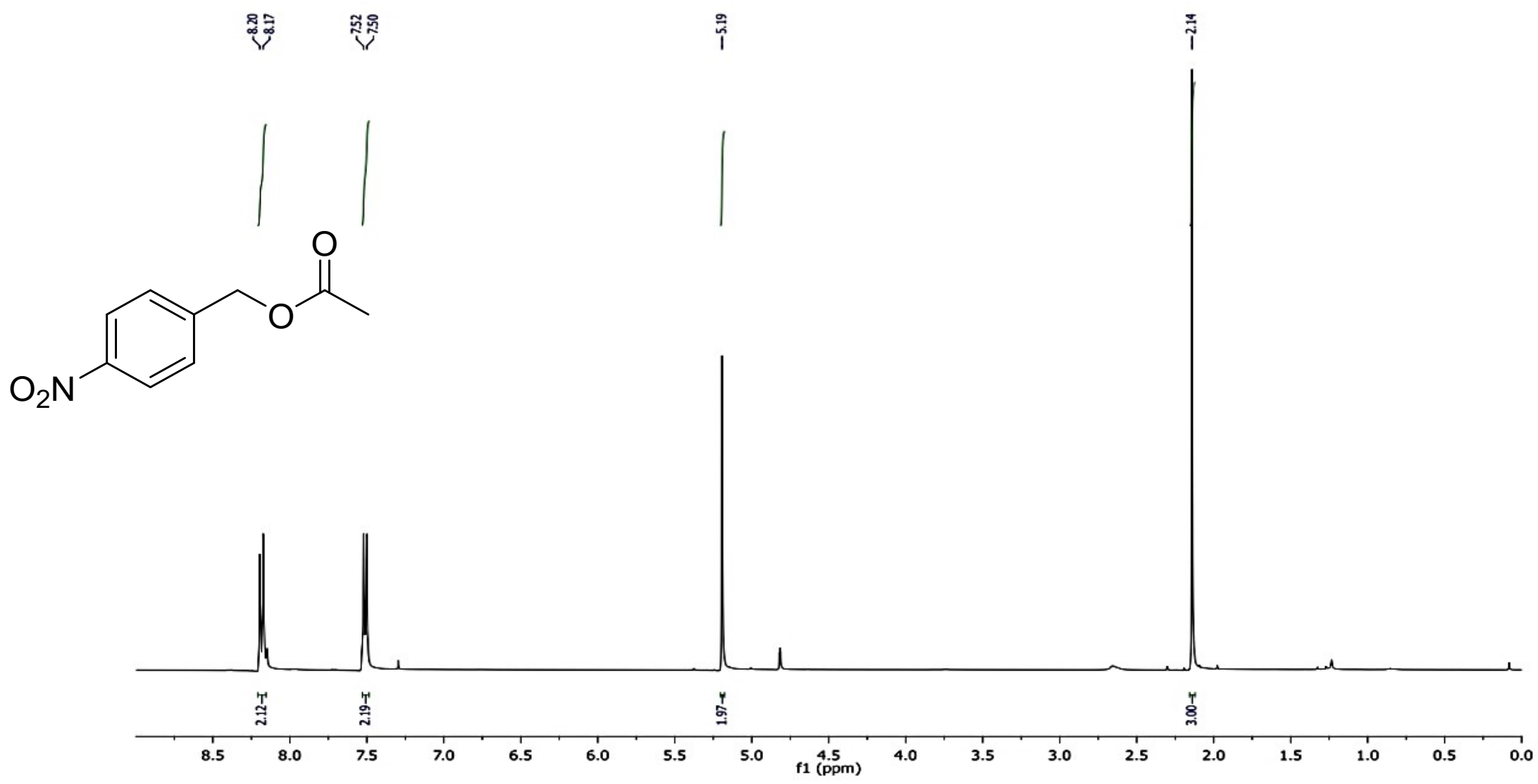

Figure S20. ^1H NMR spectrum of the 4-nitrobenzyl acetate (**5e**) product in $CDCl_3$

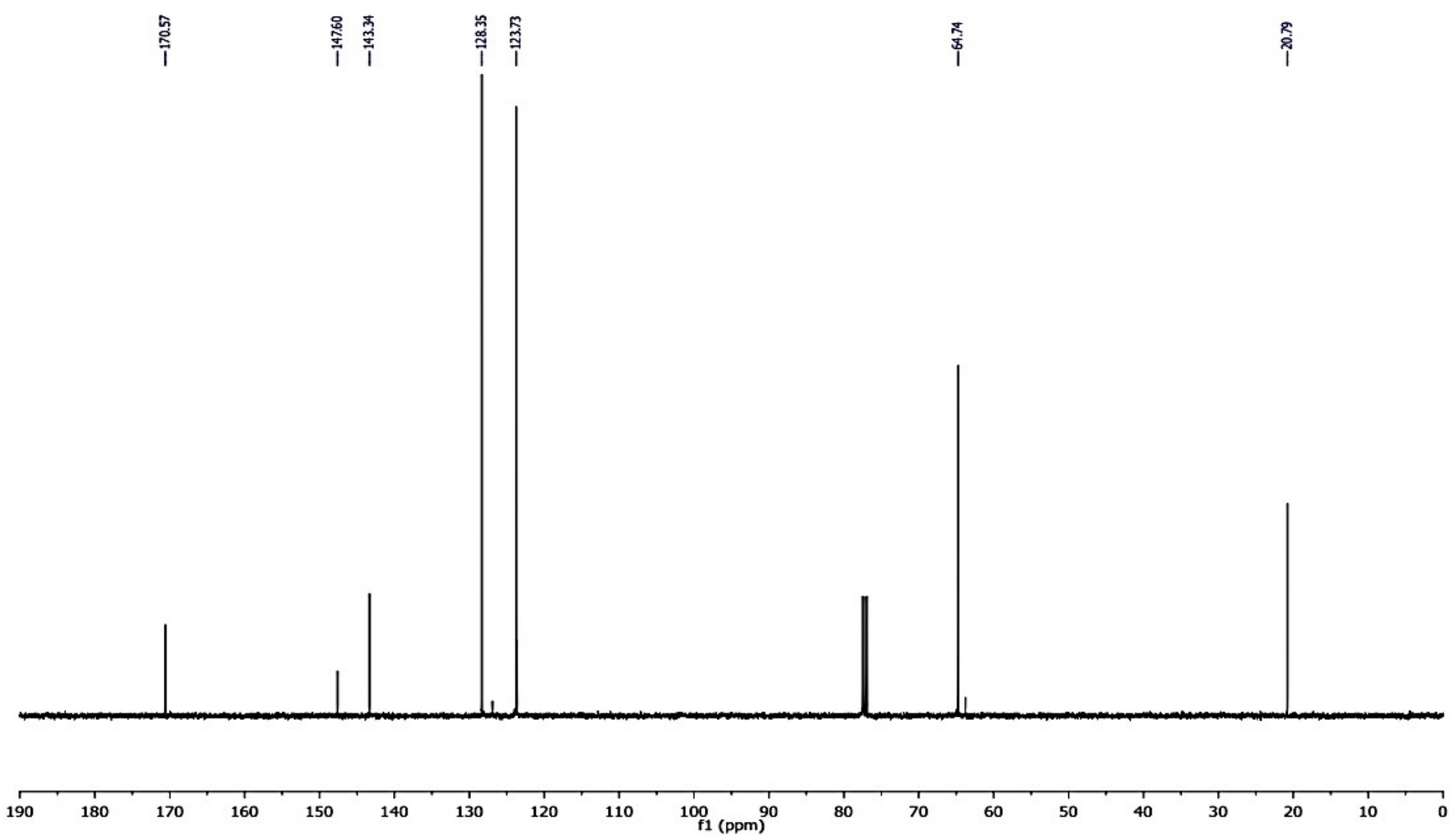

Figure S21. ^{13}C NMR spectrum of the 4-nitrobenzyl acetate (**5e**) product in $CDCl_3$

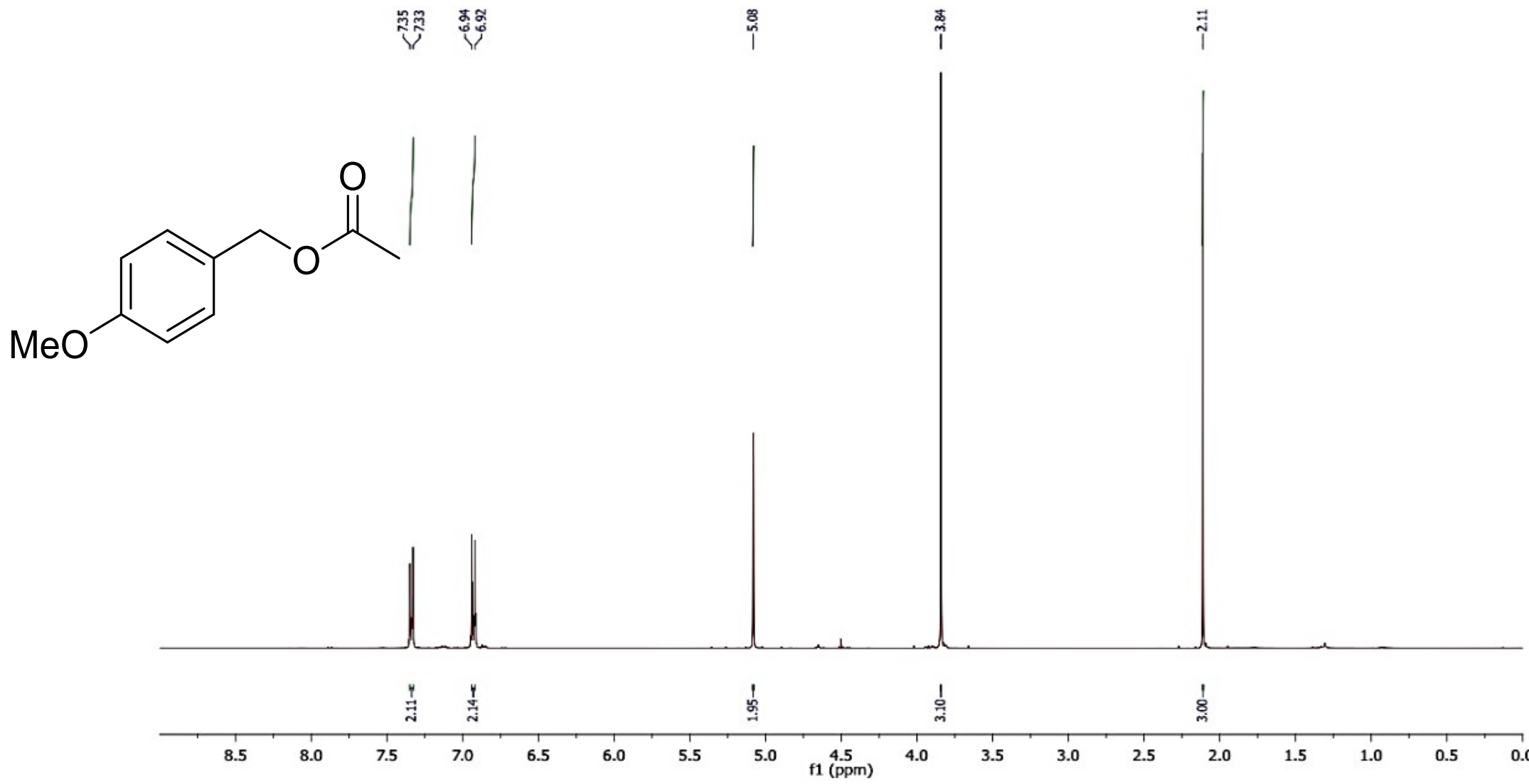

Figure S22. ^{1}H NMR spectrum of the 4-methoxybenzyl acetate (**5f**) product in $CDCl_3$

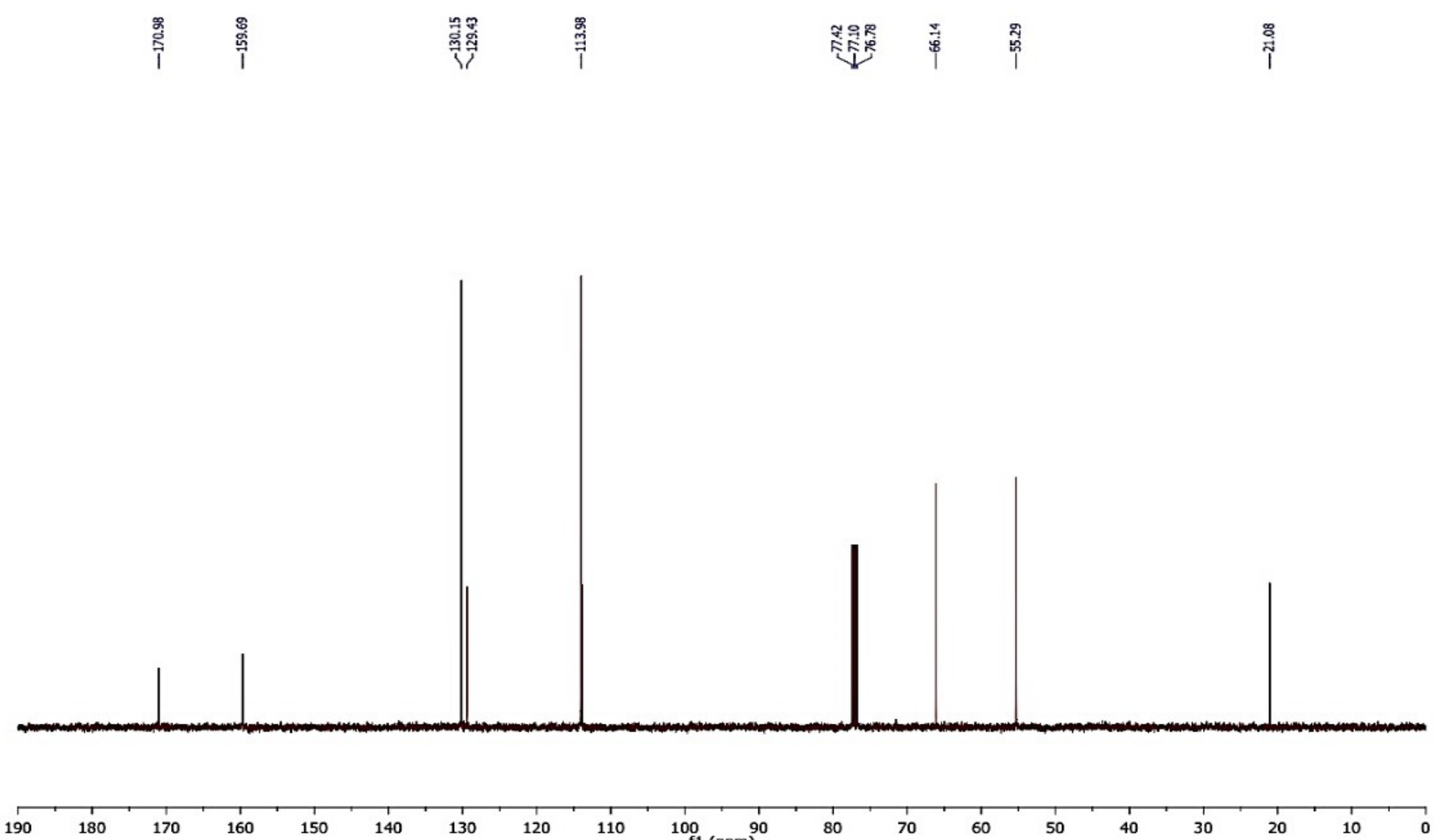

Figure S23. ^{13}C NMR spectrum of the 4-methoxybenzyl acetate (**5f**) product in $CDCl_3$

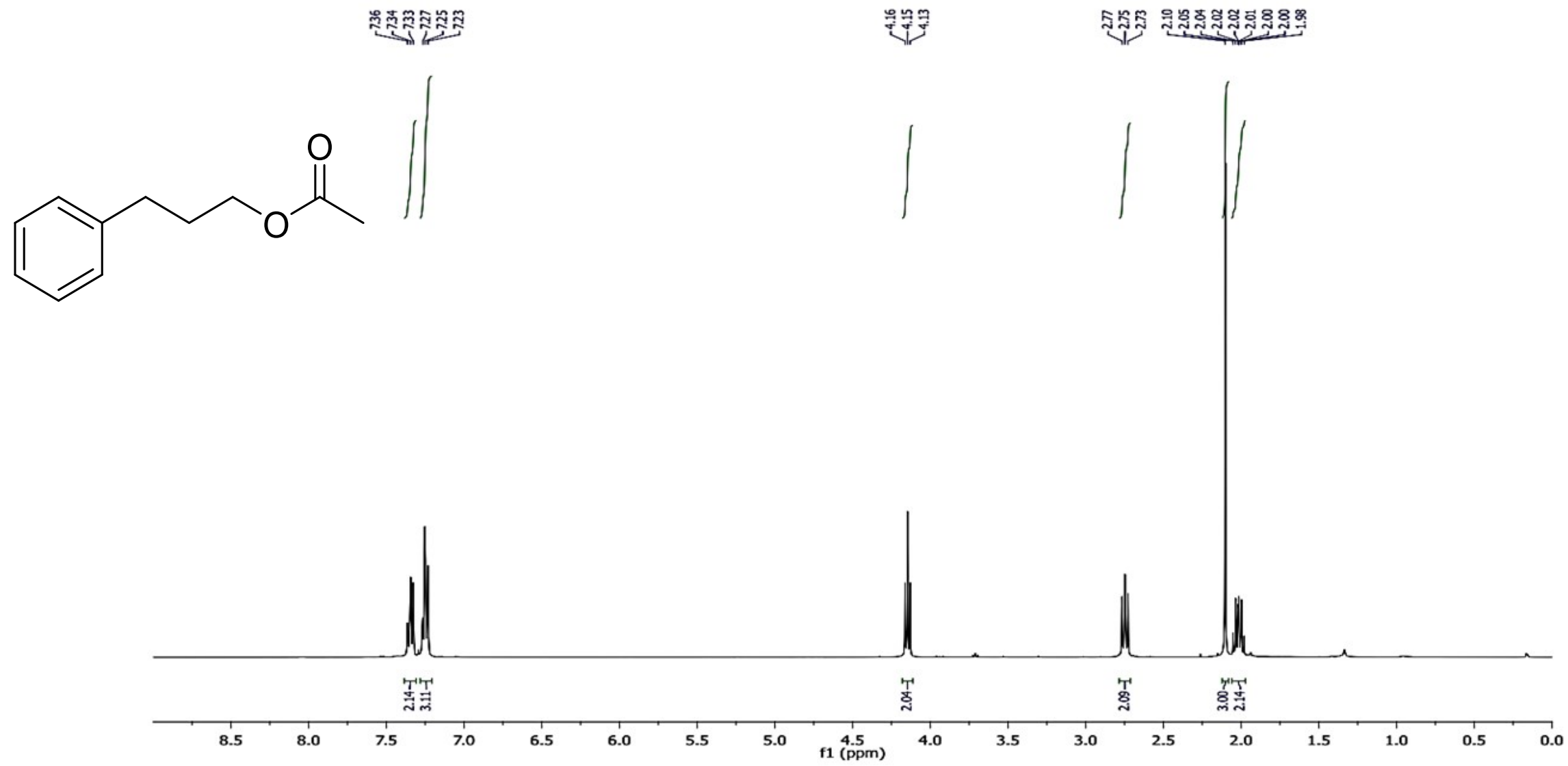

Figure S24. ^{1}H NMR spectrum of the 3-phenylpropyl acetate (**5g**) product in $CDCl_3$

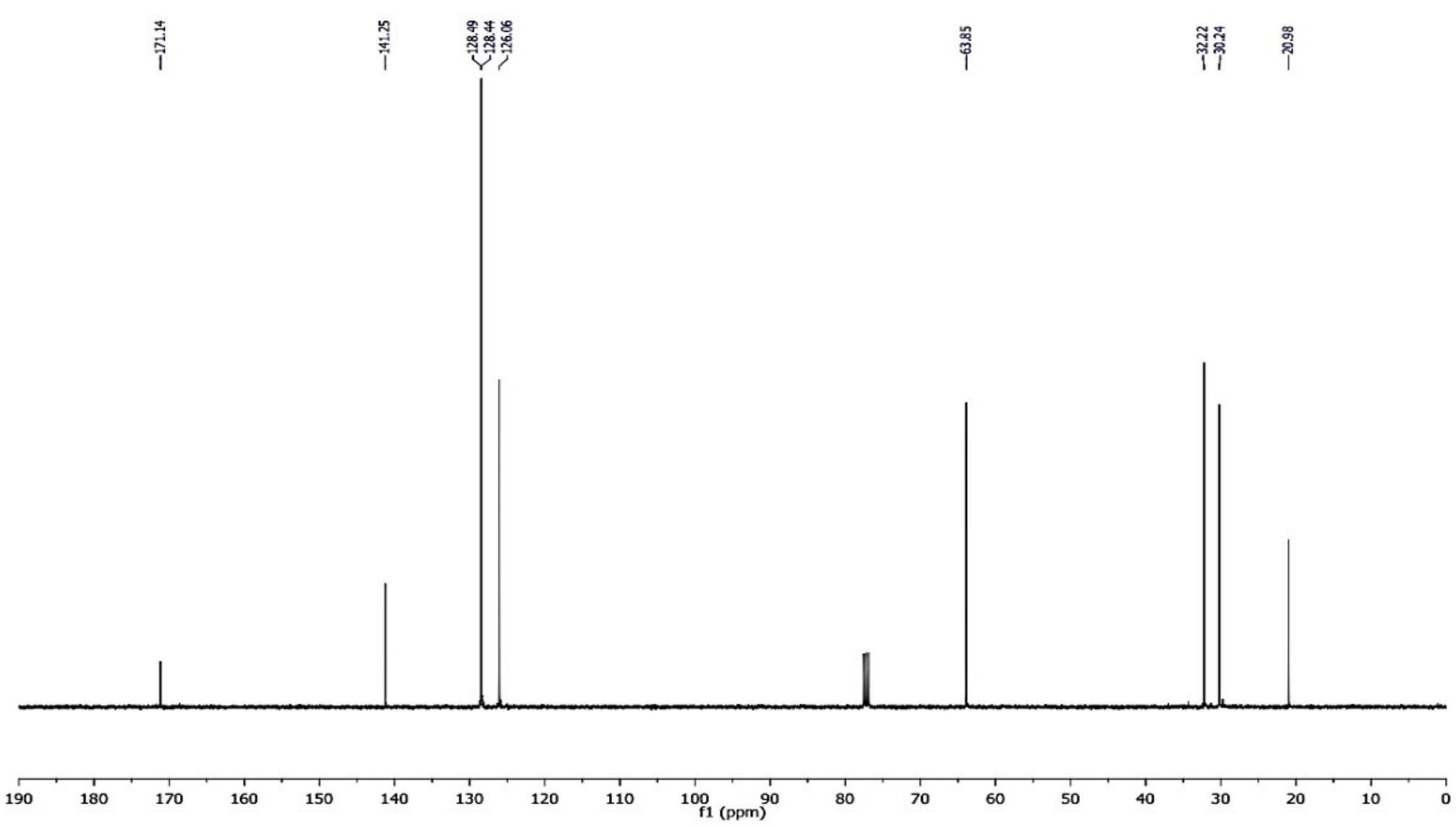

Figure S25. ^{13}C NMR spectrum of the 3-phenylpropyl acetate (**5g**) product in $CDCl_3$

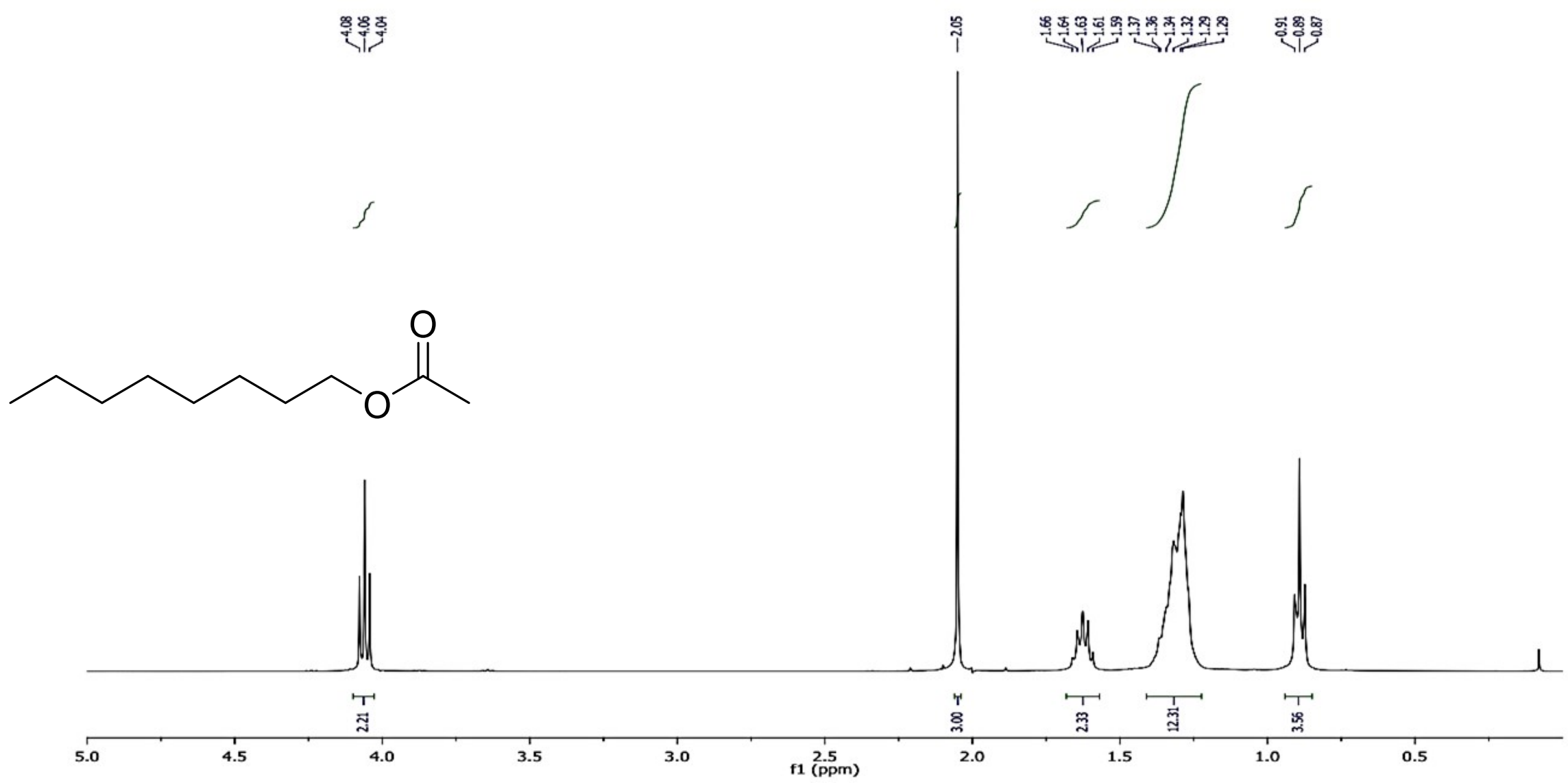

Figure S26. ^{1}H NMR spectrum of the octyl acetate (**5h**) product in $CDCl_3$

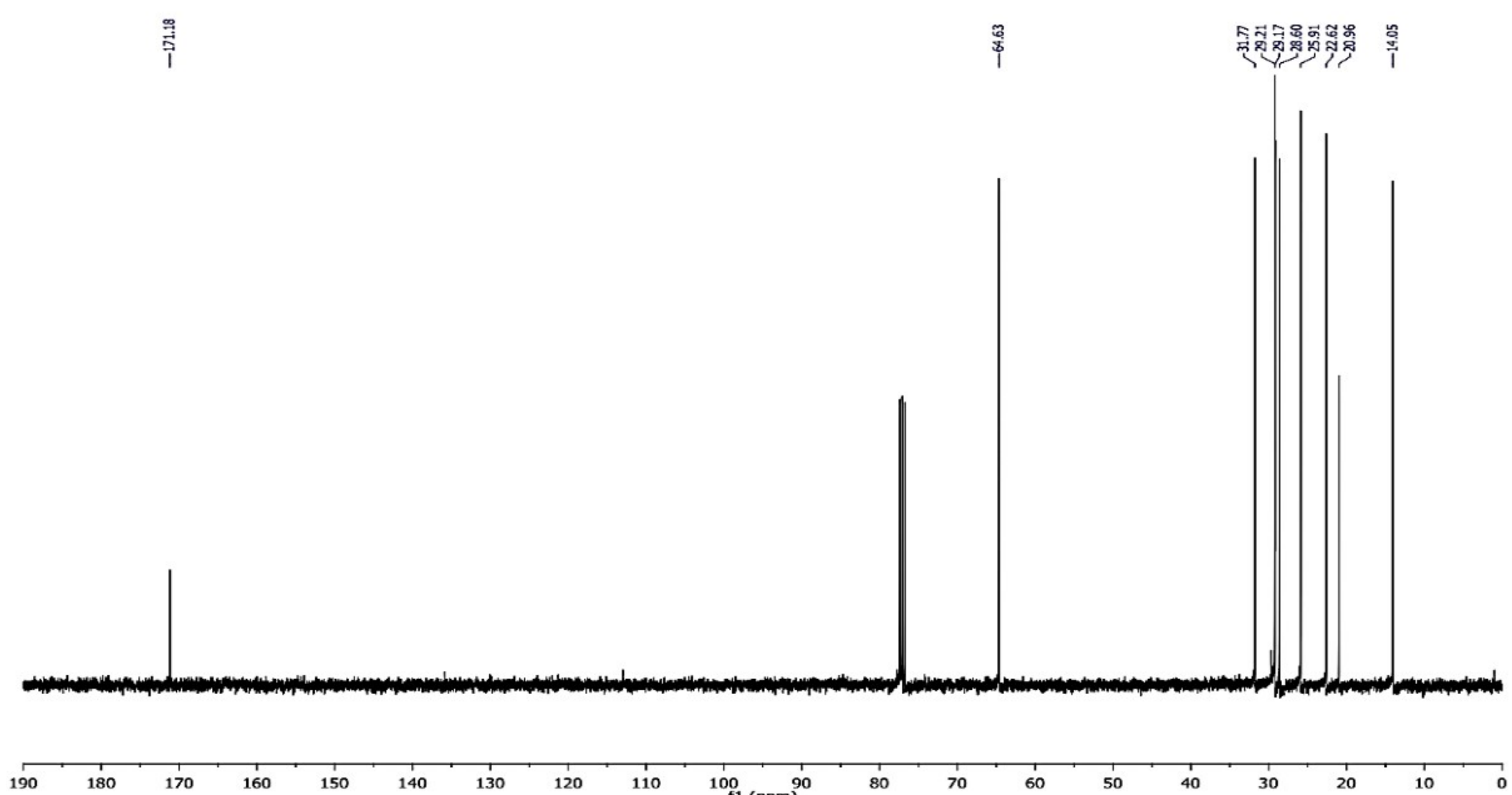

Figure S27. ^{13}C NMR spectrum of the octyl acetate (**5h**) product in $CDCl_3$

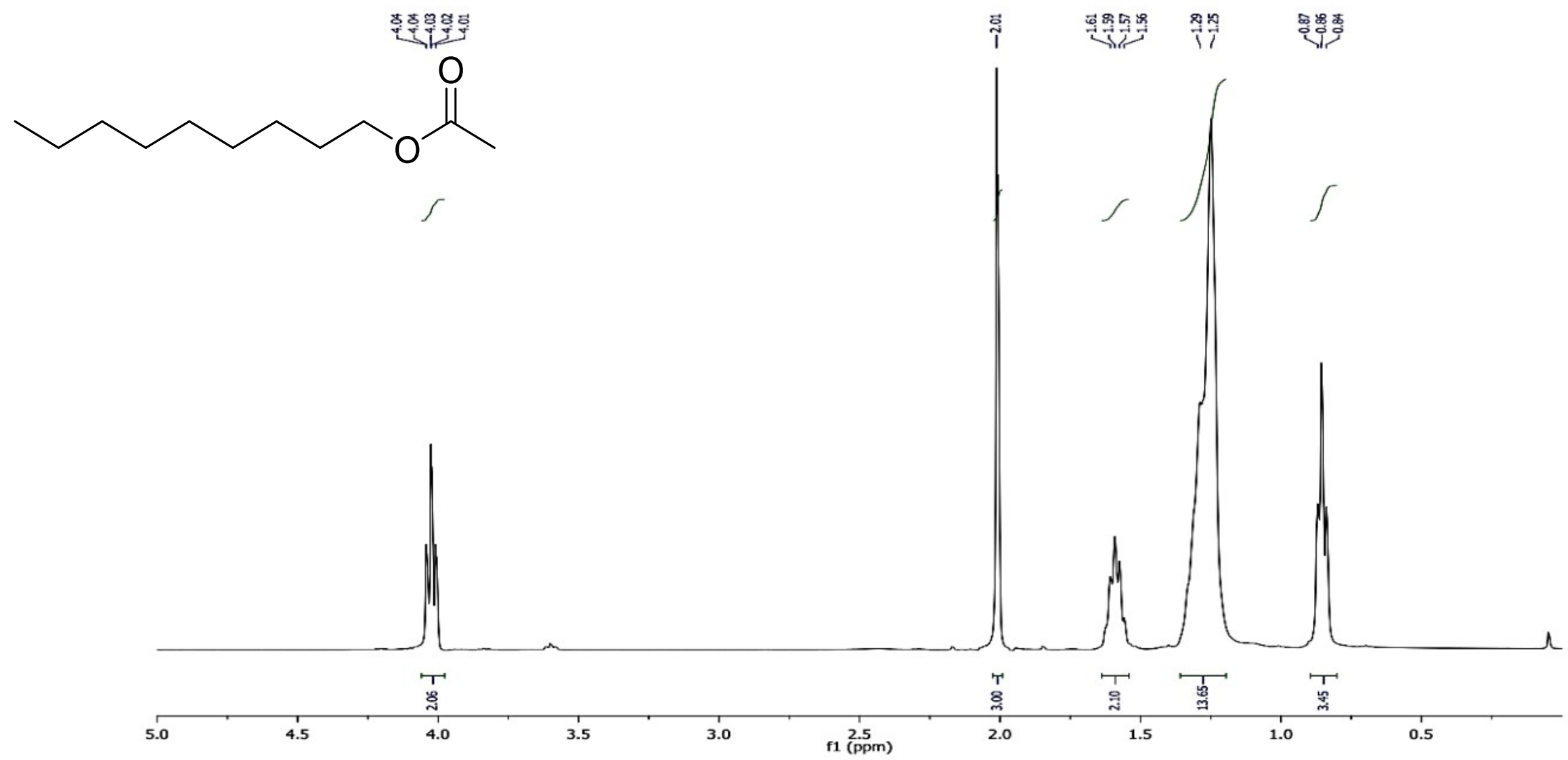

Figure S28. ^{1}H NMR spectrum of the nonyl acetate (**5i**) product in $CDCl_3$

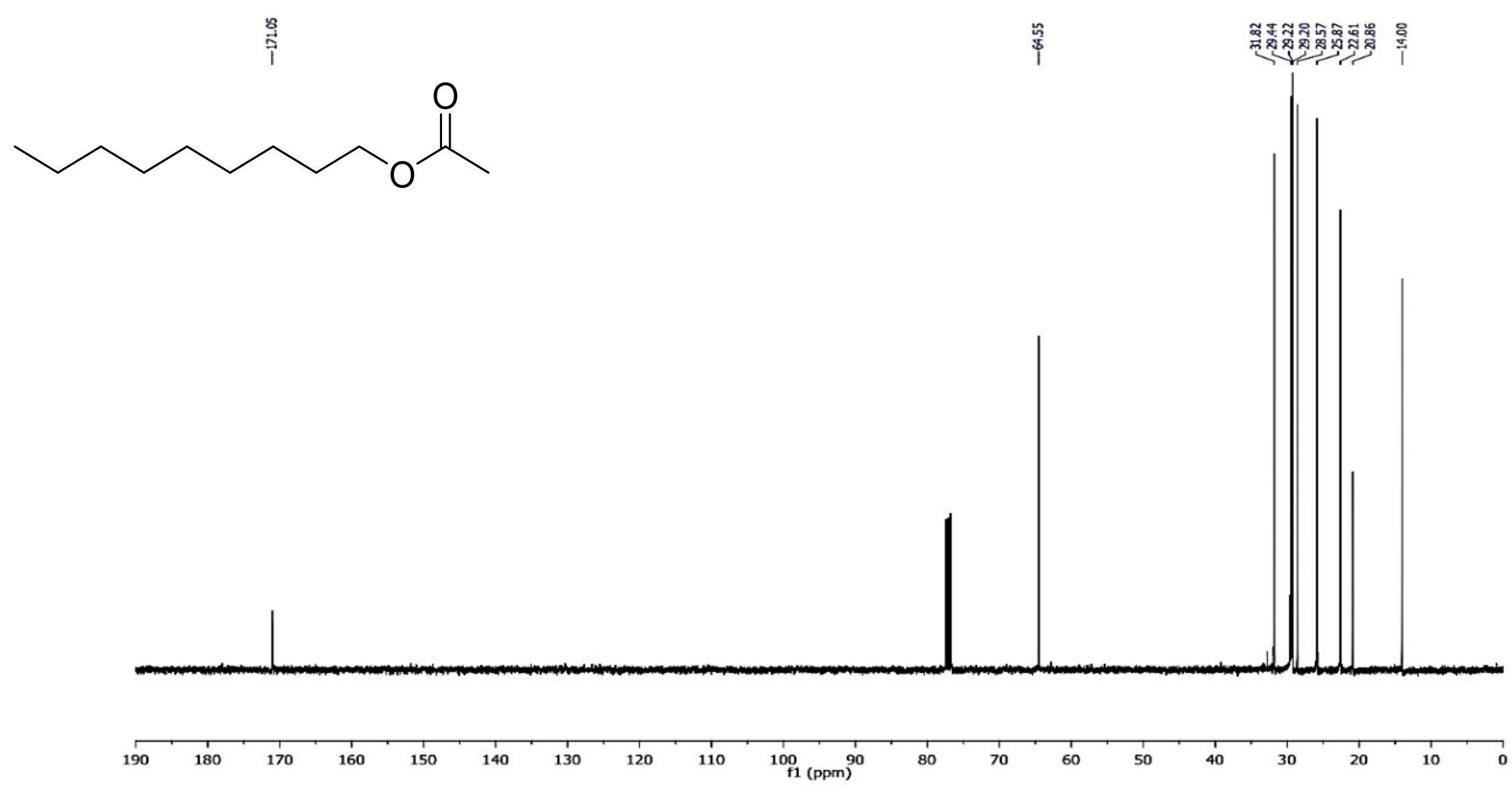

Figure S29. ^{13}C NMR spectrum of the nonyl acetate (**5i**) product in $CDCl_3$

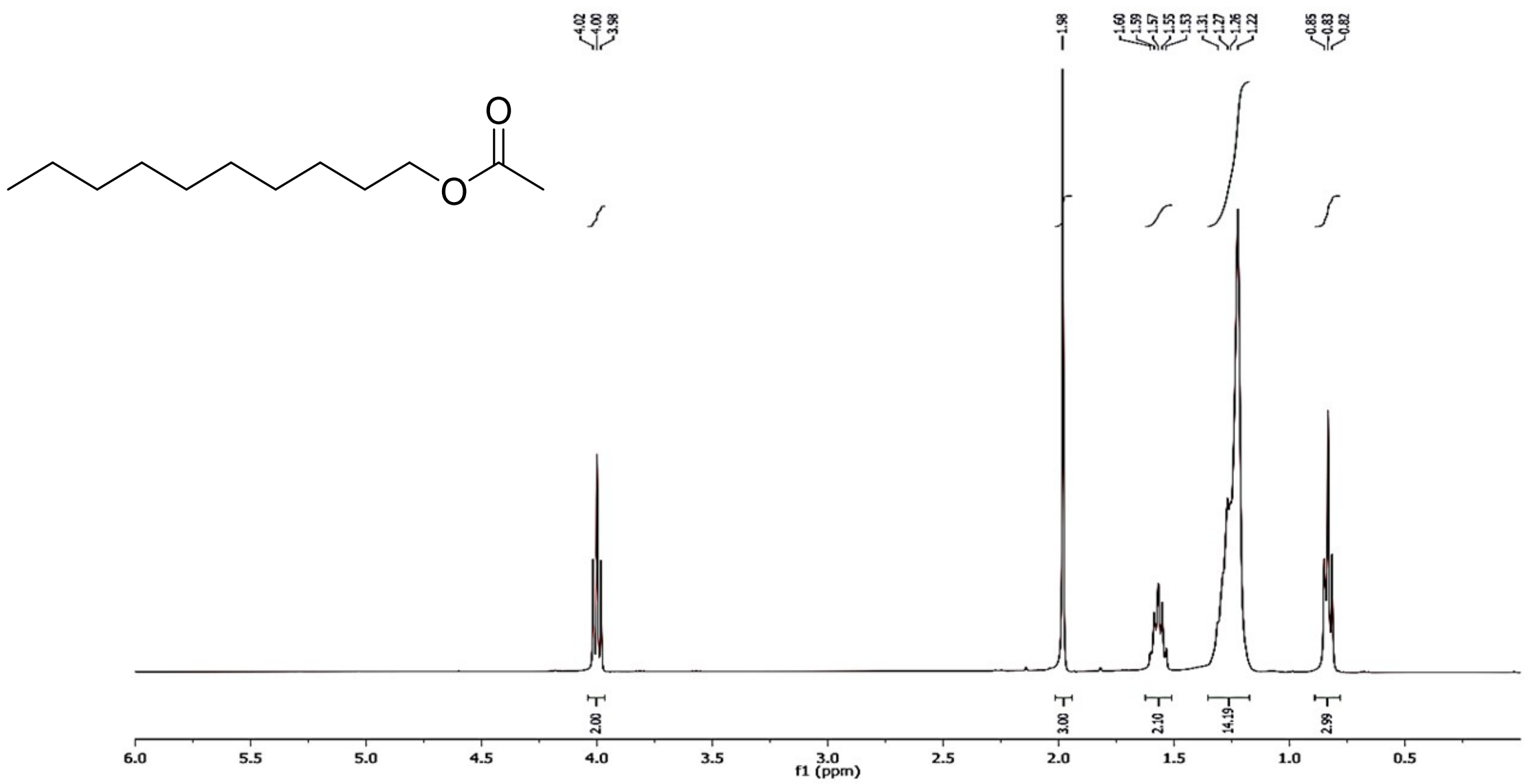

Figure S30. ^{1}H NMR spectrum of the decyl acetate (**5j**) product in $CDCl_3$

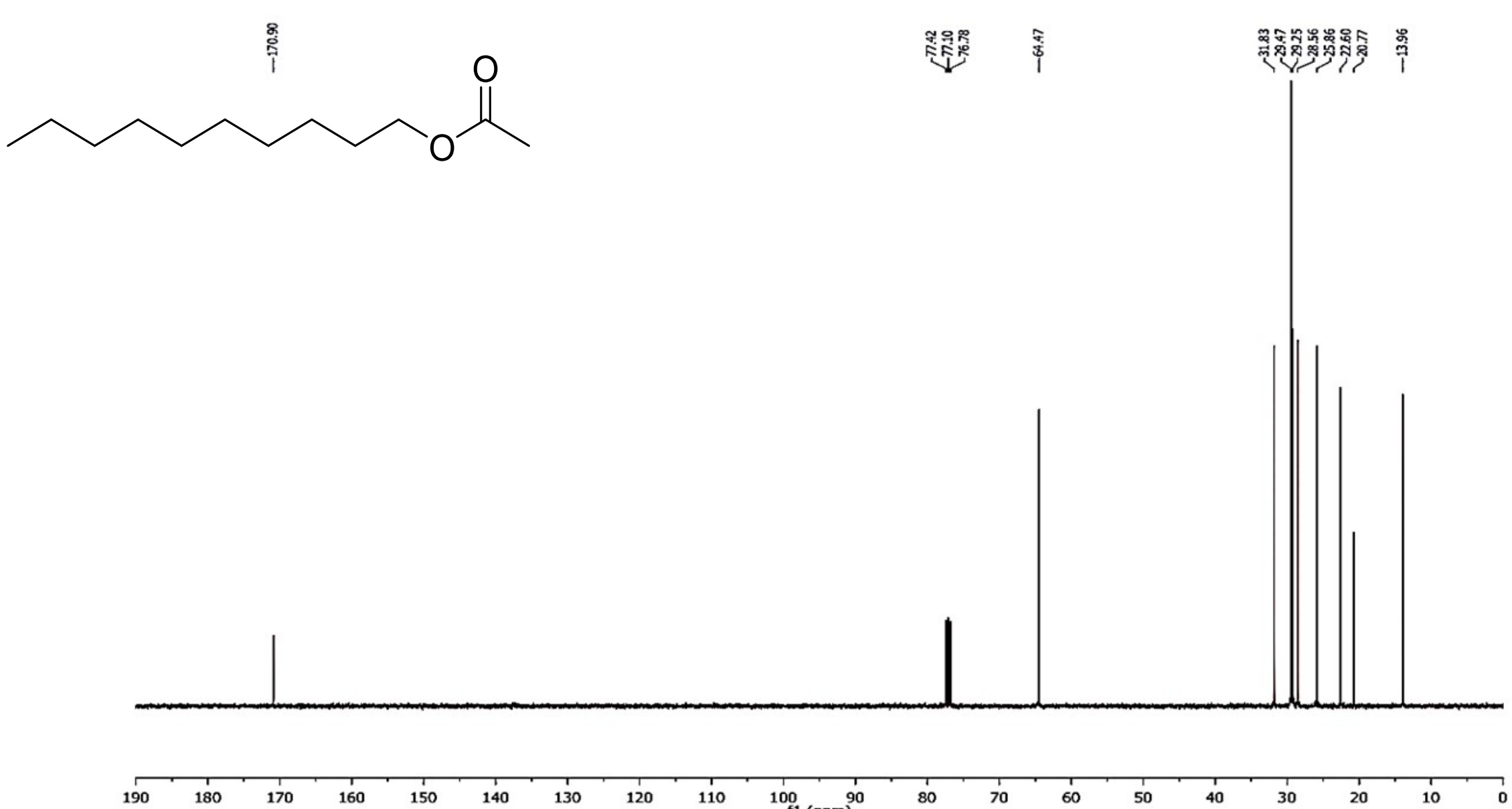

Figure S31. ^{13}C NMR spectrum of the decyl acetate (**5j**) product in $CDCl_3$

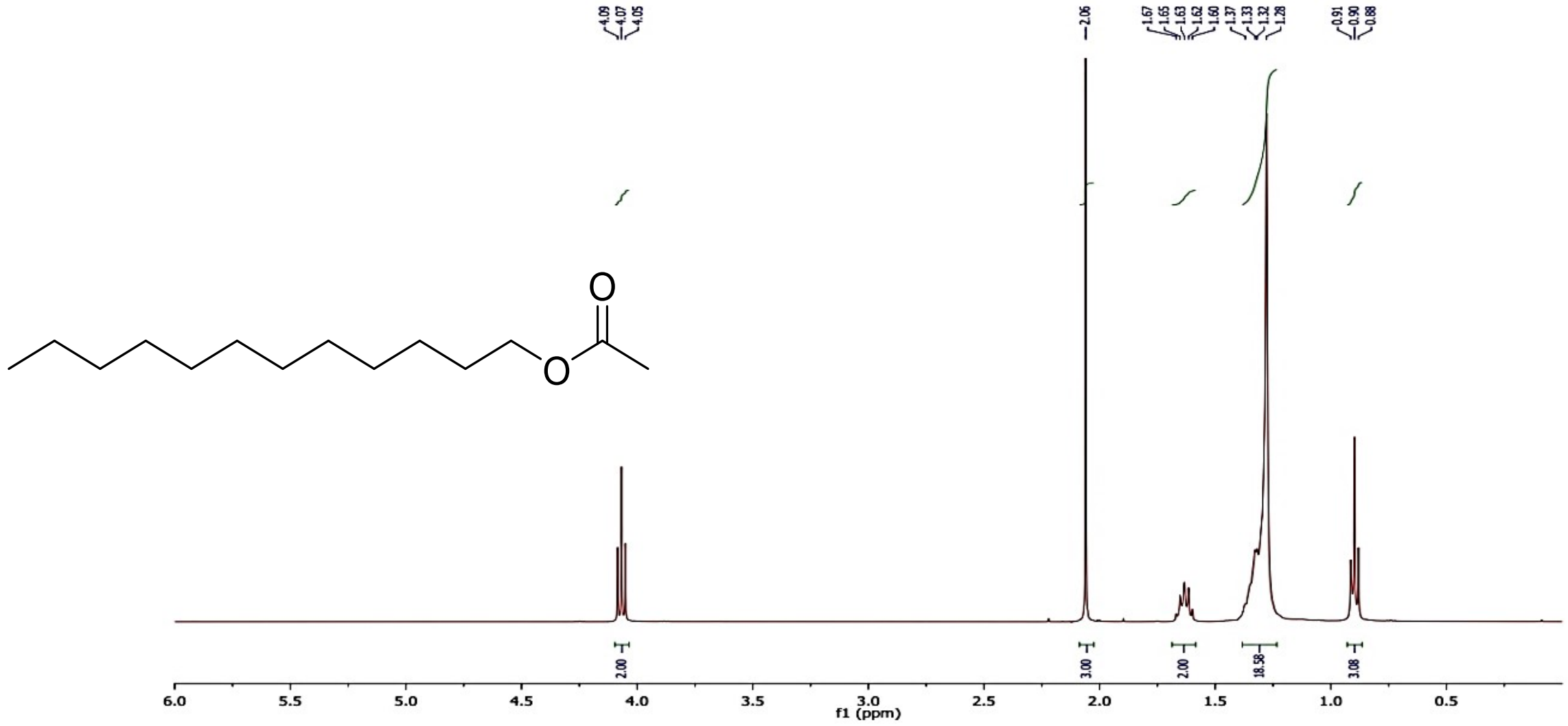

Figure S32. ^{1}H NMR spectrum of the dodecyl acetate (**5k**) product in $CDCl_3$

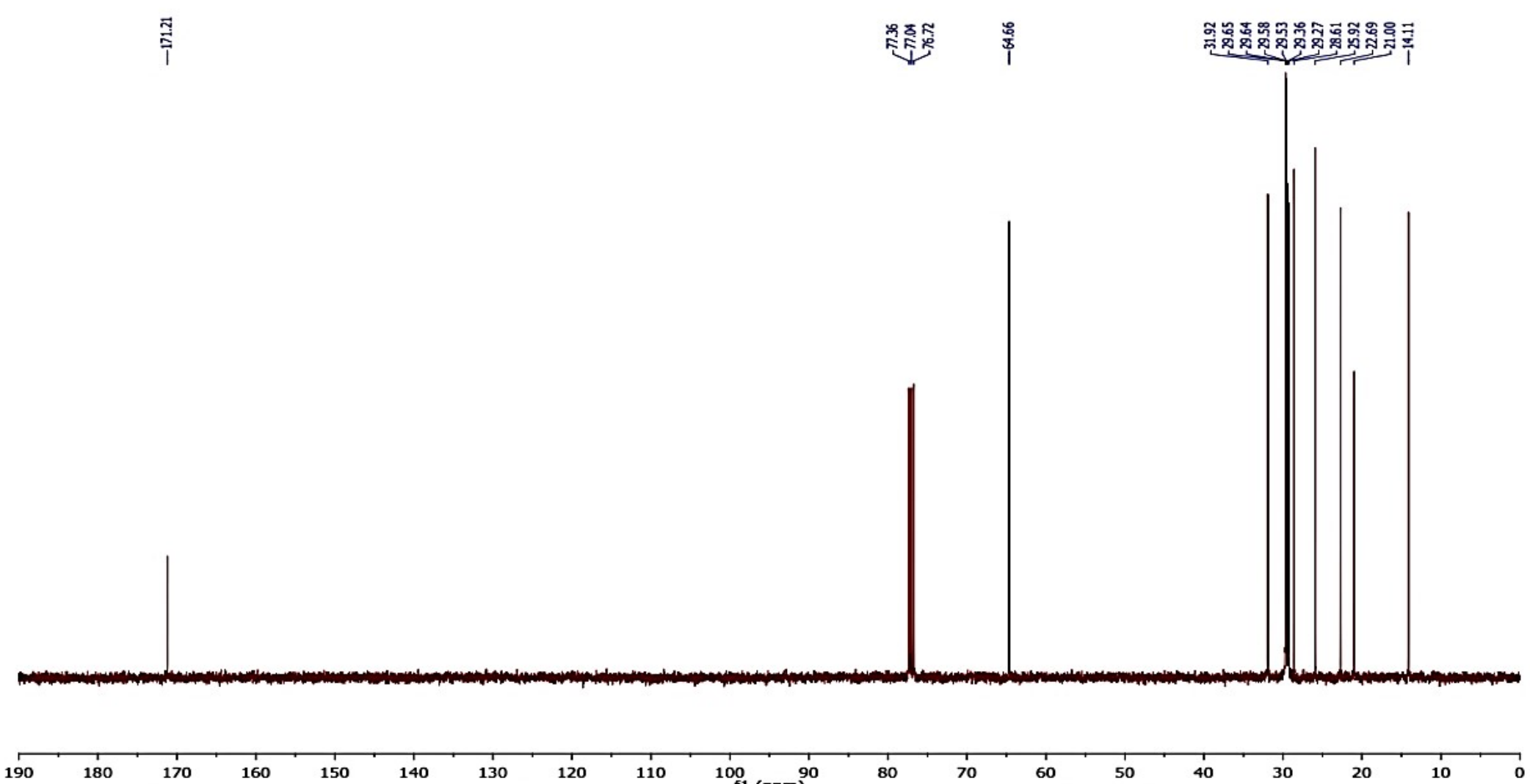

Figure S33. ^{13}C NMR spectrum of the dodecyl acetate (**5k**) product in $CDCl_3$

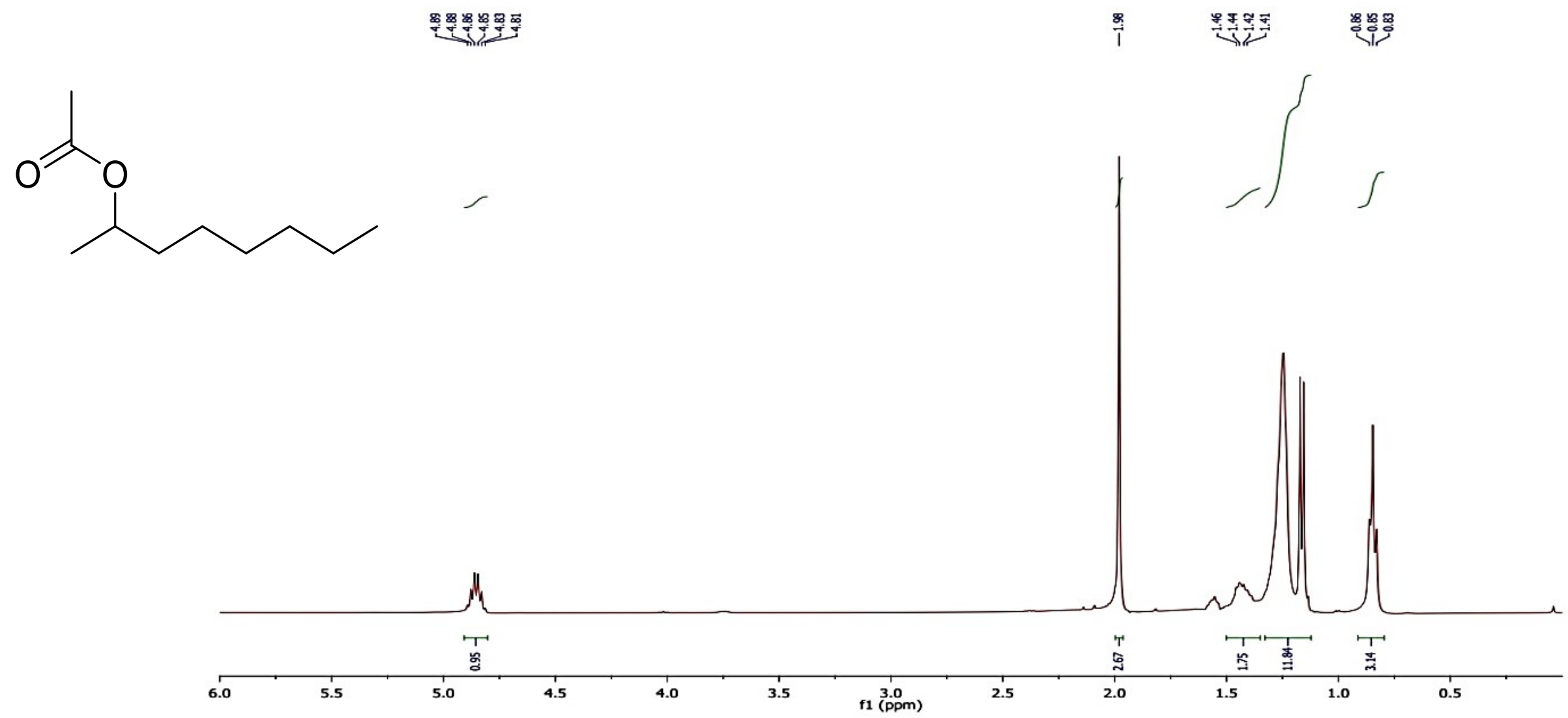

Figure S34. ^{1}H NMR spectrum of the 2-octyl acetate product (**5l**) in $CDCl_3$

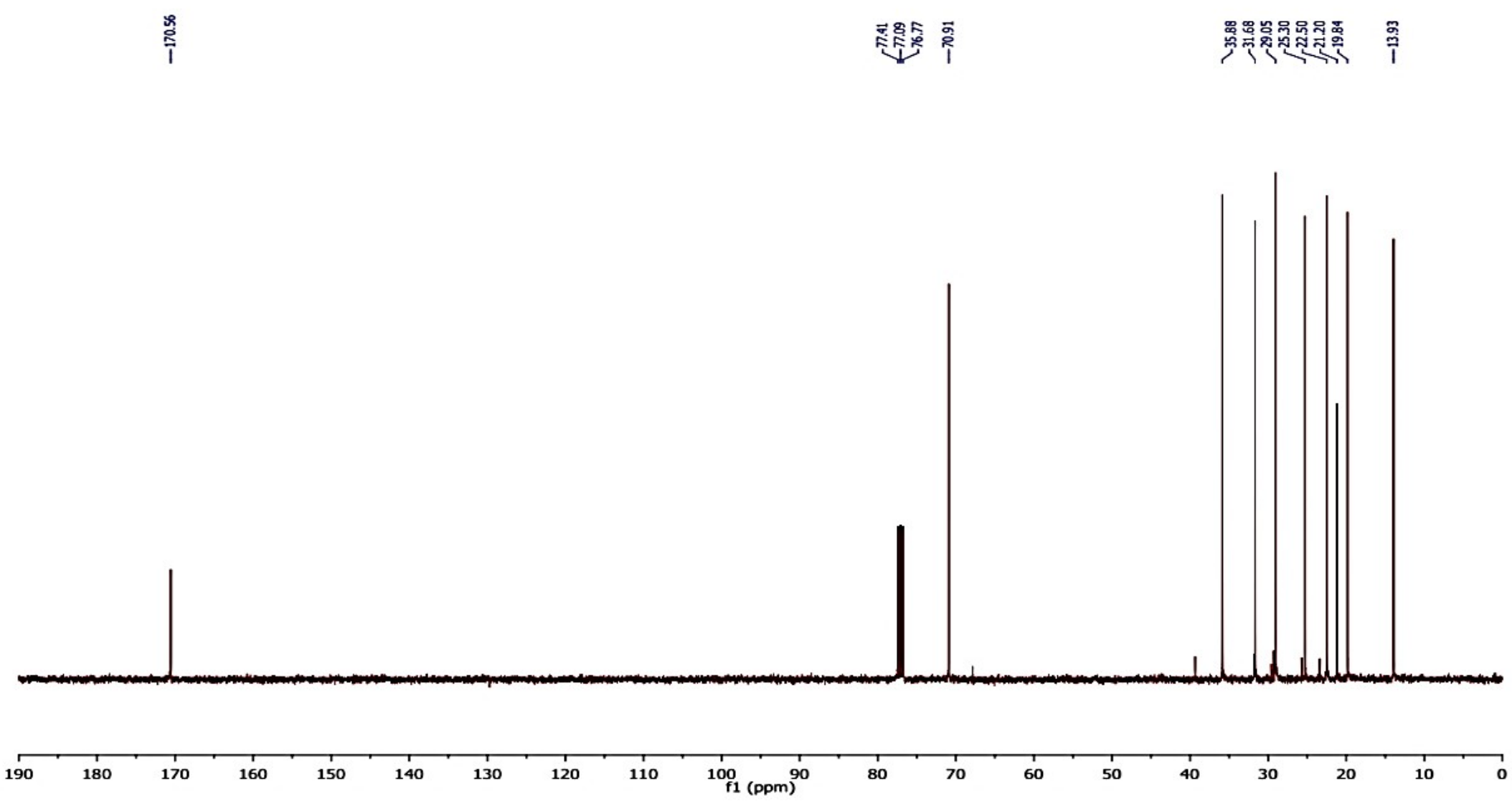

Figure S35. ^{13}C NMR spectrum of the 2-octyl acetate product (**5l**) in $CDCl_3$

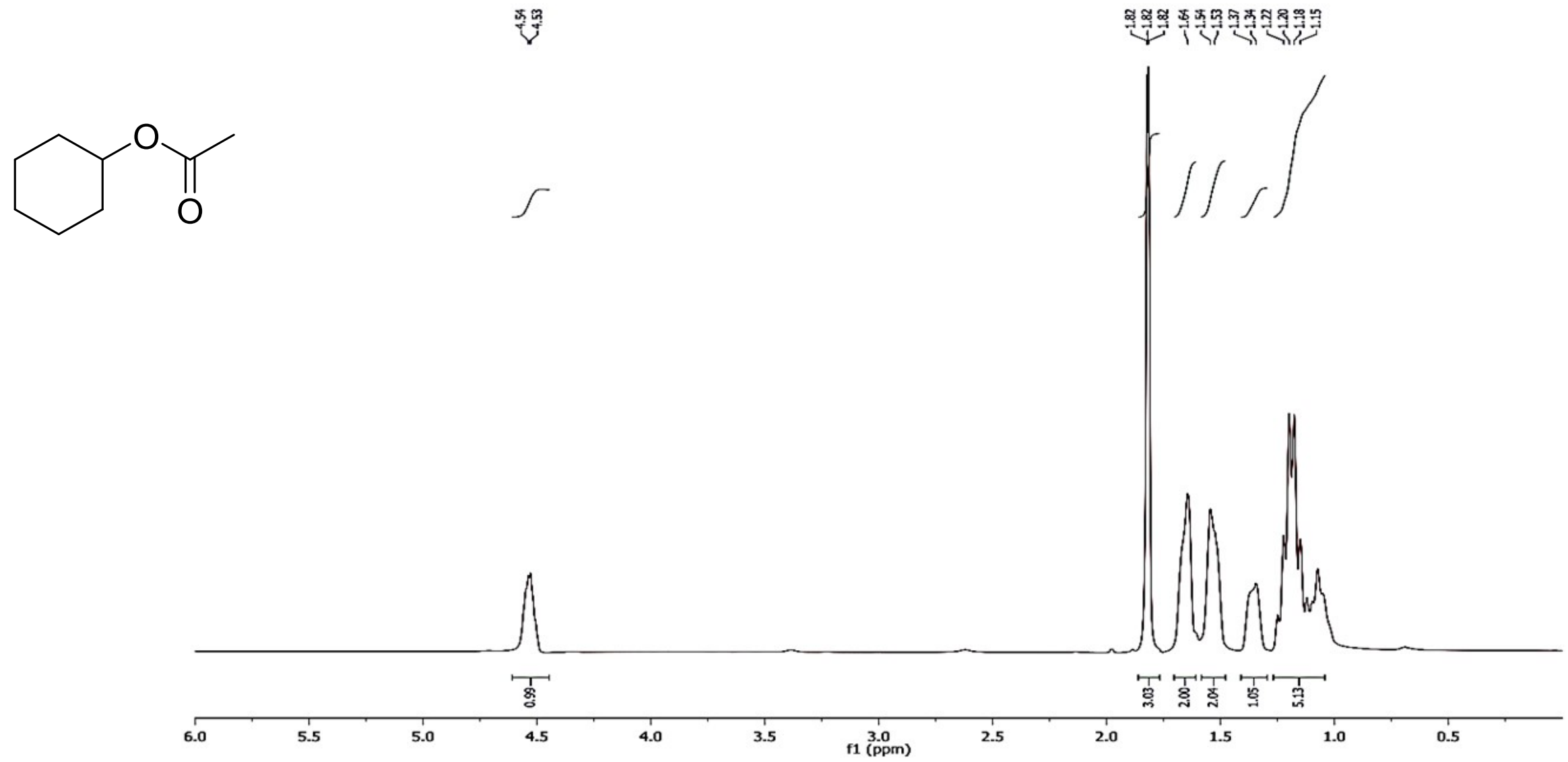

Figure S36. ^{1}H NMR spectrum of the cyclohexyl acetate (**5m**) product in $CDCl_3$

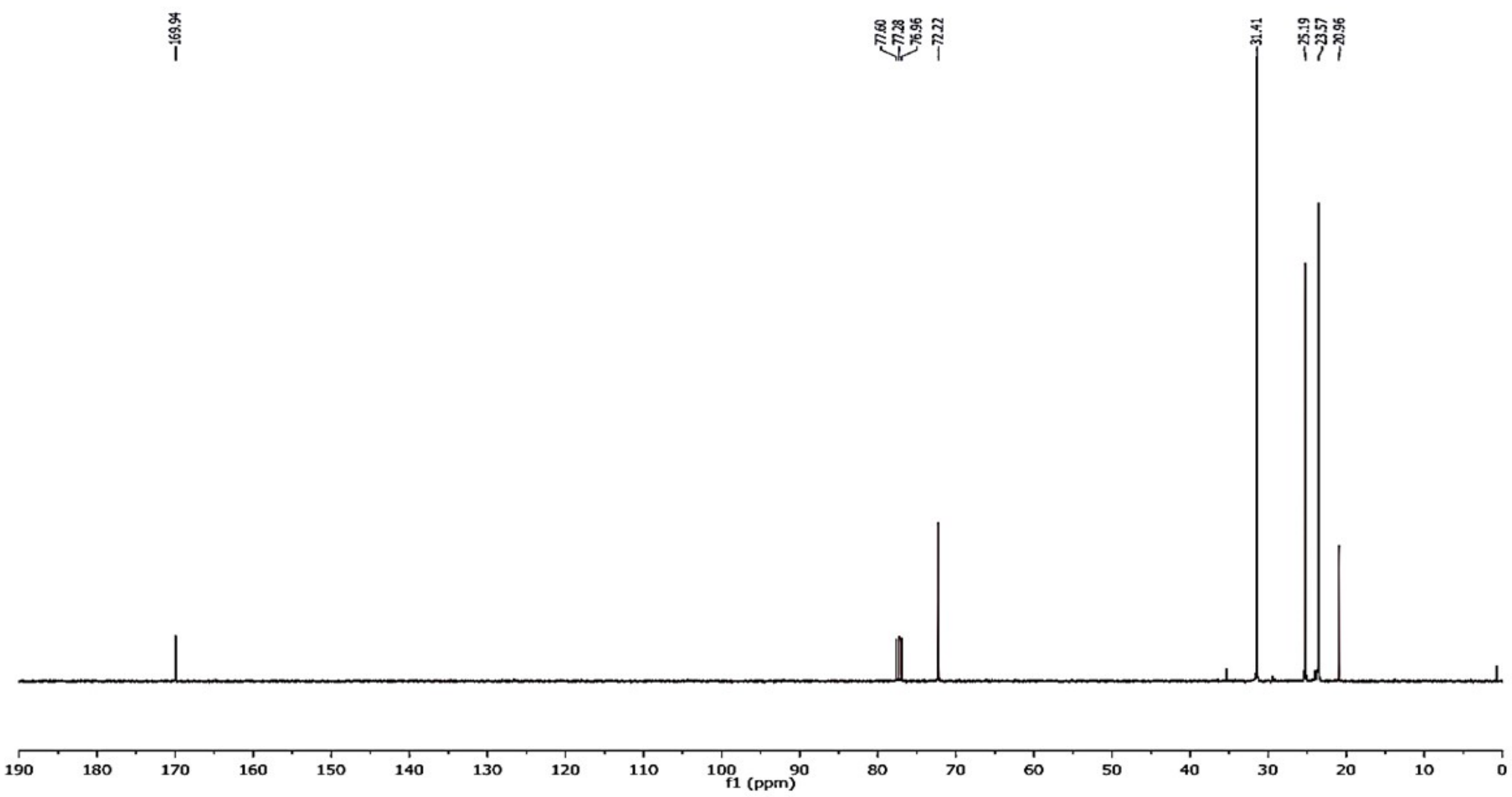

Figure S37. ^{13}C NMR spectrum of the cyclohexyl acetate (**5m**) product in $CDCl_3$

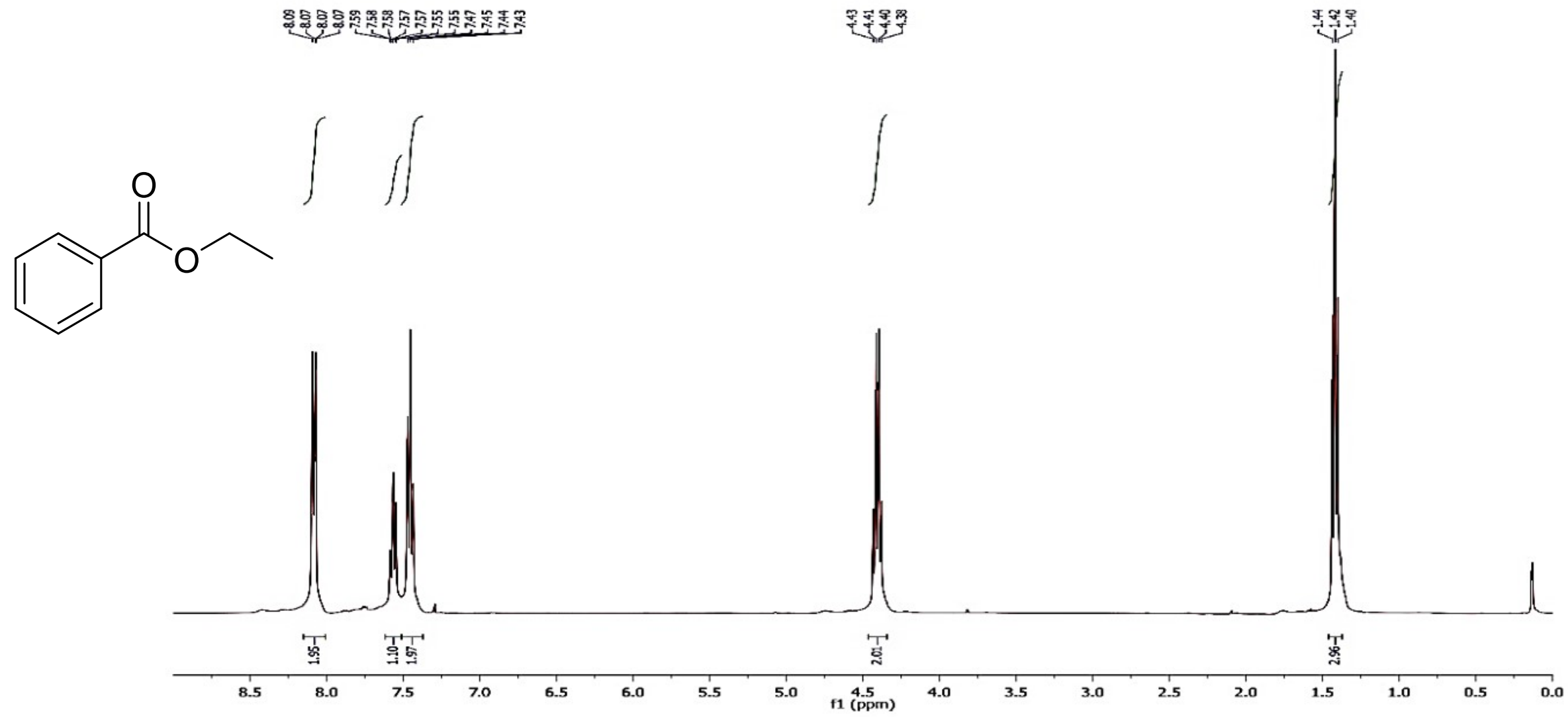

Figure S38. ^{1}H NMR spectrum of the ethyl benzoate (**7a**) product in $CDCl_3$

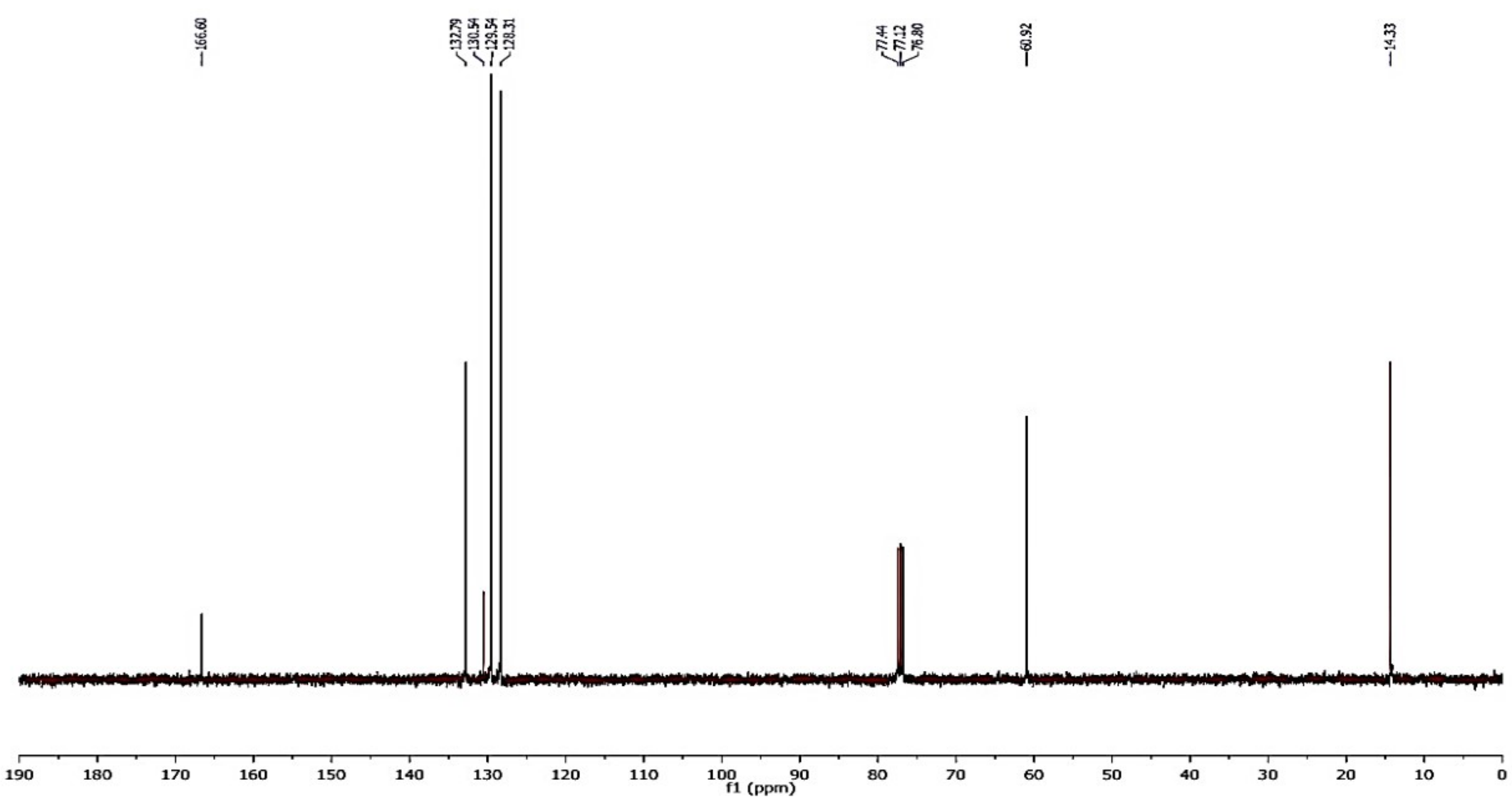

Figure S39. ^{13}C NMR spectrum of the ethyl benzoate (**7a**) product in $CDCl_3$

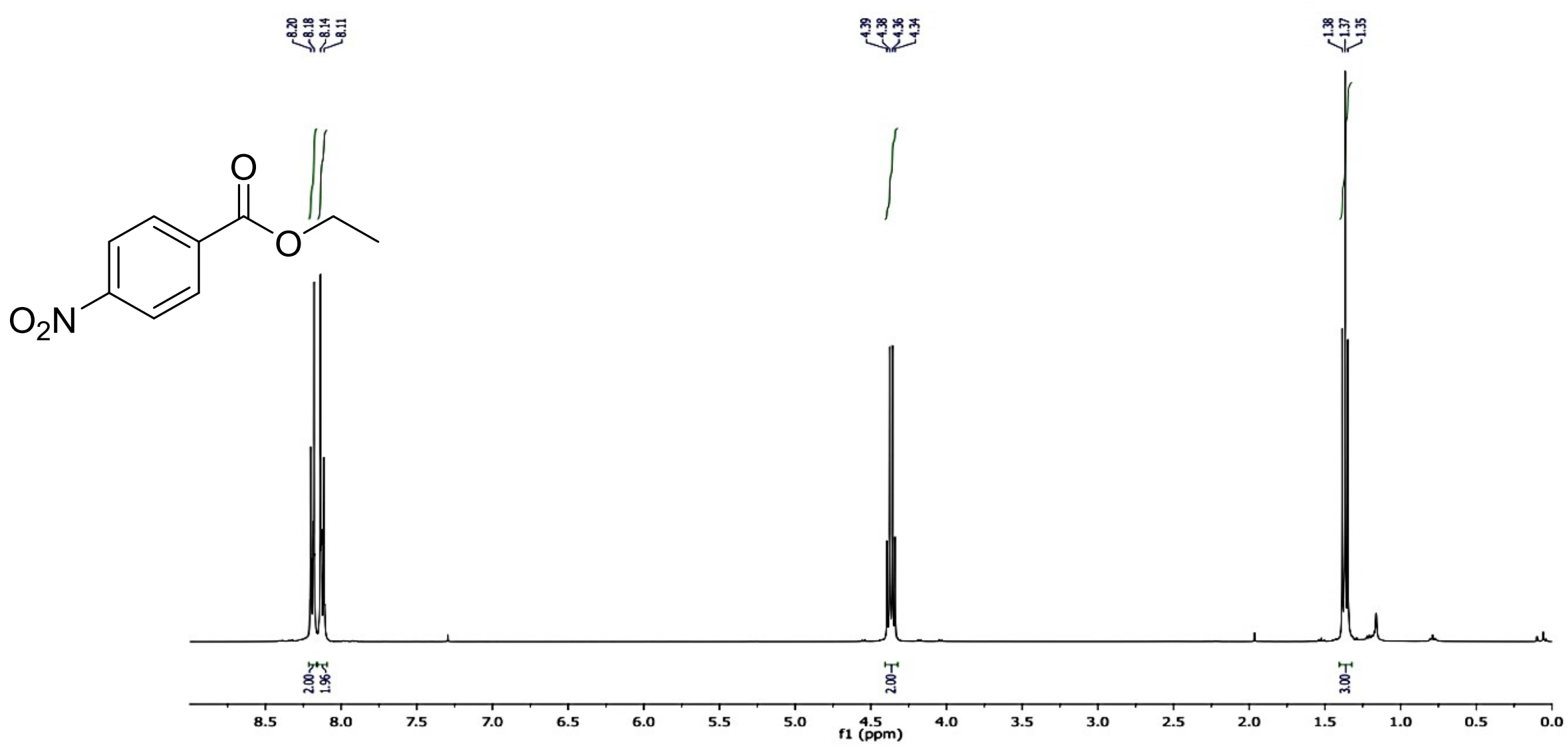

Figure S40. ^{1}H NMR spectrum of the ethyl 4-nitrobenzoate (**7b**) product in $CDCl_3$

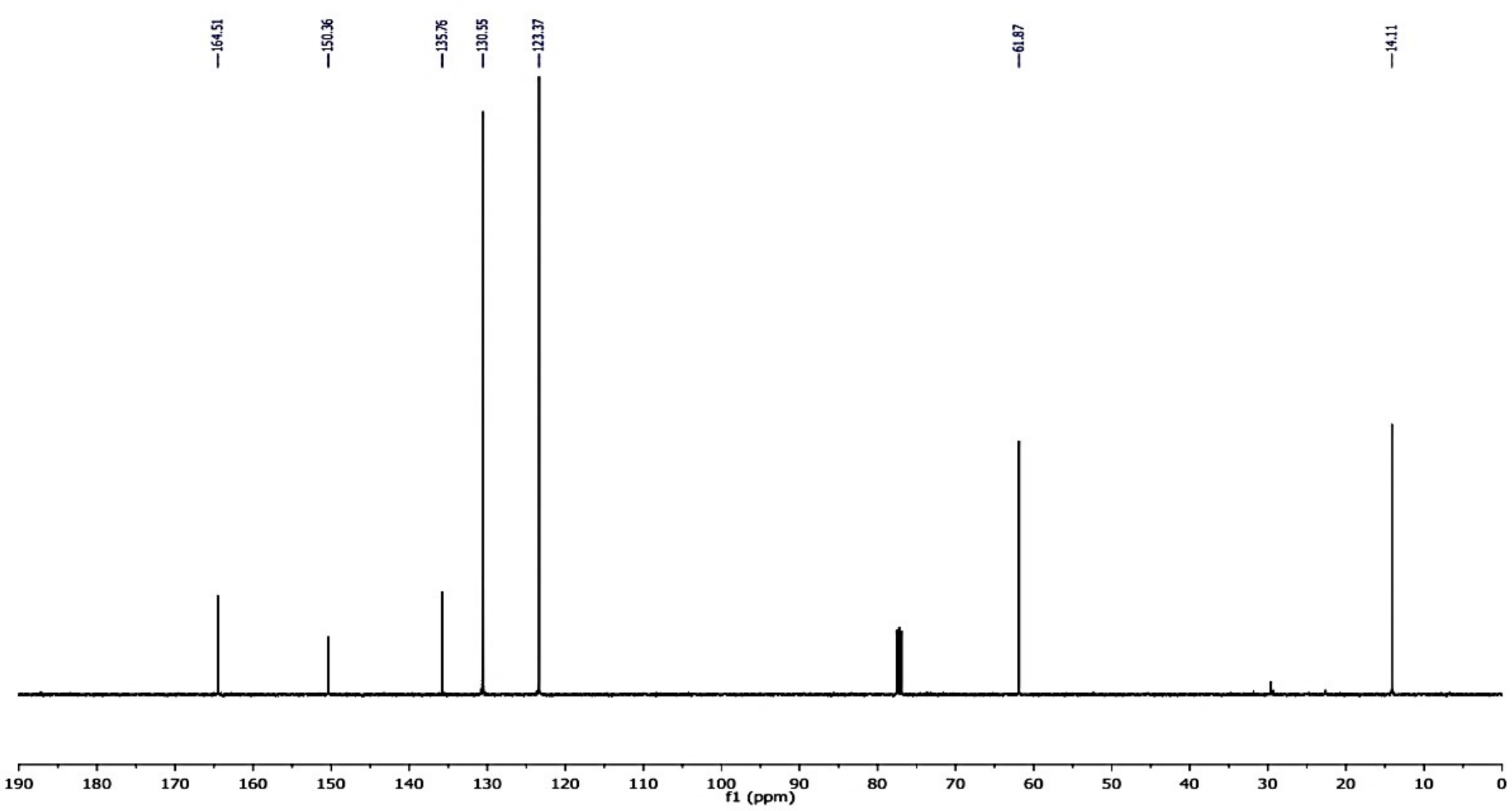

Figure S41. ^{13}C NMR spectrum of the ethyl 4-nitrobenzoate (**7b**) product in $CDCl_3$

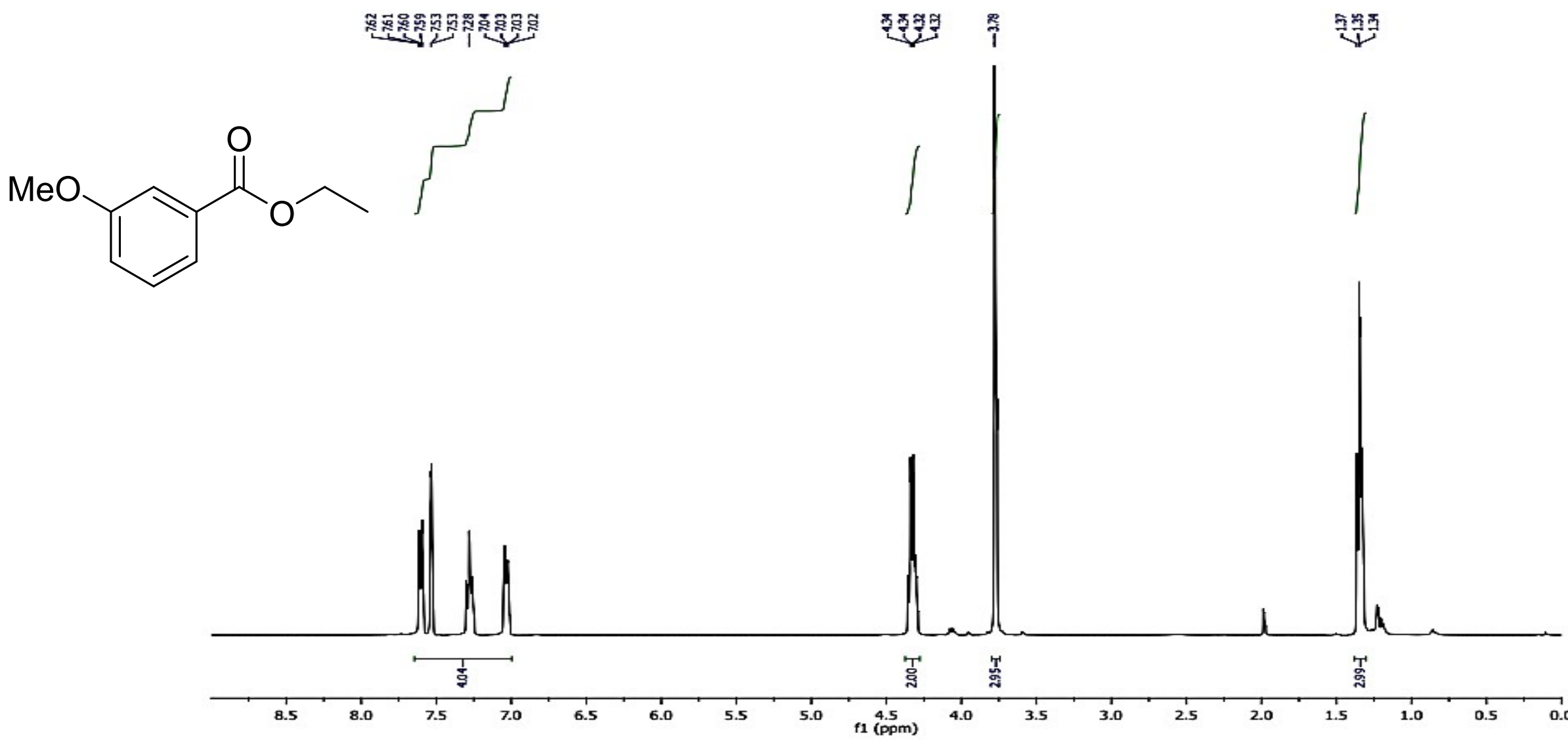

Figure S42. ^{1}H NMR spectrum of the ethyl 2-methoxybenzoate (**7c**) product in $CDCl_3$

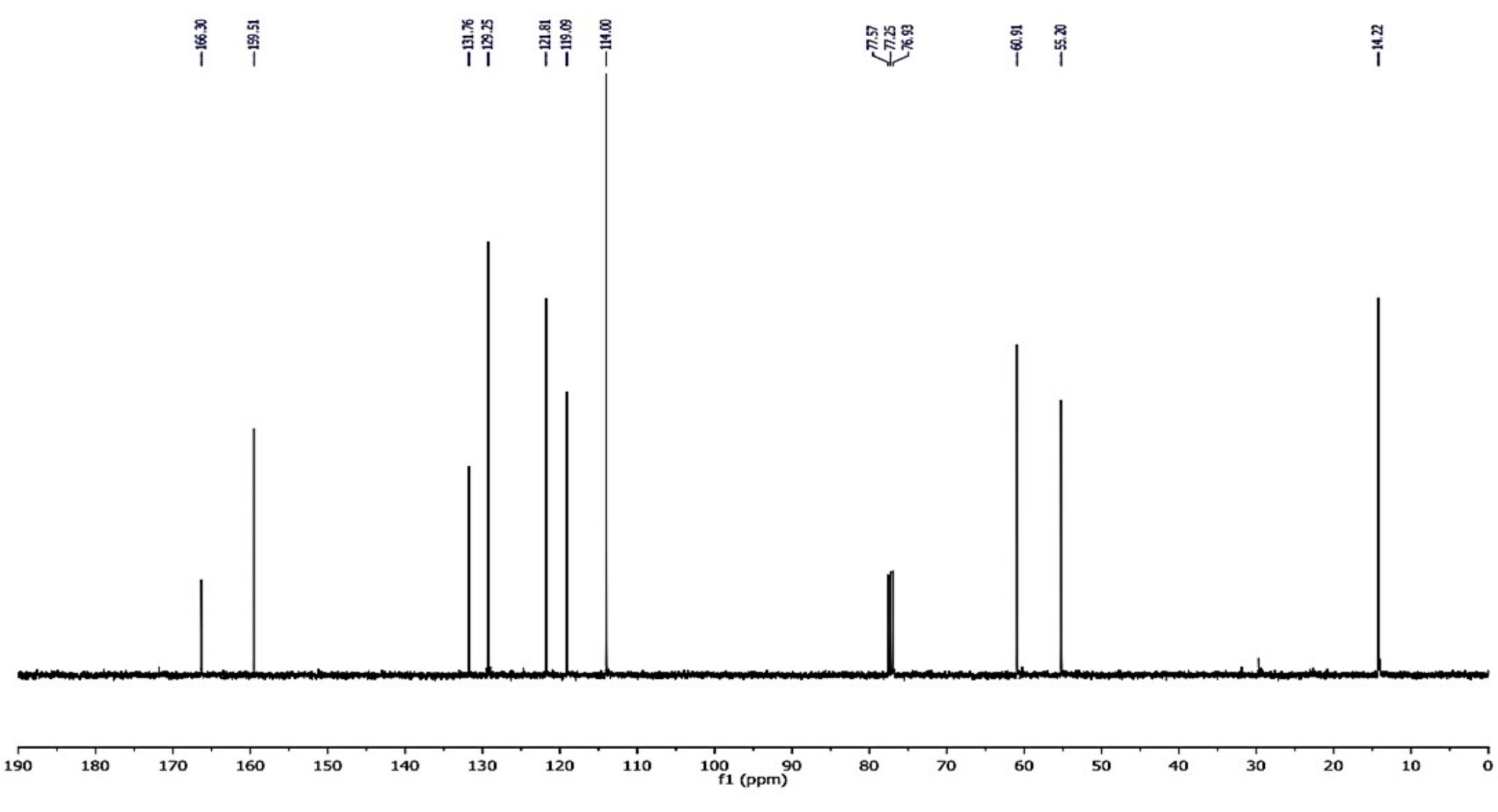

Figure S43. ^{13}C NMR spectrum of the ethyl 2-methoxybenzoate (**7c**) product in $CDCl_3$

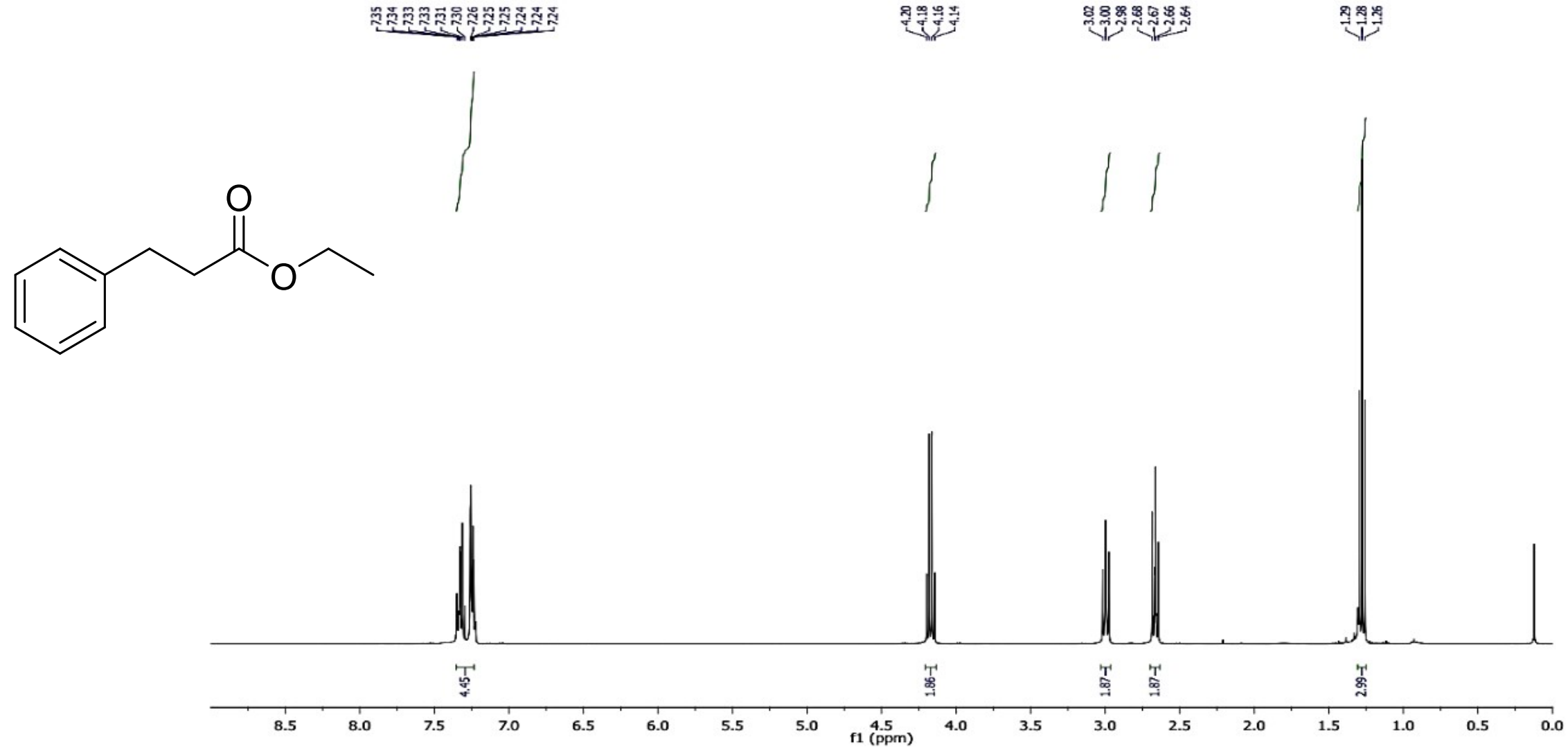

Figure S44. ^{1}H NMR spectrum of the ethyl 3-phenylpropanoate (**7d**) product in $CDCl_3$

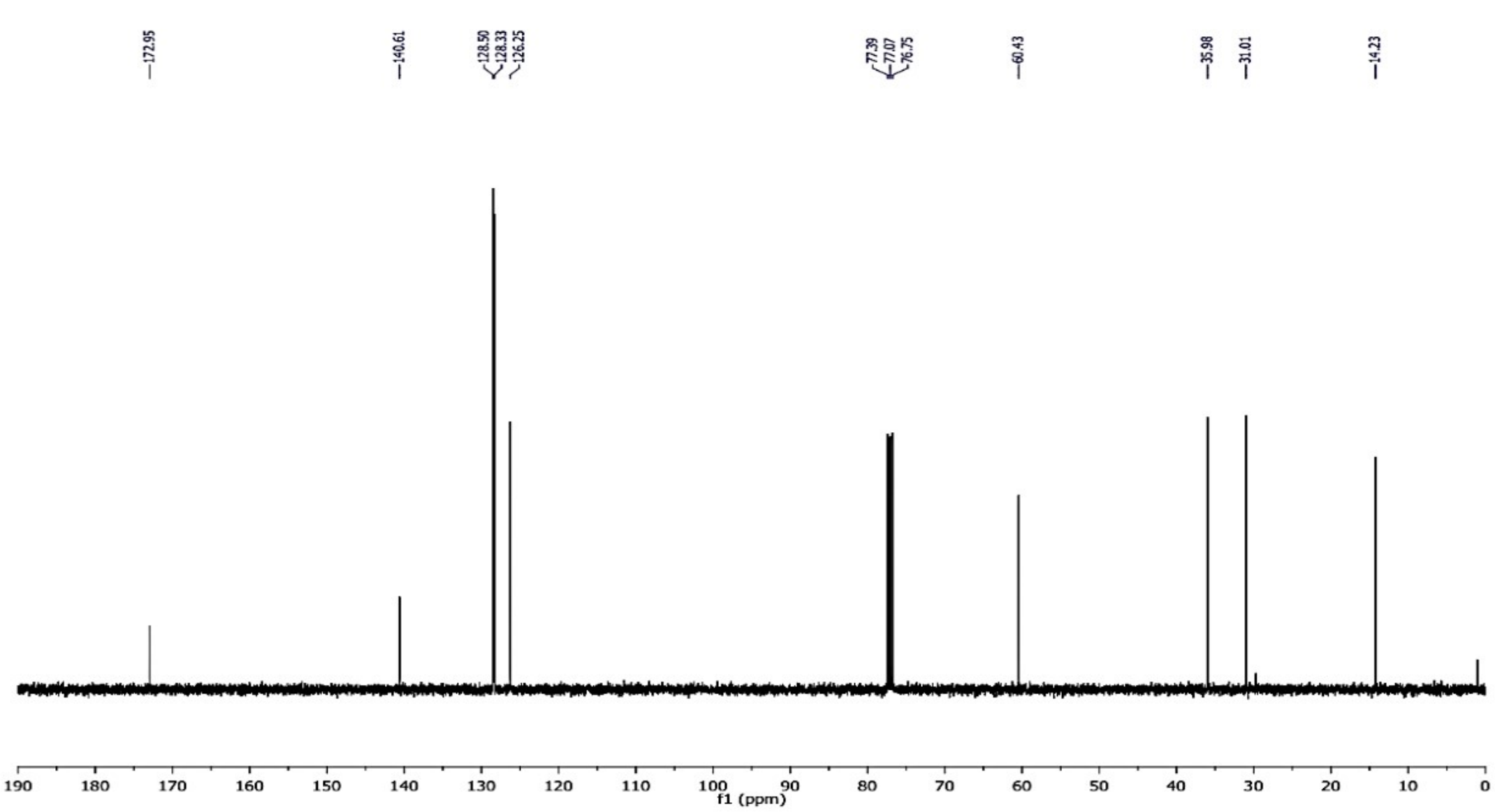

Figure S45. ^{13}C NMR spectrum of the ethyl 3-phenylpropanoate (**7d**) product in $CDCl_3$

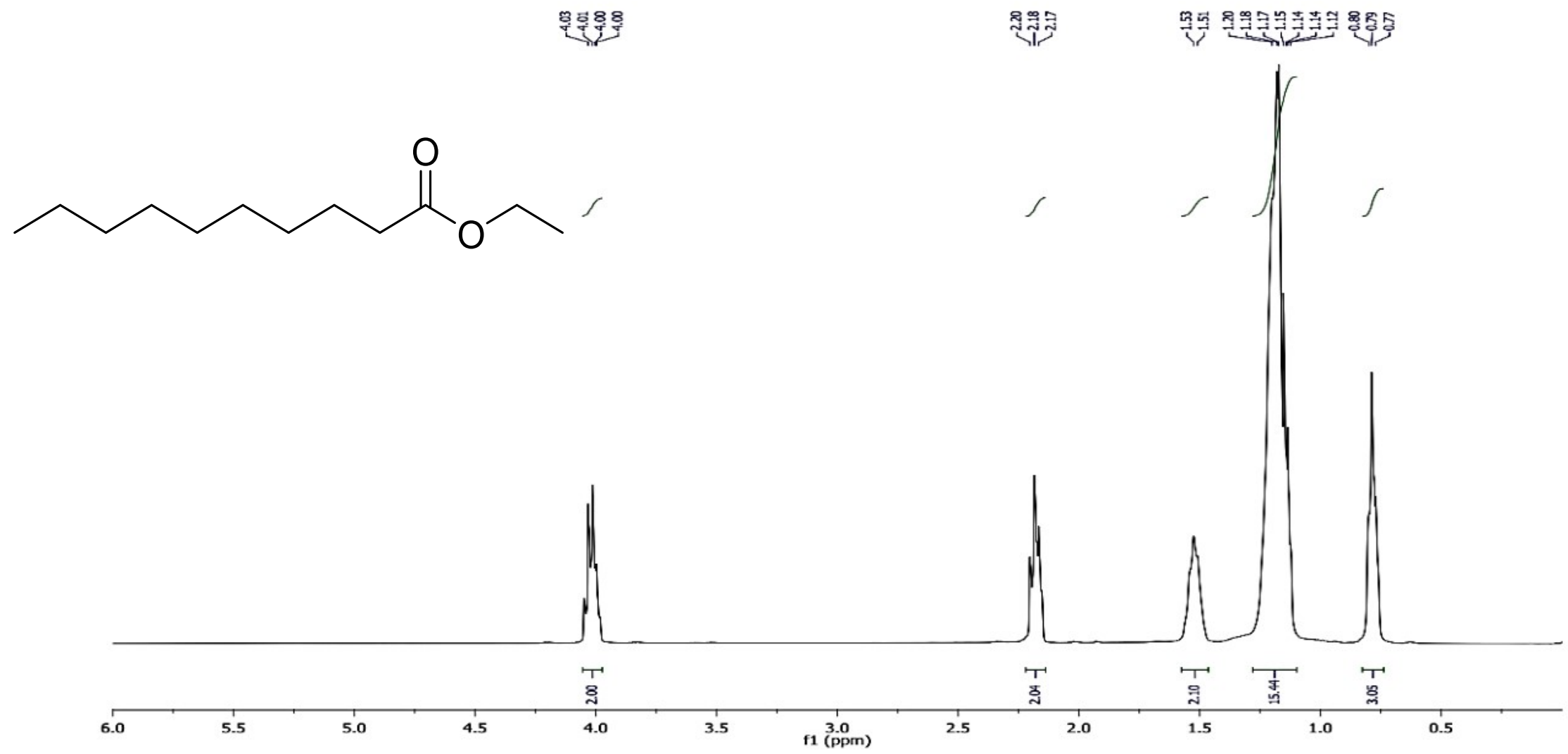

Figure S46. ^{1}H NMR spectrum of the ethyl decanoate (**7e**) product in $CDCl_3$

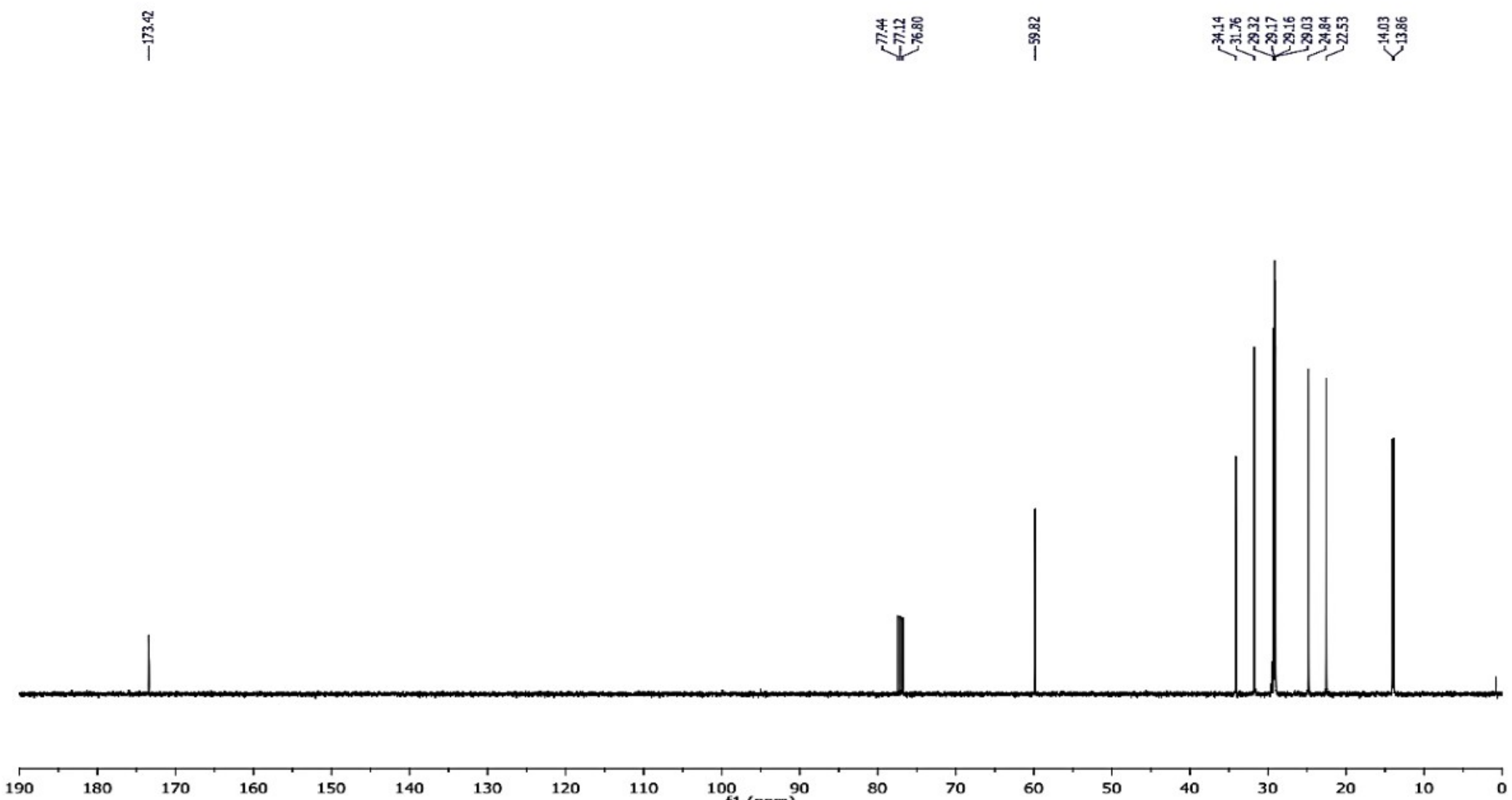

Figure S47. ^{13}C NMR spectrum of the ethyl decanoate (**7e**) product in $CDCl_3$

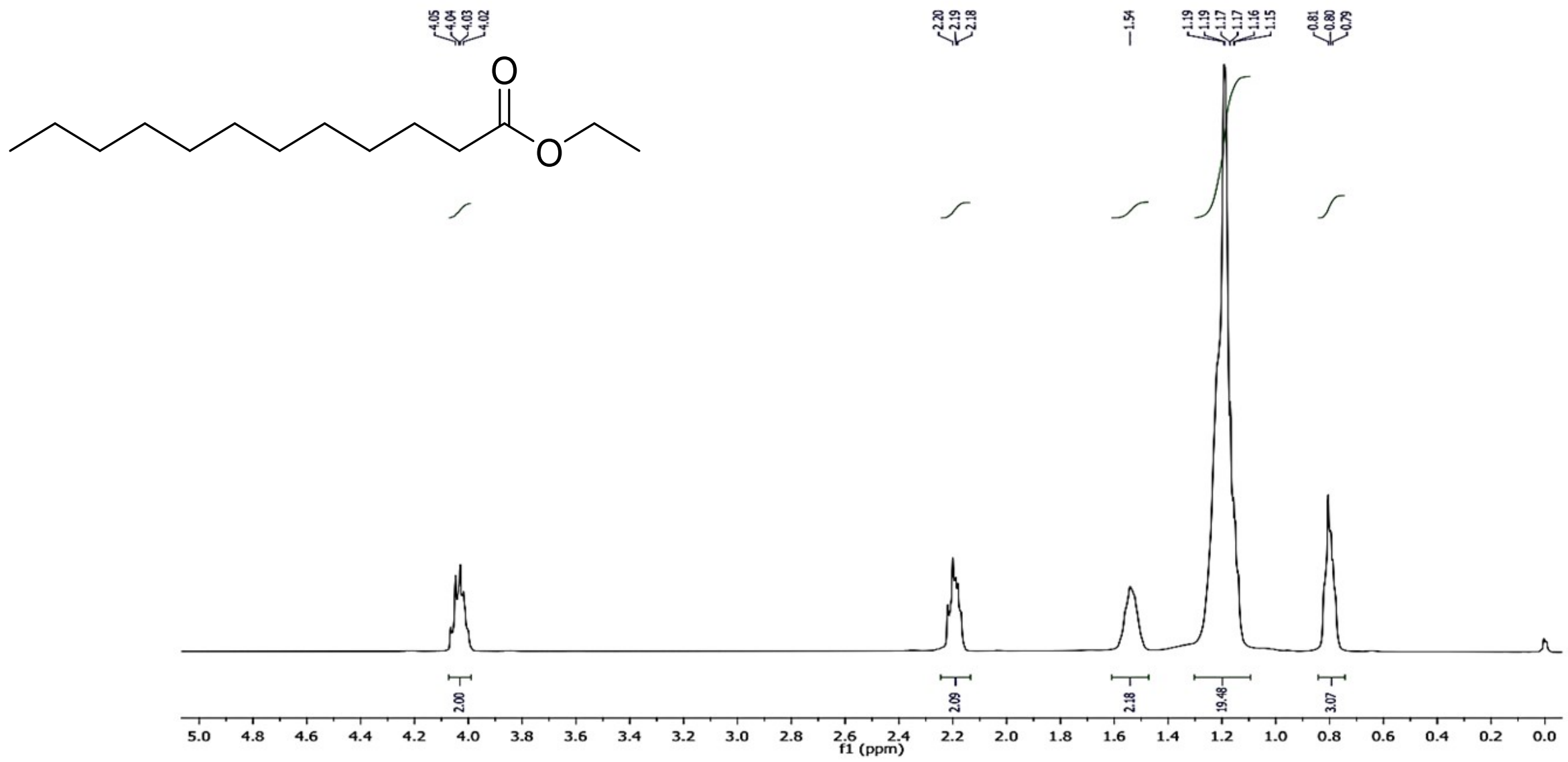

Figure S48. ^{1}H NMR spectrum of the ethyl dodecanoate (**7f**) product in $CDCl_3$

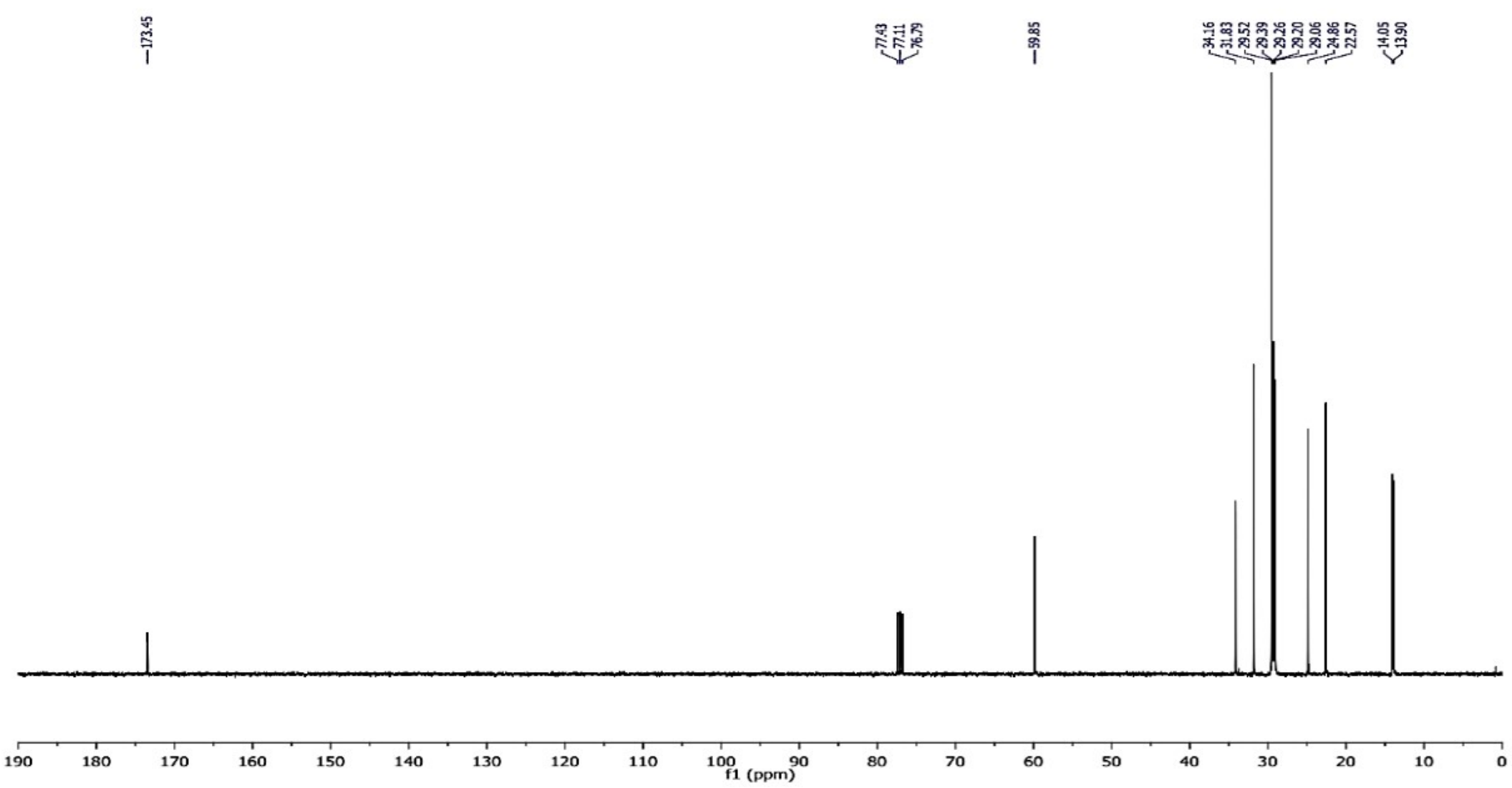

Figure S49. ^{13}C NMR spectrum of the ethyl dodecanoate (**7f**) product in $CDCl_3$

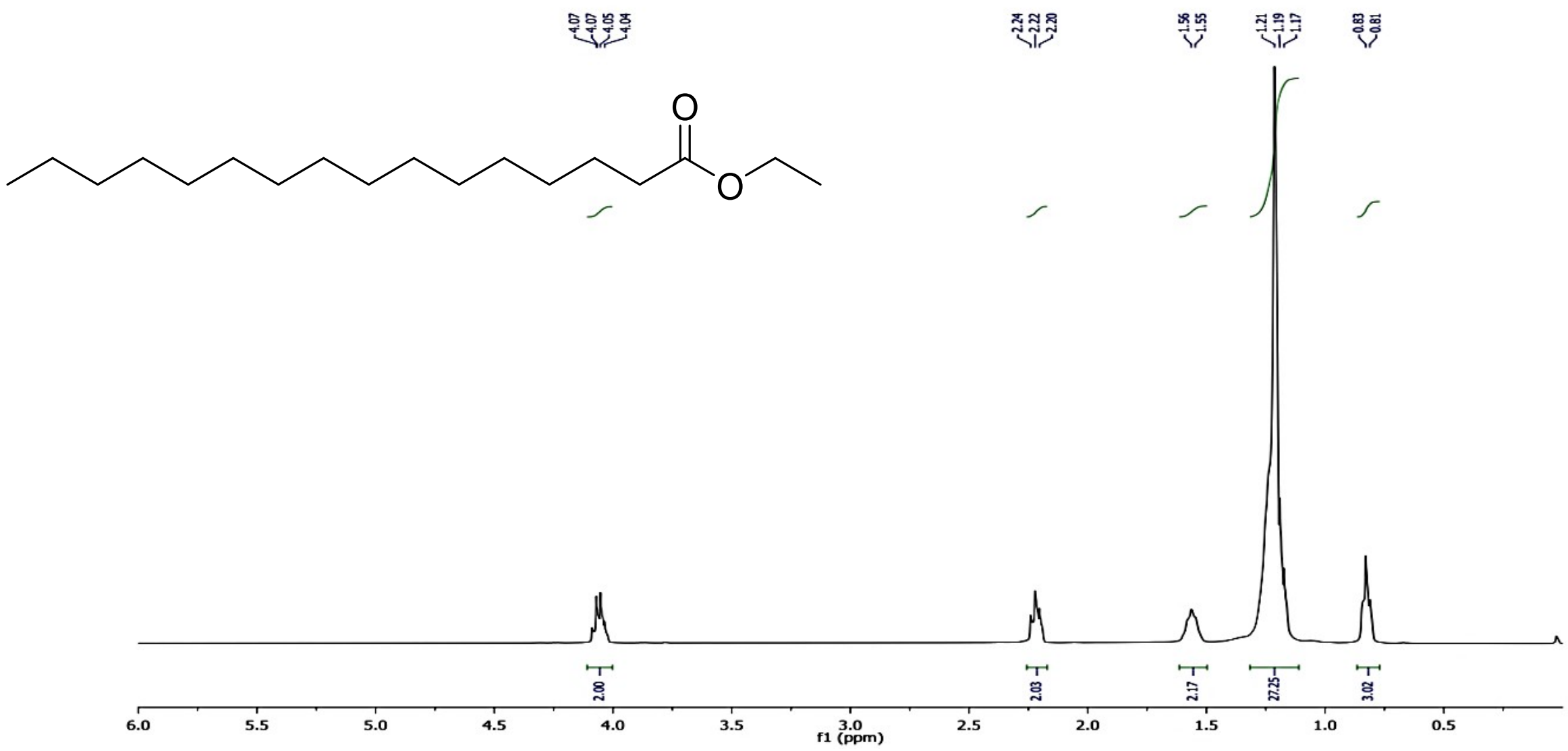

Figure S50. ^{1}H NMR spectrum of the ethyl hexadecanoate (**7g**) product in $CDCl_3$

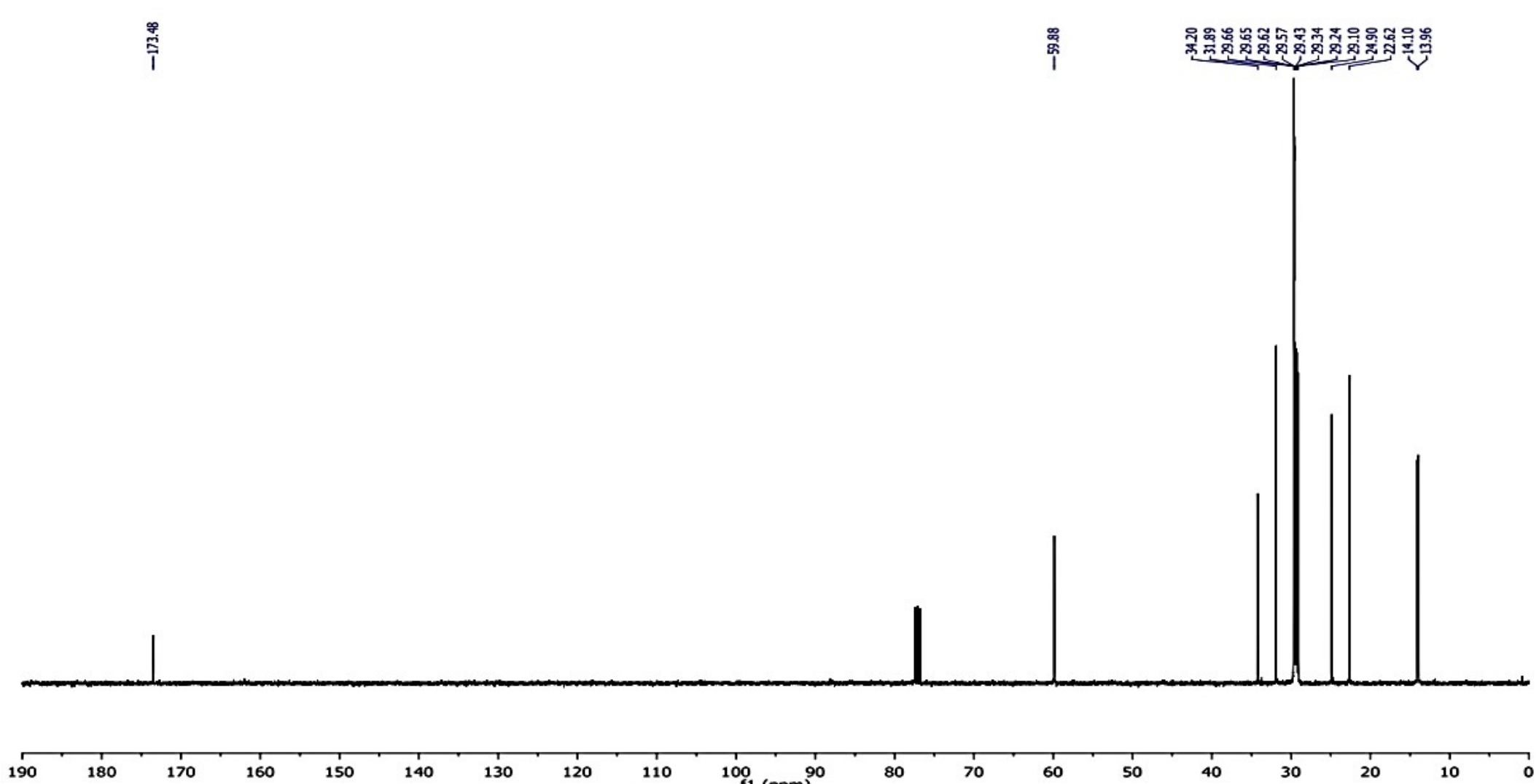

Figure S51. ^{13}C NMR spectrum of the ethyl hexadecanoate (**7g**) product in $CDCl_3$

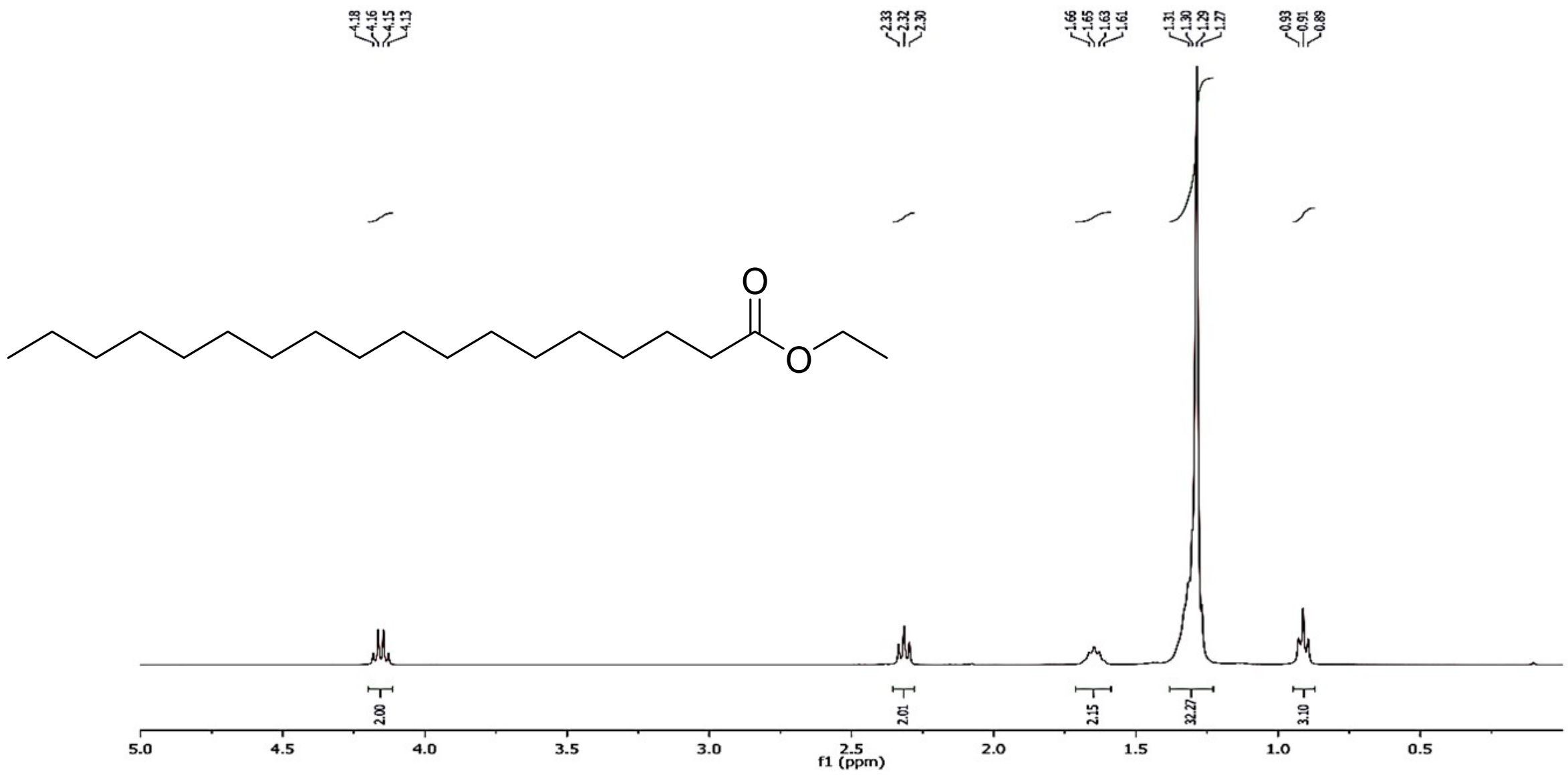

Figure S52. ^{1}H NMR spectrum of the ethyl octadecanoate (**7h**) product in $CDCl_3$

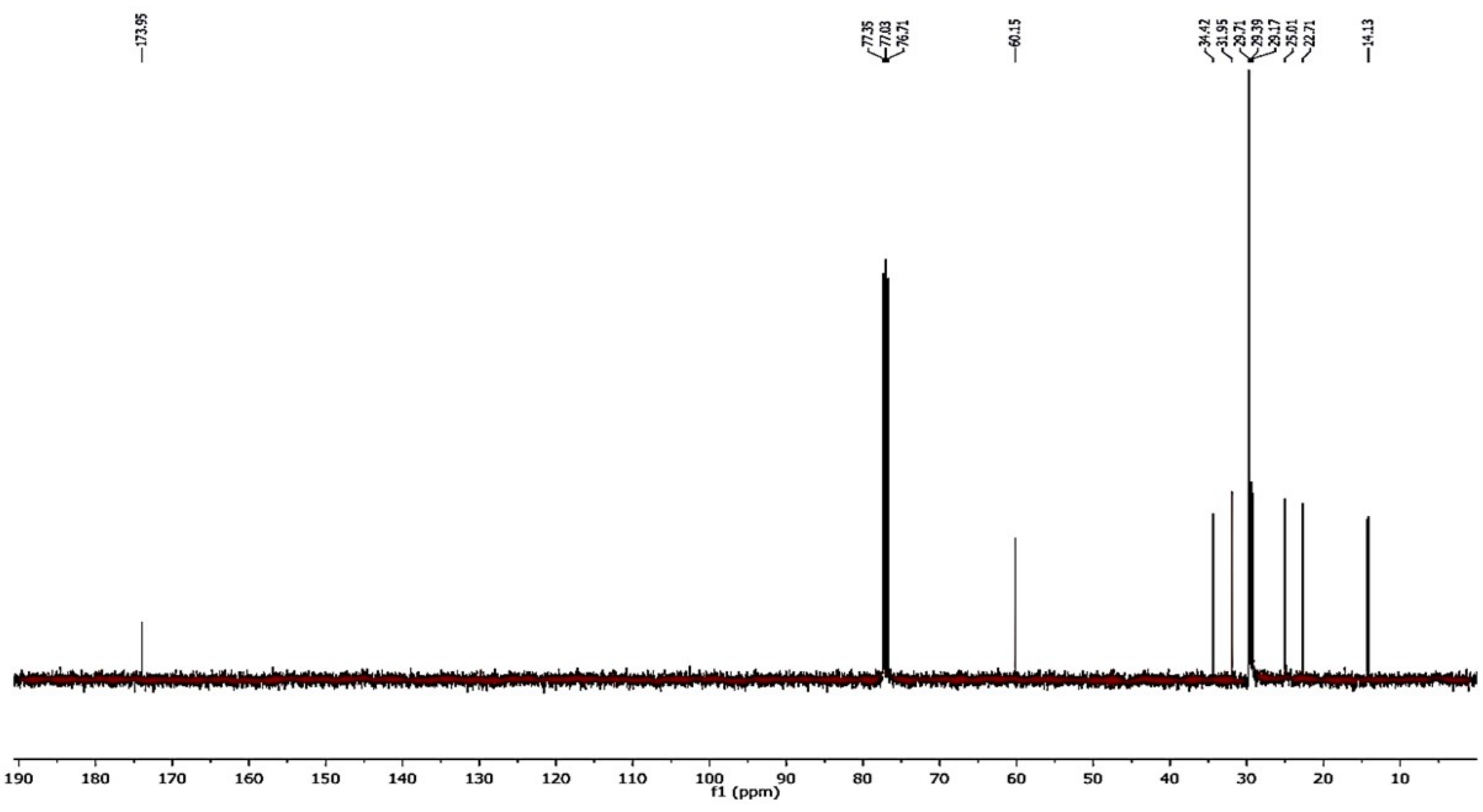

Figure S53. ^{13}C NMR spectrum of the ethyl octadecanoate (**7h**) product in $CDCl_3$

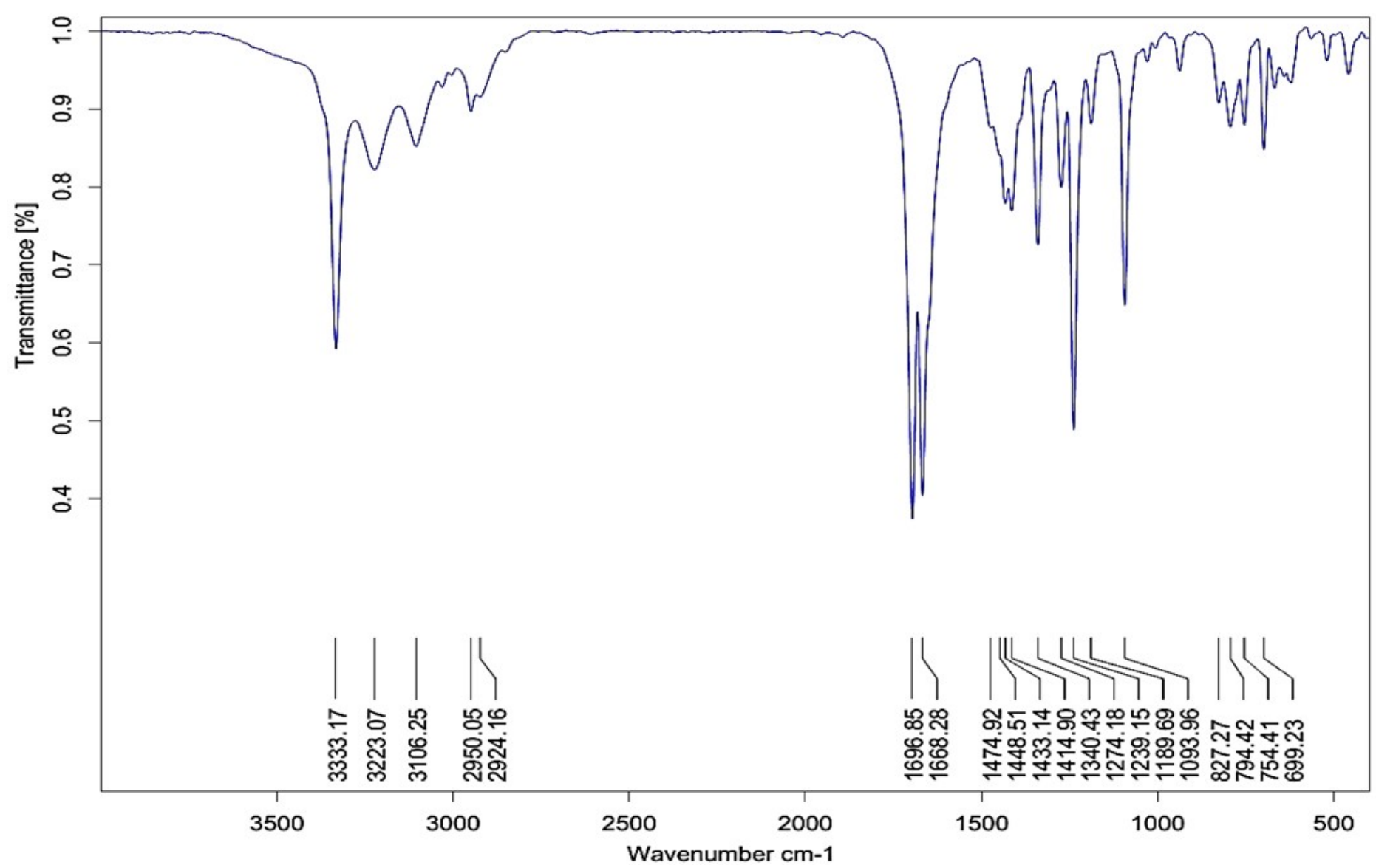

Figure S54. FTIR spectrum of the methyl 6-methyl-2-oxo-4-phenyl-1,2,3,4-tetrahydropyrimidine-5-carboxylate (**9a**) product

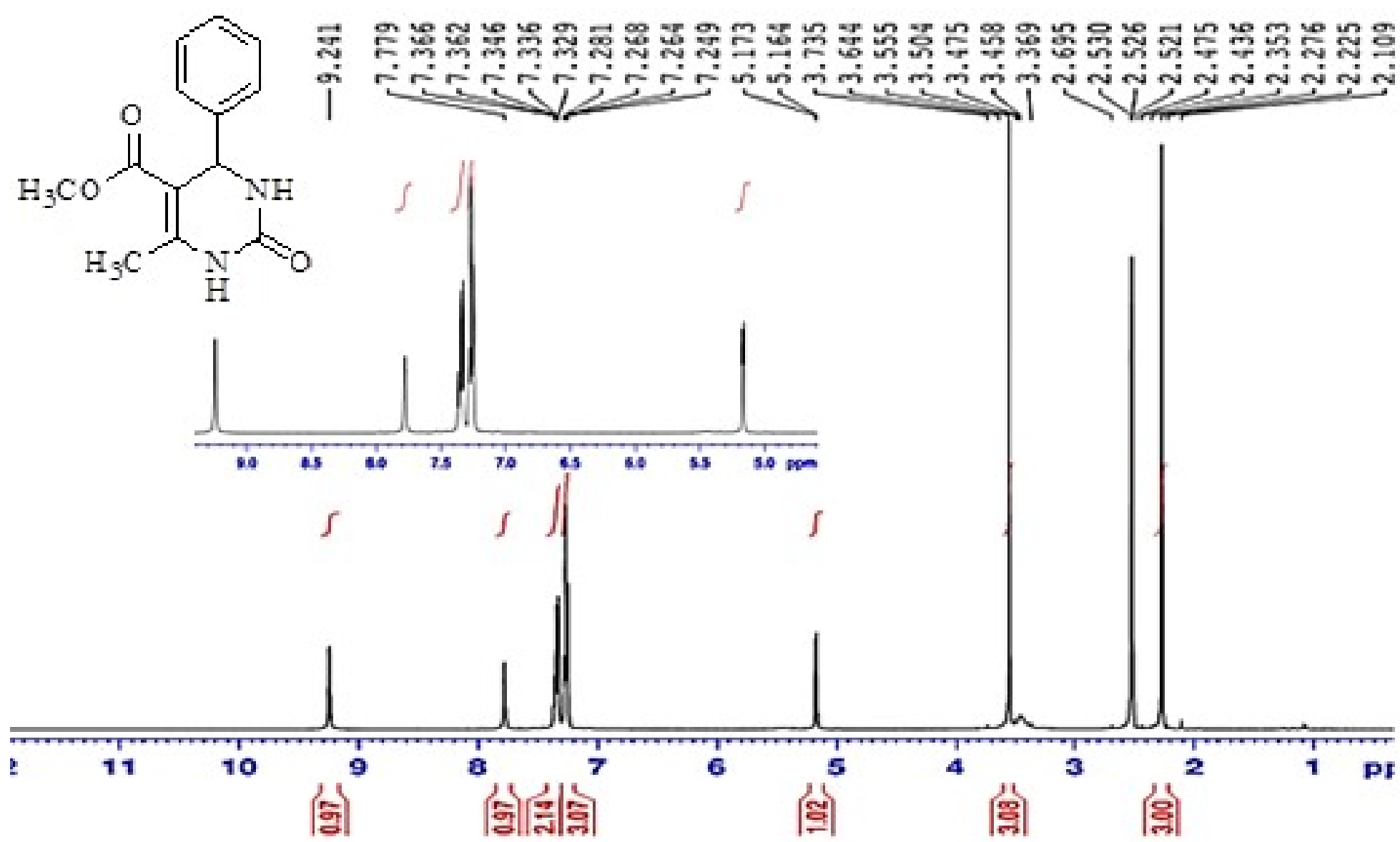

Figure S55. ^{1}H NMR spectrum of the methyl 6-methyl-2-oxo-4-phenyl-1,2,3,4-tetrahydropyrimidine-5-carboxylate (**9a**) product in DMSO

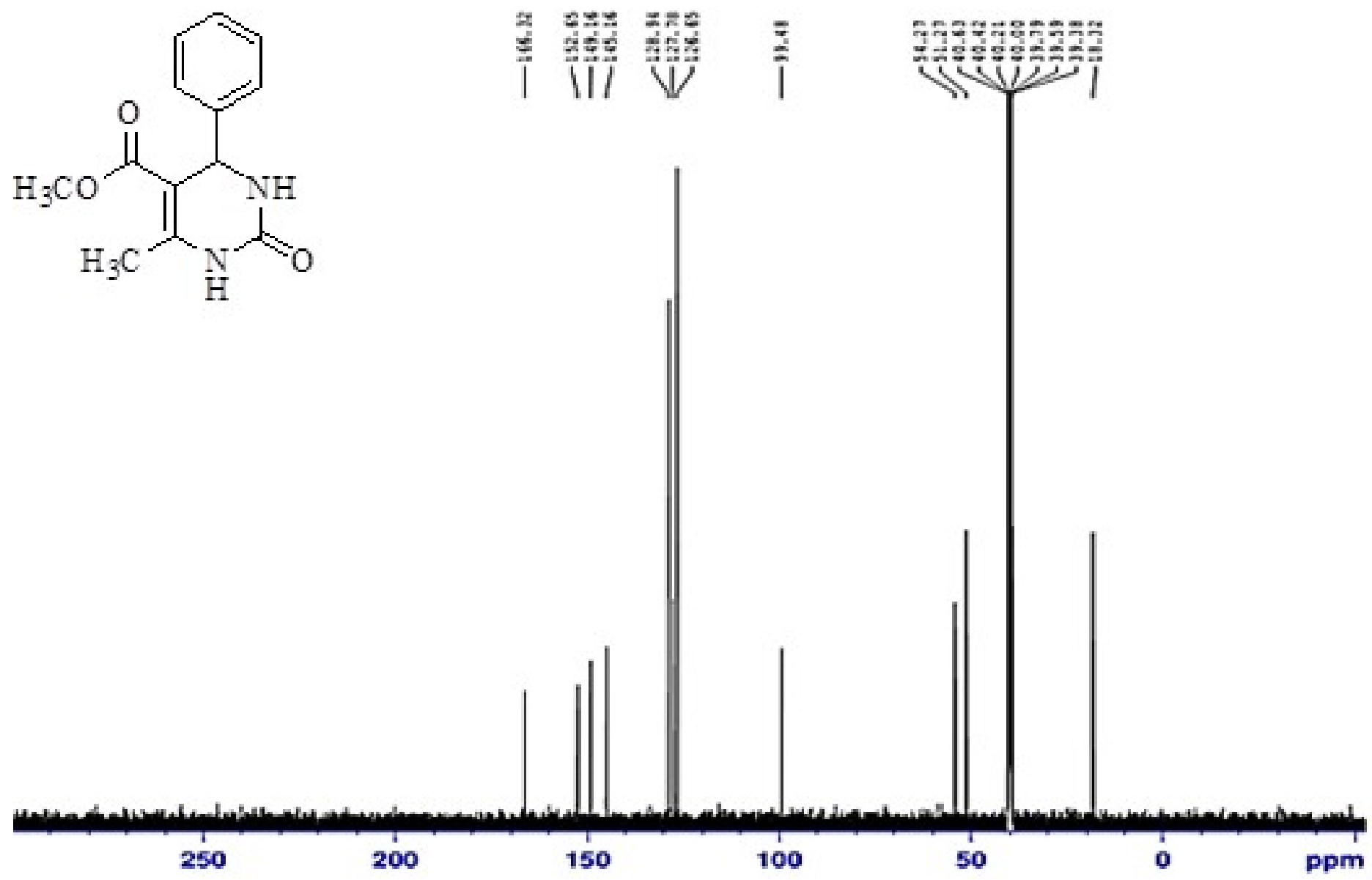

Figure S56. ^{13}C NMR spectrum of the methyl 6-methyl-2-oxo-4-phenyl-1,2,3,4-tetrahydropyrimidine-5-carboxylate (**9a**) product in DMSO

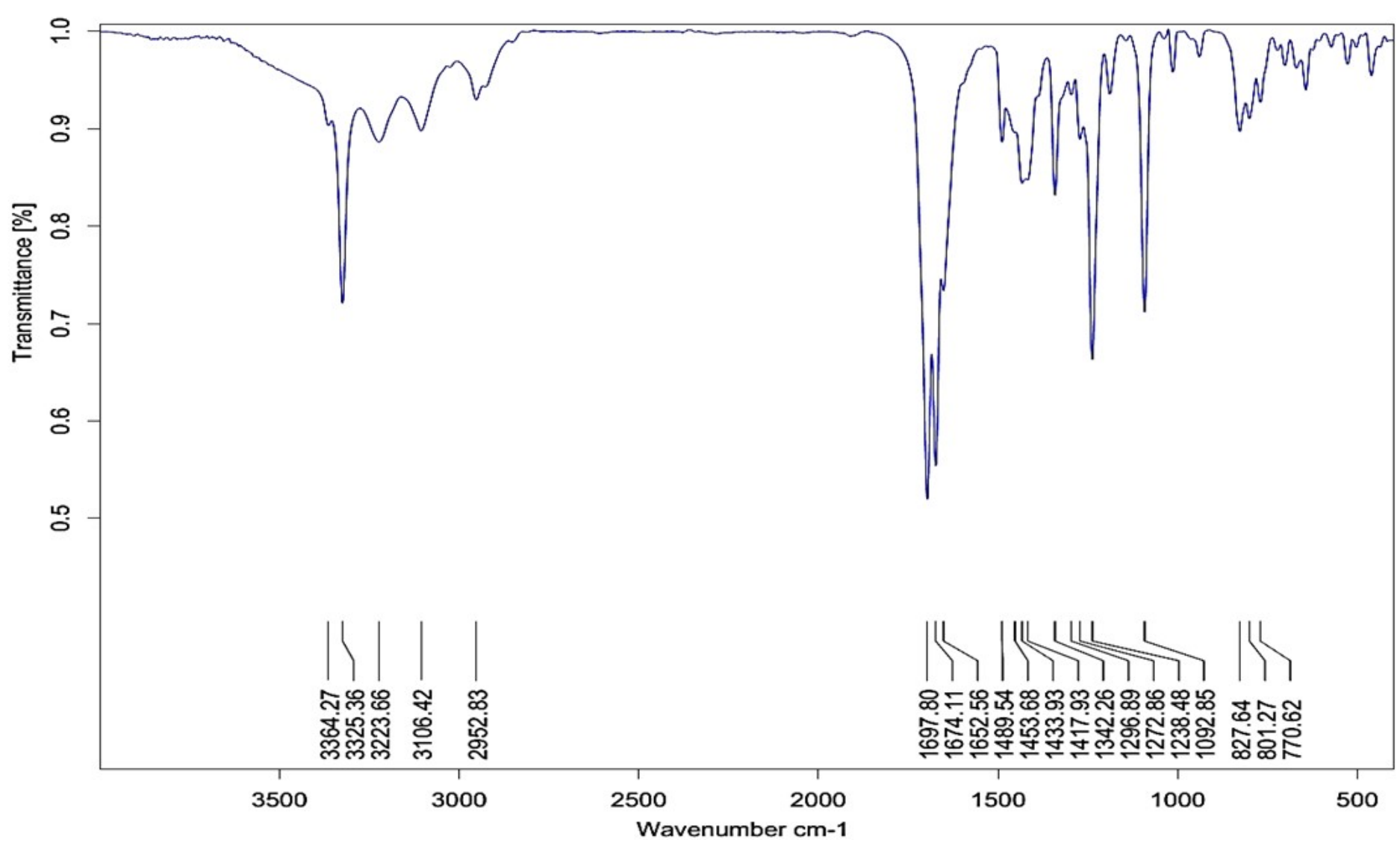

Figure S57. FTIR spectrum of the 4-(4-chlorophenyl)-6-methyl-2-oxo-1,2,3,4-tetrahydropyrimidine -5-carboxylate (**9b**) product

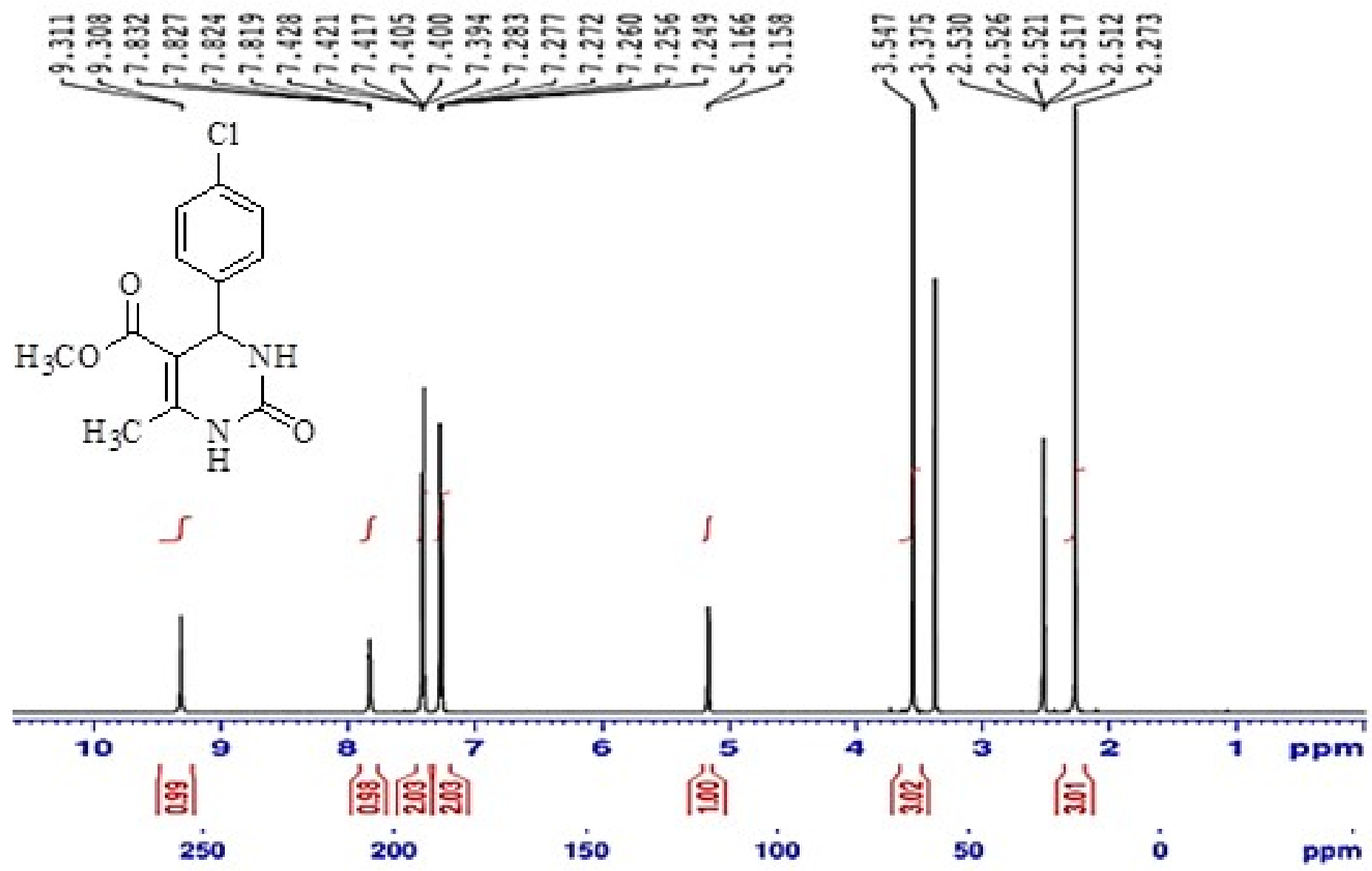

Figure S58. ^{1}H NMR spectrum of Methyl 4-(4-chlorophenyl)-6-methyl-2-oxo-1,2,3,4-tetrahydropyrimidine -5-carboxylate (**9b**) product in DMSO

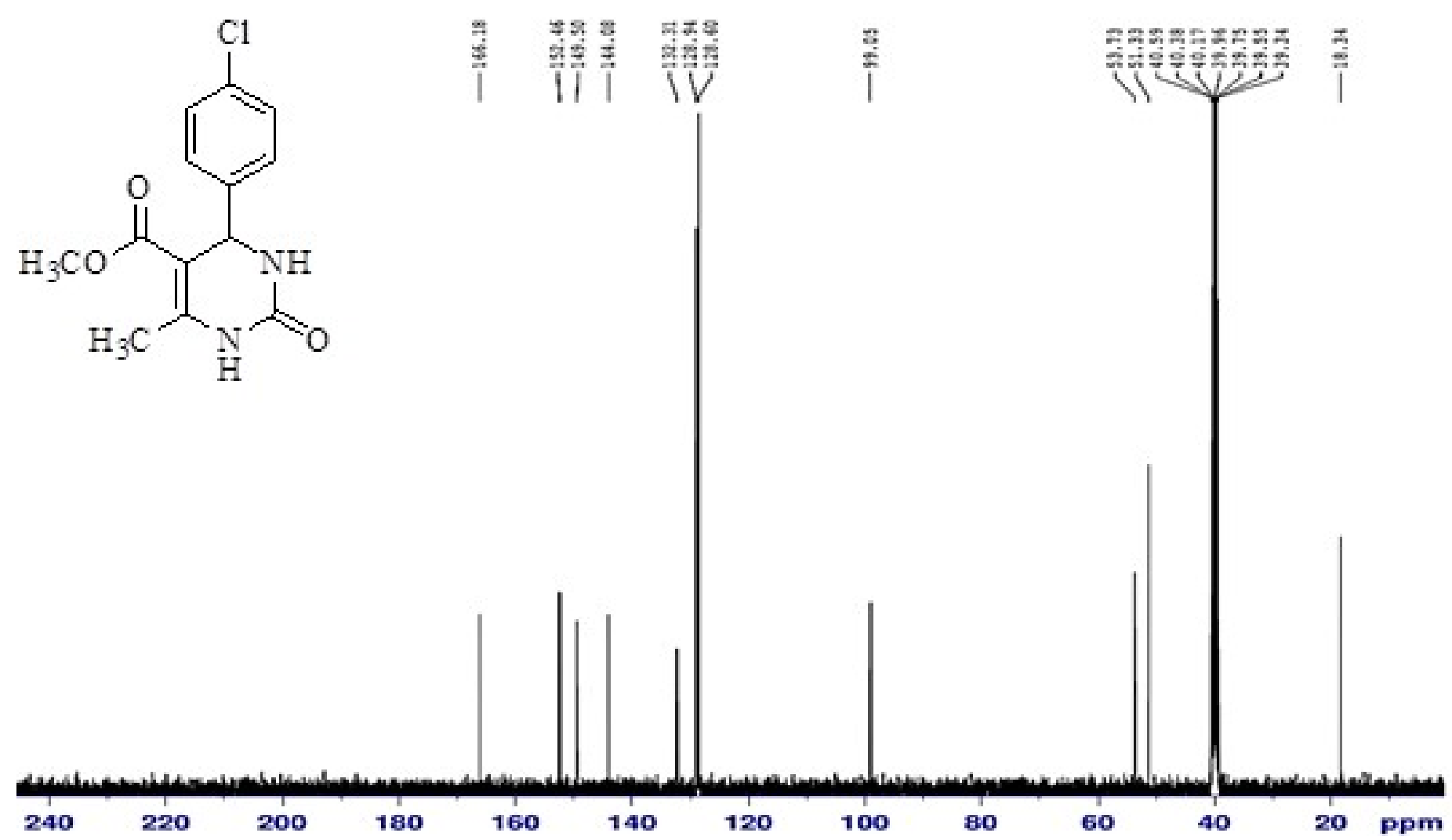

Figure S59. ^{13}C NMR spectrum of Methyl 4-(4-chlorophenyl)-6-methyl-2-oxo-1,2,3,4-tetrahydropyrimidine-5-carboxylate (**9b**) product in DMSO

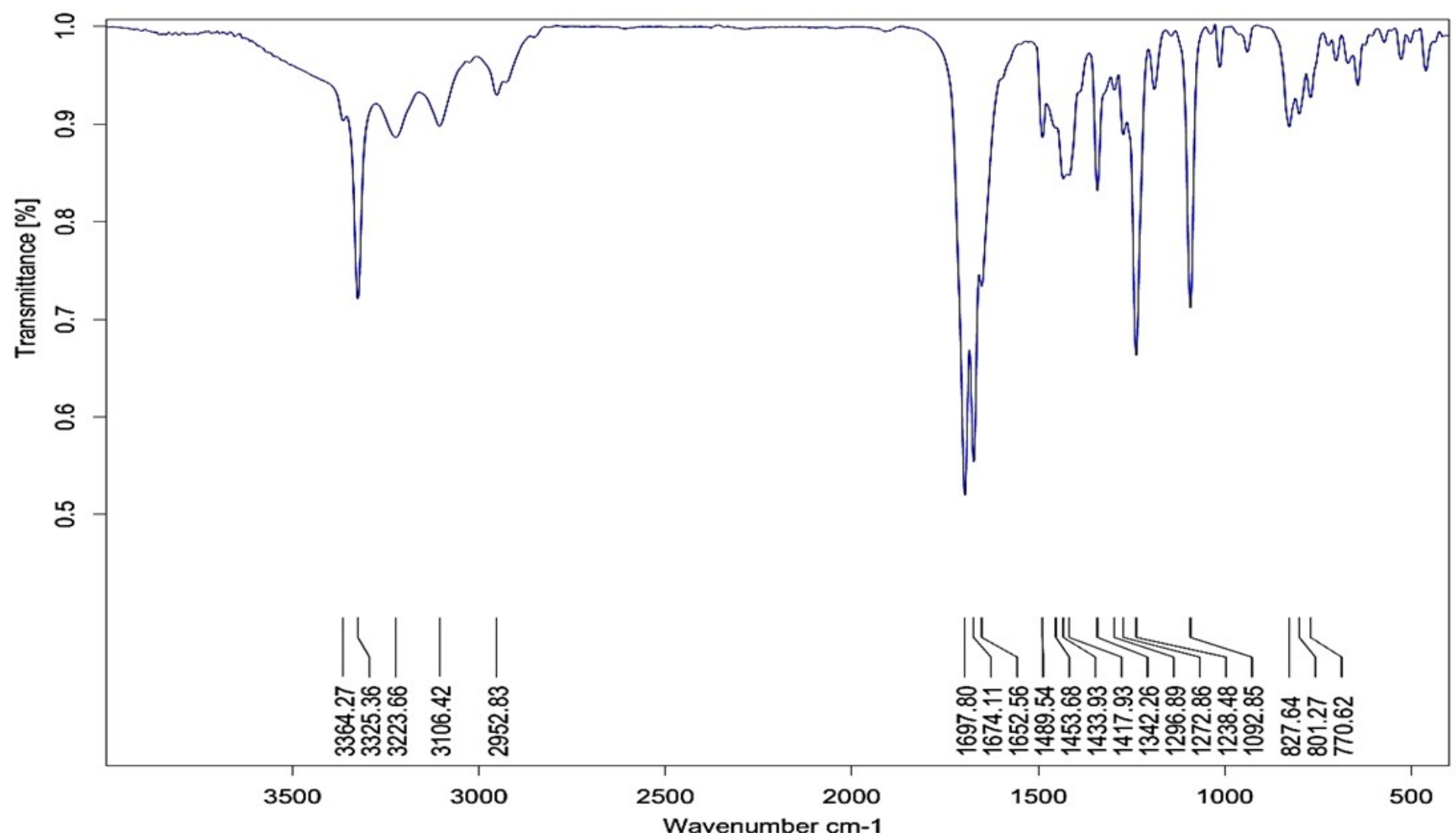

Figure S60. FTIR spectrum of the methyl 4-(4-boromophenyl)-6-methyl-2-oxo-1,2,3,4-tetrahydropyrimidine -5-carboxylate (**9c**) product

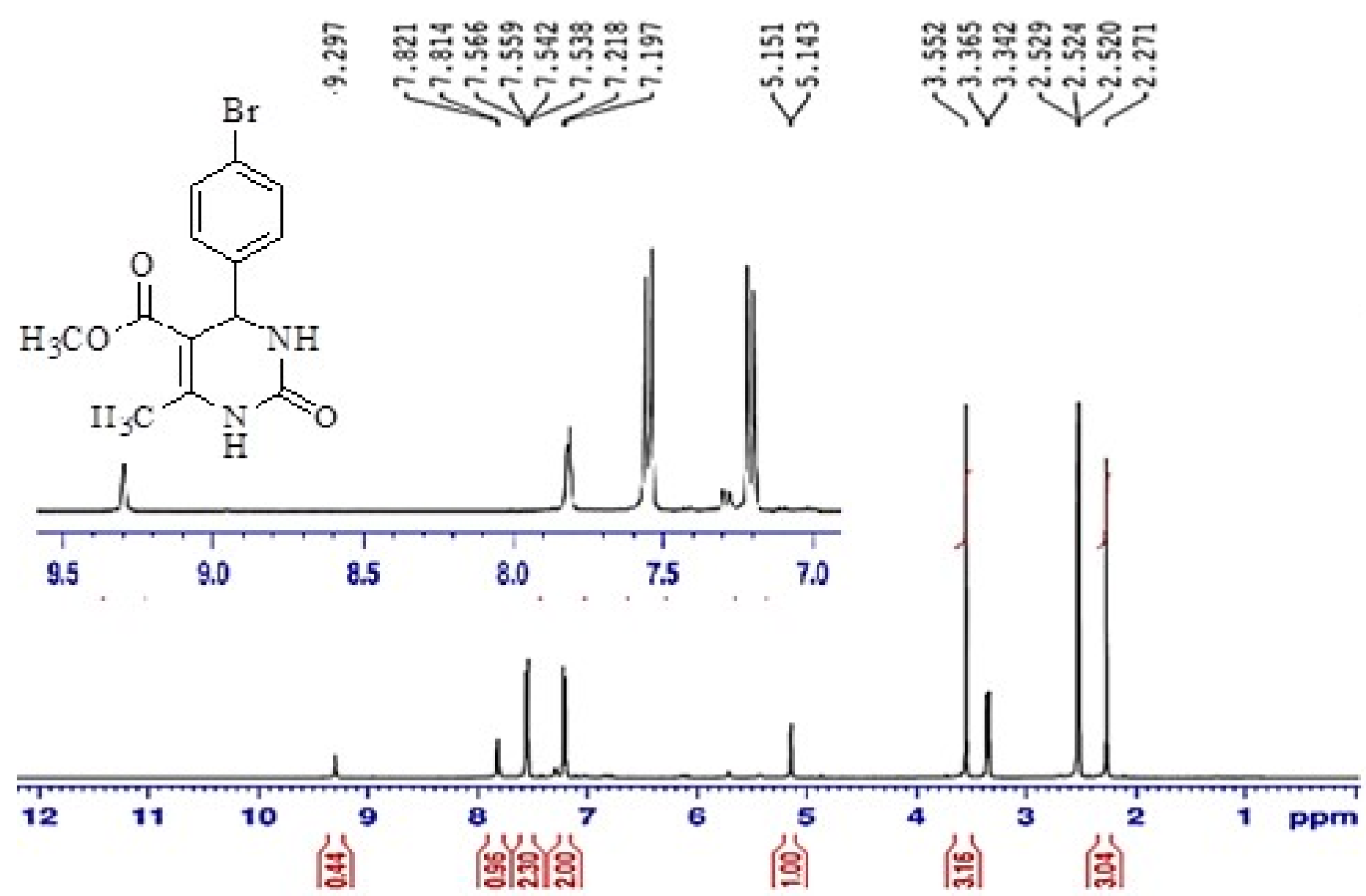

Figure S61. ^{1}H NMR spectrum of the methyl 4-(4-boromophenyl)-6-methyl-2-oxo-1,2,3,4-tetrahydropyrimidine -5-carboxylate (**9c**) product in DMSO

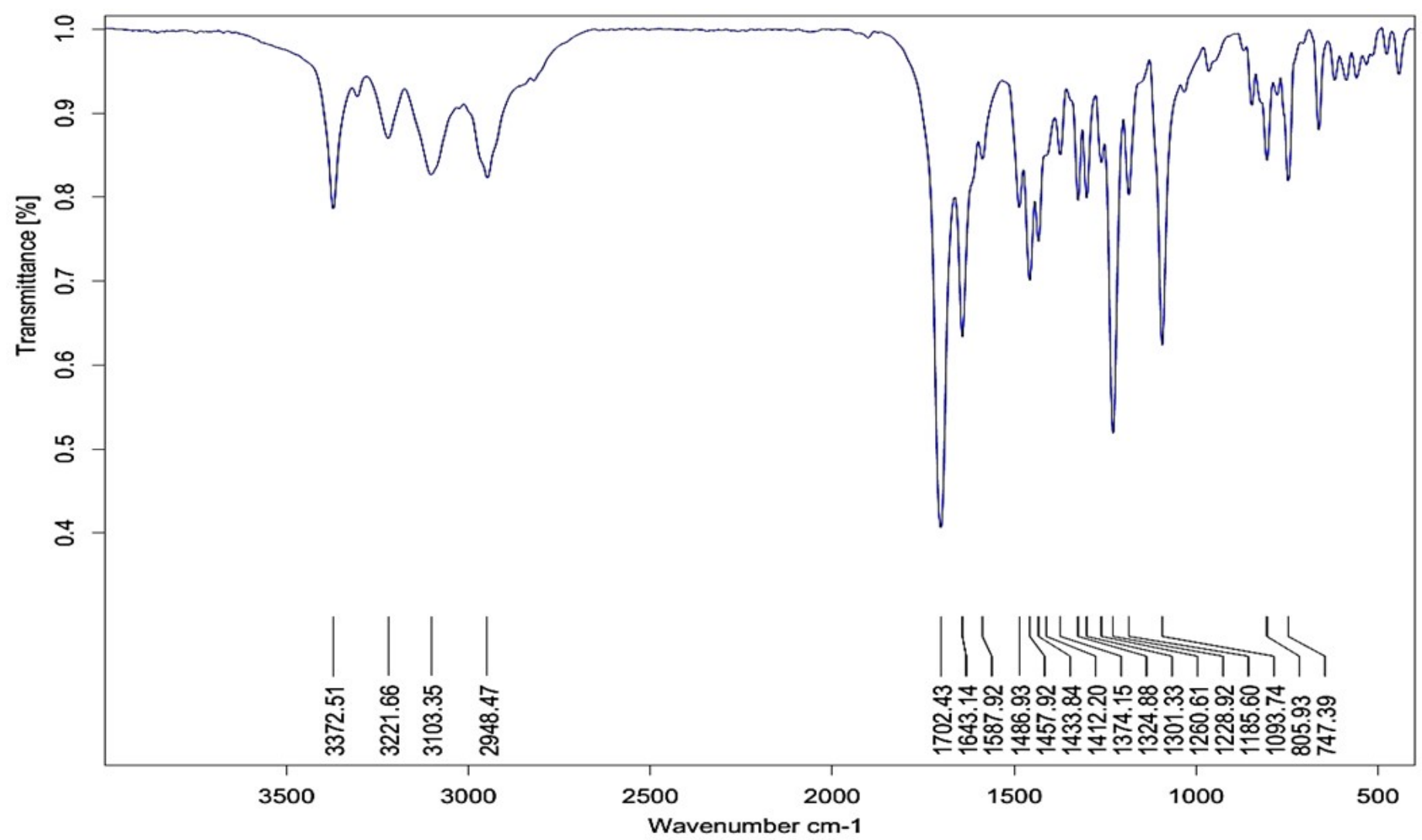

Figure S62. FTIR spectrum of the methyl 4-(2-fluorophenyl)-6-methyl-2-oxo-1,2,3,4 tetrahydropyrimidine-5-carboxylate (**9d**) product.

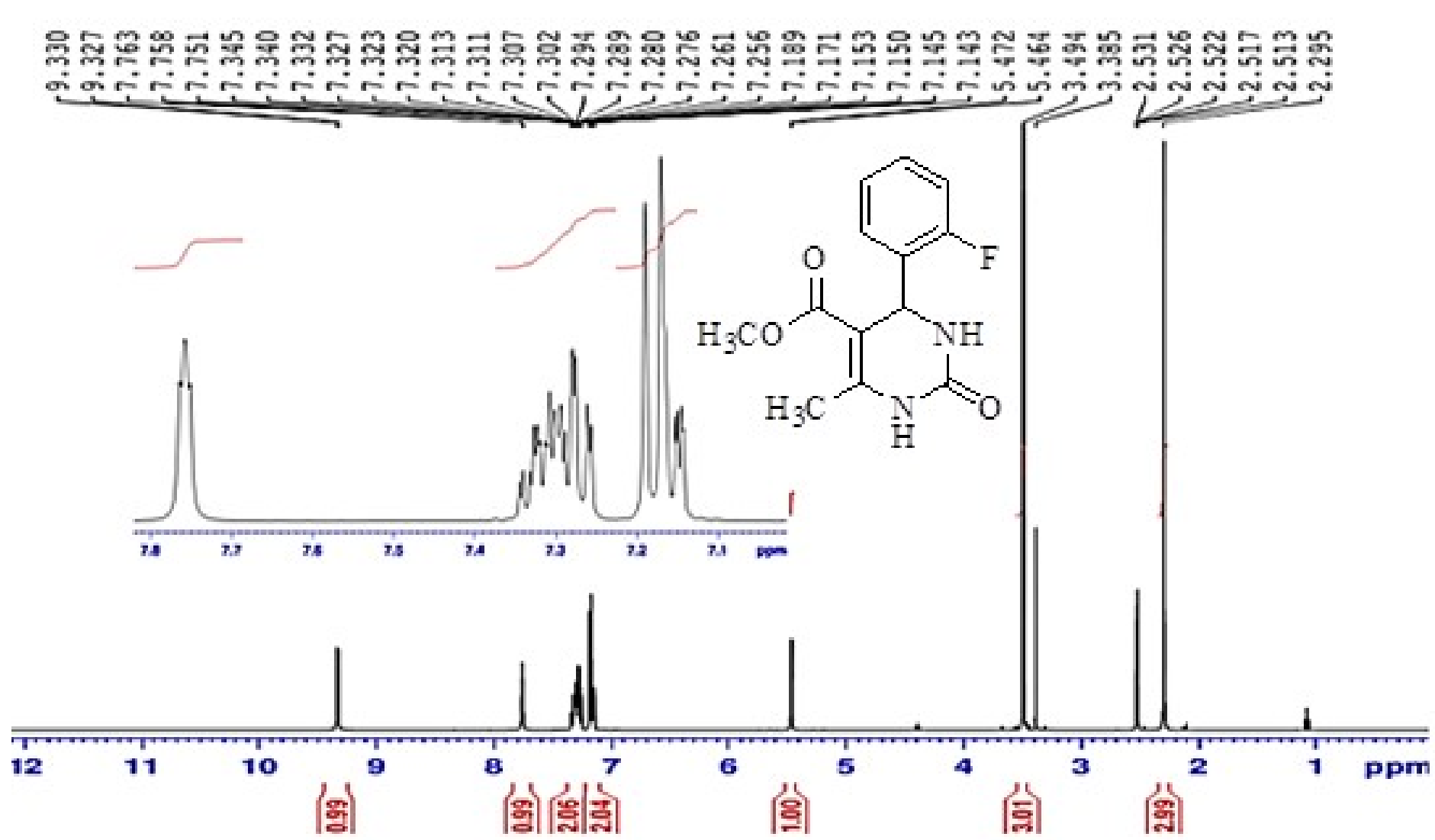

Figure S63. ^{1}H NMR spectrum of the methyl 4-(2-fluorophenyl)-6-methyl-2-oxo-1,2,3,4 tetrahydropyrimidine-5-carboxylate (**9d**) product in DMSO

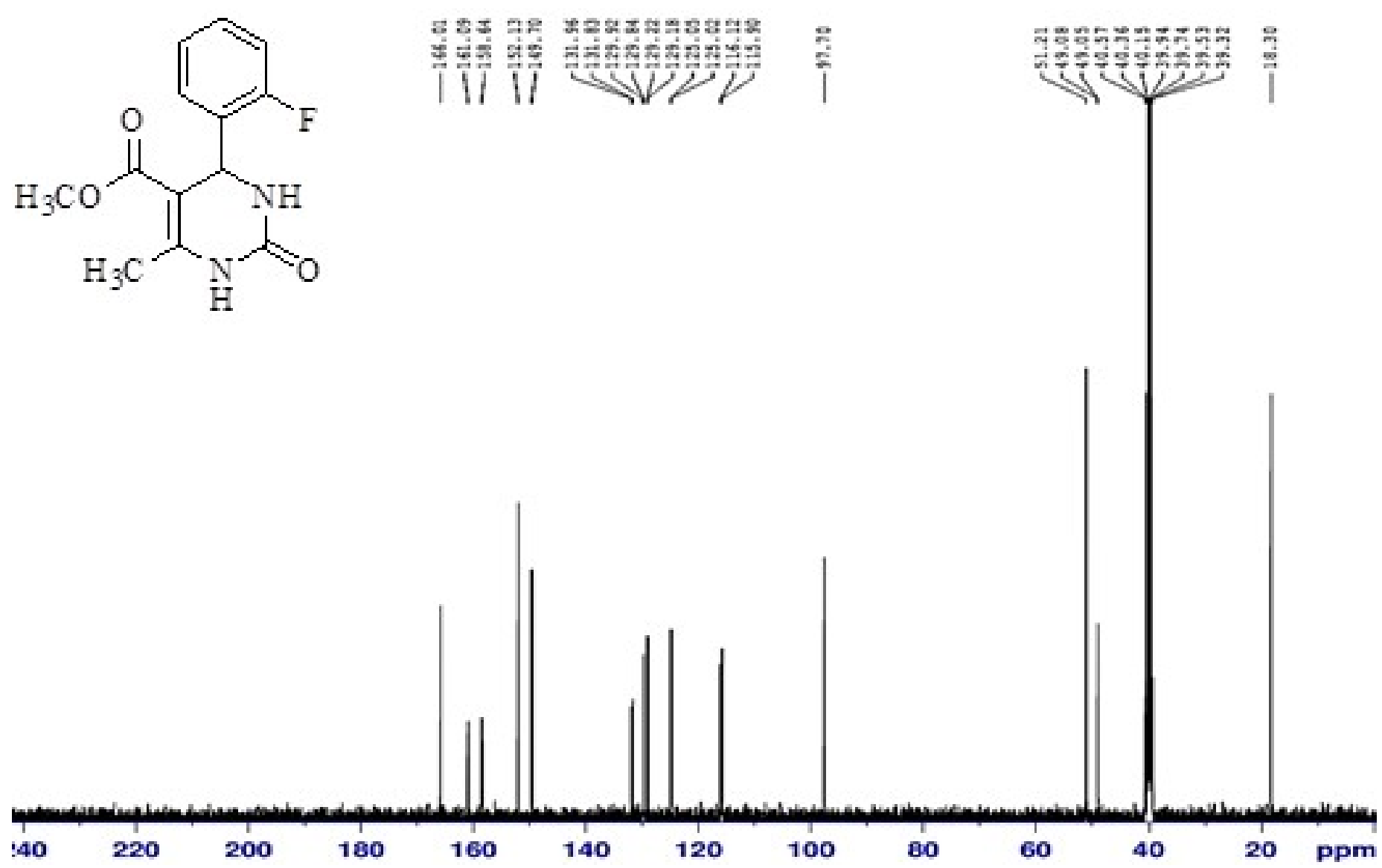

Figure S64. ^{13}C NMR spectrum of the methyl 4-(2-fluorophenyl)-6-methyl-2-oxo-1,2,3,4 tetrahydropyrimidine-5-carboxylate (**9d**) product in DMSO

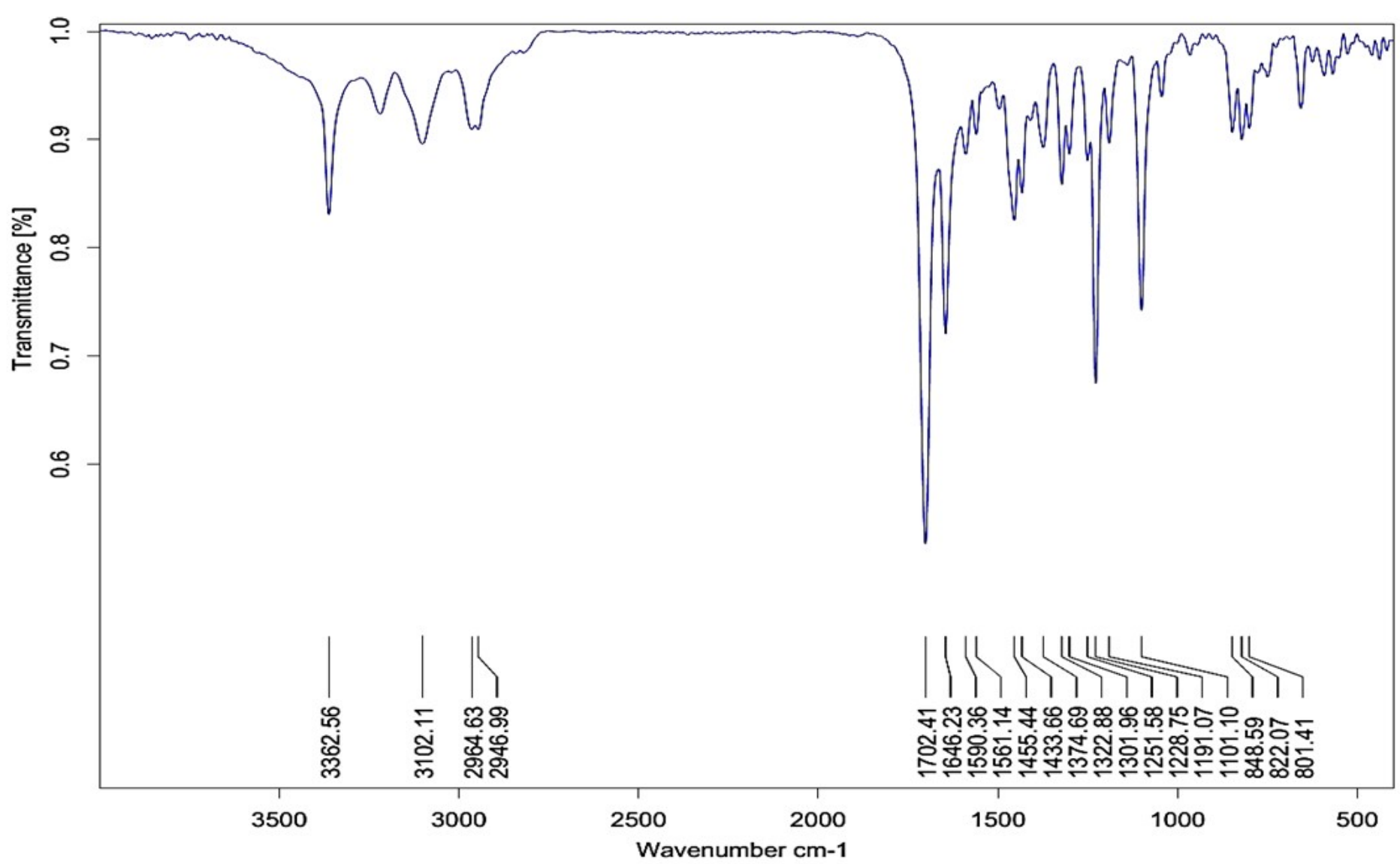

Figure S65. FTIR spectrum of the methyl 4-(2,4-dicholorophenyl)-6-methyl-2-oxo-1,2,3,4-tetrahydropyrimidine -5-carboxylate (**9e**) product

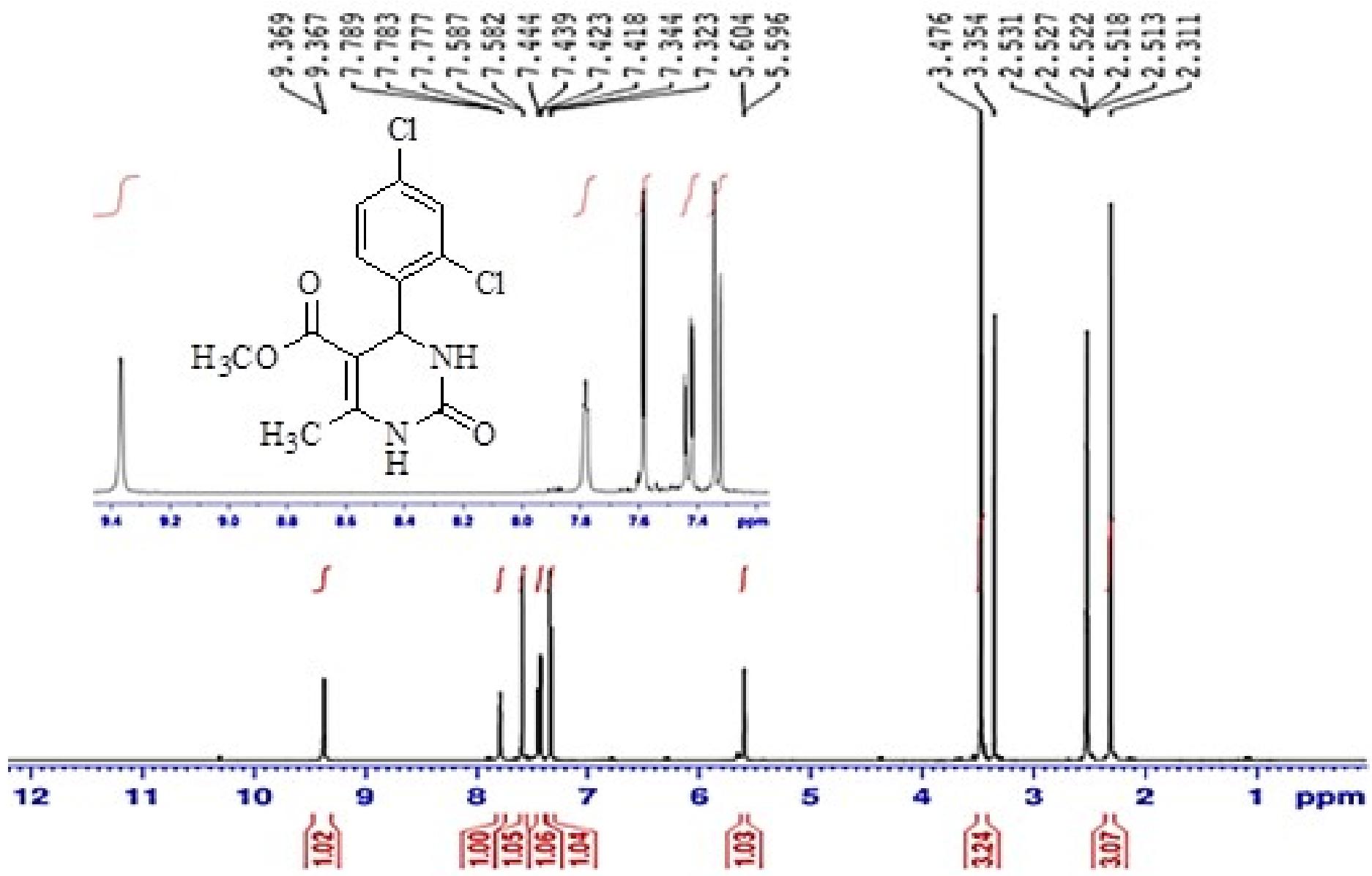

Figure S66. ^{1}H NMR spectrum of the methyl 4-(2,4-dicholorophenyl)-6-methyl-2-oxo-1,2,3,4-tetrahydropyrimidine -5-carboxylate (**9e**) product in DMSO

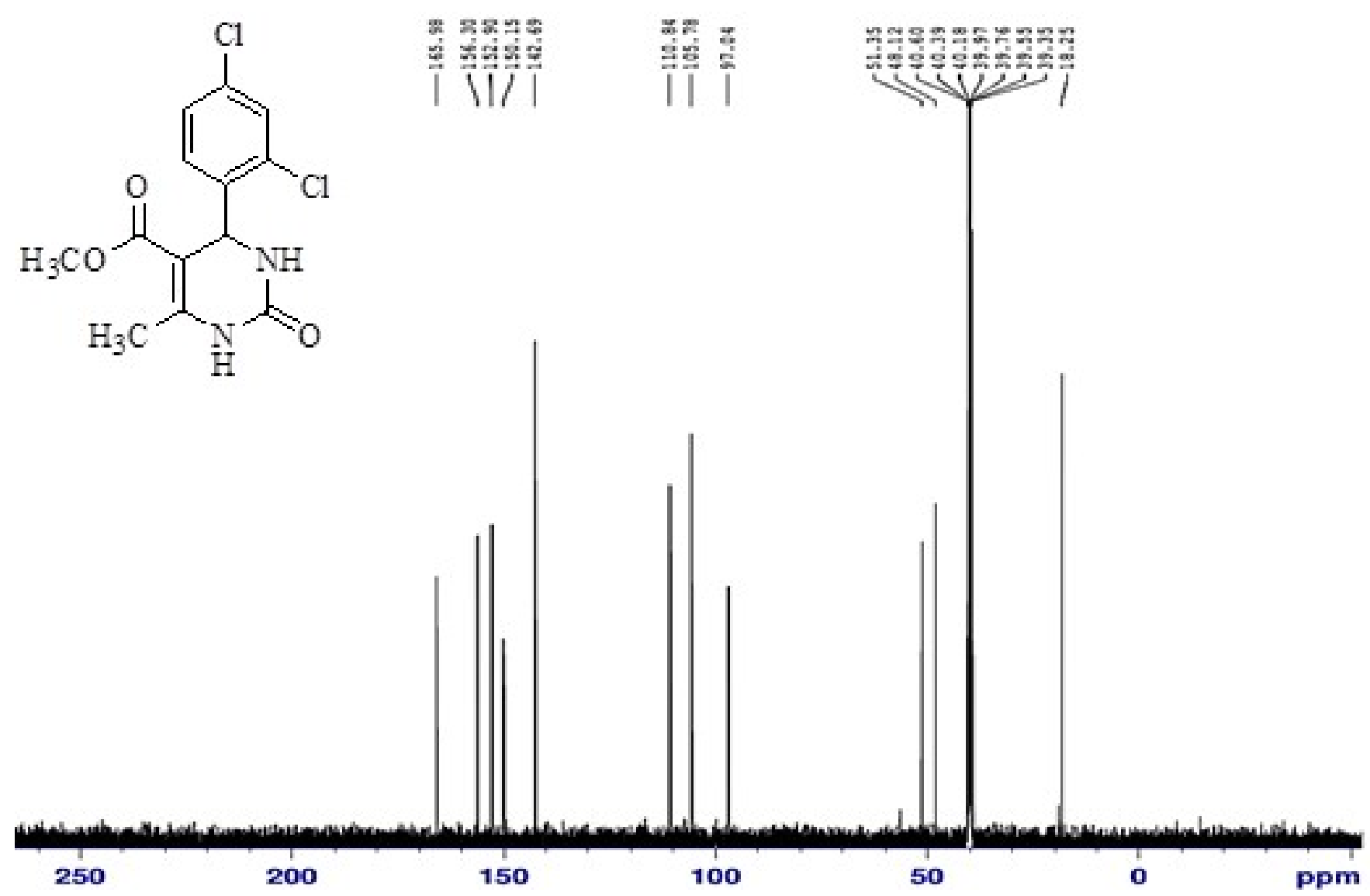

Figure S67. ^{13}C NMR spectrum of the methyl 4-(2,4-dicholorophenyl)-6-methyl-2-oxo-1,2,3,4 tetrahydropyrimidine-5-carboxylate (**9e**) product in DMSO

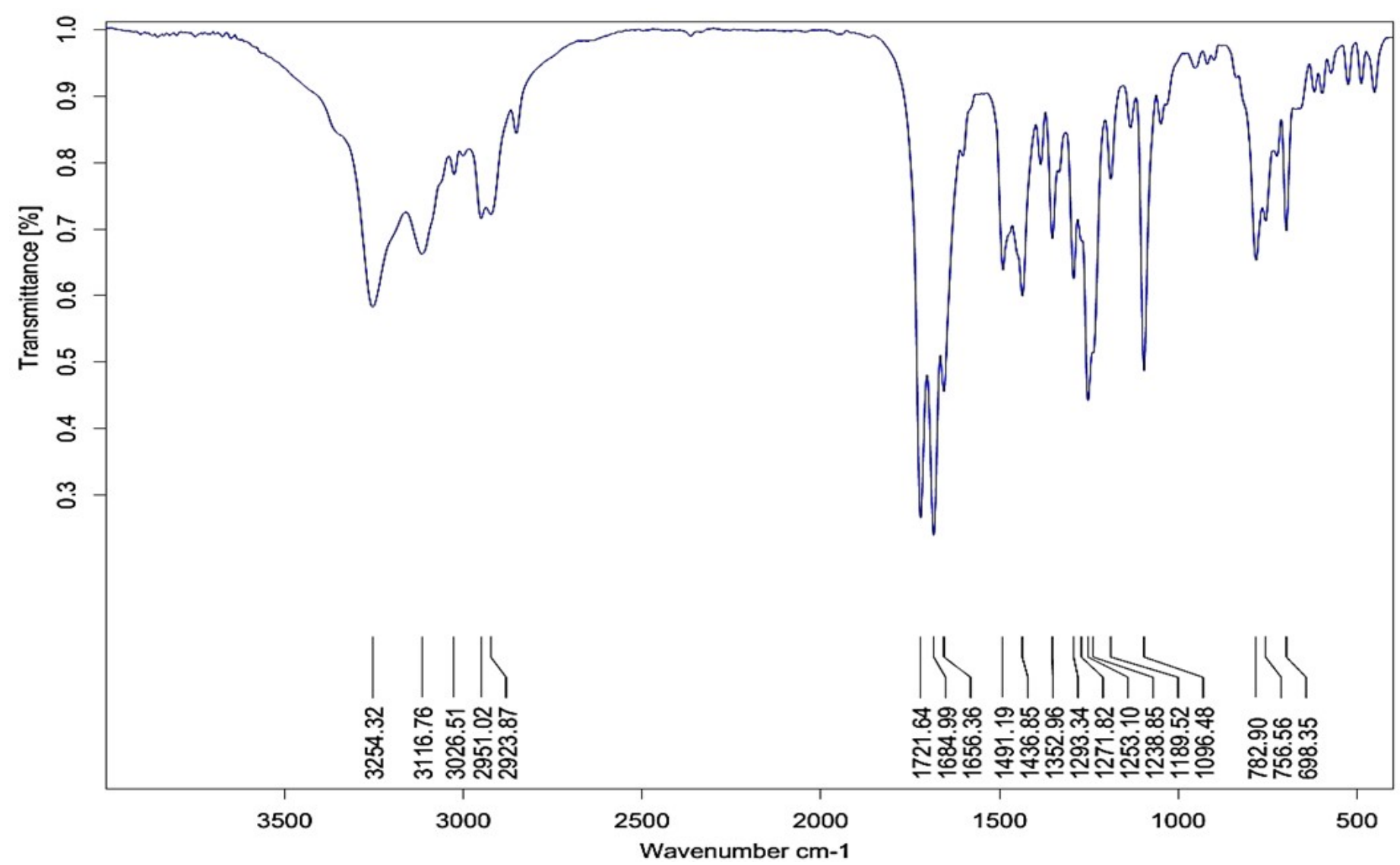

Figure S68. FTIR spectrum of the methyl 4-(4-nitrophenyl)-6-methyl-2-oxo-1,2,3,4-tetrahydropyrimidine-5-carboxylate (**9f**) product

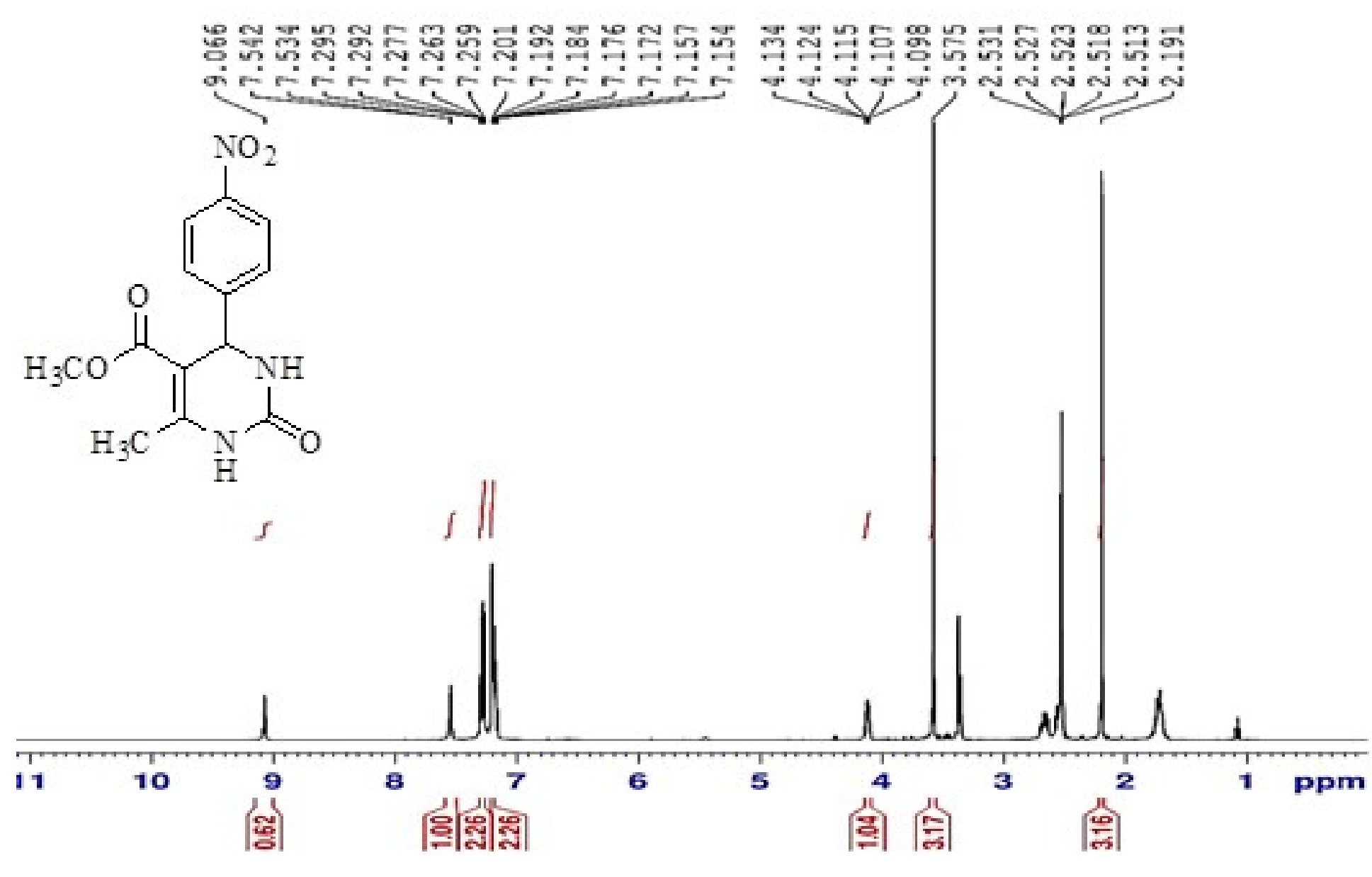

Figure S69. ^{1}H-NMR spectrum of the methyl 4-(4-nitrophenyl)-6-methyl-2-oxo-1,2,3,4-tetrahydropyrimidine-5-carboxylate (**9f**) product in DMSO

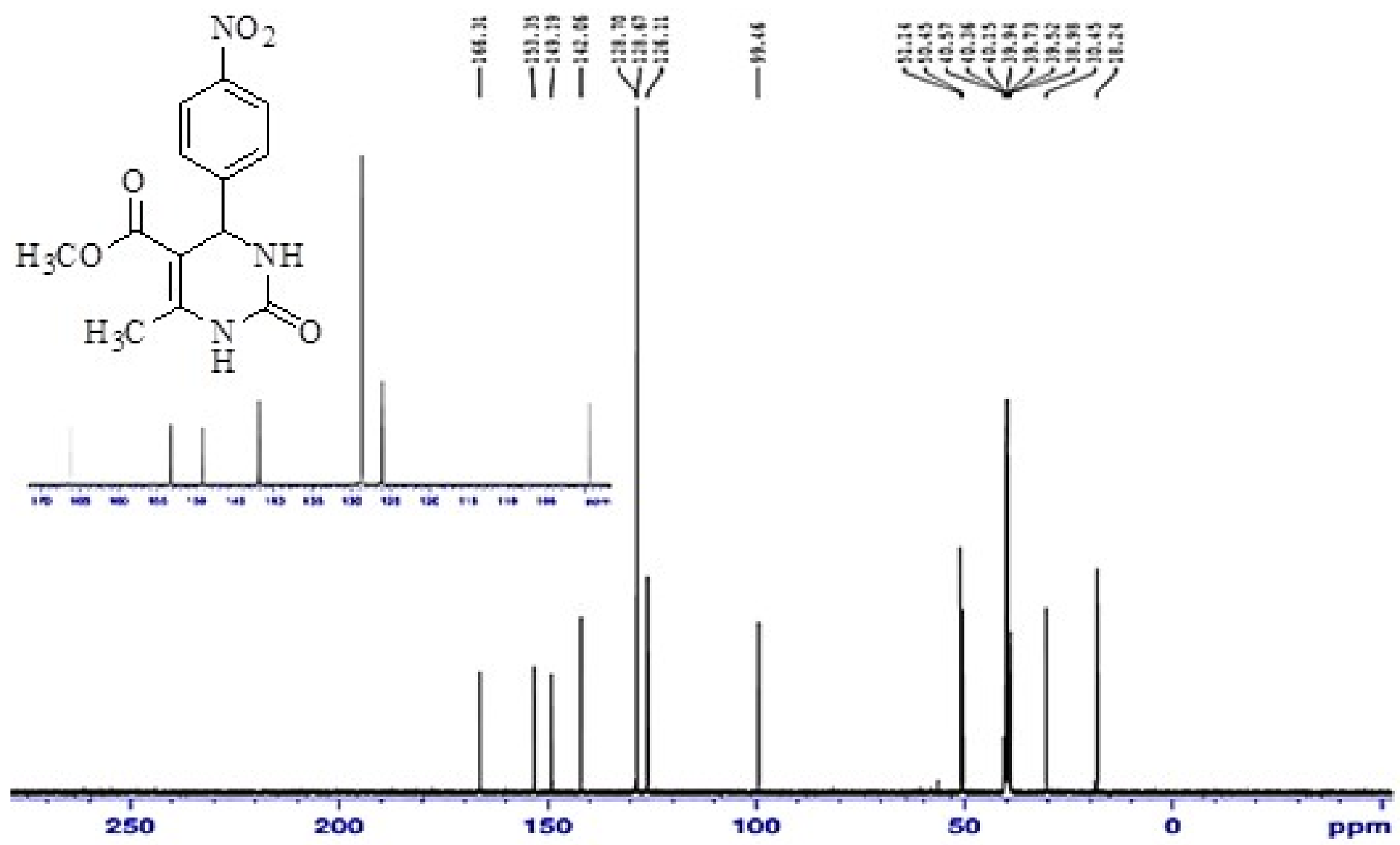

Figure S70. ^{13}C-NMR spectrum of the methyl 4-(4-nitrophenyl)-6-methyl-2-oxo-1,2,3,4-tetrahydropyrimidine-5-carboxylate (**9f**) product in DMSO

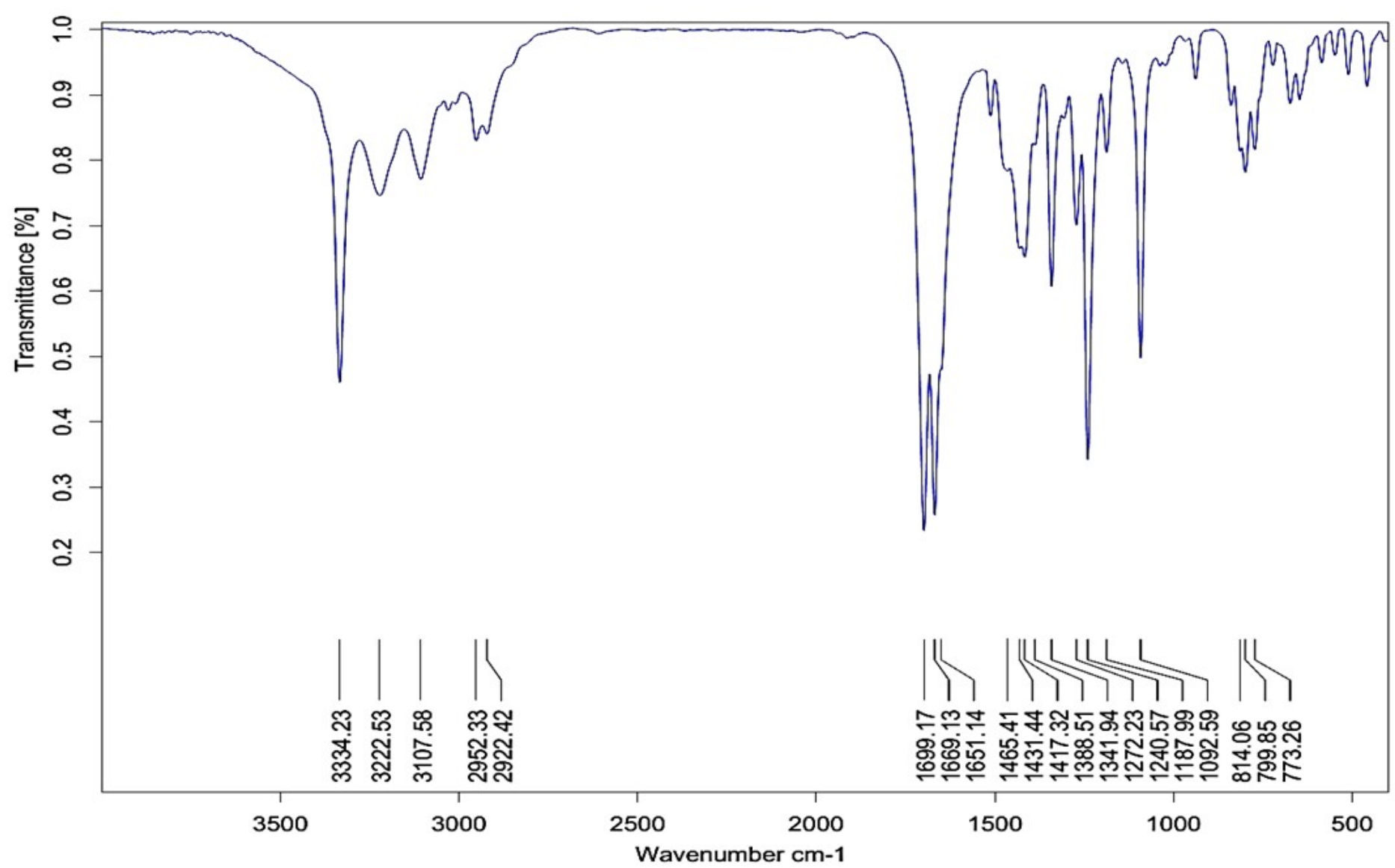

Figure S71. FTIR spectrum of the methyl 6-methyl-2-oxo-4-p-tolyl-1,2,3,4-tetrahydropyrimidine-5-carboxylate (**9g**) product

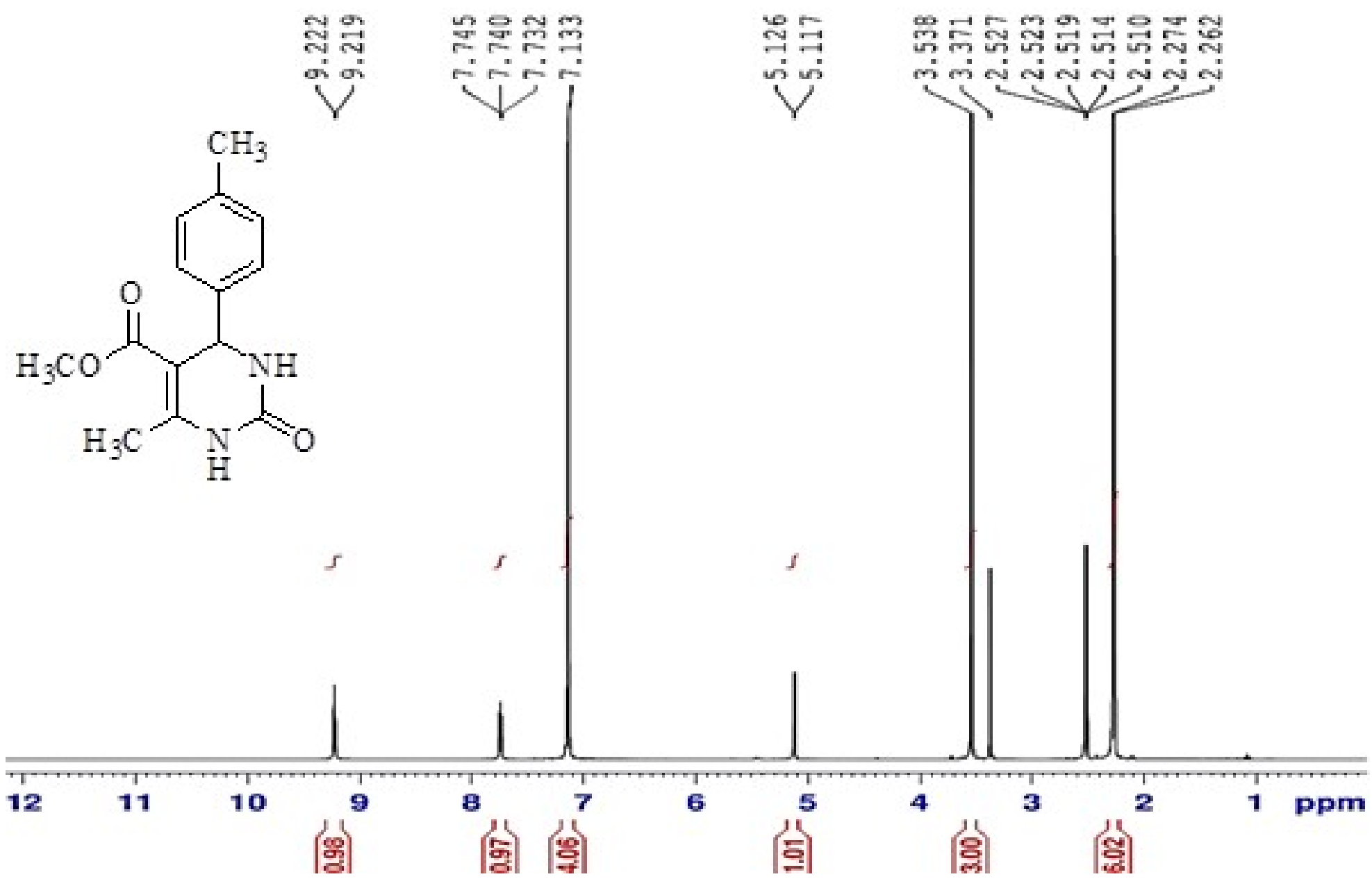

Figure S72. ^{1}H-NMR spectrum of the methyl 6-methyl-2-oxo-4-p-tolyl-1,2,3,4-tetrahydropyrimidine-5-carboxylate (**9g**) product in DMSO

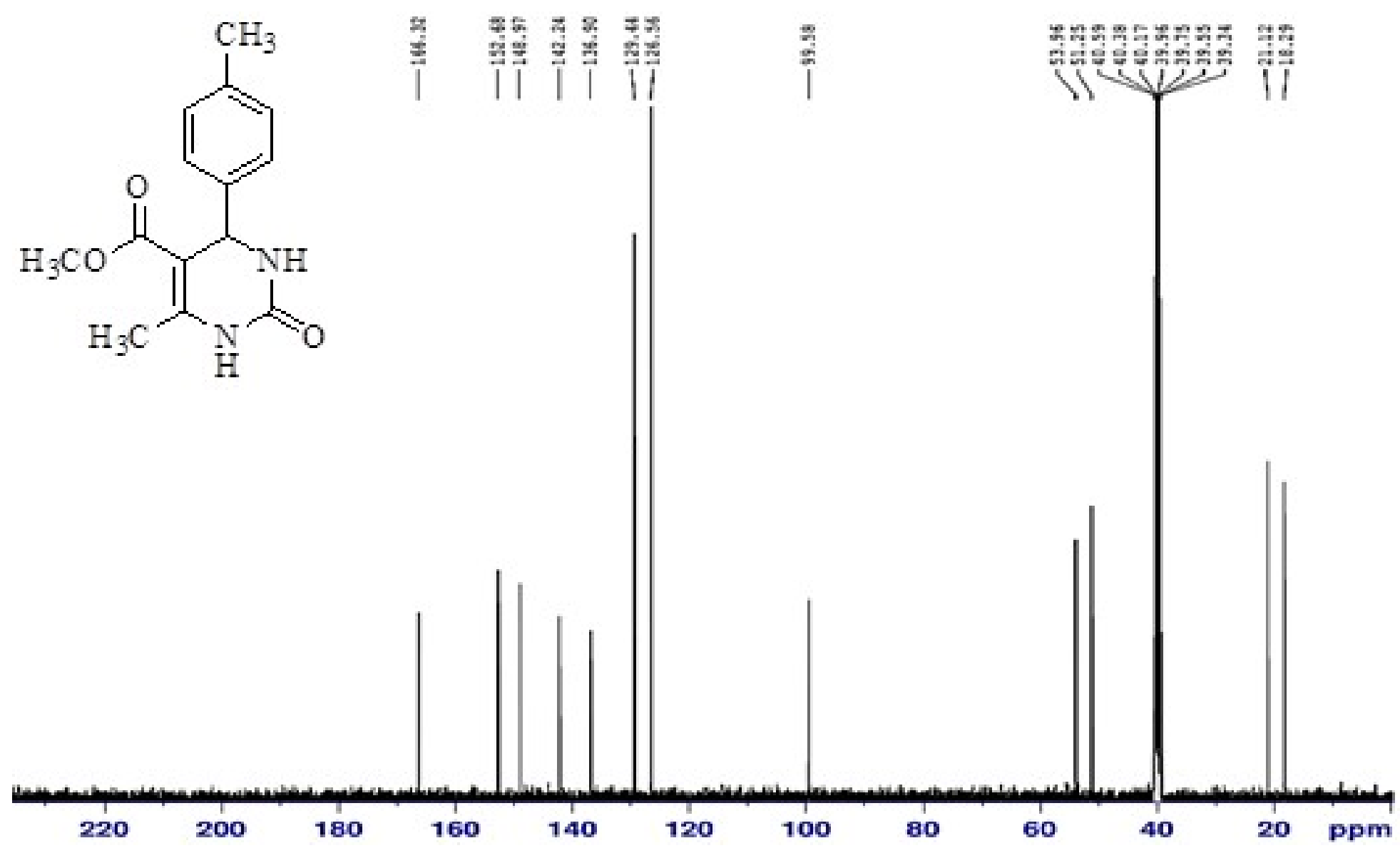

Figure S73. ^{13}C-NMR spectrum of the methyl 6-methyl-2-oxo-4-p-tolyl-1,2,3,4-tetrahydropyrimidine-5-carboxylate (**9g**) product in DMSO

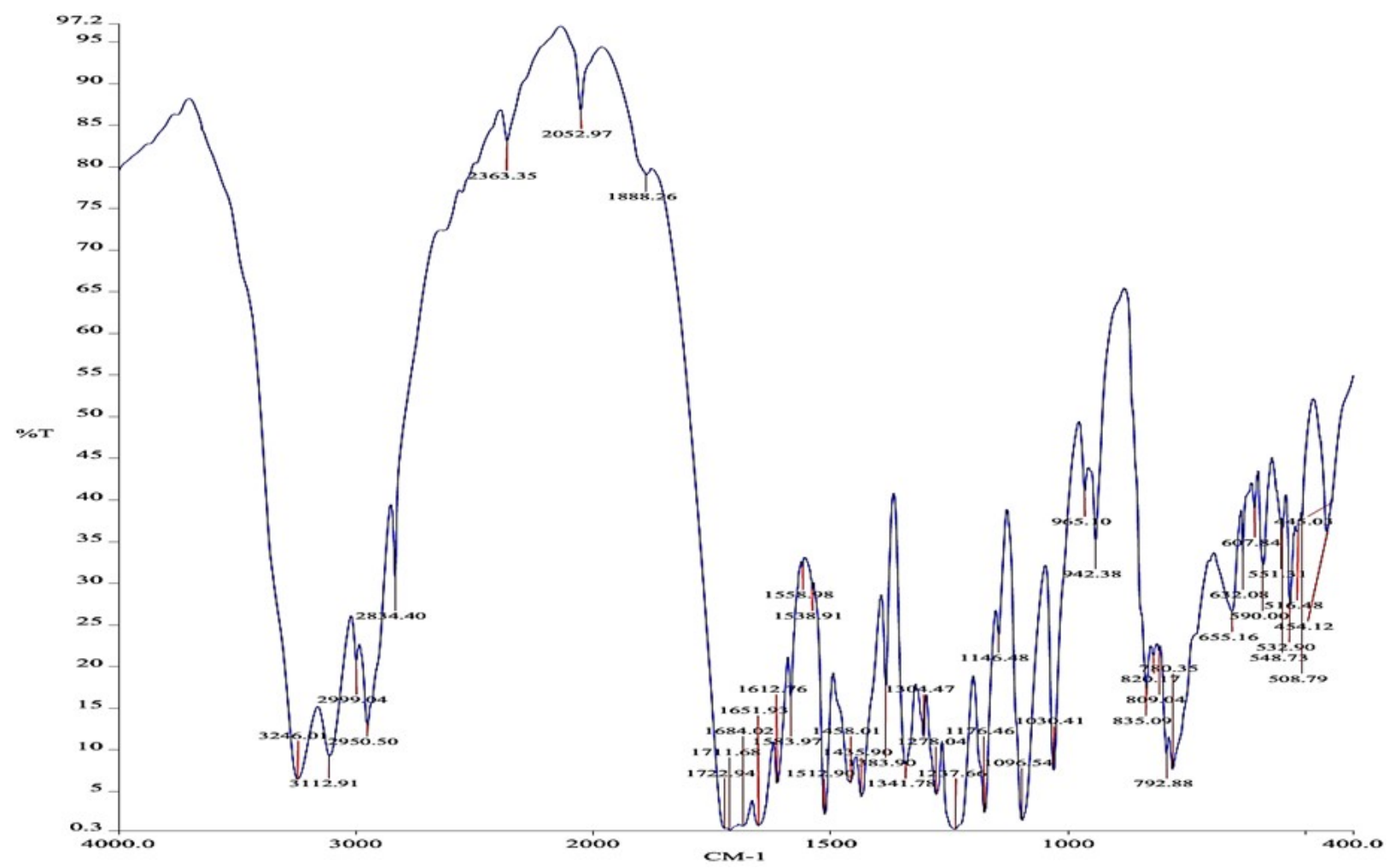

Figure S74. FTIR spectrum of the methyl 4-(4-methoxyphenyl)-6-methyl-2-oxo-1,2,3,4-tetrahydropyrimidine-5-carboxylate (**9h**) product

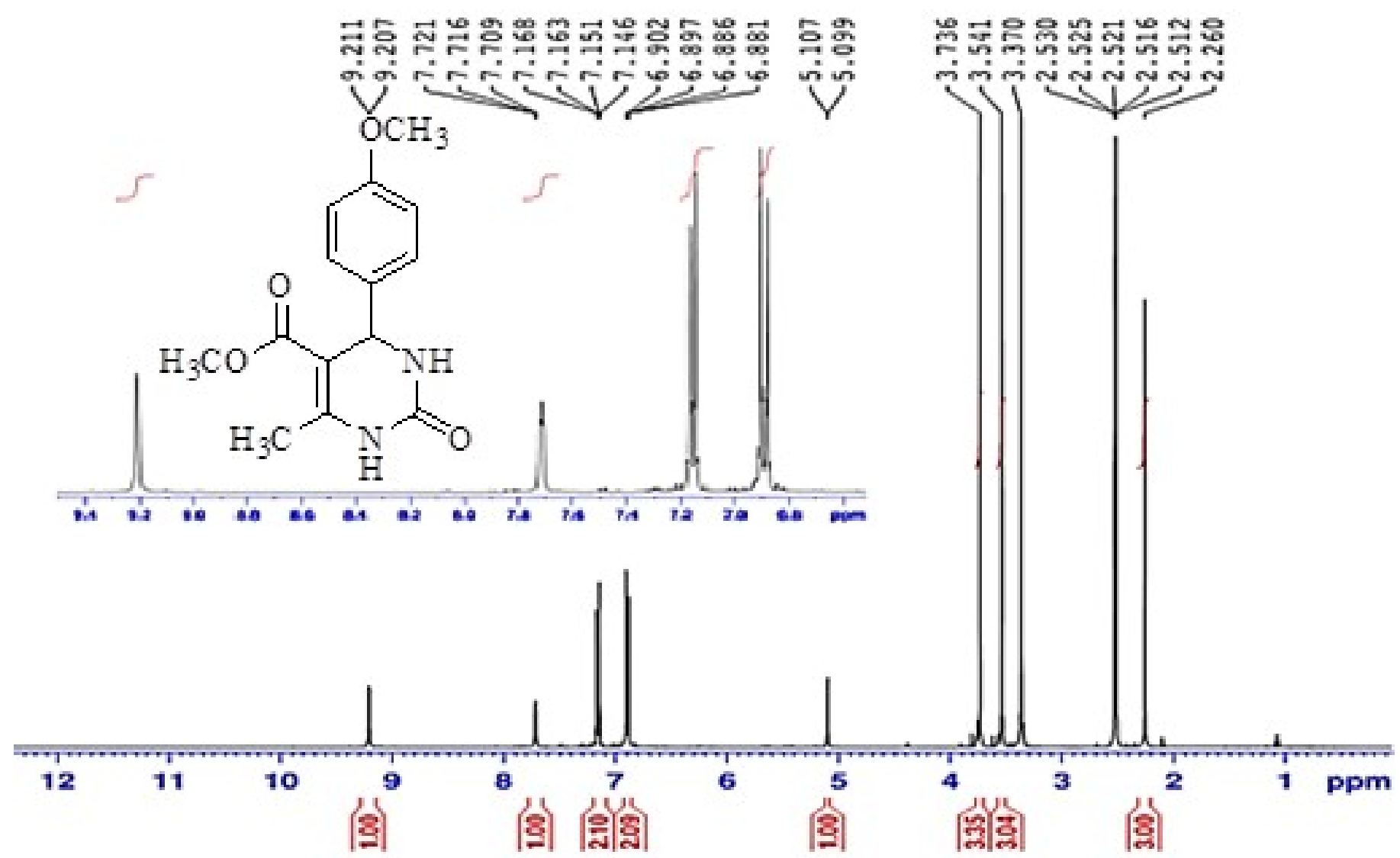

Figure S75. ^{1}H-NMR spectrum of the methyl 4-(4-methoxyphenyl)-6-methyl-2-oxo-1,2,3,4-tetrahydropyrimidine-5-carboxylate (**9h**) product in DMSO

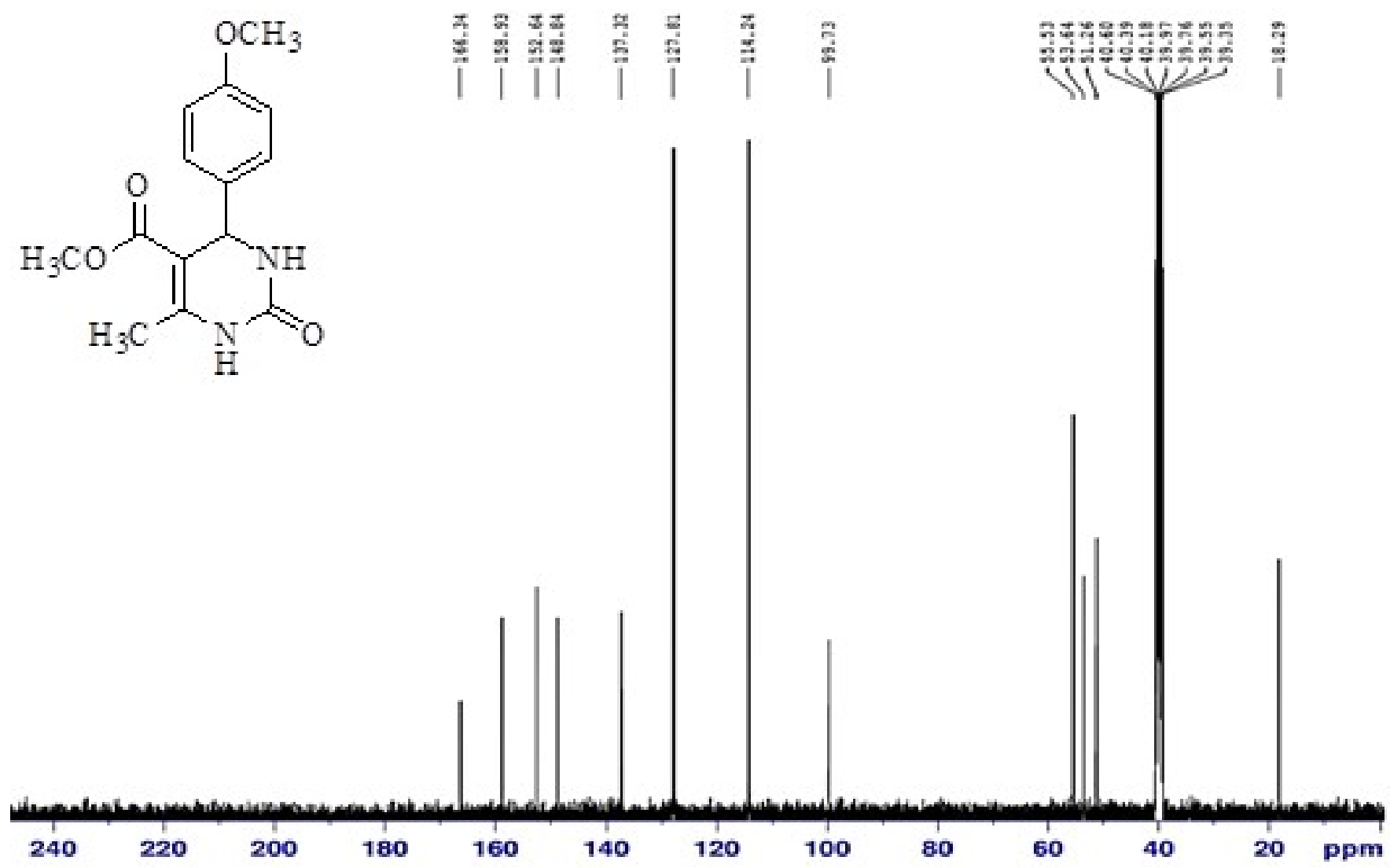

Figure S76. ^{13}C NMR spectrum of the methyl 4-(4-methoxyphenyl)-6-methyl-2-oxo-1,2,3,4-tetrahydropyrimidine-5-carboxylate (**9h**) product in DMSO

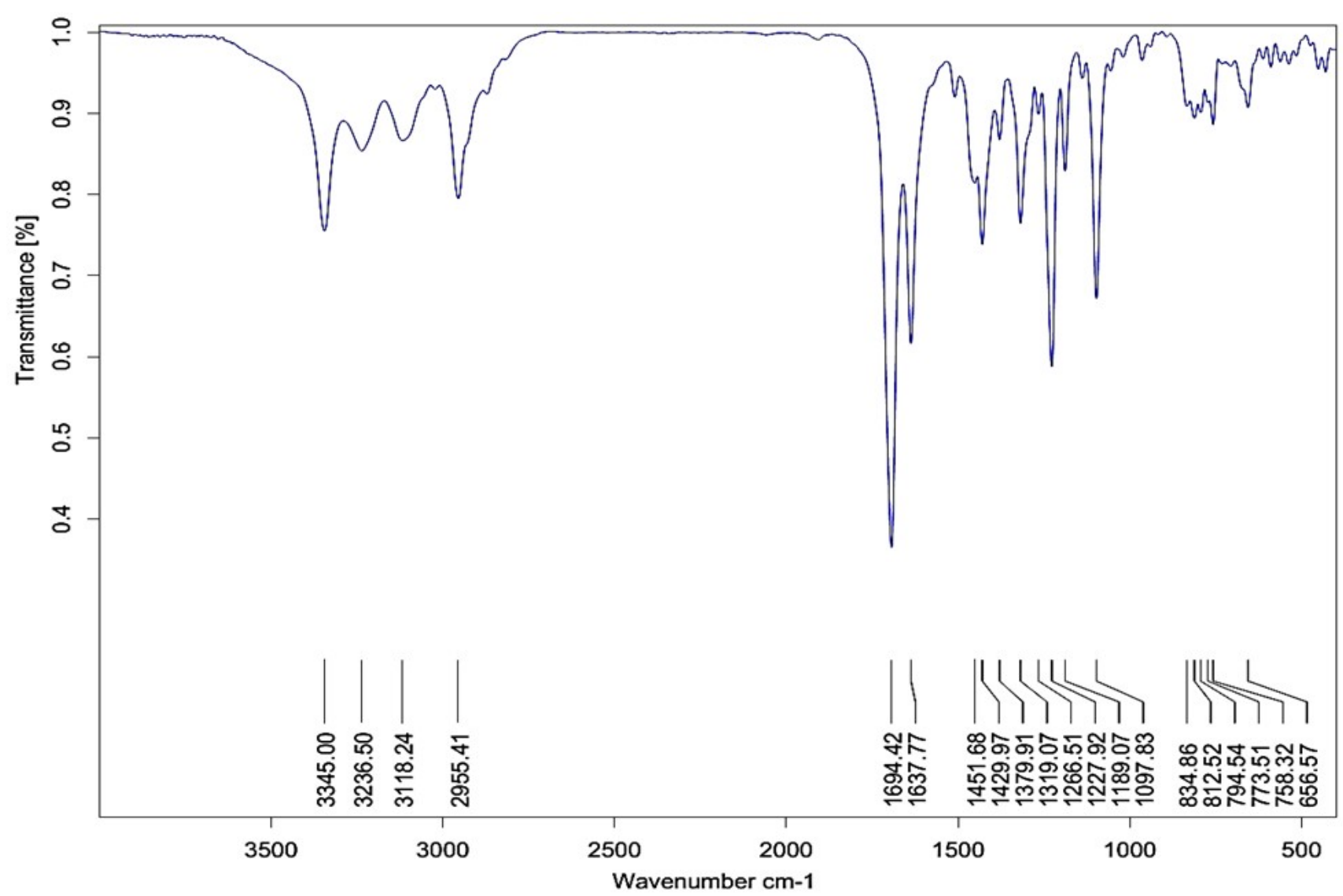

Figure S77. FTIR spectrum of the methyl 4-(4-isopropylphenyl)-6-methyl-2-oxo-1,2,3,4-tetrahydropyrimidine-5-carboxylate (**9i**) product

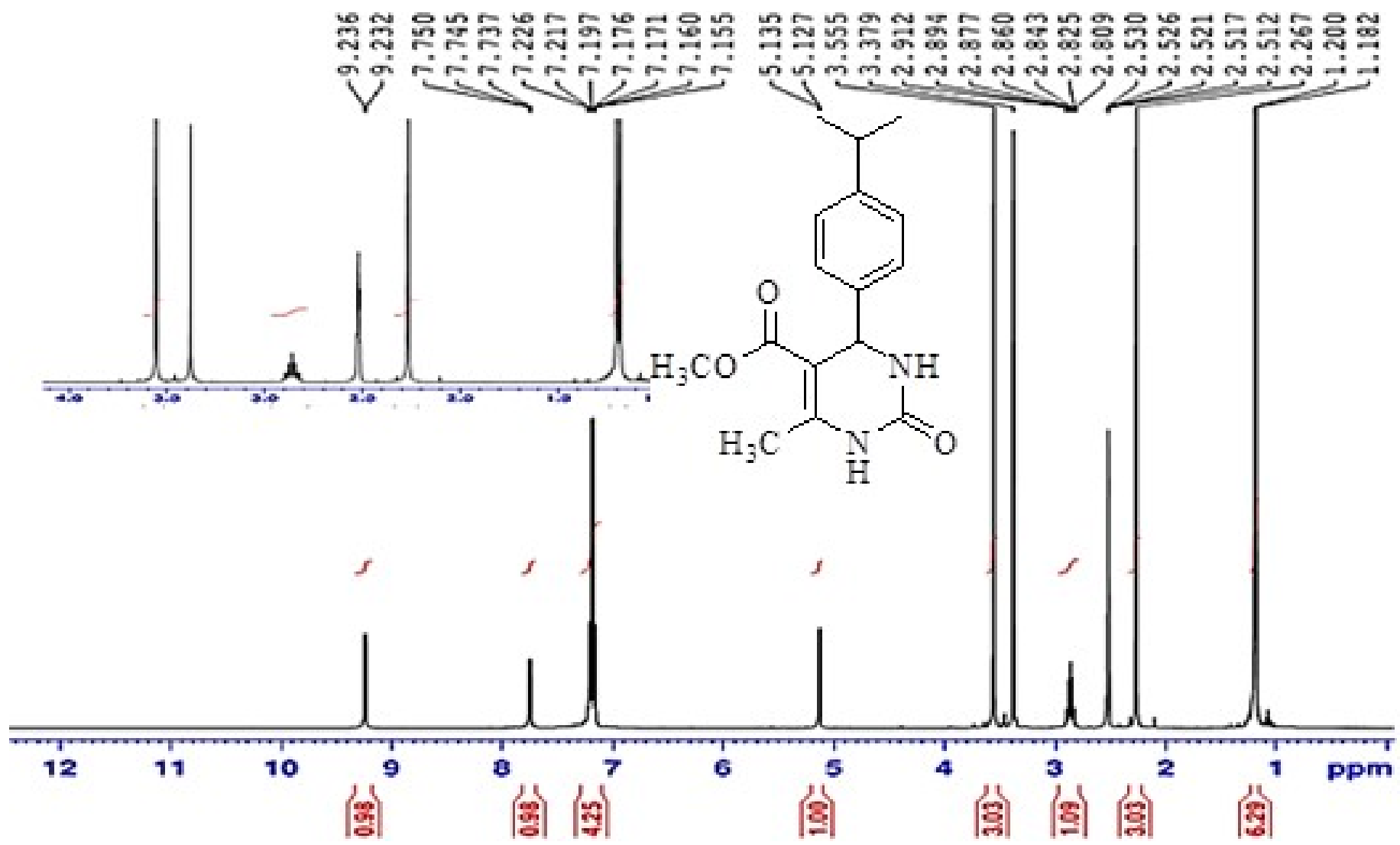

Figure S78. ^{1}H NMR spectrum of the methyl 4-(4-isopropylphenyl)-6-methyl-2-oxo-1,2,3,4-tetrahydropyrimidine-5-carboxylate (**9i**) product in DMSO

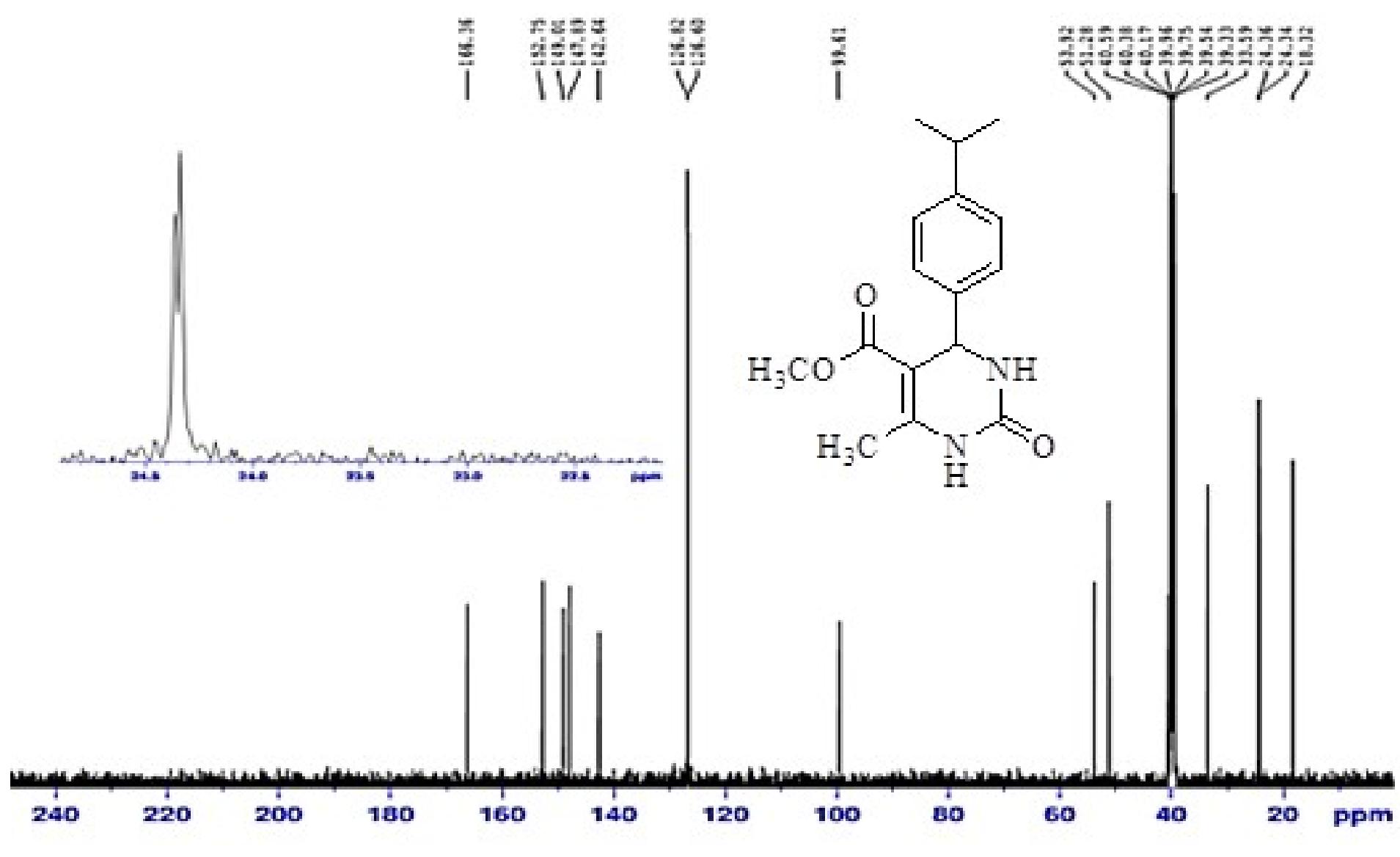

Figure S79. ^{13}C NMR spectrum of the methyl 4-(4-isopropylphenyl)-6-methyl-2-oxo-1,2,3,4-tetrahydropyrimidine-5-carboxylate (**9i**) product in DMSO

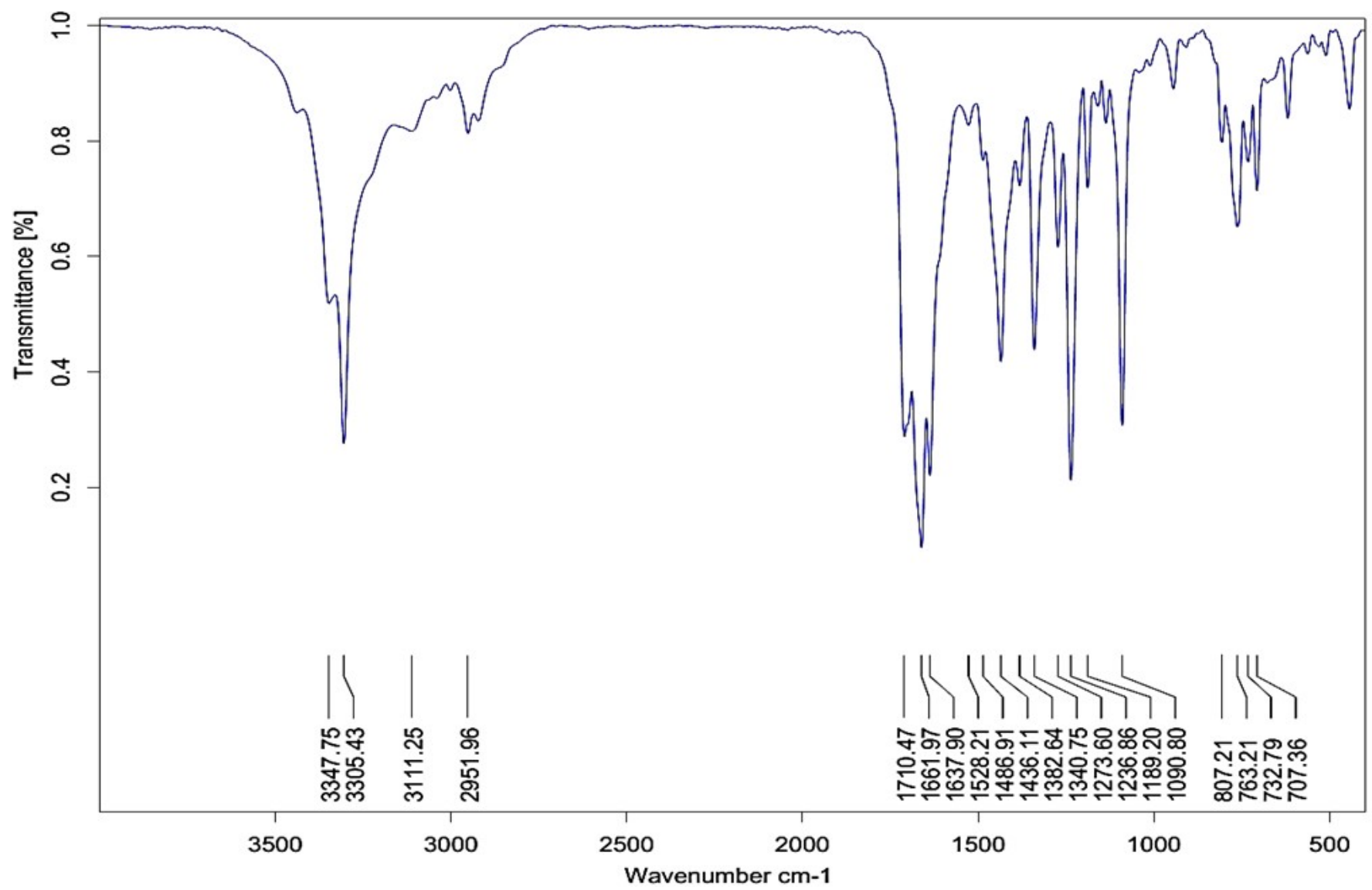

Figure S80. FTIR spectrum of the methyl 6-methyl-2-oxo-4-m-tolyl-1,2,3,4-tetrahydropyrimidine-5-carboxylate (**9j**) product

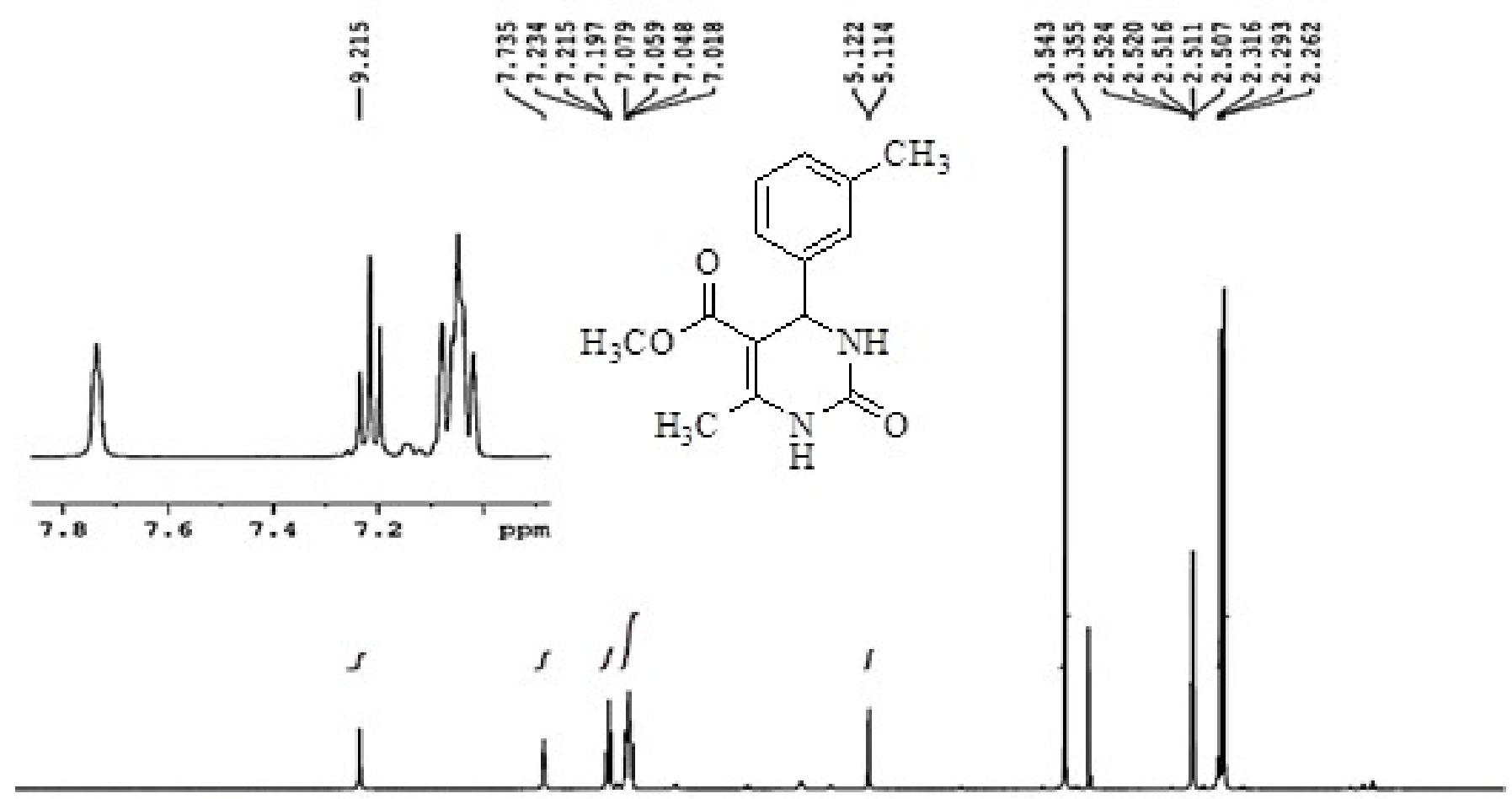

Figure S81. ^{1}H-NMR spectrum of the methyl 6-methyl-2-oxo-4-m-tolyl-1,2,3,4-tetrahydropyrimidine-5-carboxylate (**9j**) product in DMSO

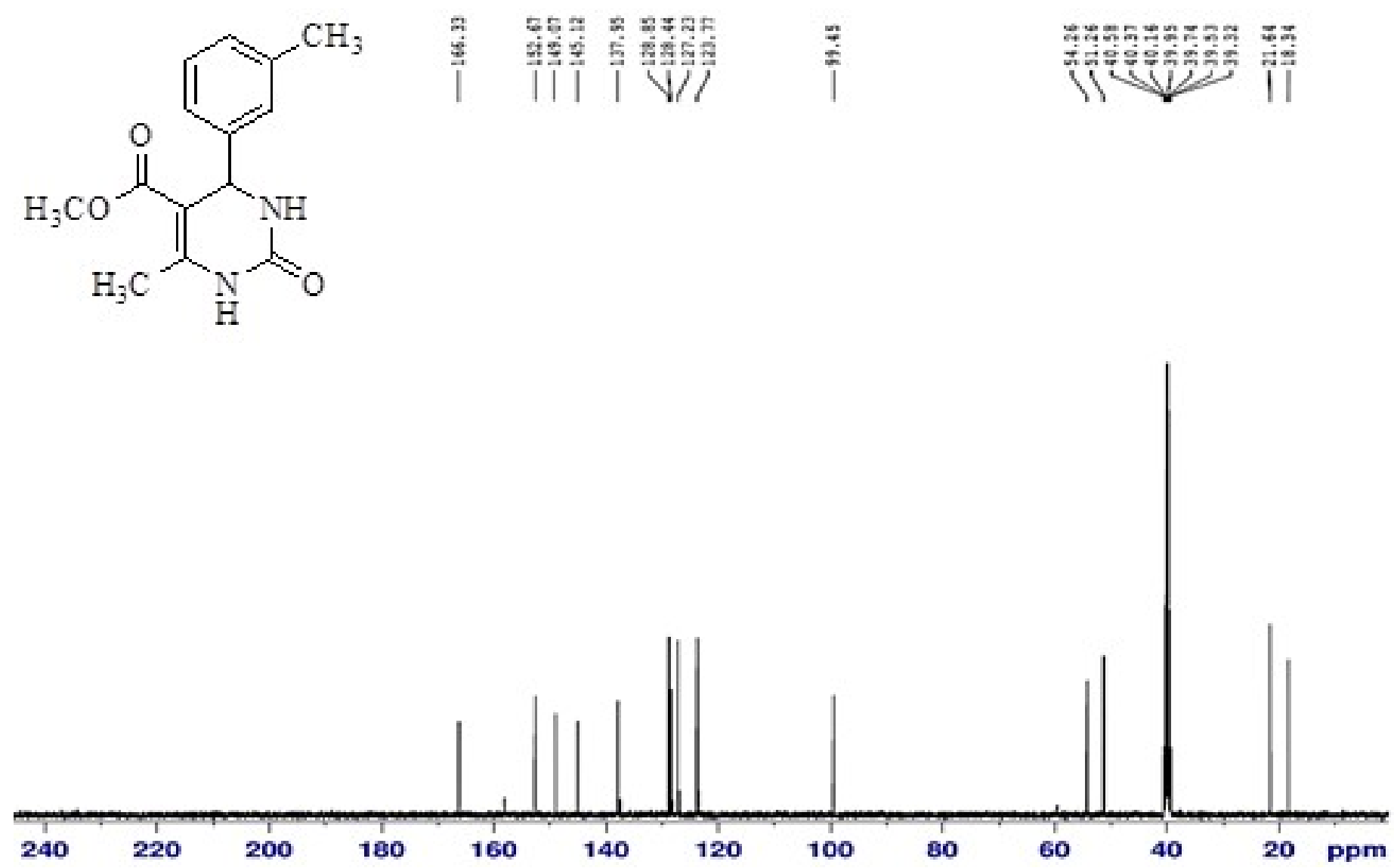

Figure S82. ^{13}C-NMR spectrum of the methyl 6-methyl-2-oxo-4-m-tolyl-1,2,3,4-tetrahydropyrimidine-5-carboxylate (**9j**) product in DMSO

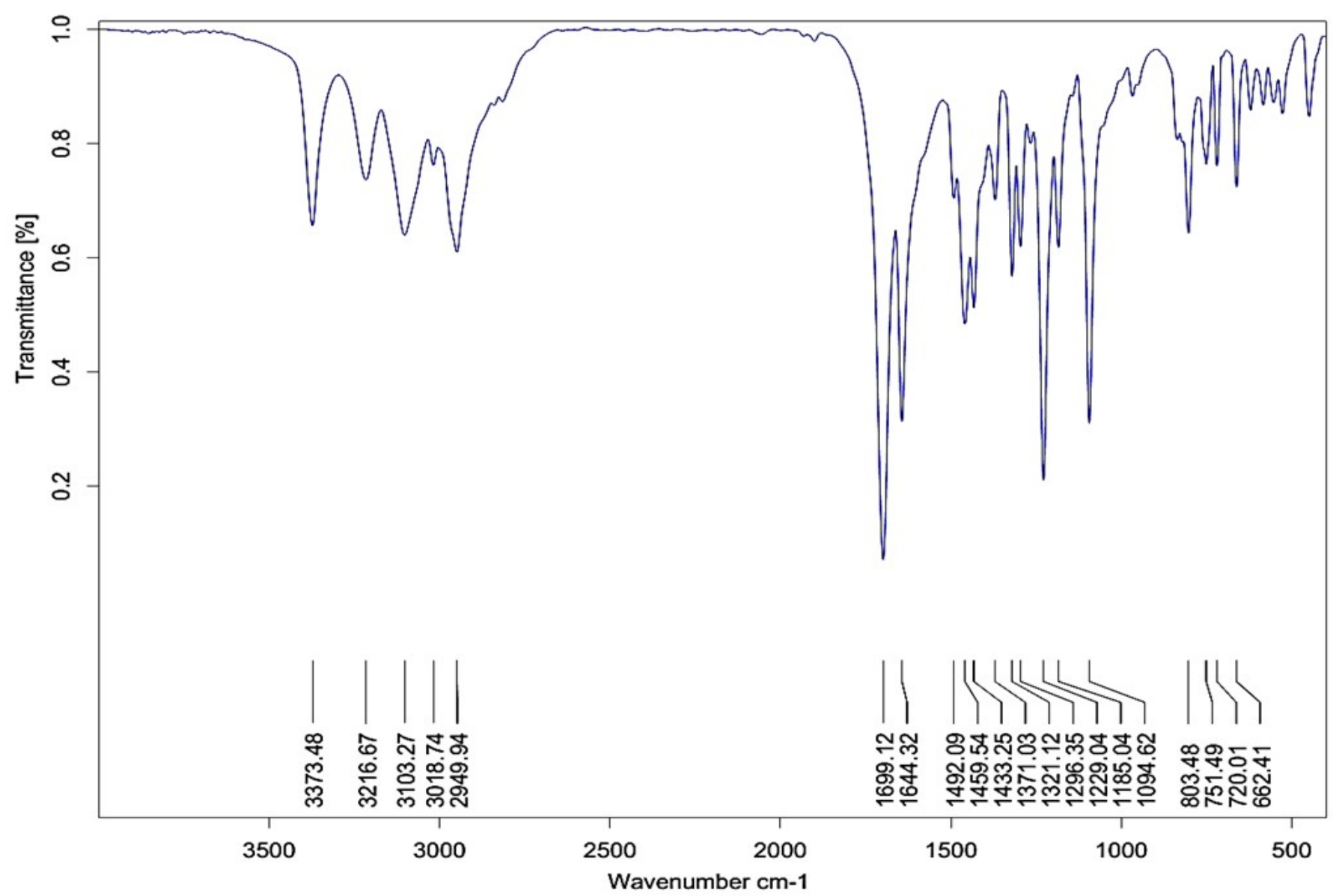

Figure S83. FTIR spectrum of the methyl 6-methyl-2-oxo-4-o-tolyl-1,2,3,4-tetrahydropyrimidine-5-carboxylate (**9k**) product.

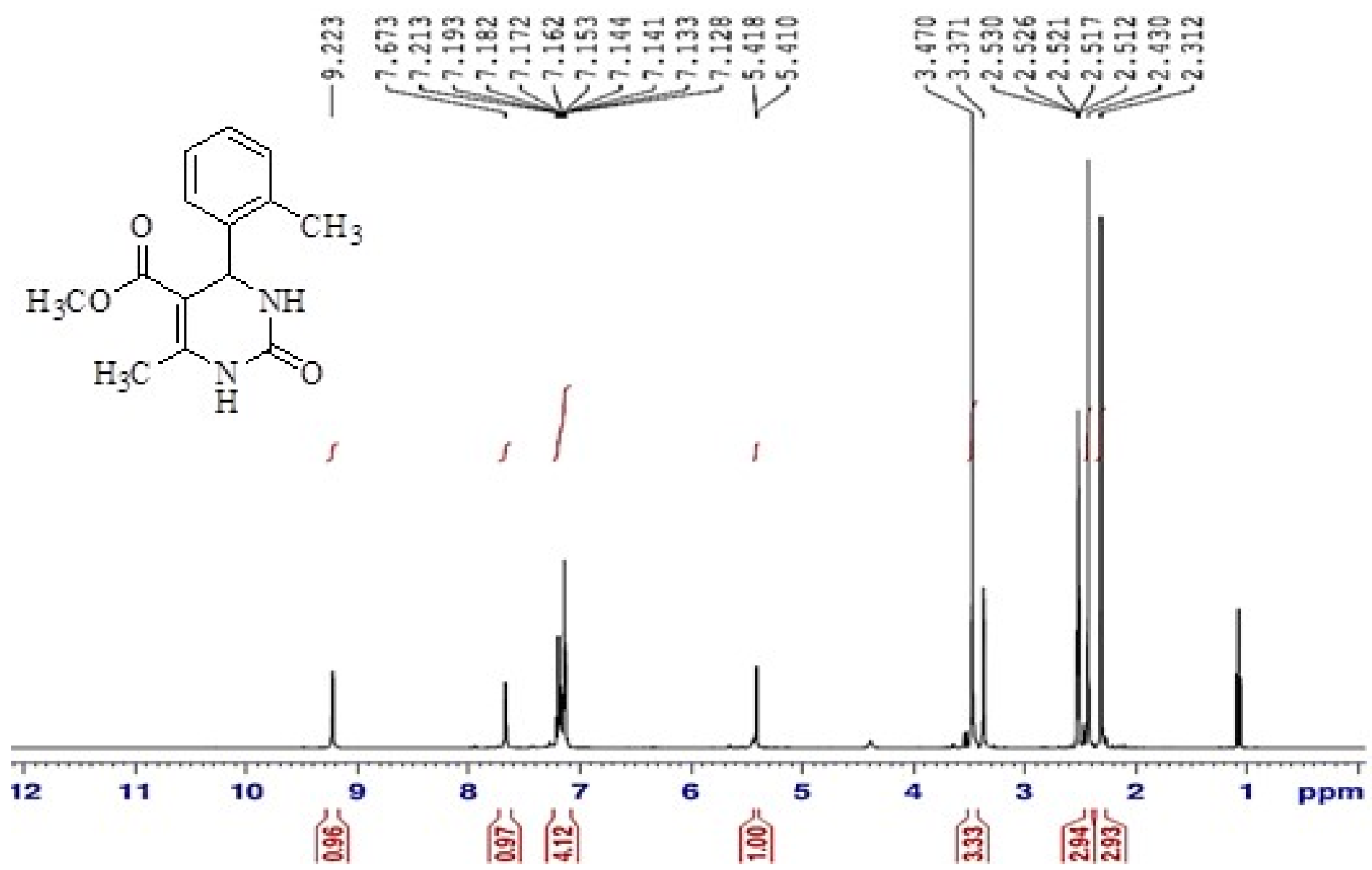

Figure S84. ^{1}H-NMR spectrum of the methyl 6-methyl-2-oxo-4-o-tolyl-1,2,3,4-tetrahydropyrimidine-5-carboxylate (**9k)** product in DMSO

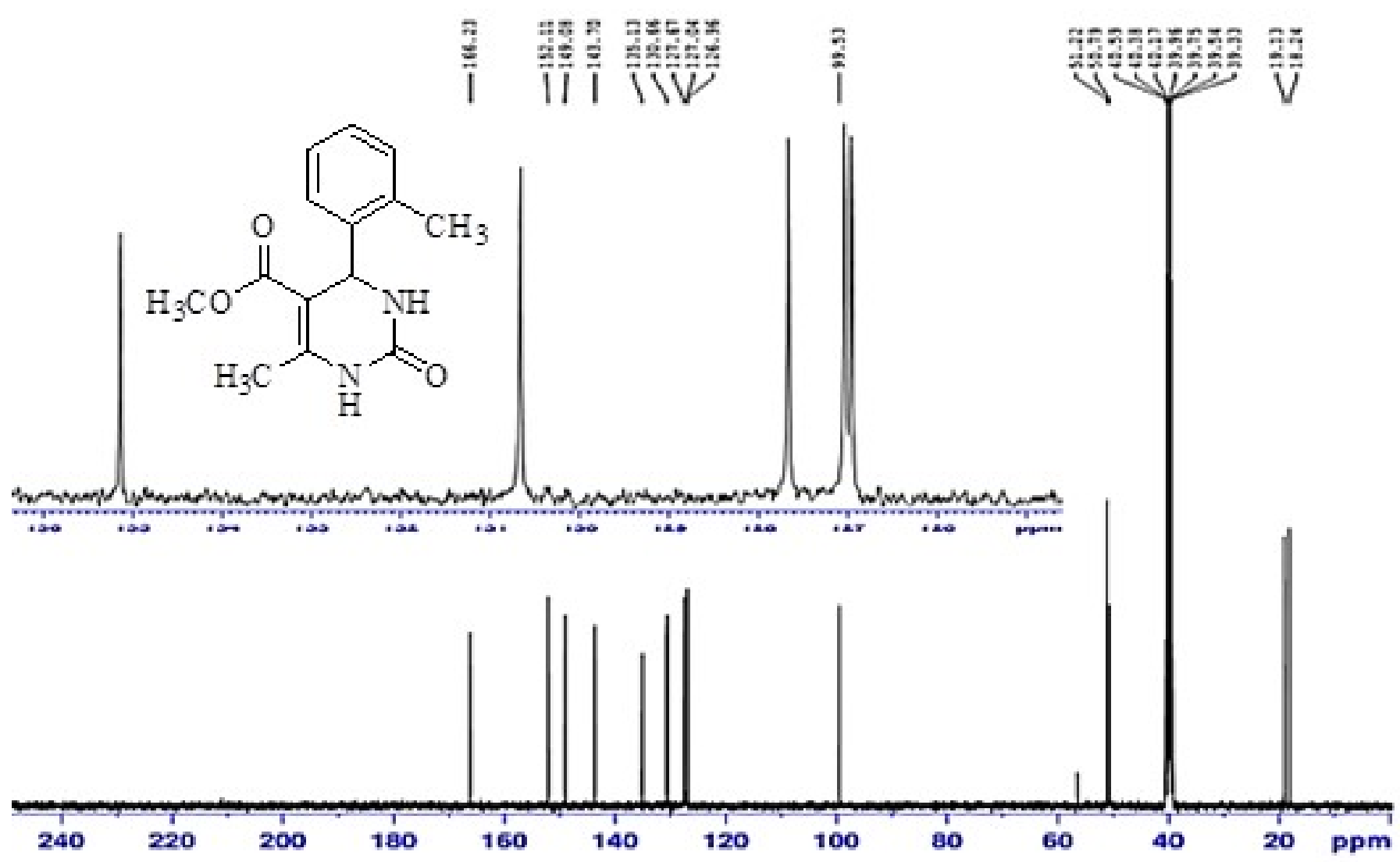

Figure S85. ^{13}C-NMR spectrum of the methyl 6-methyl-2-oxo-4-o-tolyl-1,2,3,4-tetrahydropyrimidine-5-carboxylate (**9k**) product in DMSO

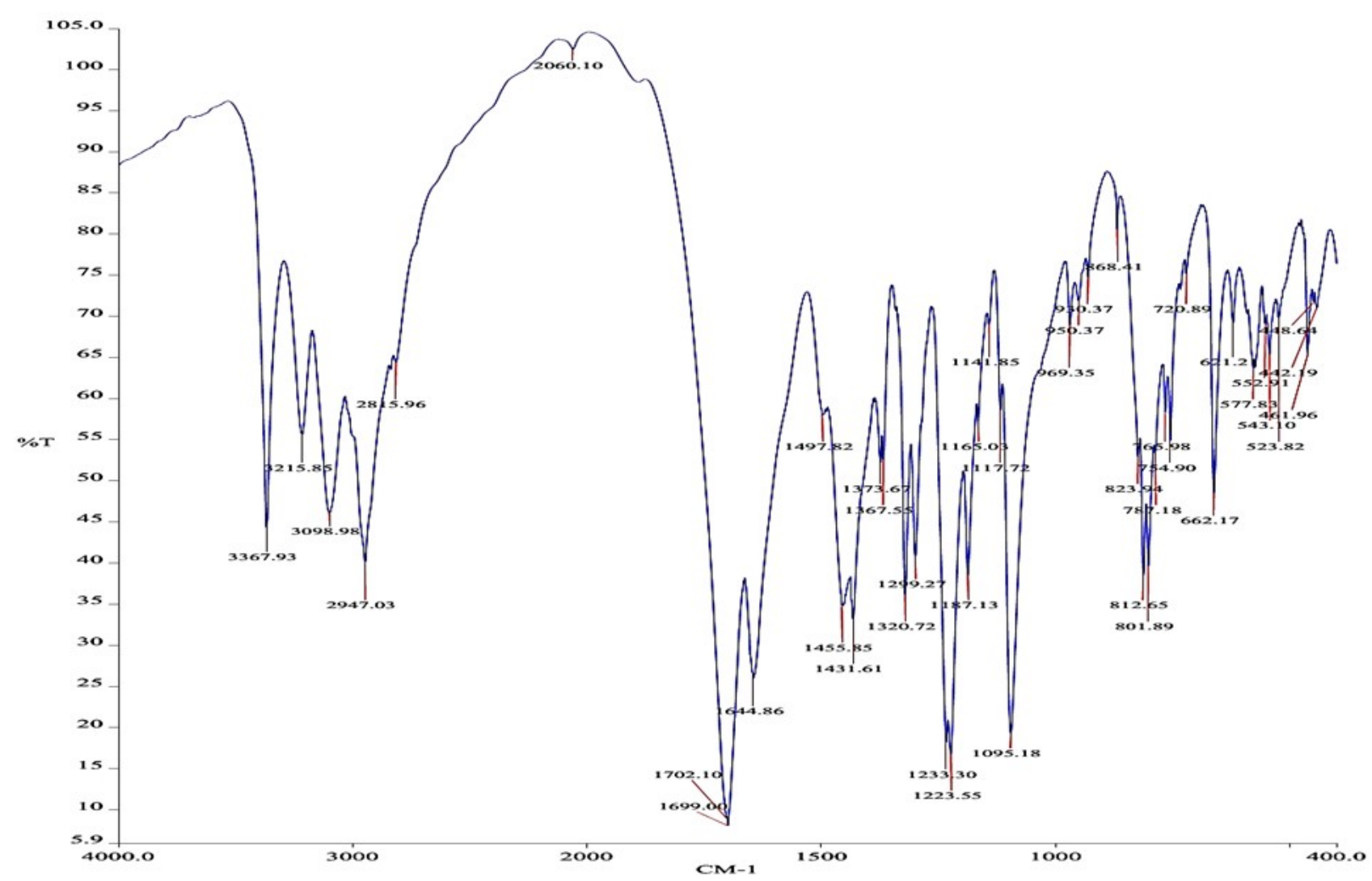

Figure S86. FTIR spectrum of the methyl 4-(2,4-dimethylphenyl)-6-methyl-2-oxo-1,2,3,4-tetrahydropyrimidine-5-carboxylate (**9l**) product

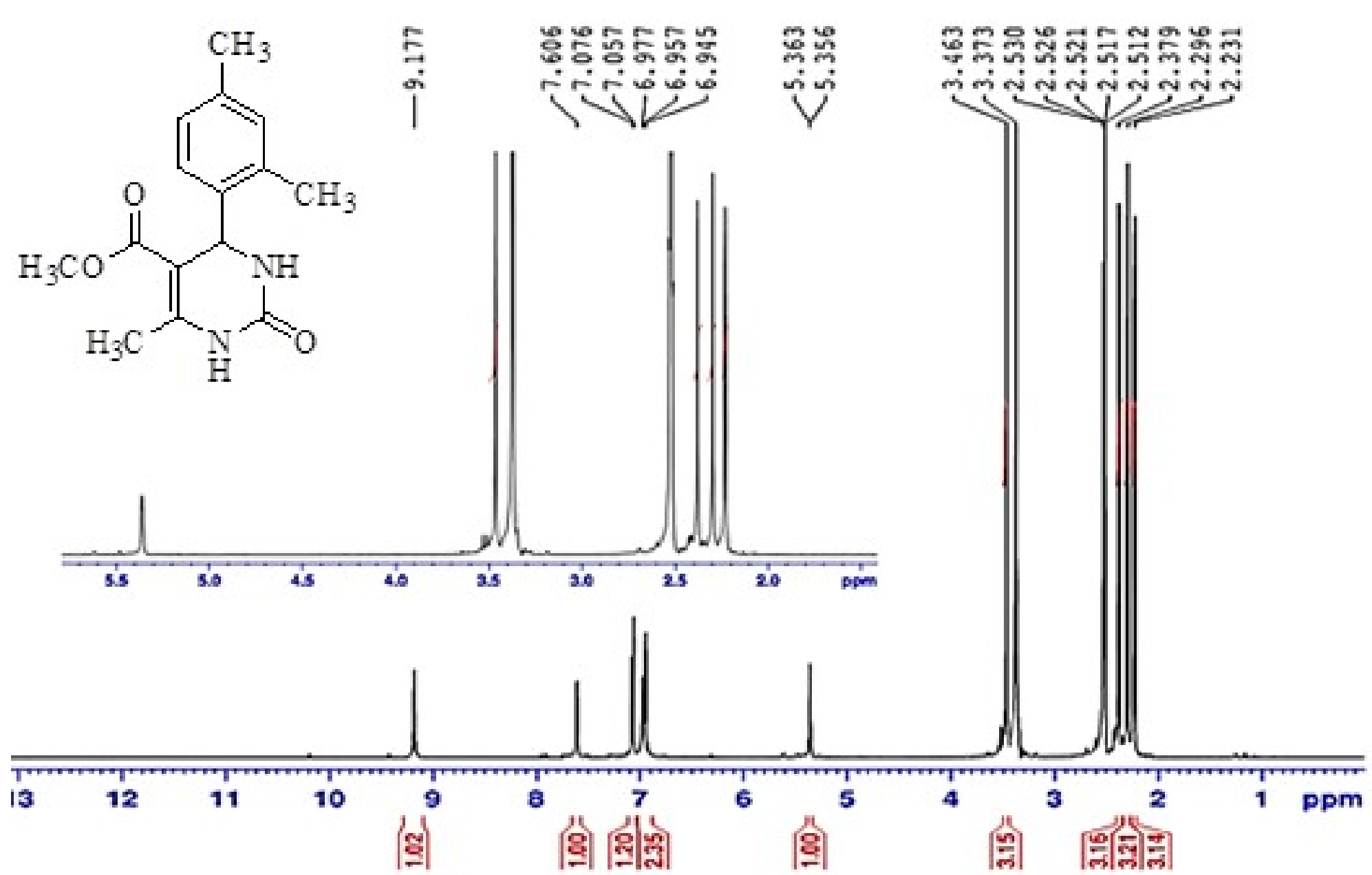

Figure S87. ^{1}H-NMR spectrum of the methyl 4-(2,4-dimethylphenyl)-6-methyl-2-oxo-1,2,3,4-tetrahydropyrimidine-5-carboxylate (**9l**) product in DMSO

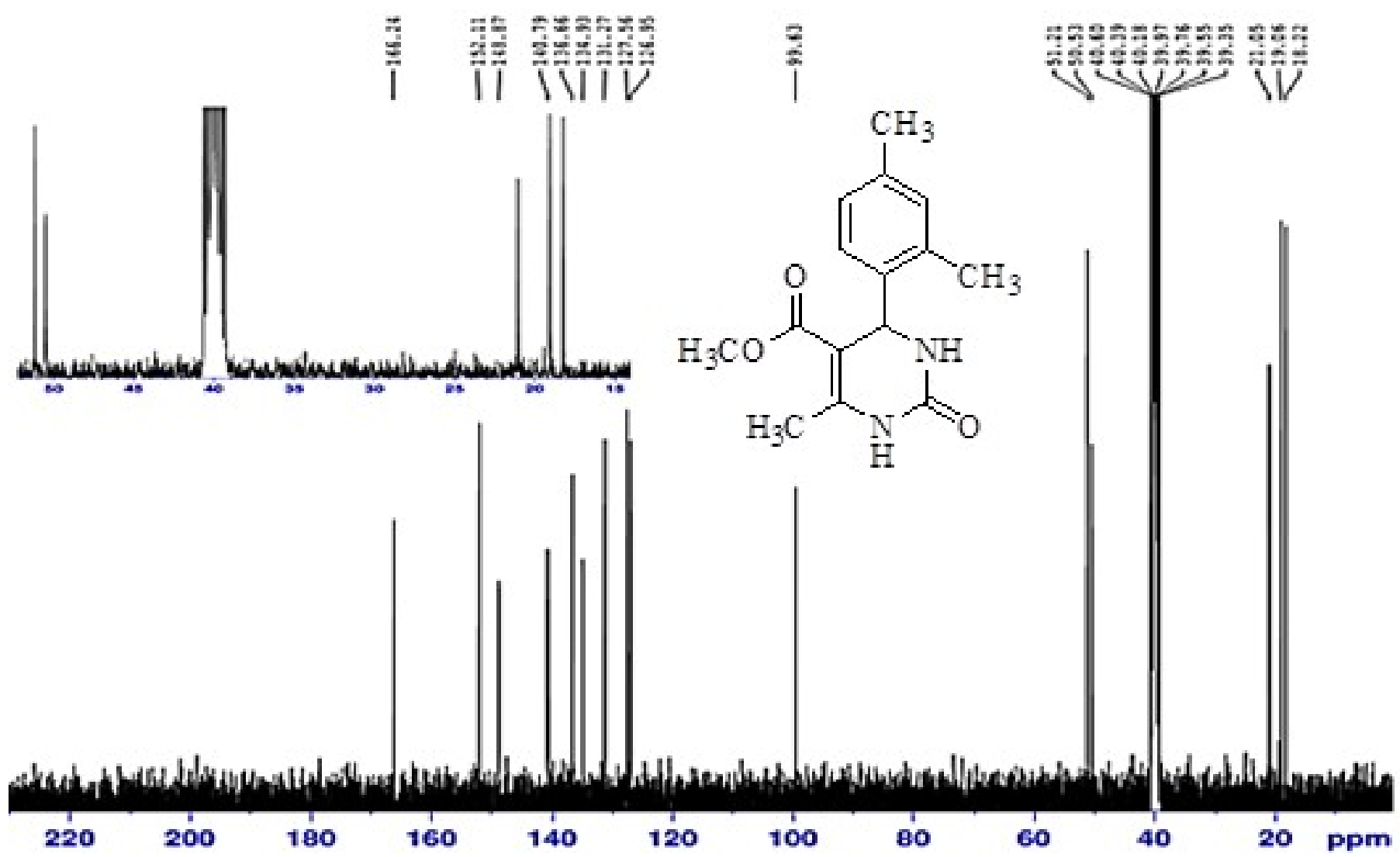

Figure S88. ^{13}C-NMR spectrum of the methyl 4-(2,4-dimethylphenyl)-6-methyl-2-oxo-1,2,3,4-tetrahydropyrimidine-5-carboxylate (**9l**) product in DMSO

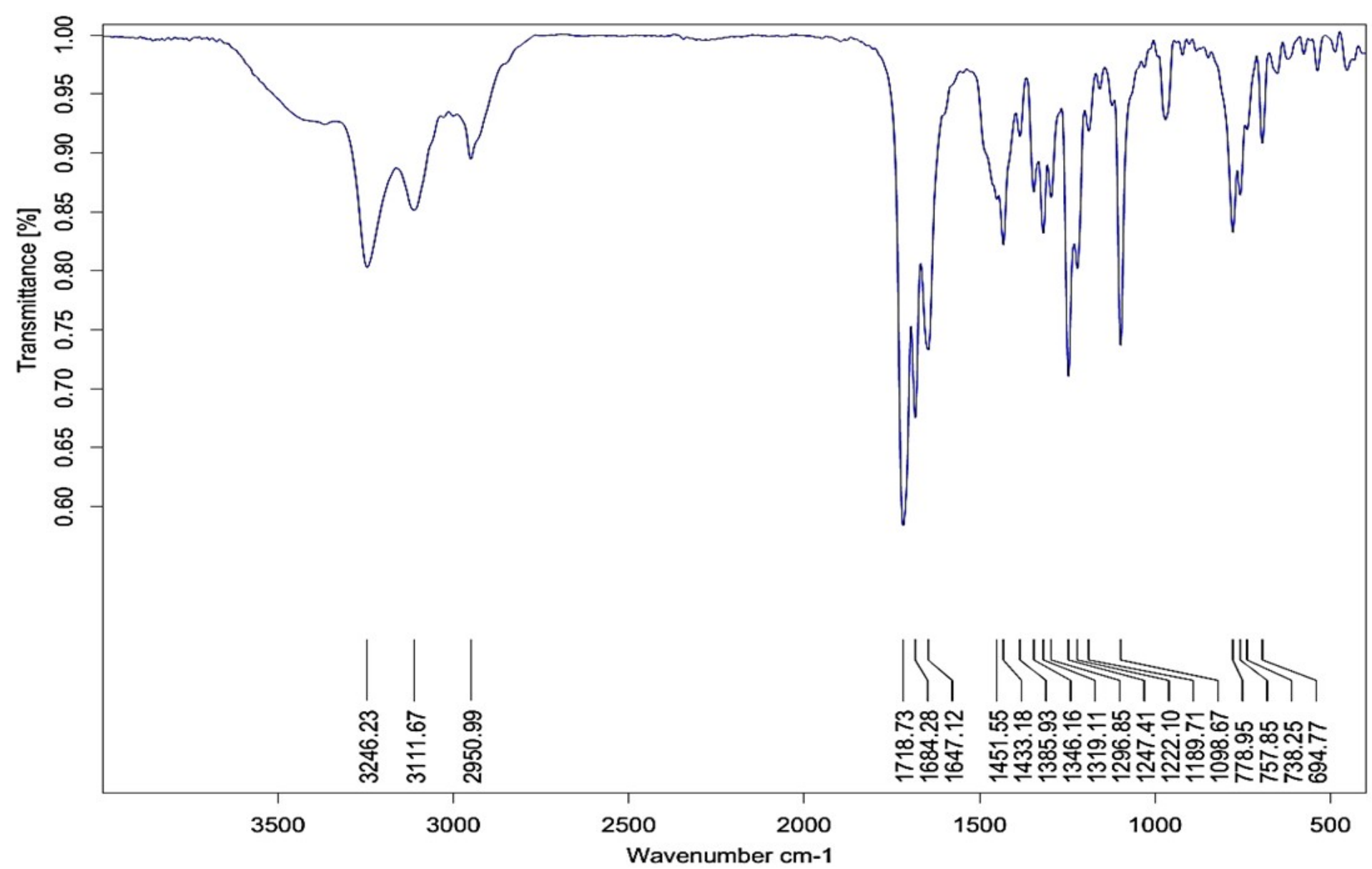

Figure S89. FTIR spectrum of the methyl 6-methyl-2-oxo-4-styryl-1,2,3,4-tetrahydropyrimidine -5-carboxylate (**9m**) product

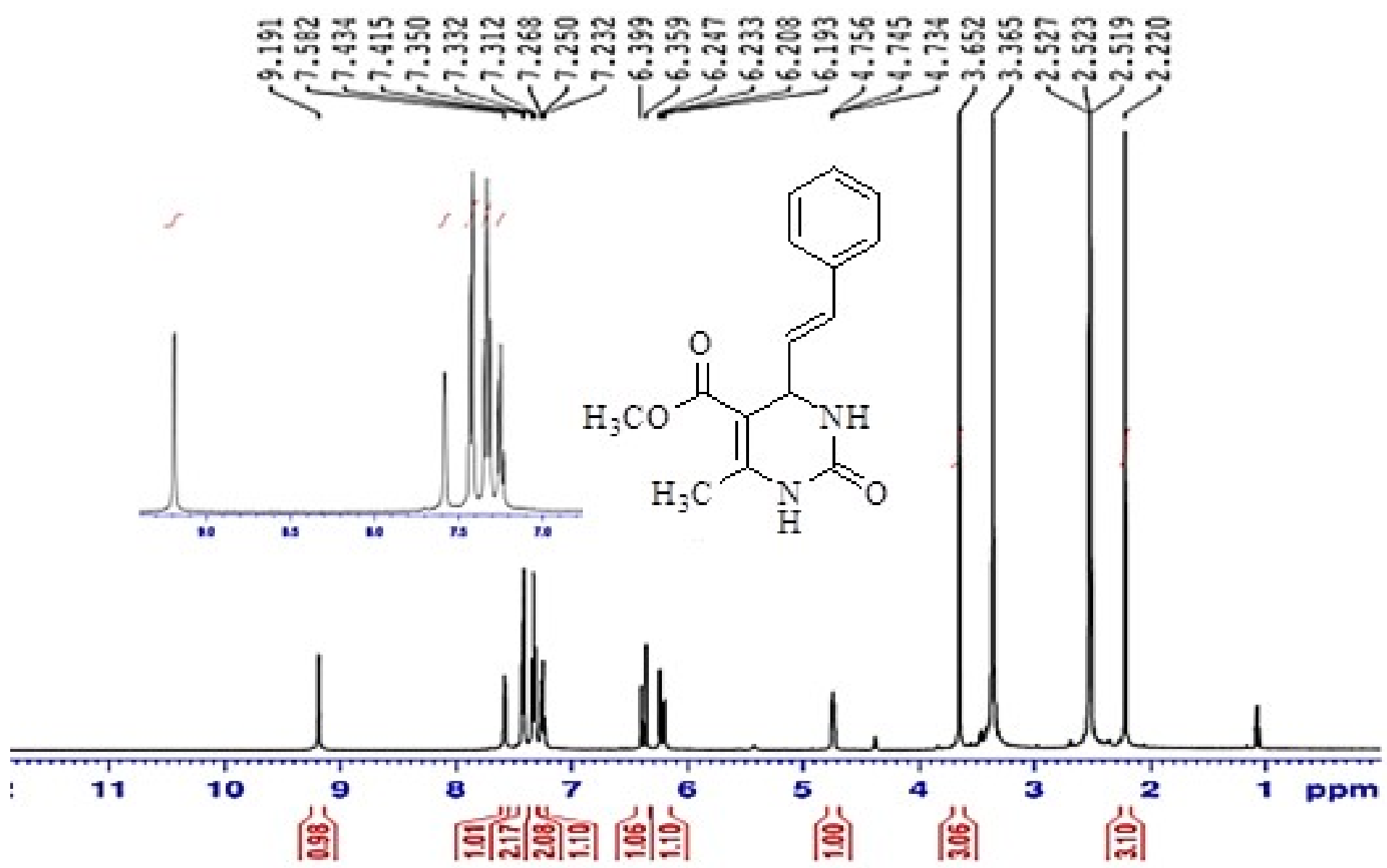

Figure S90. ^{1}H-NMR spectrum of the methyl 6-methyl-2-oxo-4-styryl-1,2,3,4-tetrahydropyrimidine -5-carboxylate (**9m**) product in DMSO

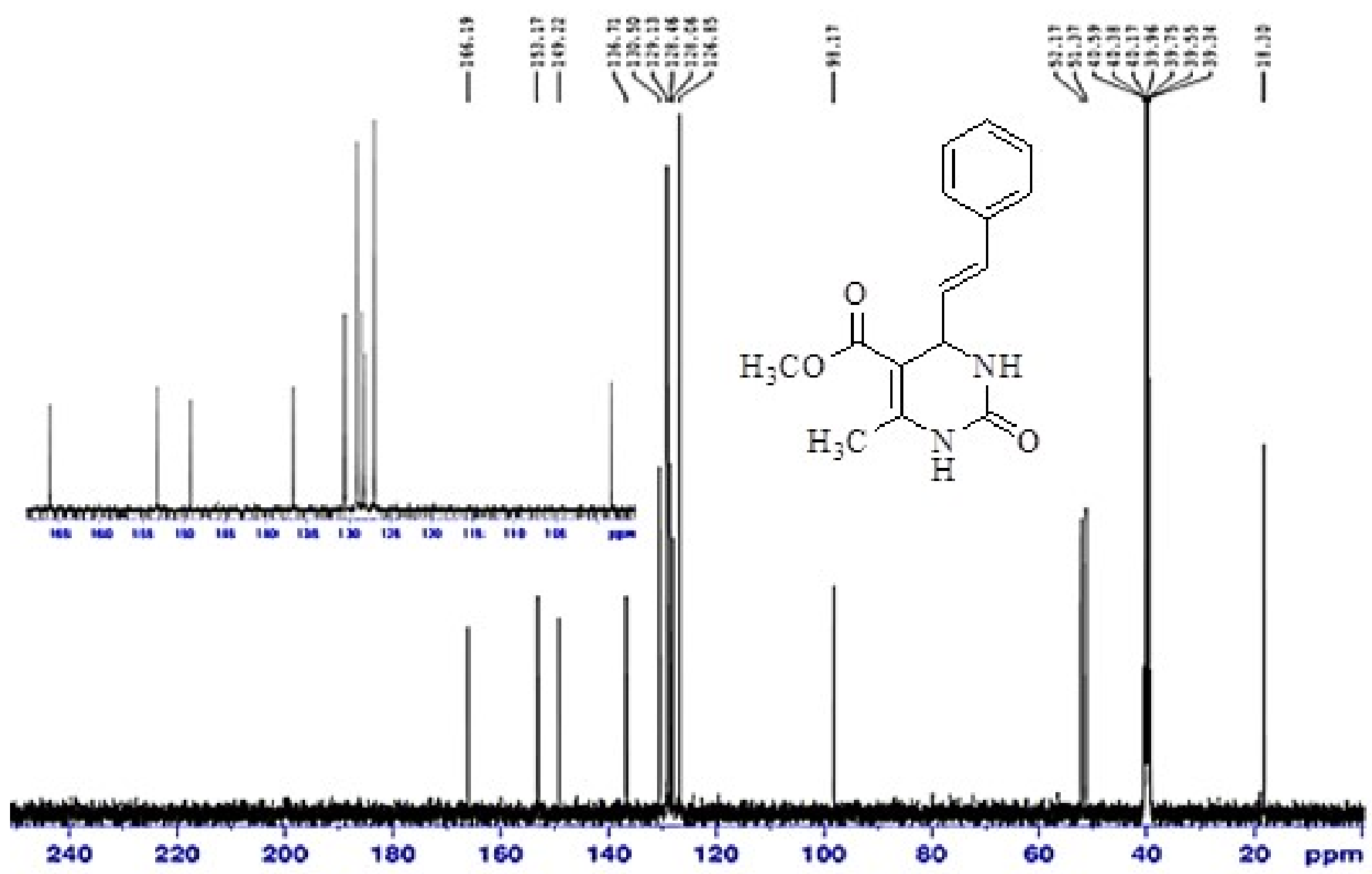

Figure S91. ^{13}C-NMR spectrum of the methyl 6-methyl-2-oxo-4-styryl-1,2,3,4-tetrahydropyrimidine -5-carboxylate (**9m**) product in DMSO

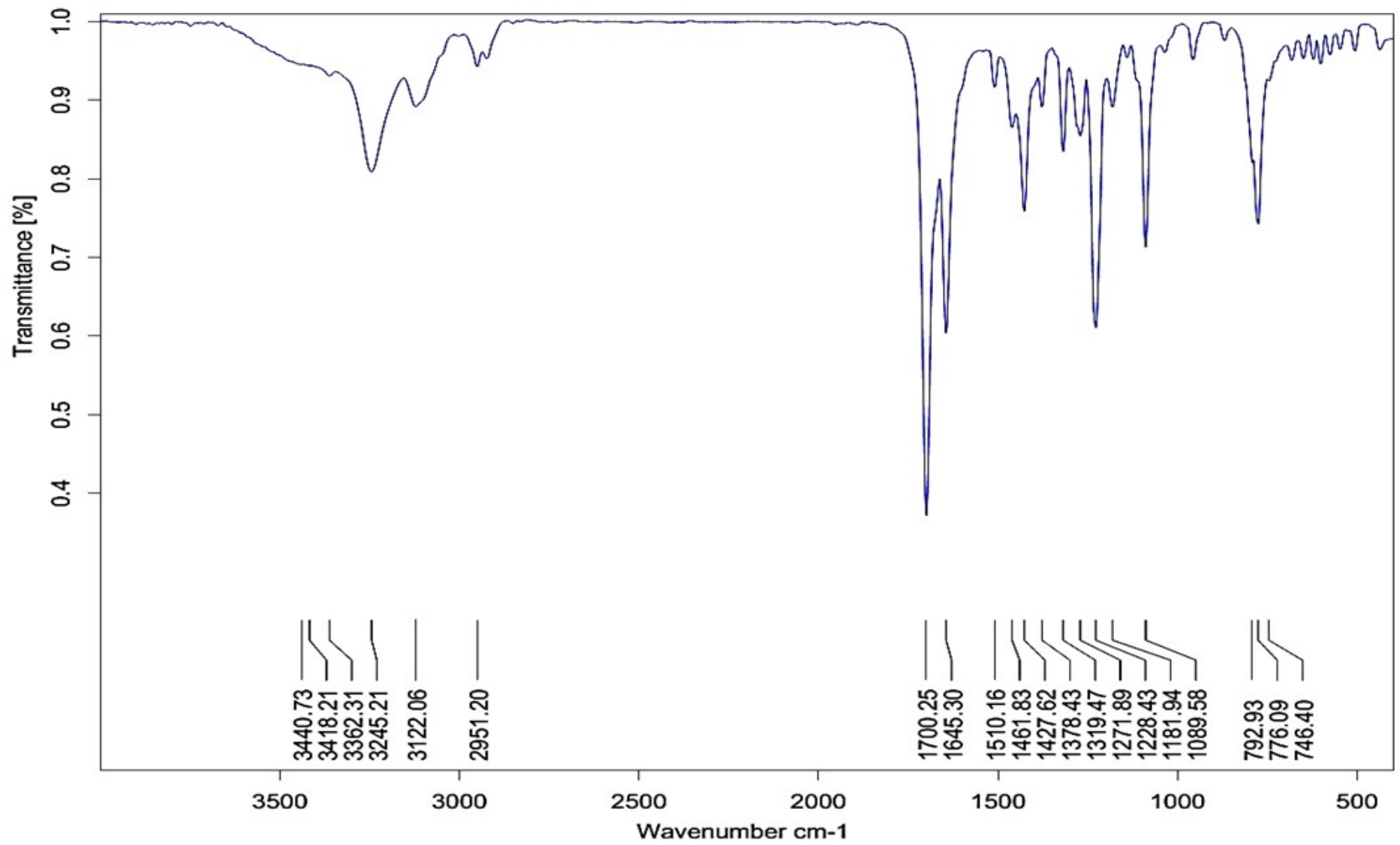

Figure S92. FTIR spectrum of the methyl 6-methyl-4-(naphthalene-1-yl)-2-oxo-1,2,3,4-tetrahydropyrimidine -5-carboxylate (**9n**) product

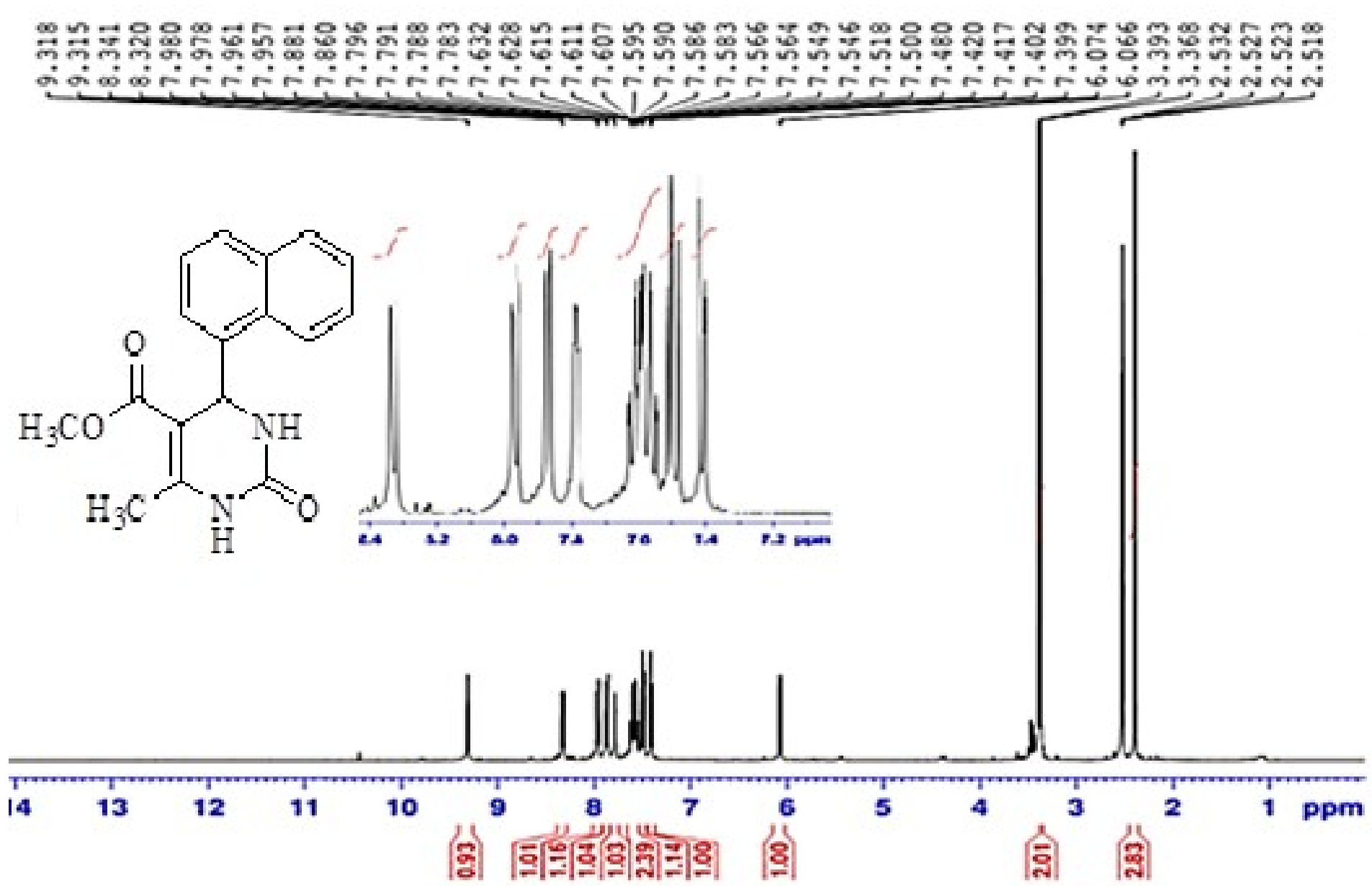

Figure S93. ^{1}H-NMR spectrum of the methyl 6-methyl-4-(naphthalene-1-yl)-2-oxo-1,2,3,4-tetrahydropyrimidine -5-carboxylate (**9n**) product in DMSO

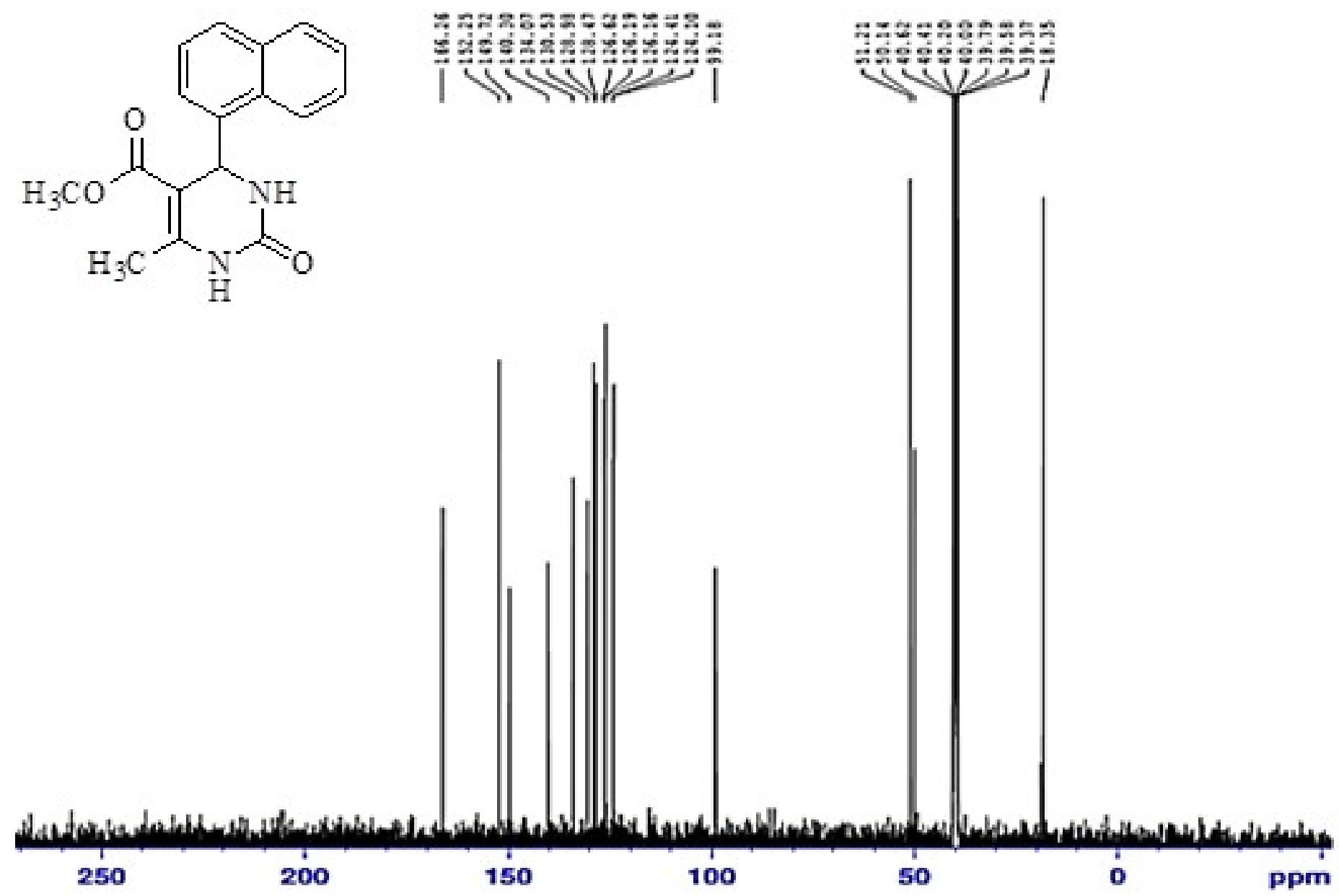

Figure S94. ^{13}C-NMR spectrum of the methyl 6-methyl-4-(naphthalene-1-yl)-2-oxo-1,2,3,4-tetrahydropyrimidine - 5-carboxylate (**9n**) product in DMSO

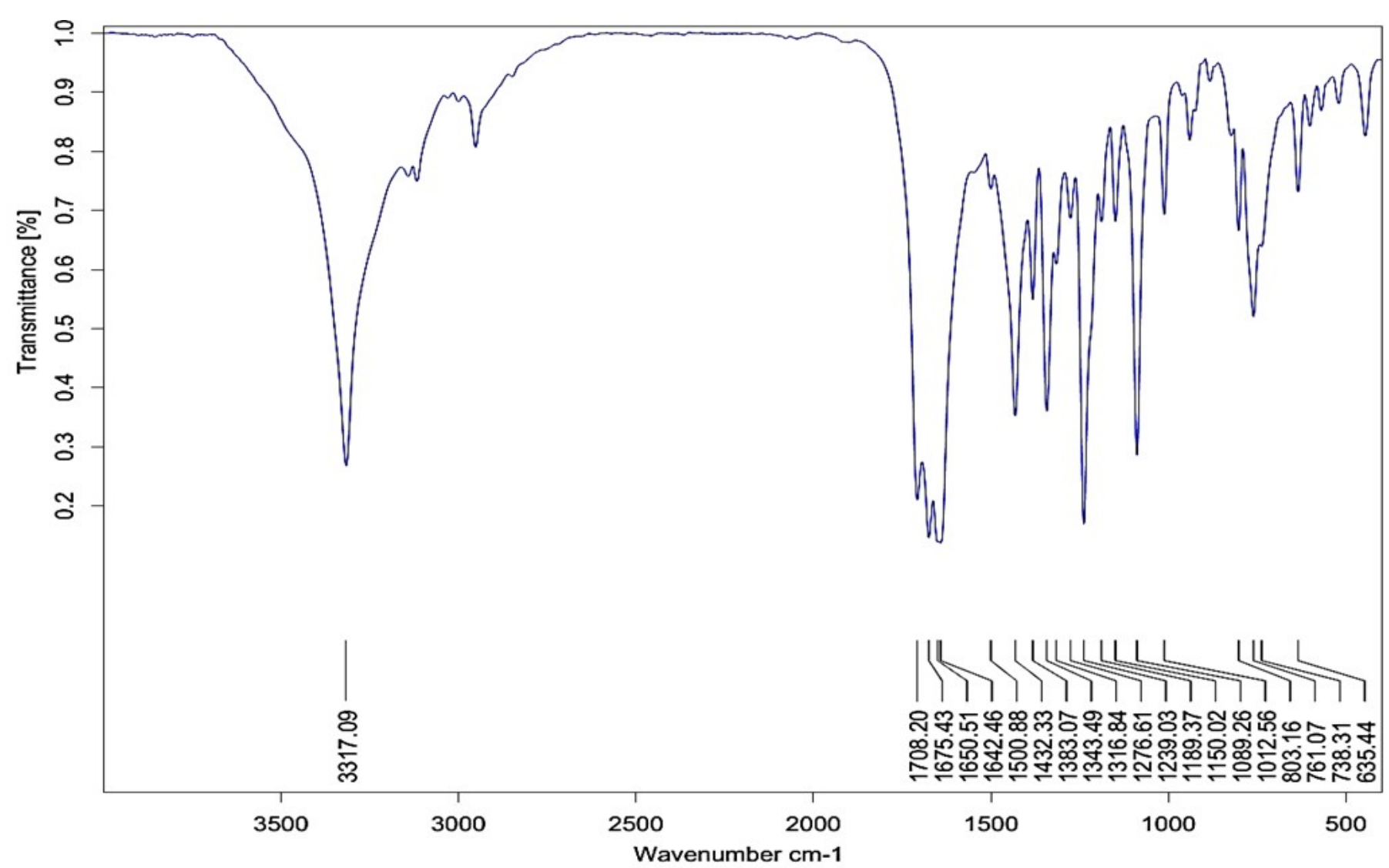

Figure S95. FTIR spectrum of the methyl 4-(4-furan-2-yl)-6-methyl-2-oxo-1,2,3,4-tetrahydropyrimidine-5-carboxylate (**9o**) product

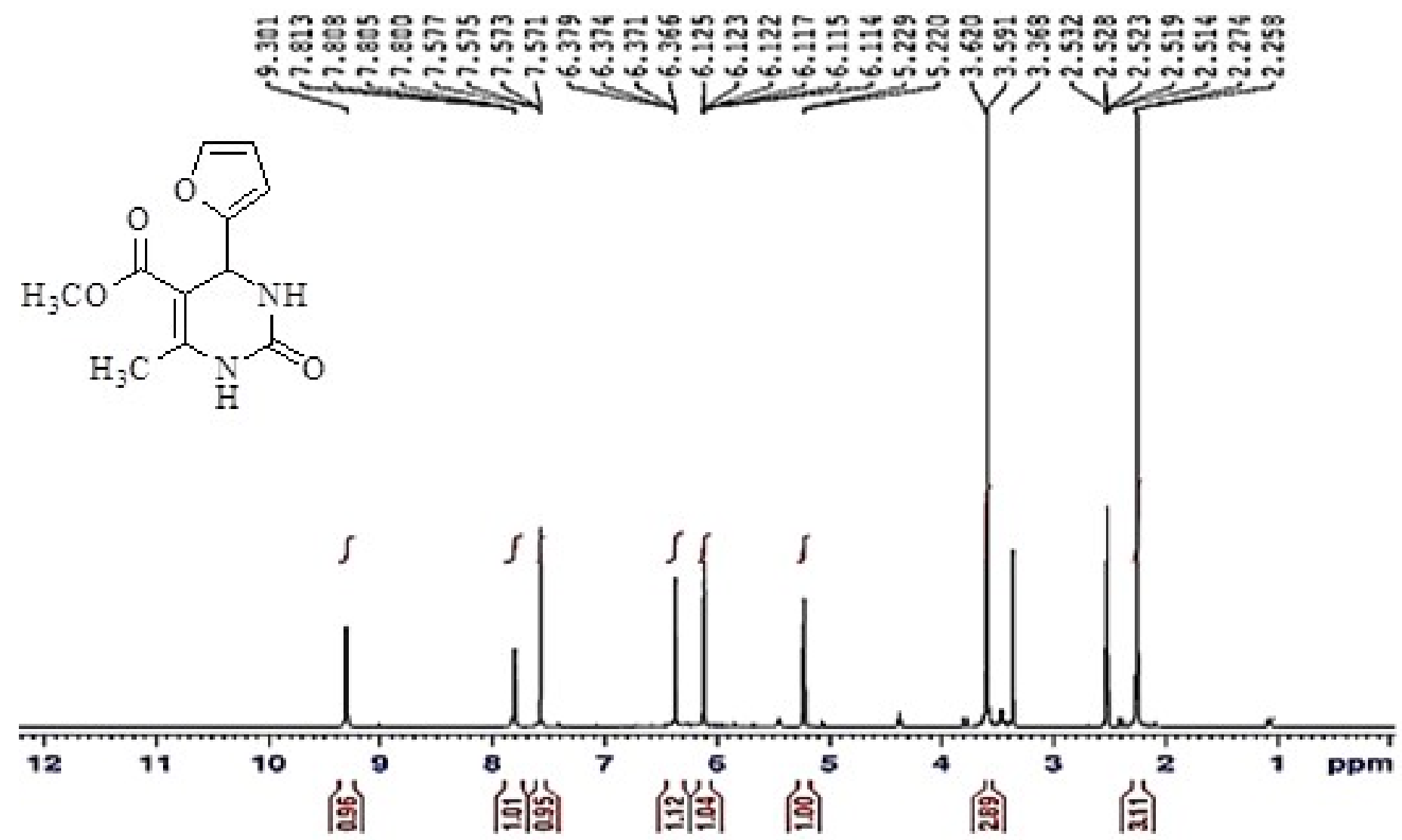

Figure S96. ^{1}H-NMR spectrum of the methyl 4-(4-furan-2-yl)-6-methyl-2-oxo-1,2,3,4-tetrahydropyrimidine-5-carboxylate (**9o**) produc**t** in DMSO

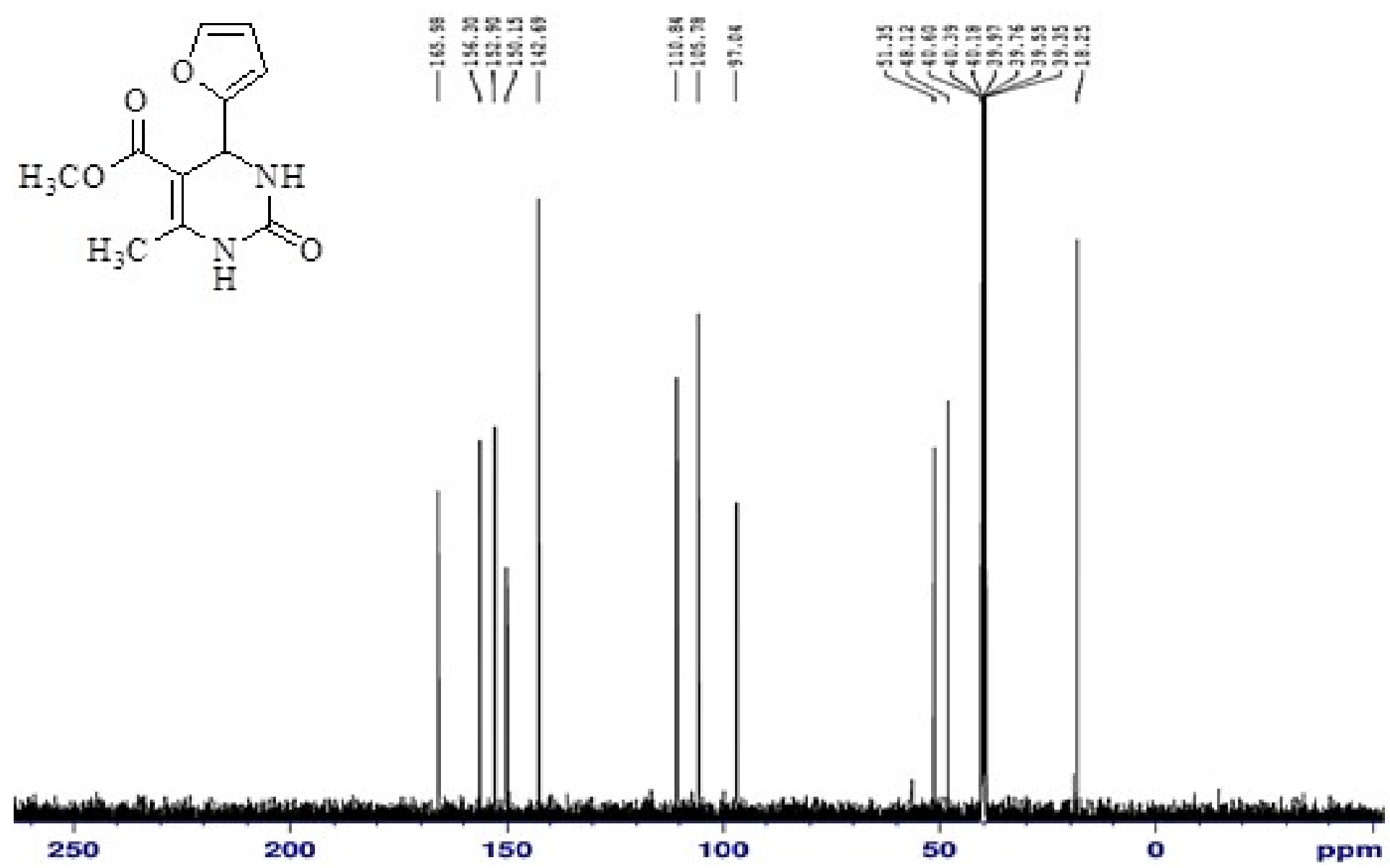

Figure S97. ^{13}C-NMR spectrum of the methyl 4-(4-furan-2-yl)-6-methyl-2-oxo-1,2,3,4-tetrahydropyrimidine-5-carboxylate (**9o**) produc**t** in DMSO

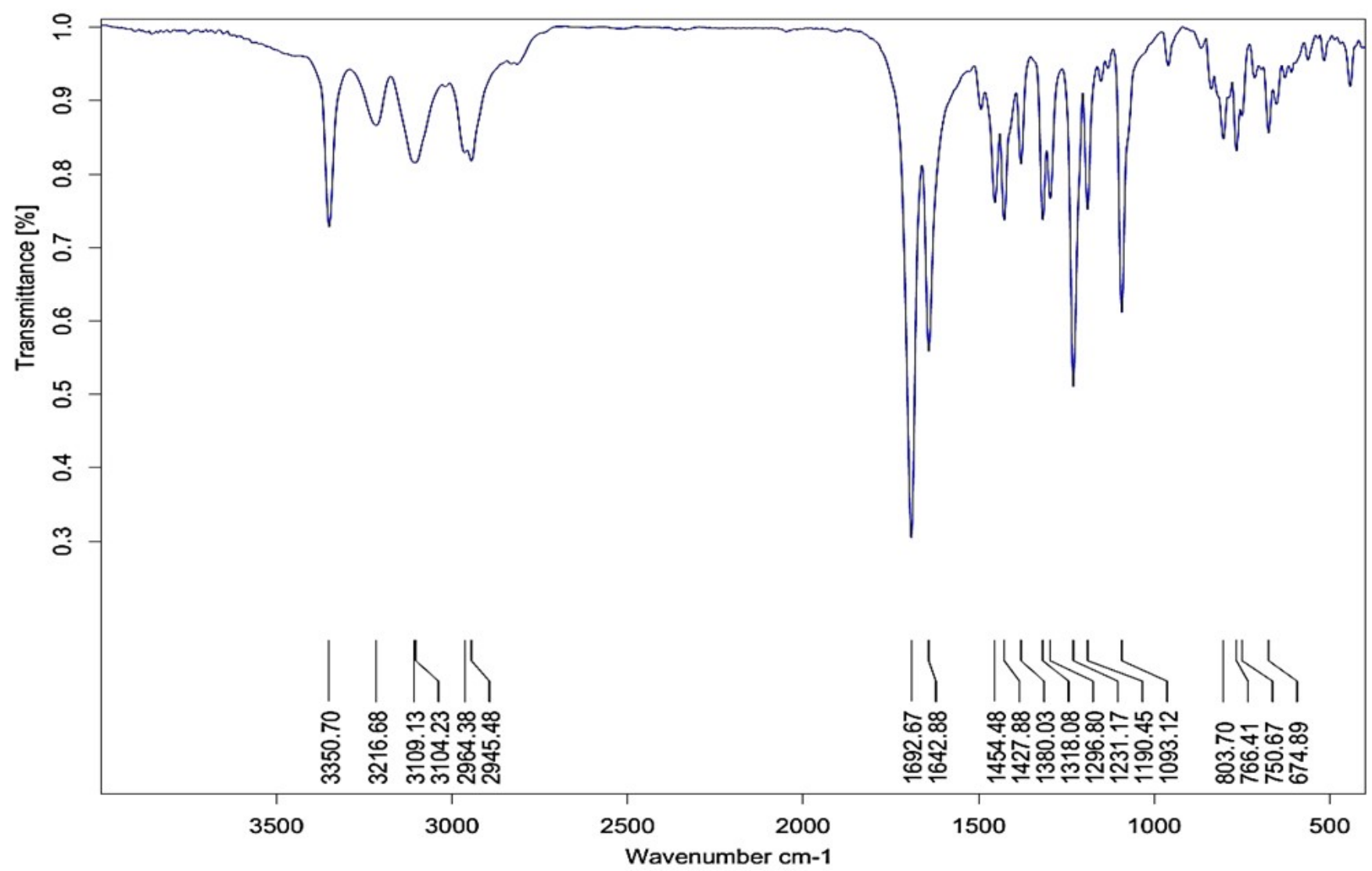

Figure S98. FTIR spectrum of the methyl 4-(4-thiol-3-yl)-6-methyl-2-oxo-1,2,3,4-tetrahydropyrimidine-5-carboxylate (**9p**) product

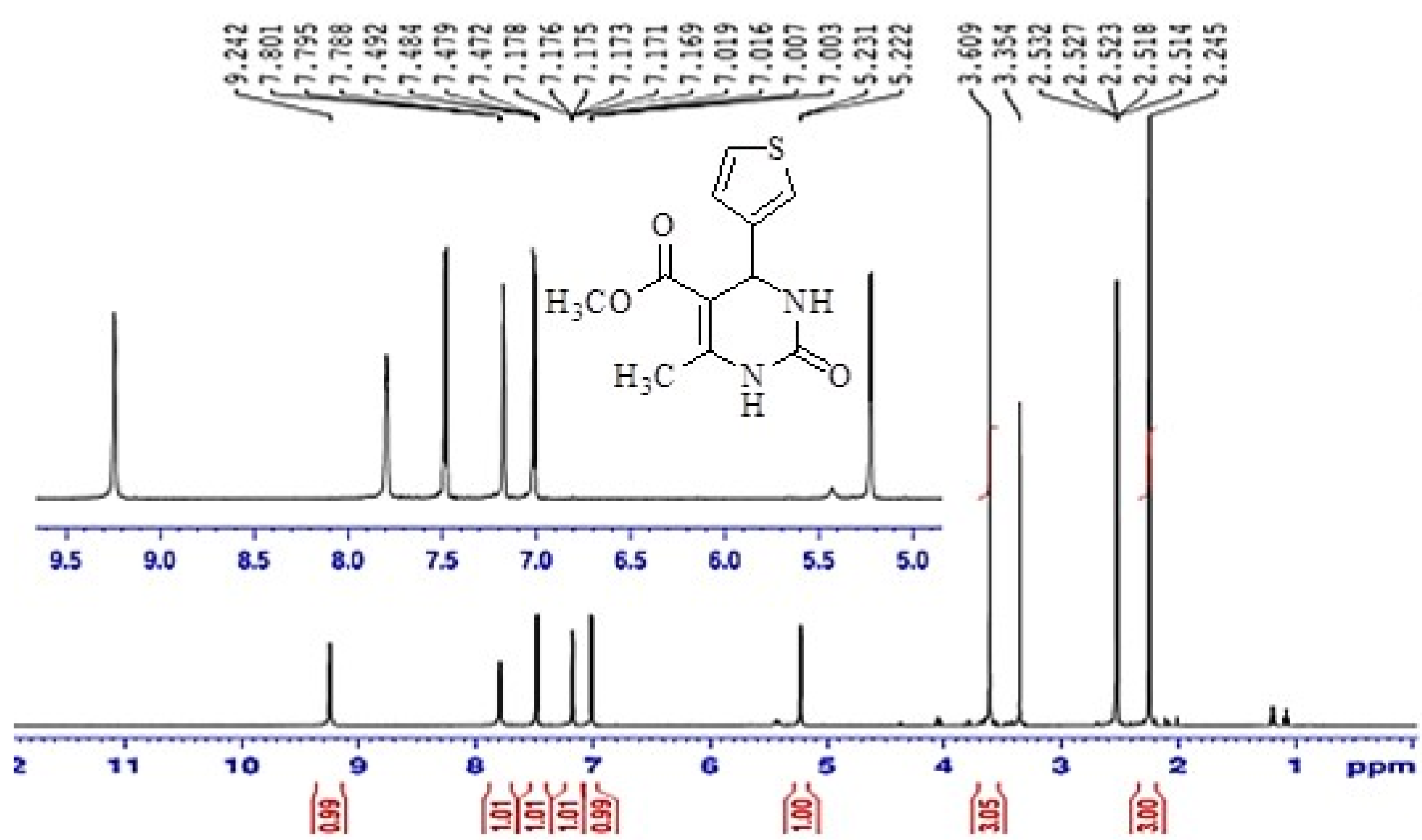

Figure S99. ^{1}H-NMR spectrum of the methyl 4-(4-thiol-3-yl)-6-methyl-2-oxo-1,2,3,4-tetrahydropyrimidine -5-carboxylate (**9p**) product in DMSO

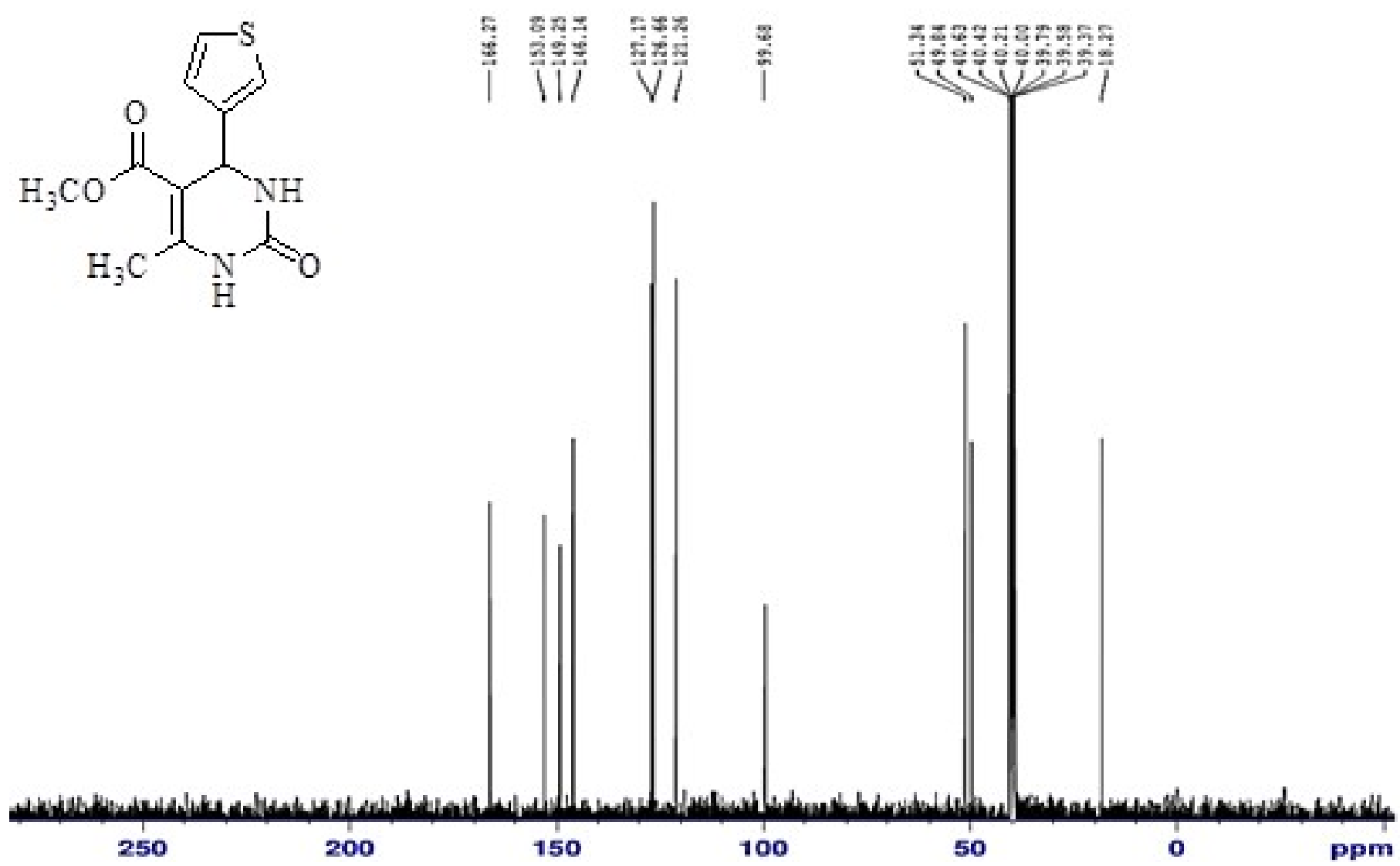

Figure S100. ^{13}C-NMR spectrum of the methyl 4-(4-thiol-3-yl)-6-methyl-2-oxo-1,2,3,4-tetrahydropyrimidine -5-carboxylate (**9p**) product in DMSO

GC calibration curves

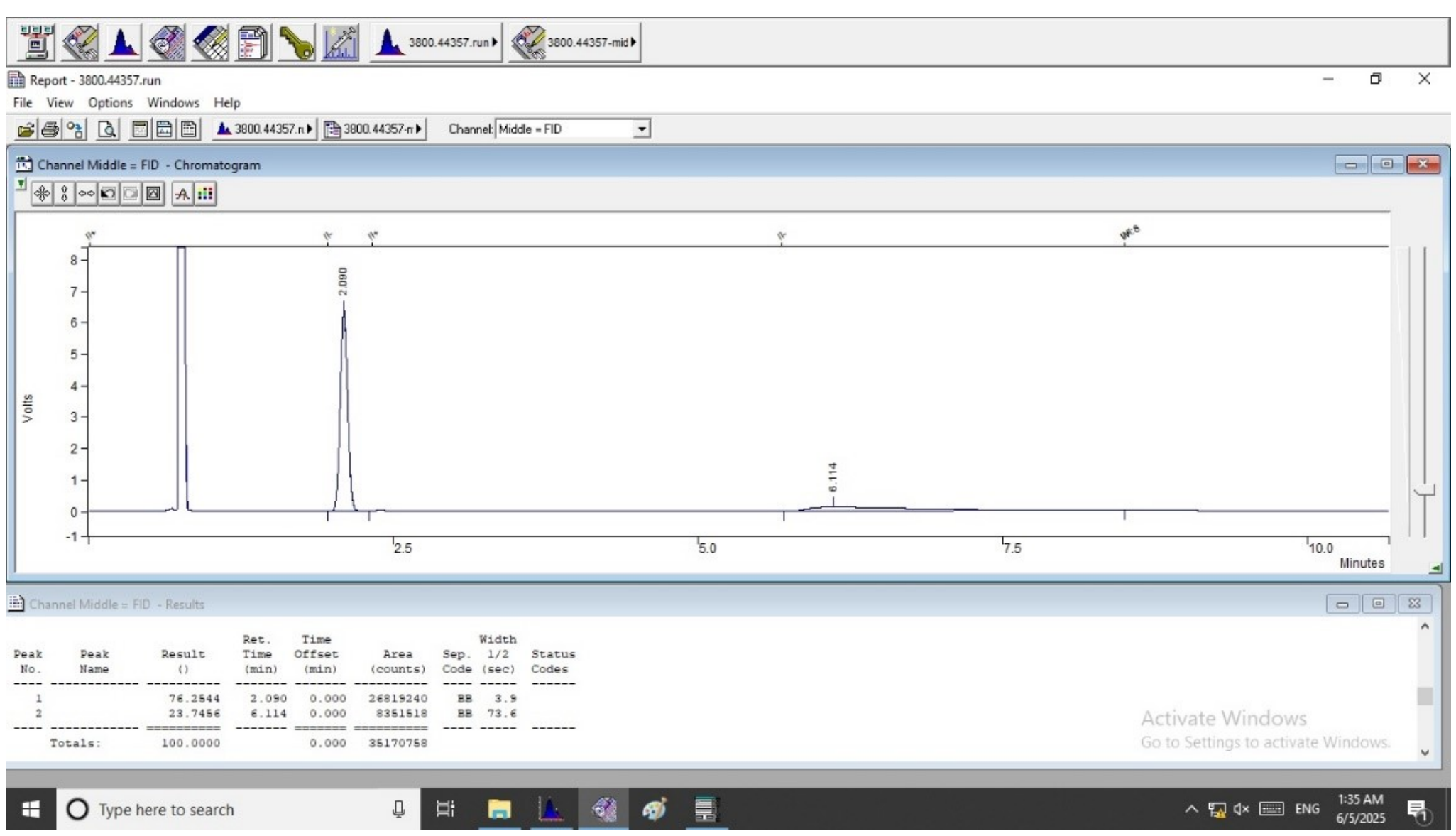

Peak No.	Peak Name	Result ()	Ret. Time (min)	Time Offset (min)	Area (counts)	Sep. Code	Width 1/2 (sec)	Status Codes
1		76.2544	2.090	0.000	26819240	BB	3.9	
2		23.7456	6.114	0.000	8351518	BB	73.6	
	Totals:	100.0000		0.000	35170758			

Figure S101. Gas chromatogram of the solution containing 0.25 mmol benzyl alcohol and 0.75 mmol TMB-first injection

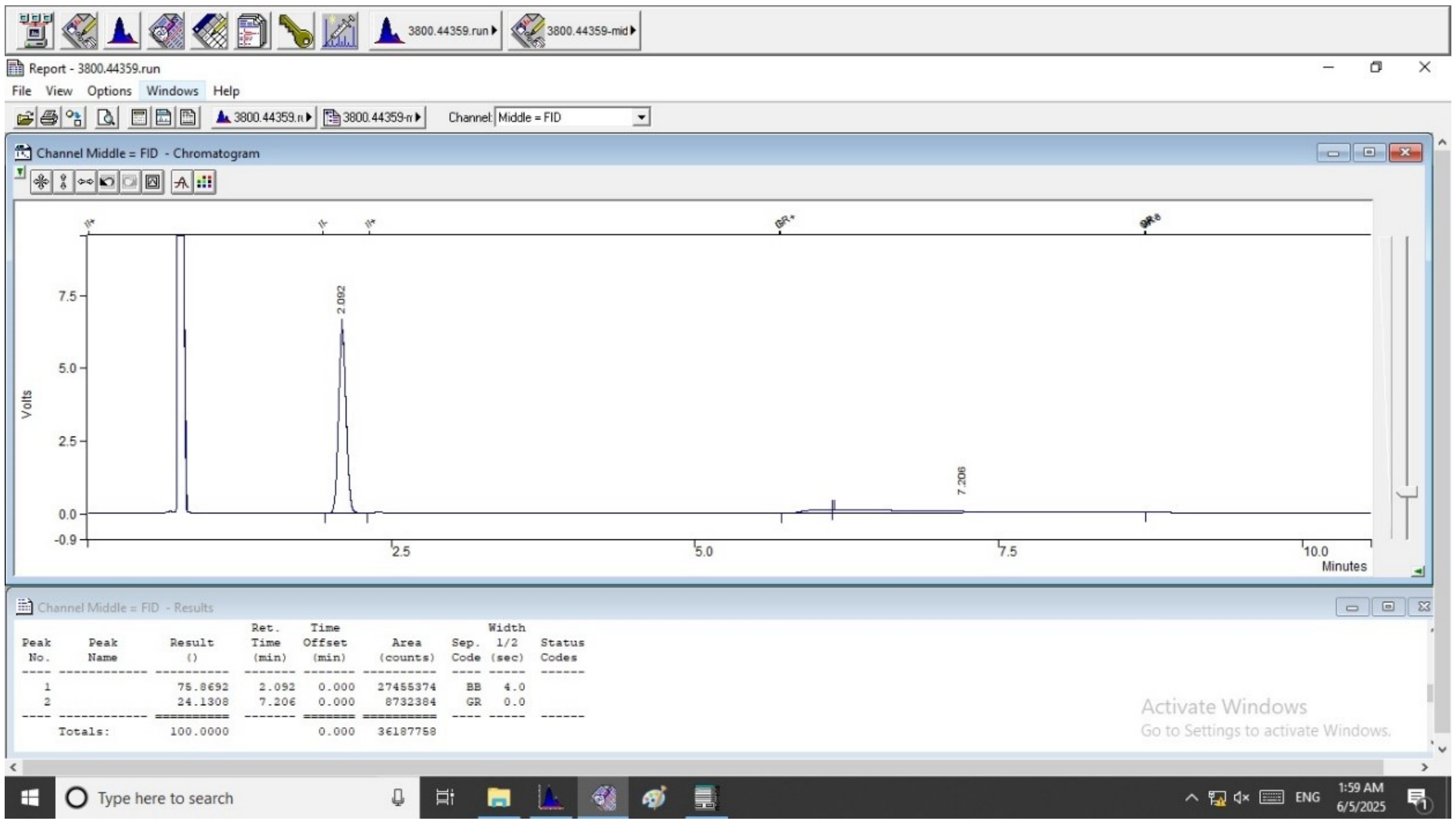

Figure S102. Gas chromatogram of the solution containing 0.25 mmol benzyl alcohol and 0.75 mmol TMB-second injection

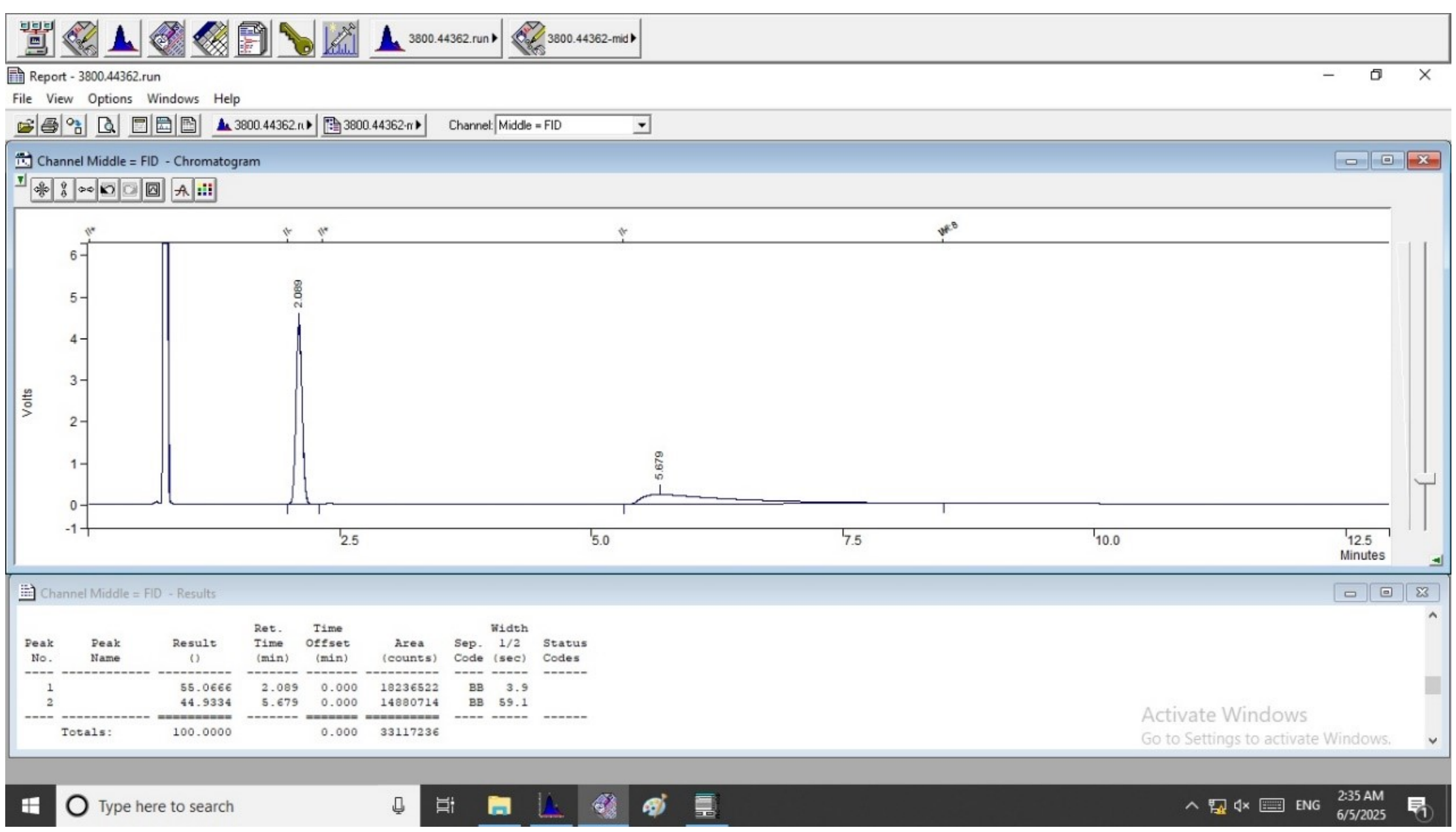

Figure S103. Gas chromatogram of the solution containing 0.5 mmol benzyl alcohol and 0.5 mmol TMB-first injection

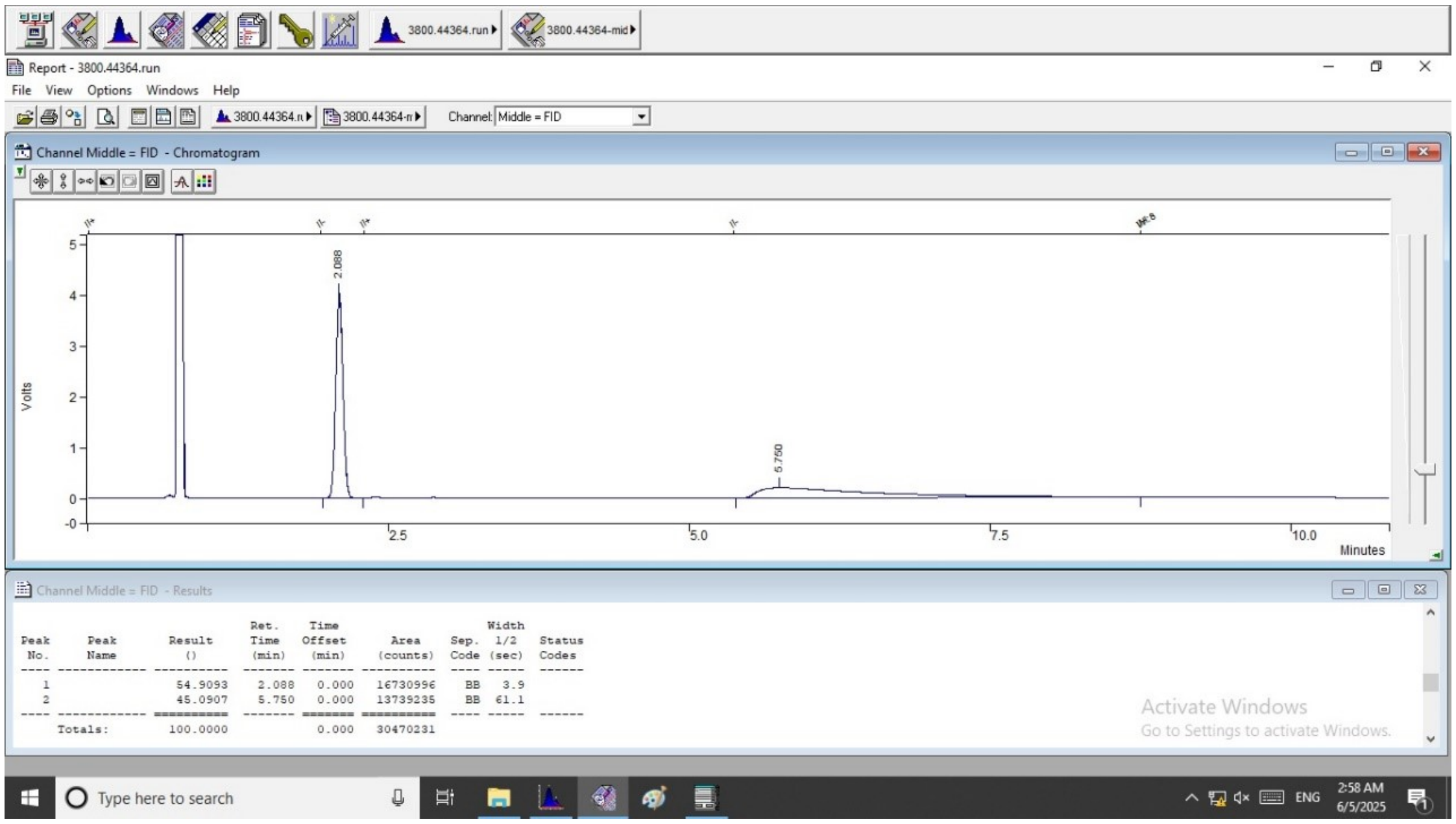

Figure S104. Gas chromatogram of the solution containing 0.5 mmol benzyl alcohol and 0.5 mmol TMB-second injection

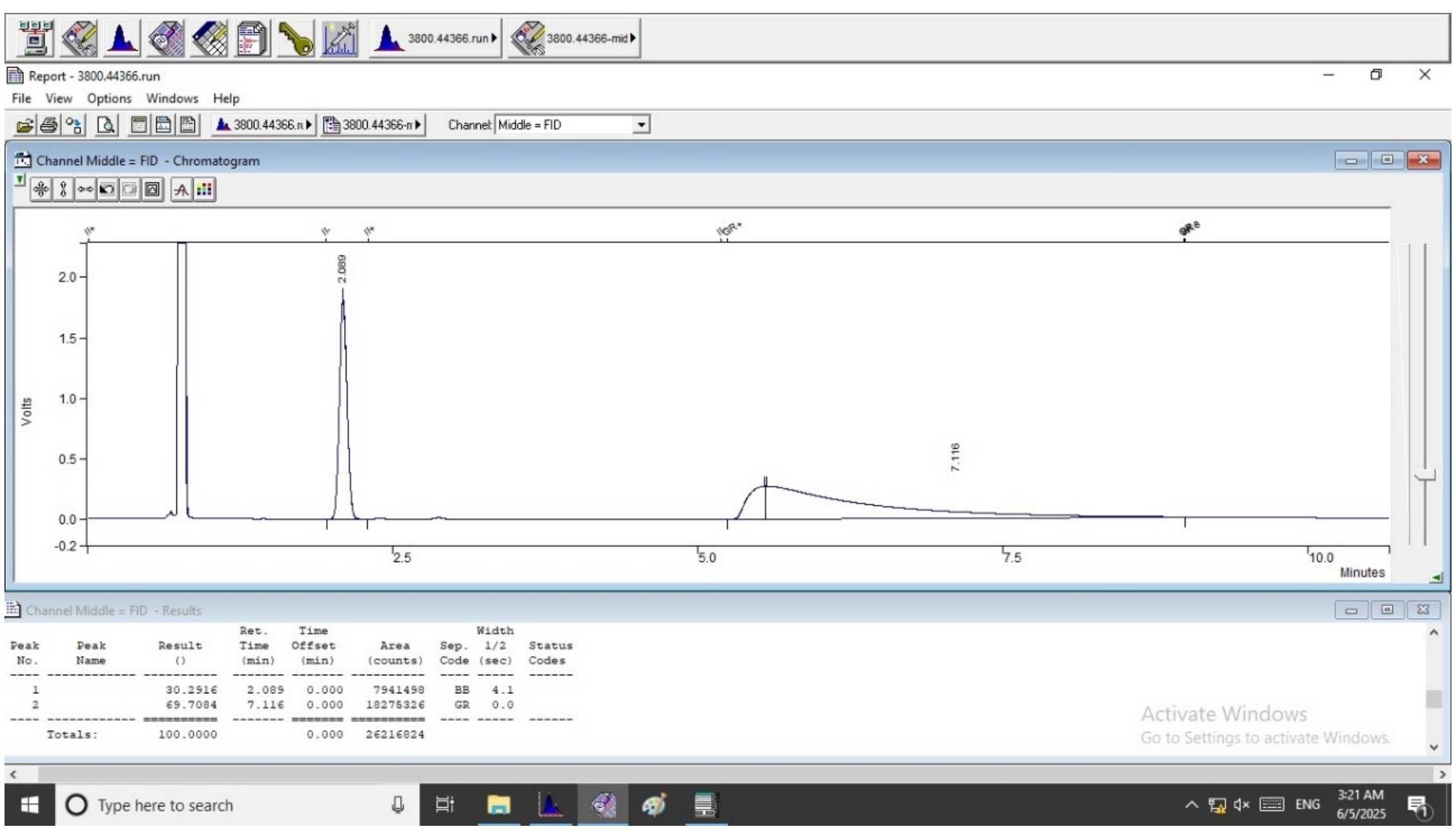

Figure S105. Gas chromatogram of the solution containing 0.75 mmol benzyl alcohol and 0.25 mmol TMB-first injection

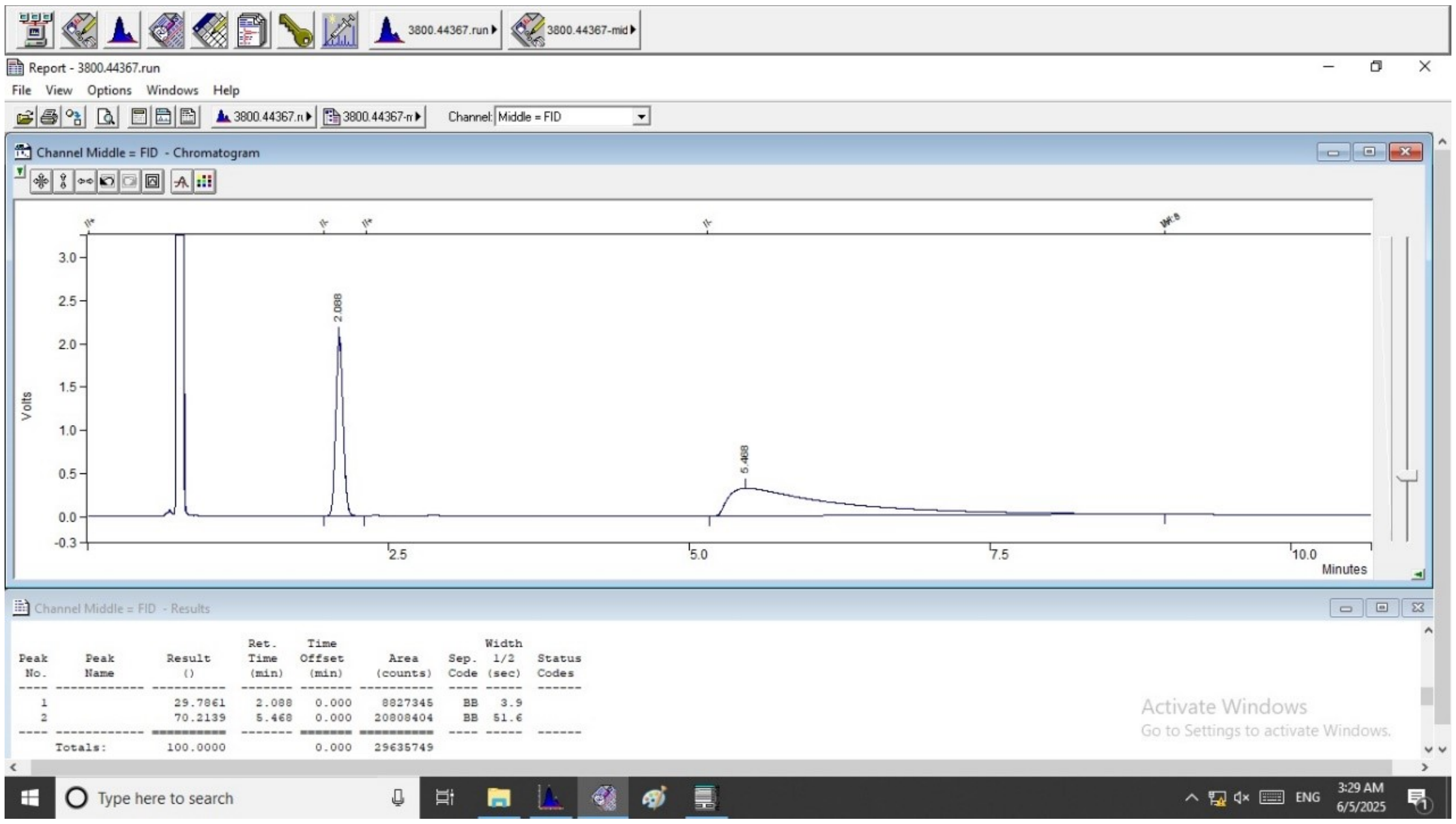

Figure S106. Gas chromatogram of the solution containing 0.75 mmol benzyl alcohol and 0.25 mmol TMB-second injection

Table S4. Summary of GC results

	X (Theoretical)	X first injection	X second injection	average
TMB	0.75	0.763	0.759	0.761
Benzyl alcohol	0.25	0.237	0.241	0.239
TMB	0.5	0.551	0.549	0.55
Benzyl alcohol	0.5	0.449	0.451	0.45
TMB	0.25	0.303	0.298	0.3
Benzyl alcohol	0.75	0.697	0.702	0.699

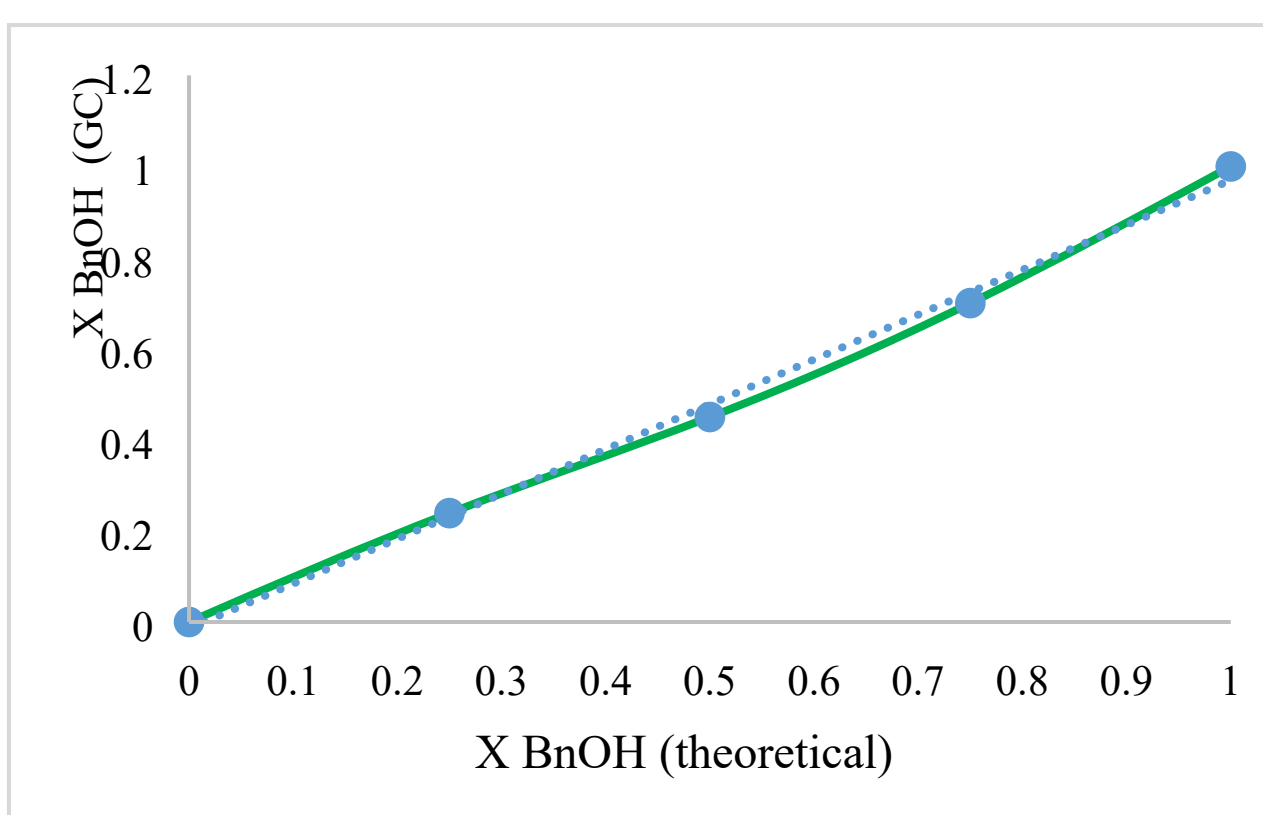

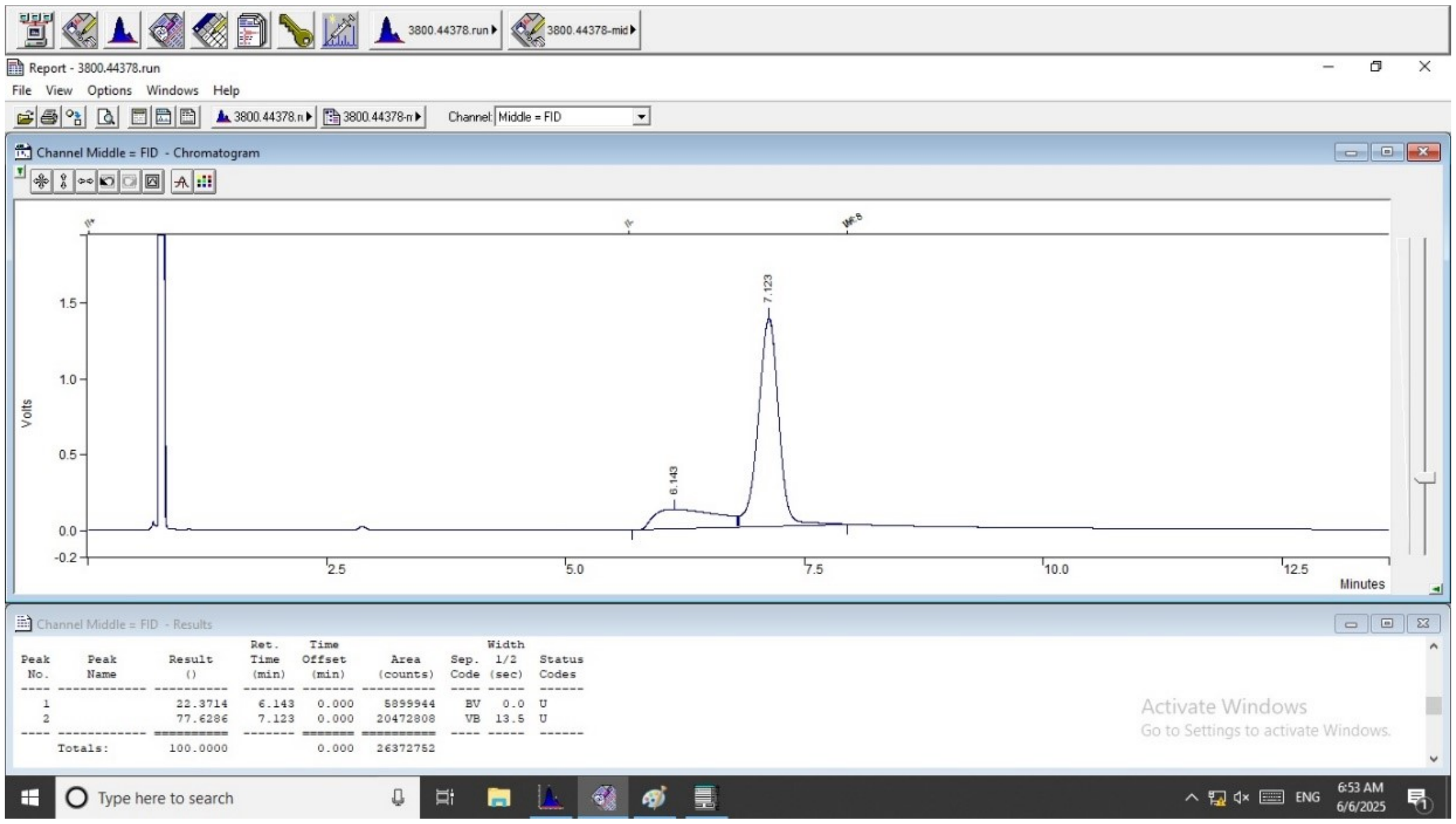

Figure S107. Gas chromatogram of the solution containing 0.25 mmol benzyl alcohol and 0.75 mmol benzyl acetate-first injection

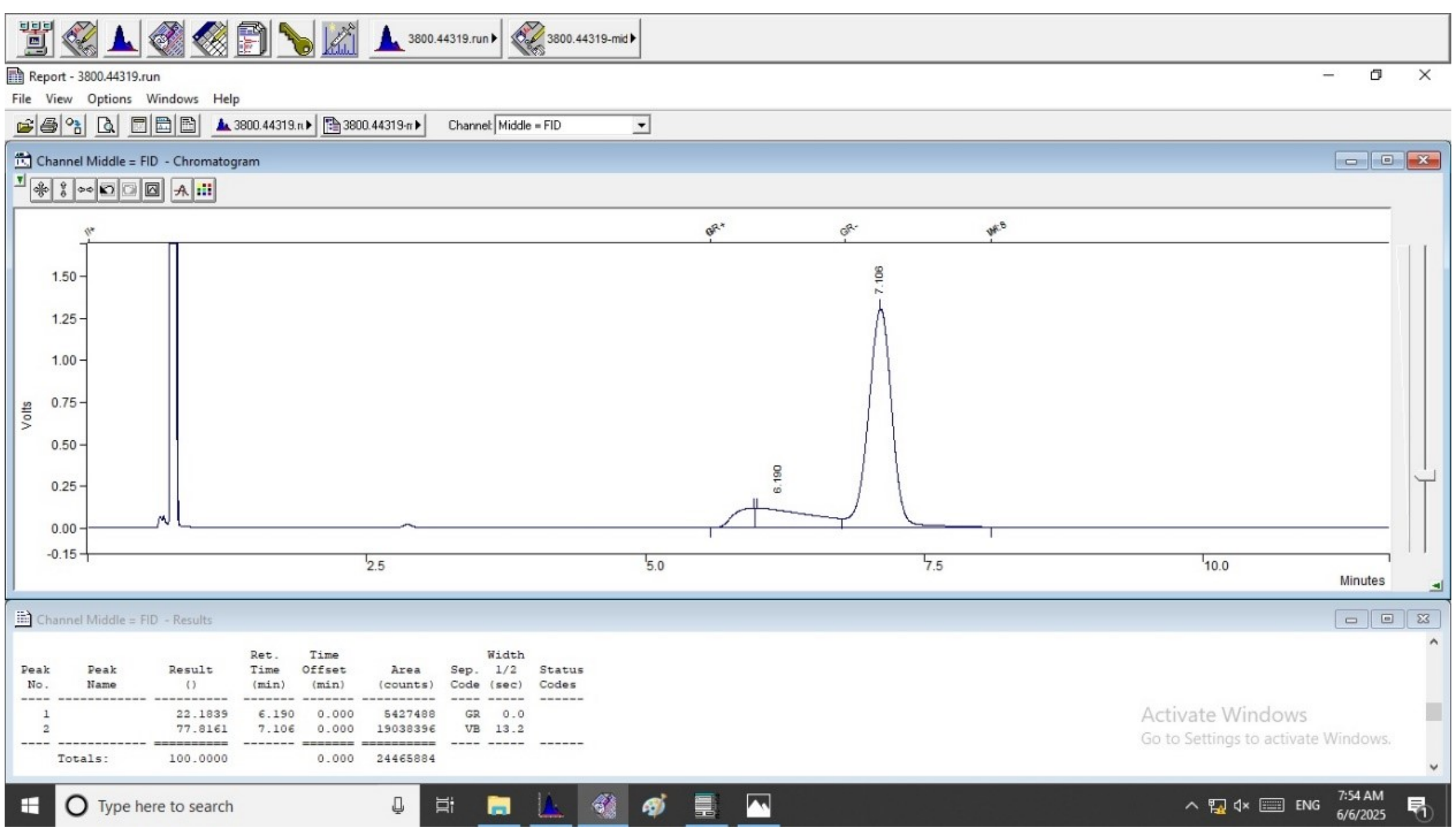

Figure S108. Gas chromatogram of the solution containing 0.25 mmol benzyl alcohol and 0.75 mmol benzyl acetate-second injection

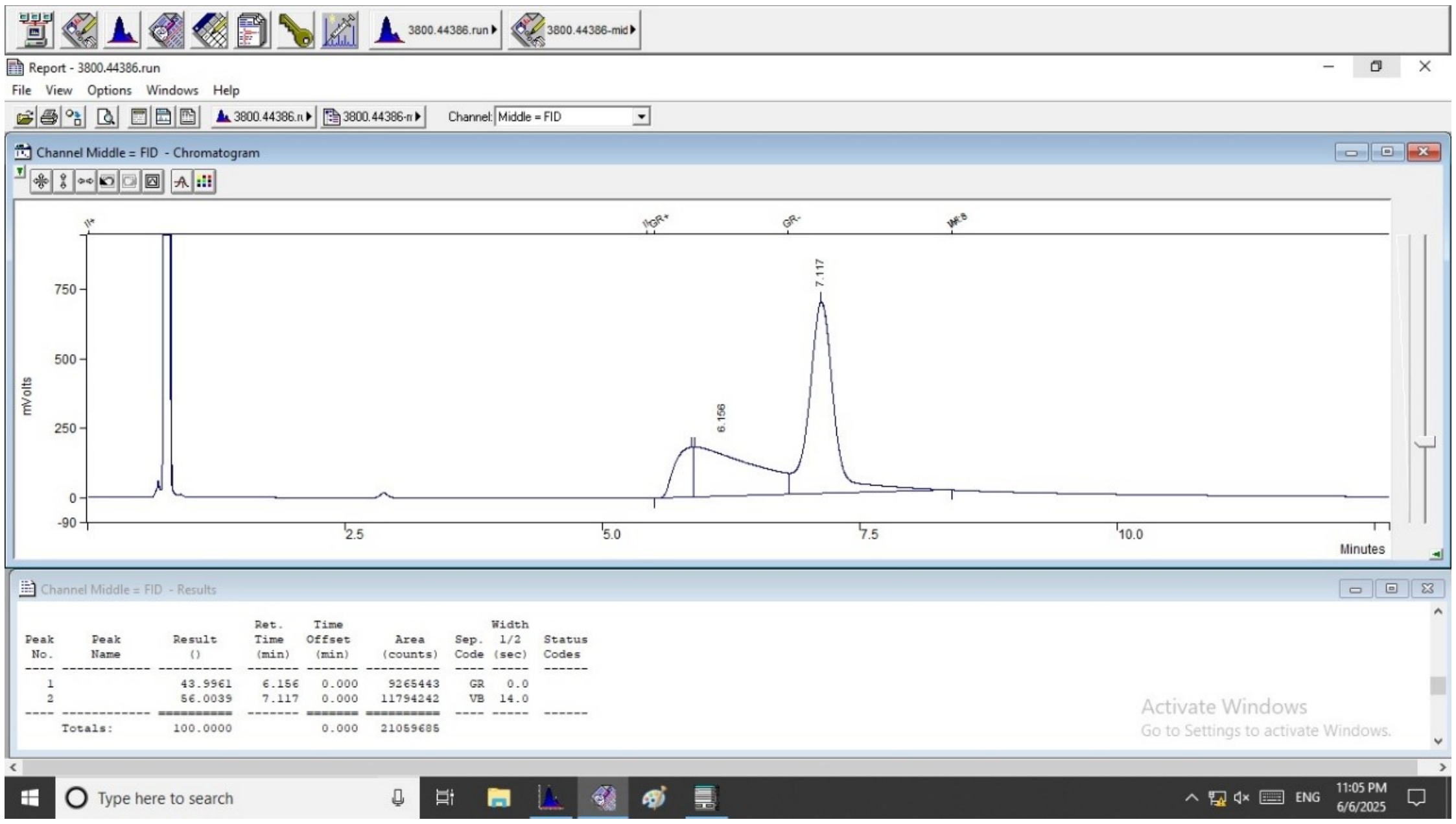

Figure S109. Gas chromatogram of the solution containing 0.5 mmol benzyl alcohol and 0.5 mmol benzyl acetate-first injection

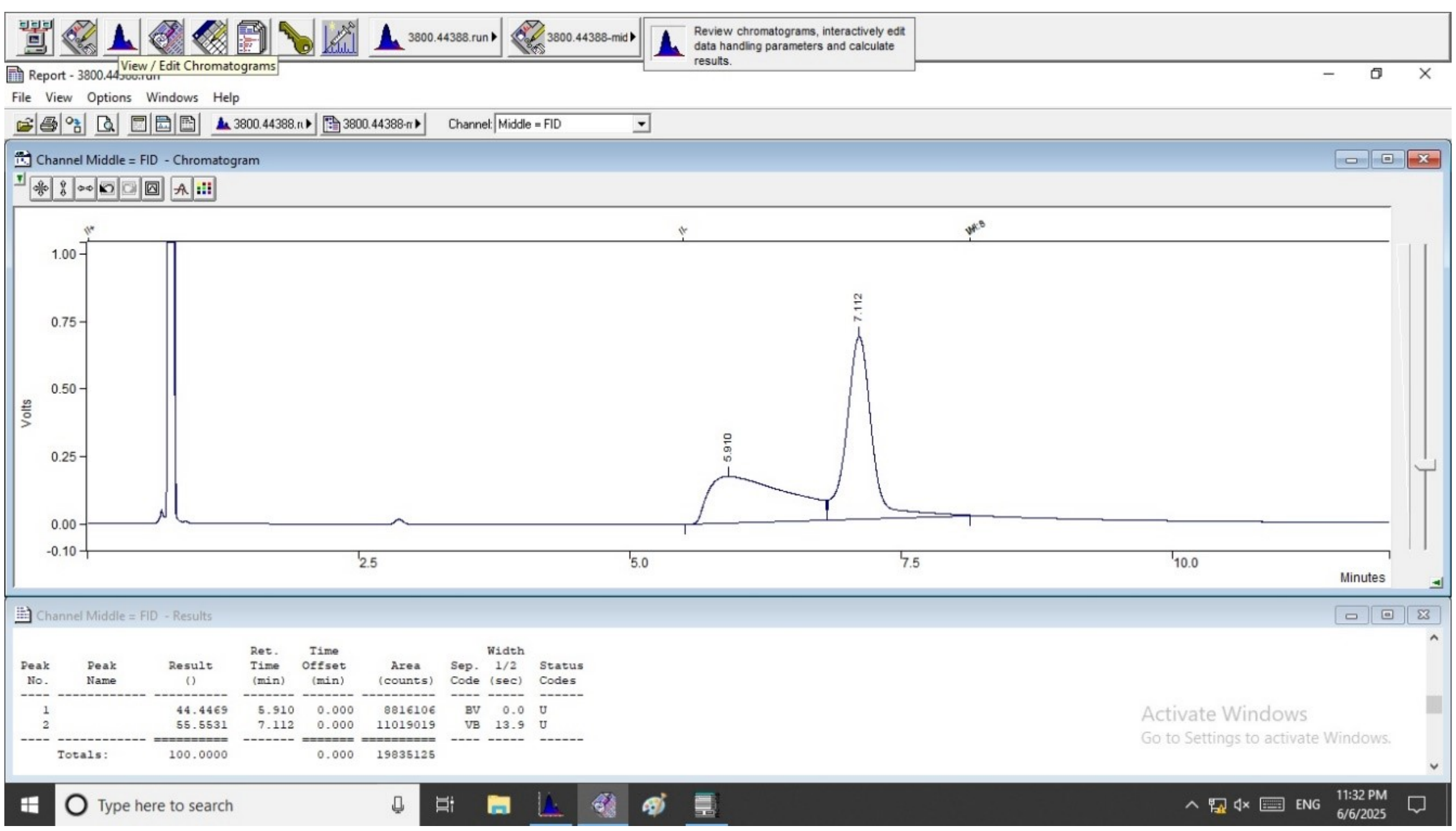

Figure S110. Gas chromatogram of the solution containing 0.5 mmol benzyl alcohol and 0.5 mmol benzyl acetate-second injection

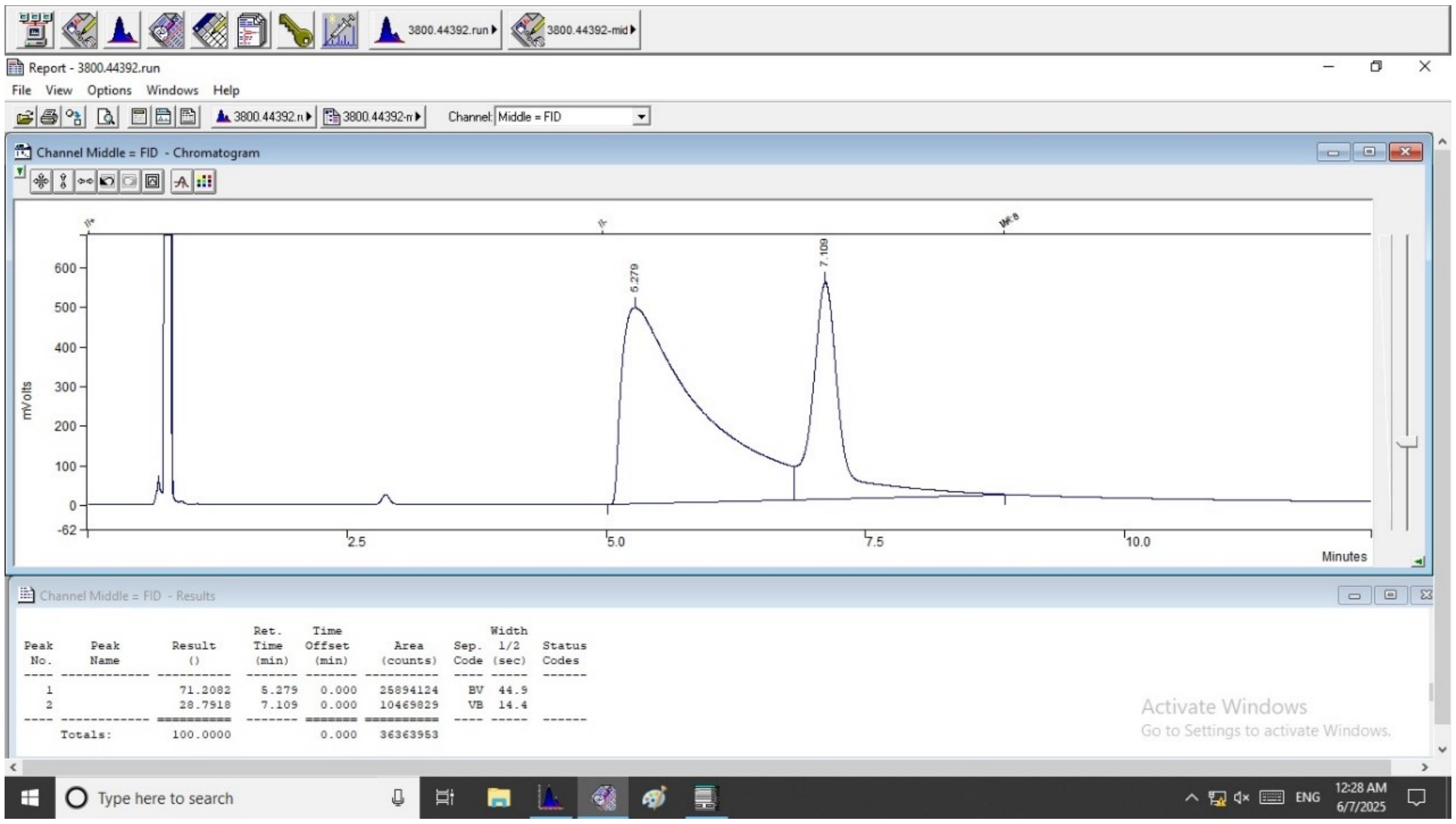

Figure S111. Gas chromatogram of the solution containing 0.75 mmol benzyl alcohol and 0.25 mmol benzyl acetate-first injection

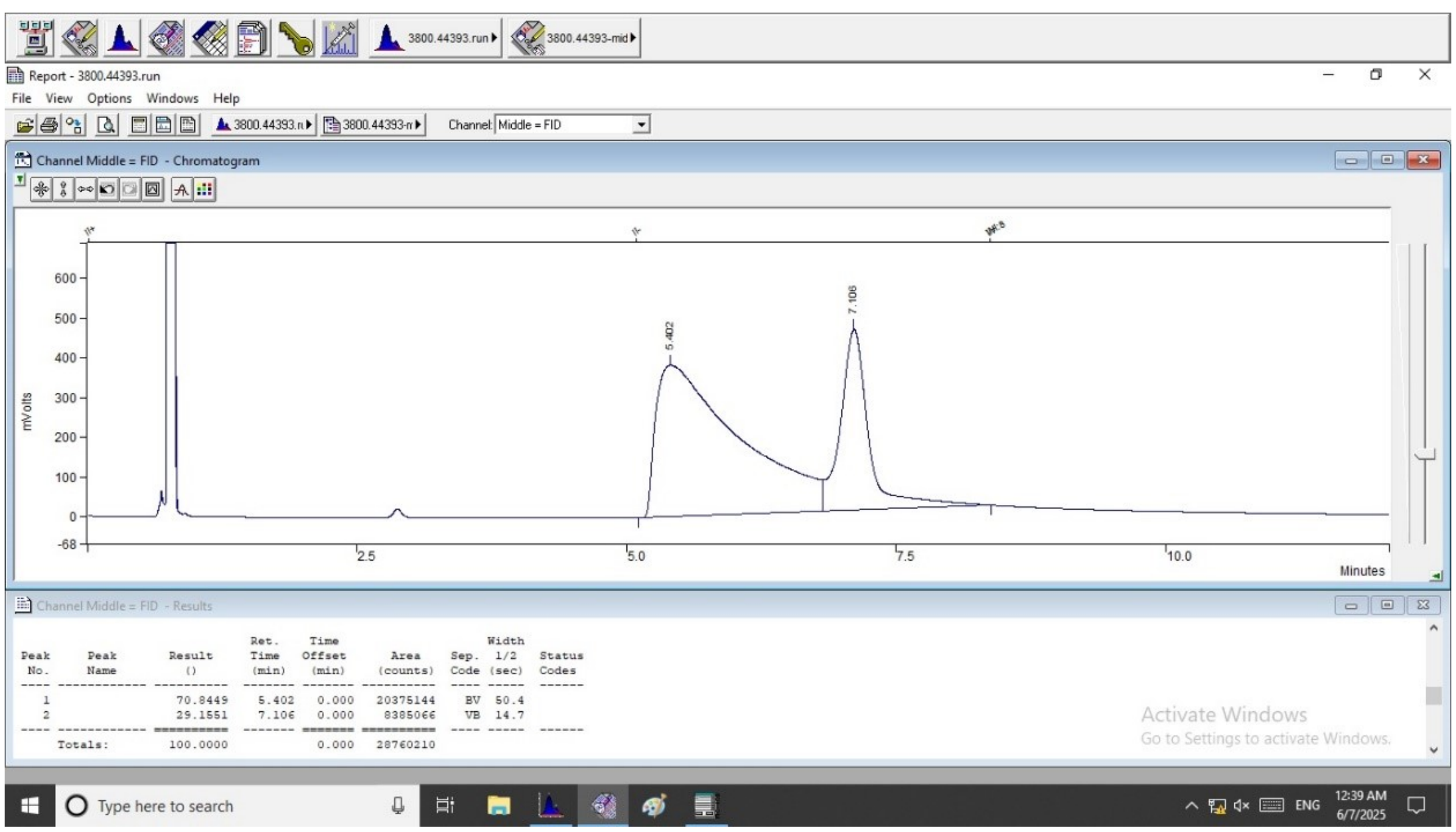

Figure S112. Gas chromatogram of the solution containing 0.75 mmol benzyl alcohol and 0.25 mmol benzyl acetate-second injection

Table S5. Summary of GC results

	X (Theoretical)	X first injection	X second injection	average
Benzyl acetate	0.75	0.776	0.778	0.777
Benzyl alcohol	0.25	0.224	0.222	0.223
Benzyl acetate	0.5	0.560	0.555	0.557
Benzyl alcohol	0.5	0.440	0.444	0.442
Benzyl acetate	0.25	0.288	0.291	0.289
Benzyl alcohol	0.75	0.712	0.708	0.710

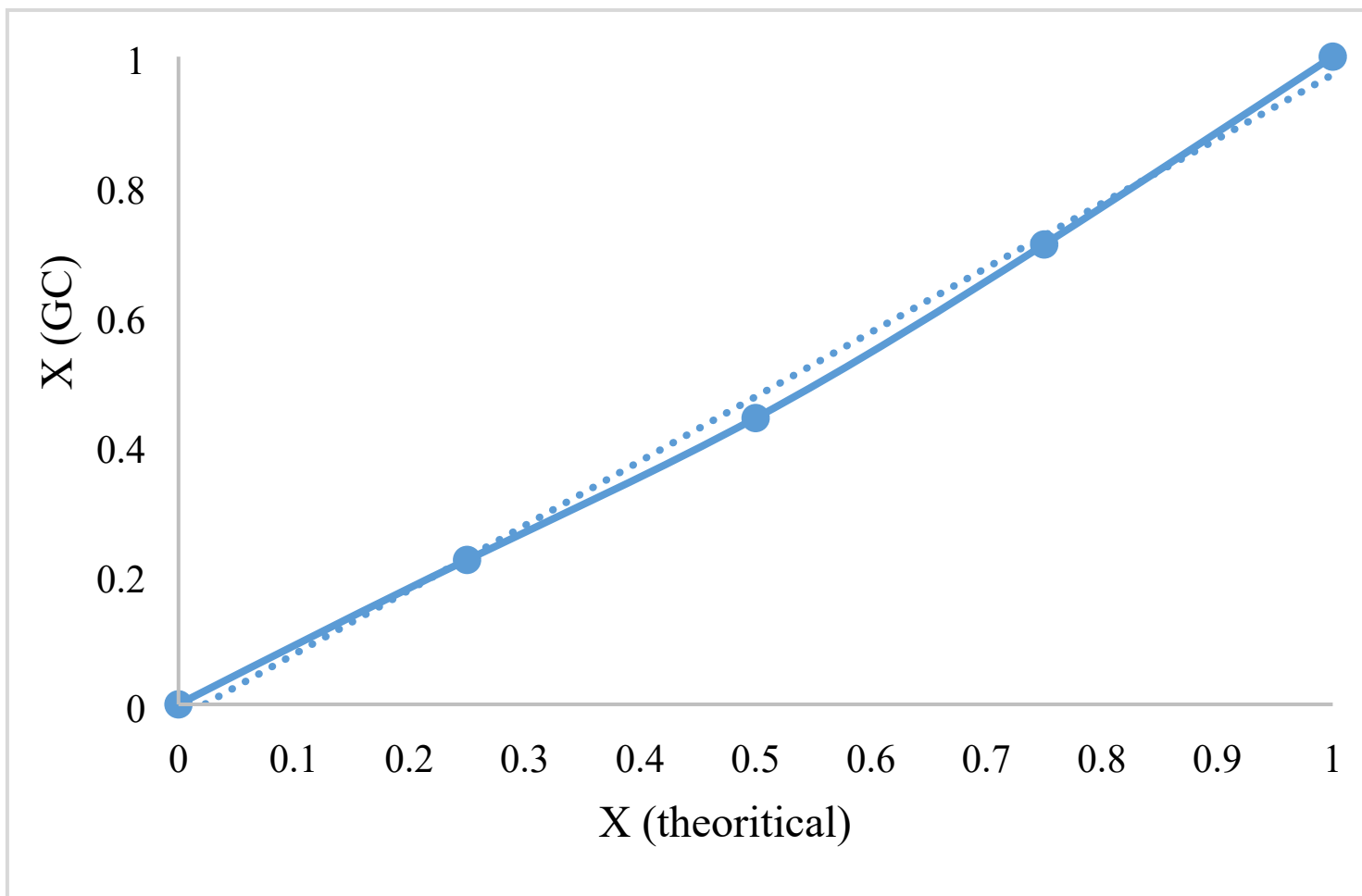

GC chromatograms for reaction mixtures:

GC chromatogram for the reaction mixture of 1 mmol of benzyl alcohol under optimized reaction conditions after 4 h in the presence of 0.8 mmol TMB as an internal standard:

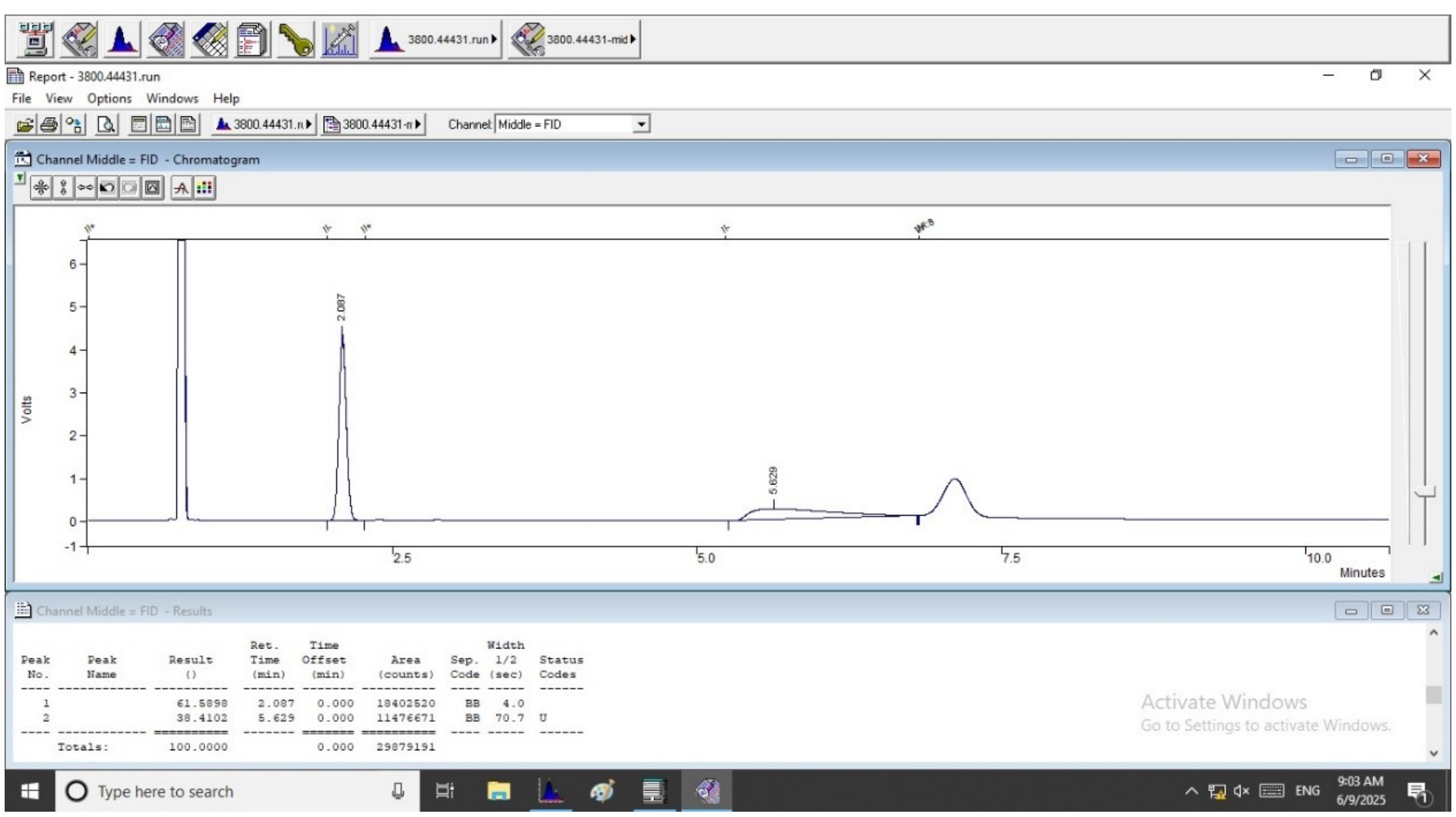

Figure S113. GC Results considering TMB and benzyl alcohol peaks after 4 h

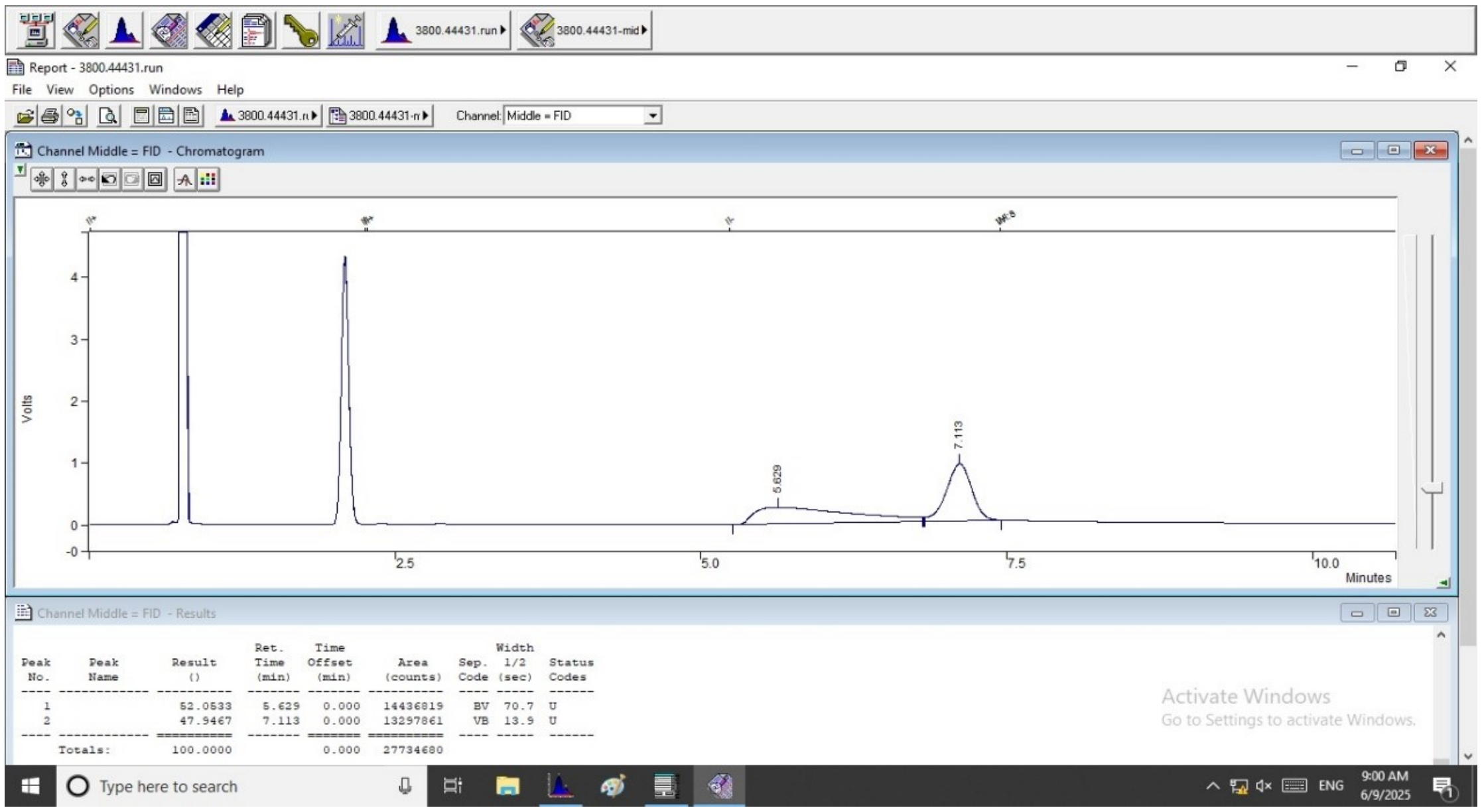

Figure S114. GC Results considering benzyl acetate and benzyl alcohol peaks after 4 h

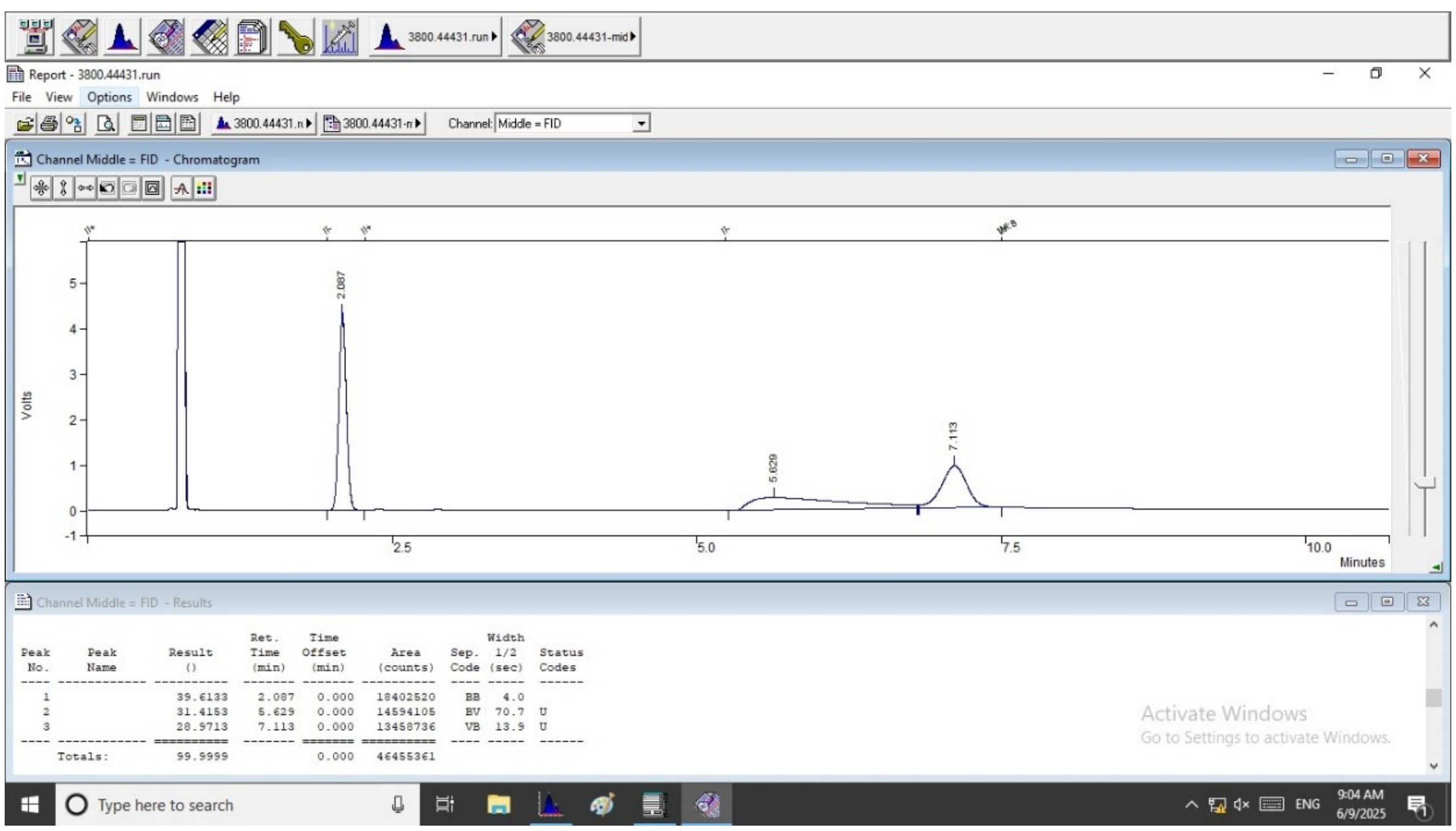

Figure S115. GC Results considering TMB, benzyl acetate and benzyl alcohol peaks after 4 h

**According to above GC chromatograms:

TMB : BnOH = 61.59 : 38.41 →→ XBnOH = 0.384

**According to the plot equation obtained from the GC results of BnOH vs TMB:

Y= 0.9844 XBnOH – 0.0144 →→ 0.384 = 0.9844 XBnOH – 0.0144 →→ XBnOH = 0.405

XBnOH = nBnOH/(nBnOH+ nTMB) →→ nBnOH = (XBnOH × nTMB)/((1-XBnOH)) = (0.405 ×0.8)/((1-0.405)) = 0.544 mmol

**According to the plot equation obtained from the GC results of BnOH vs BnOAc:

Y= 0.9948 XBnOH – 0.0224 →→ 0.523 = 0.9948 XBnOH – 0.0224 →→ XBnOH = 0.5402

XBnOH = nBnOH/(nBnOH+ nBnOAc) →→ nBnOAc = (XBnOH (1- XBnOH))/XBnOH = (0.54 (1-0.54))/0.54 = 0.46

→→ nBnOAc = 0.46 mmol

mmol total= 0.54 + 0.46 = 1 mmol

Conversion (%) = (1- nBnOH)/1 ×100 = (1-0.54)/1 × 100 →→ Reaction Conversion = 46 %

GC chromatogram for the reaction mixture of 1 mmol of benzyl alcohol under optimized reaction conditions after 8 h in the presence of 0.8 mmol TMB as an internal standard:

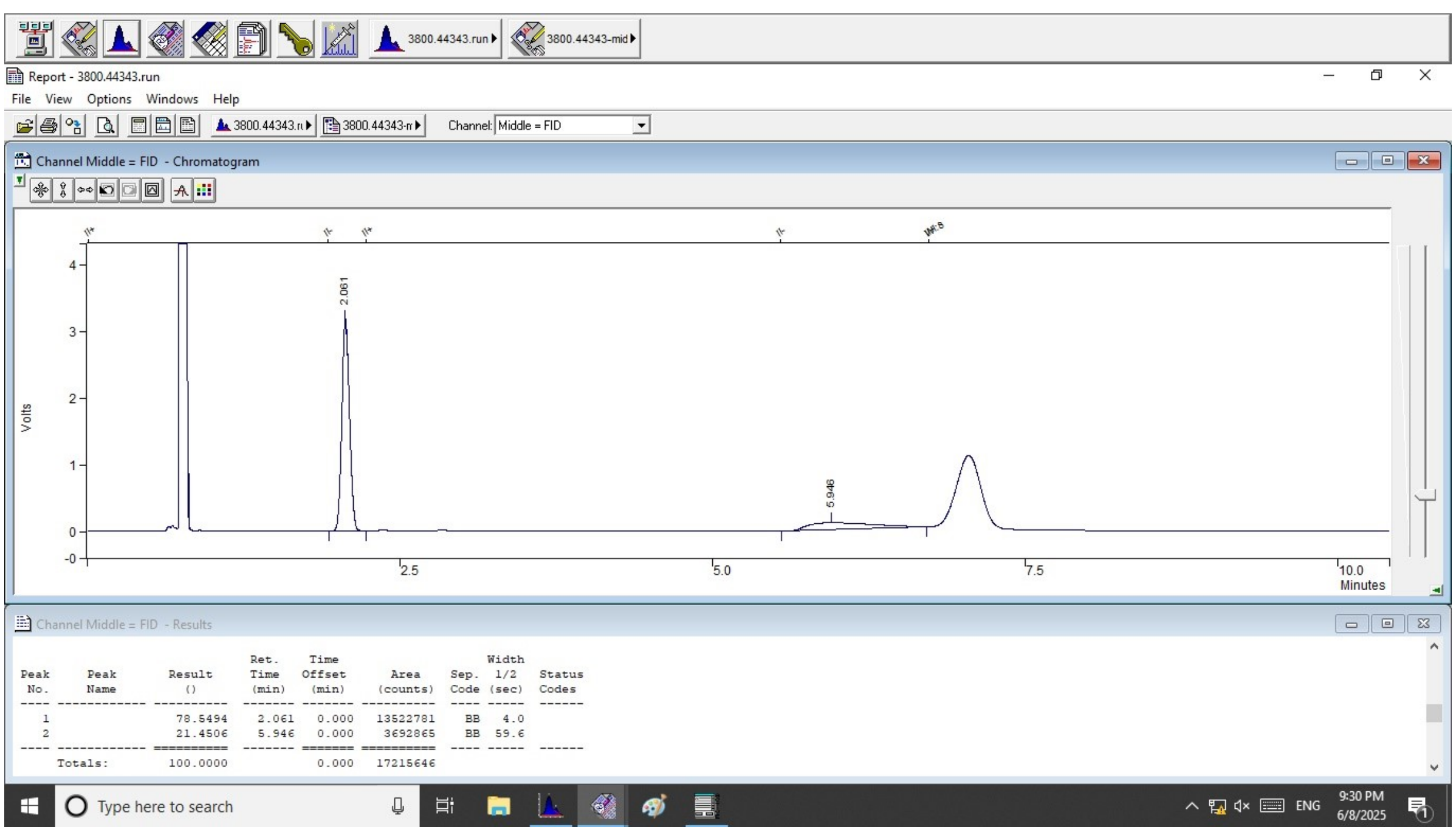

Figure S116. GC Results considering TMB and benzyl alcohol peaks after 8 h

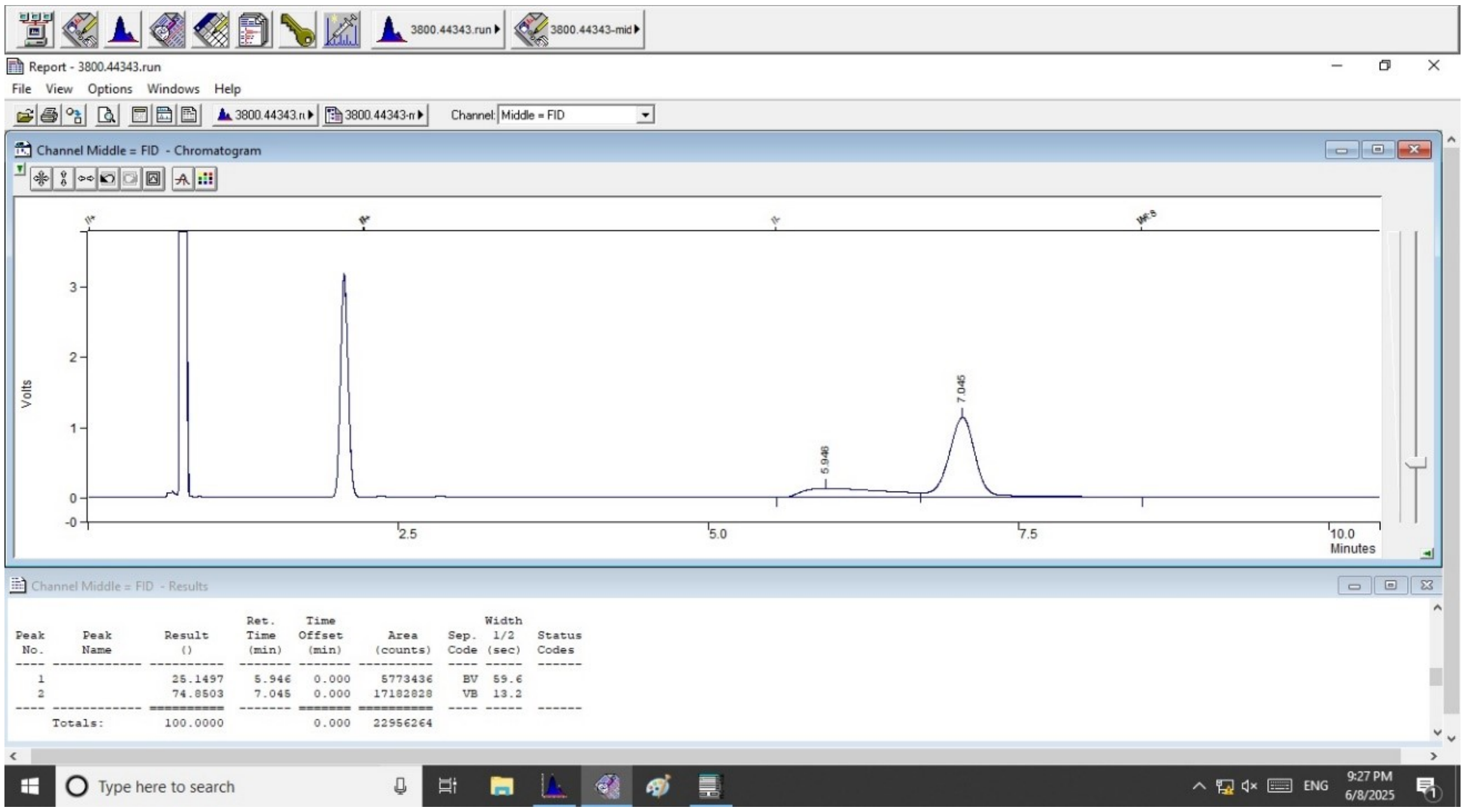

Figure S117. GC Results considering benzyl acetate and benzyl alcohol peaks after 8 h

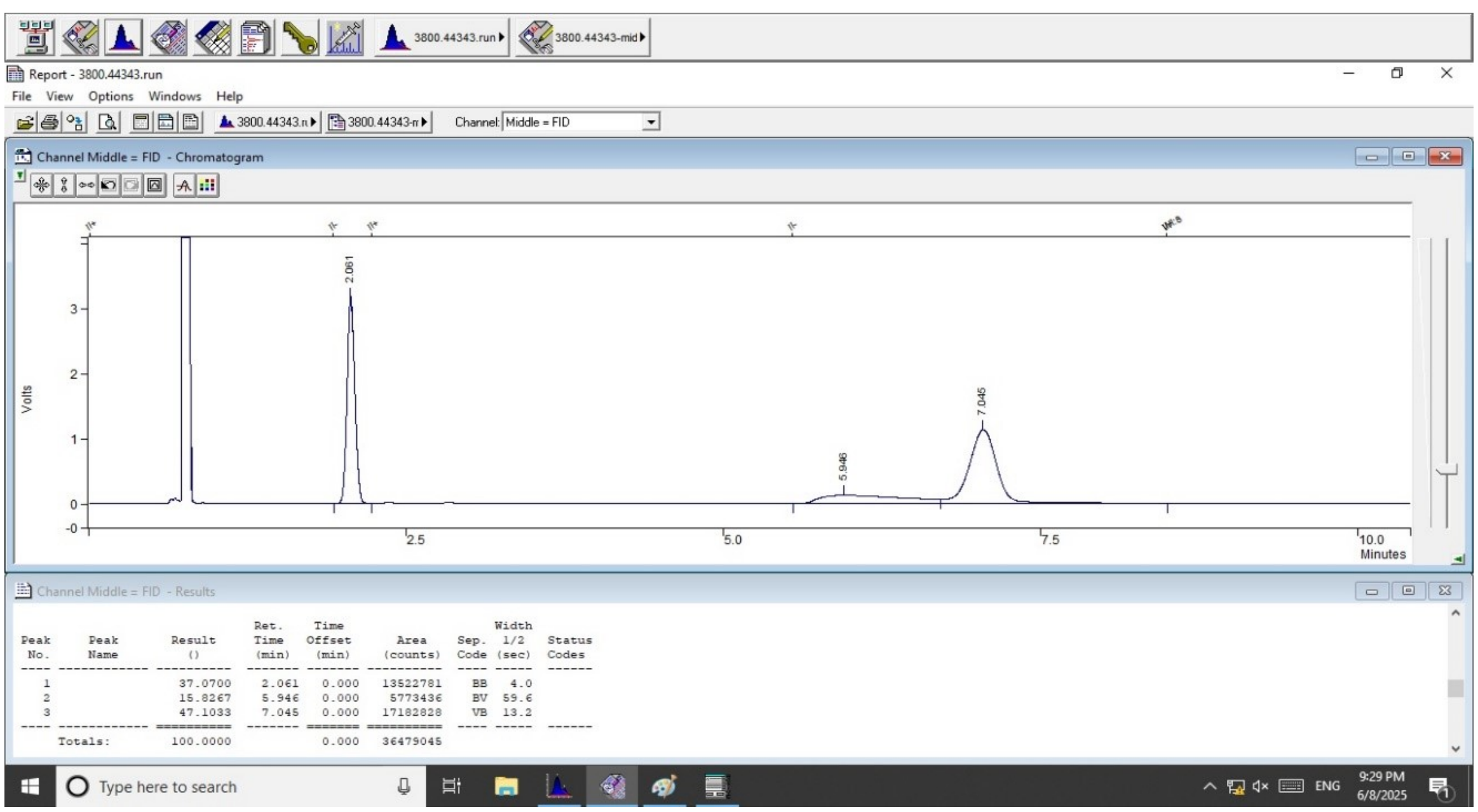

Figure S118. GC Results considering TMB, benzyl acetate and benzyl alcohol peaks after 8 h

GC chromatogram for the reaction mixture of 1 mmol of benzyl alcohol under optimized reaction conditions after12 h in the presence of 0.8 mmol TMB as an internal standard:

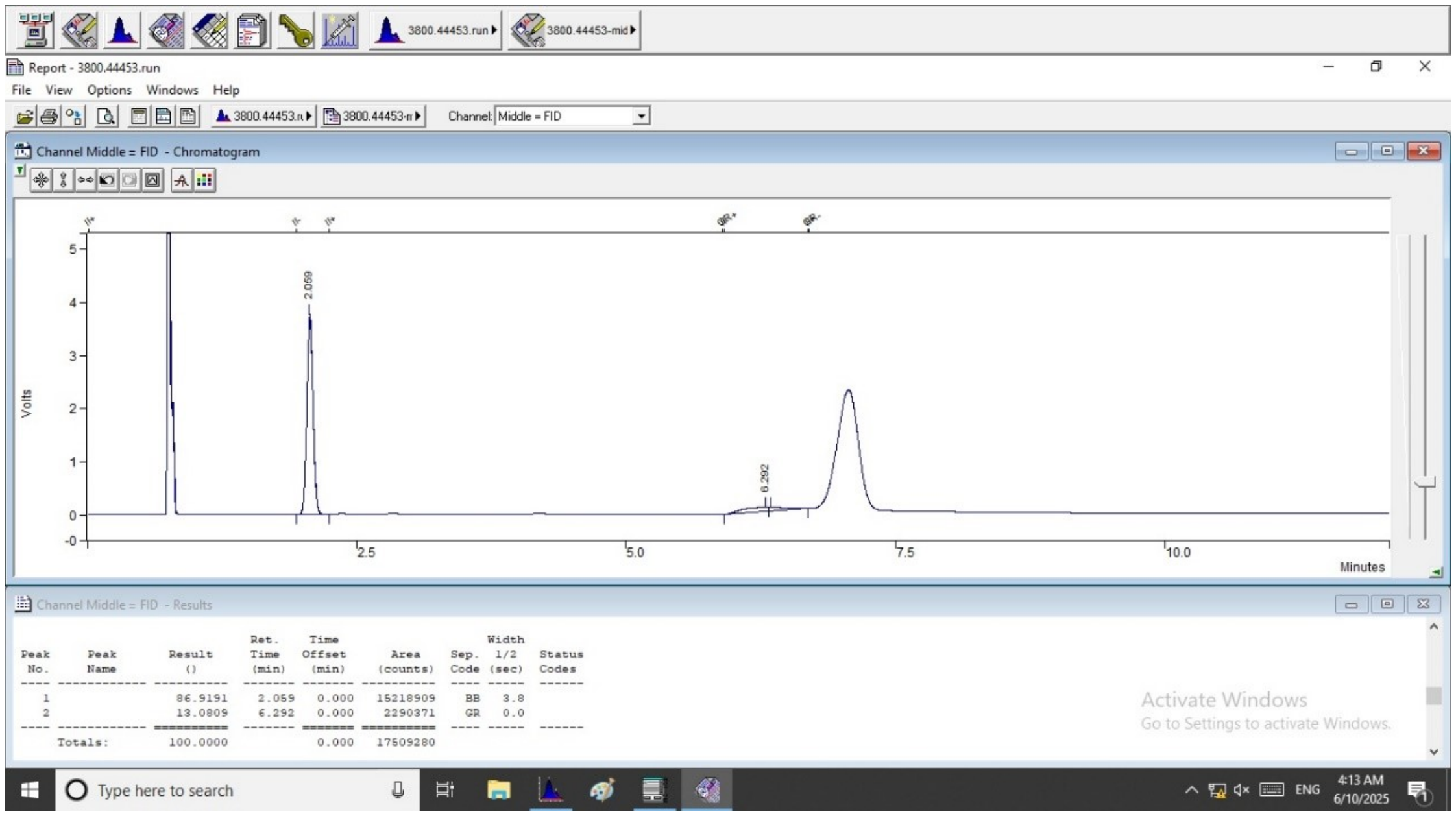

Figure S119. GC Results considering TMB and benzyl alcohol peaks after 12 h

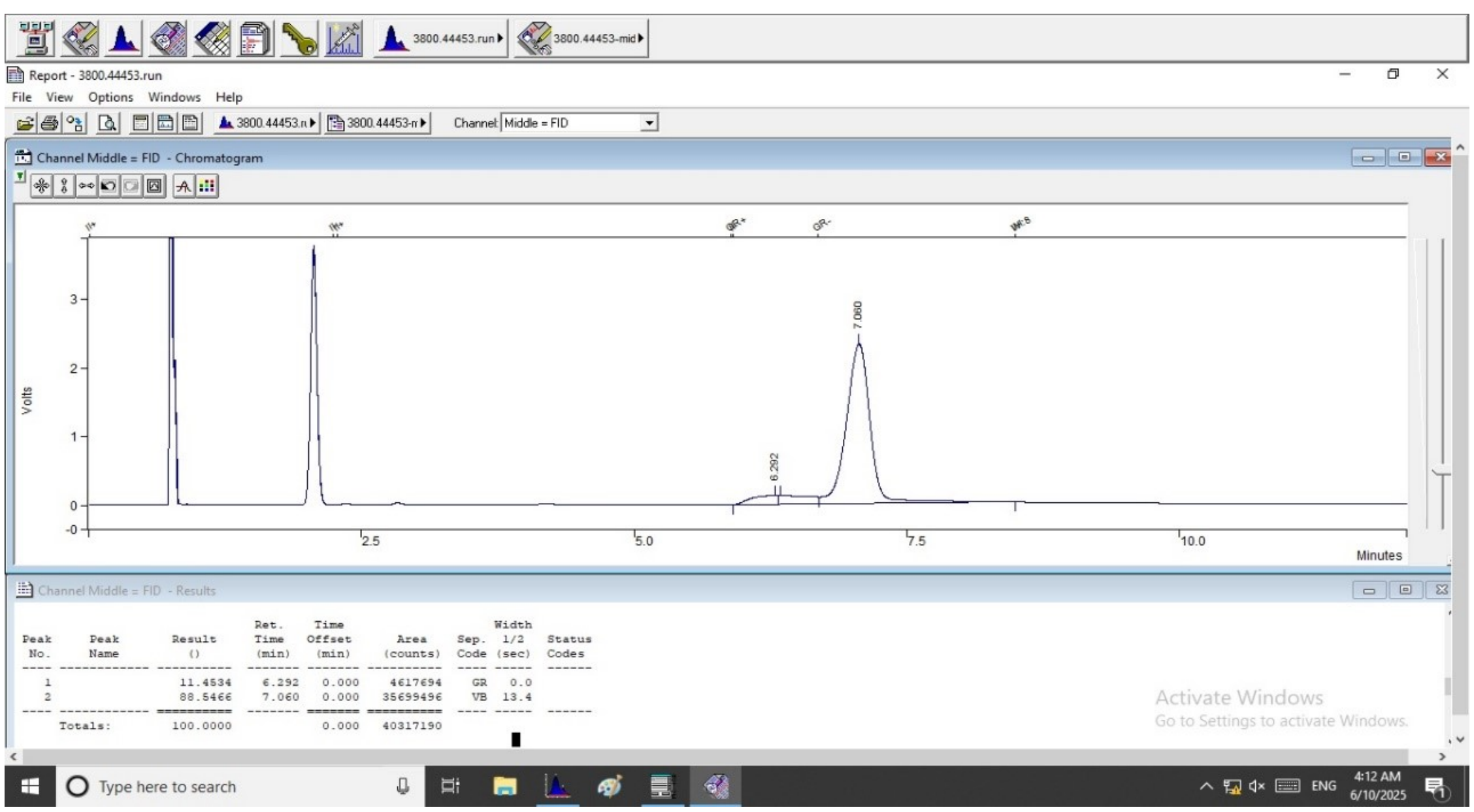

Figure S120. GC Results considering benzyl acetate and benzyl alcohol peaks after 12 h

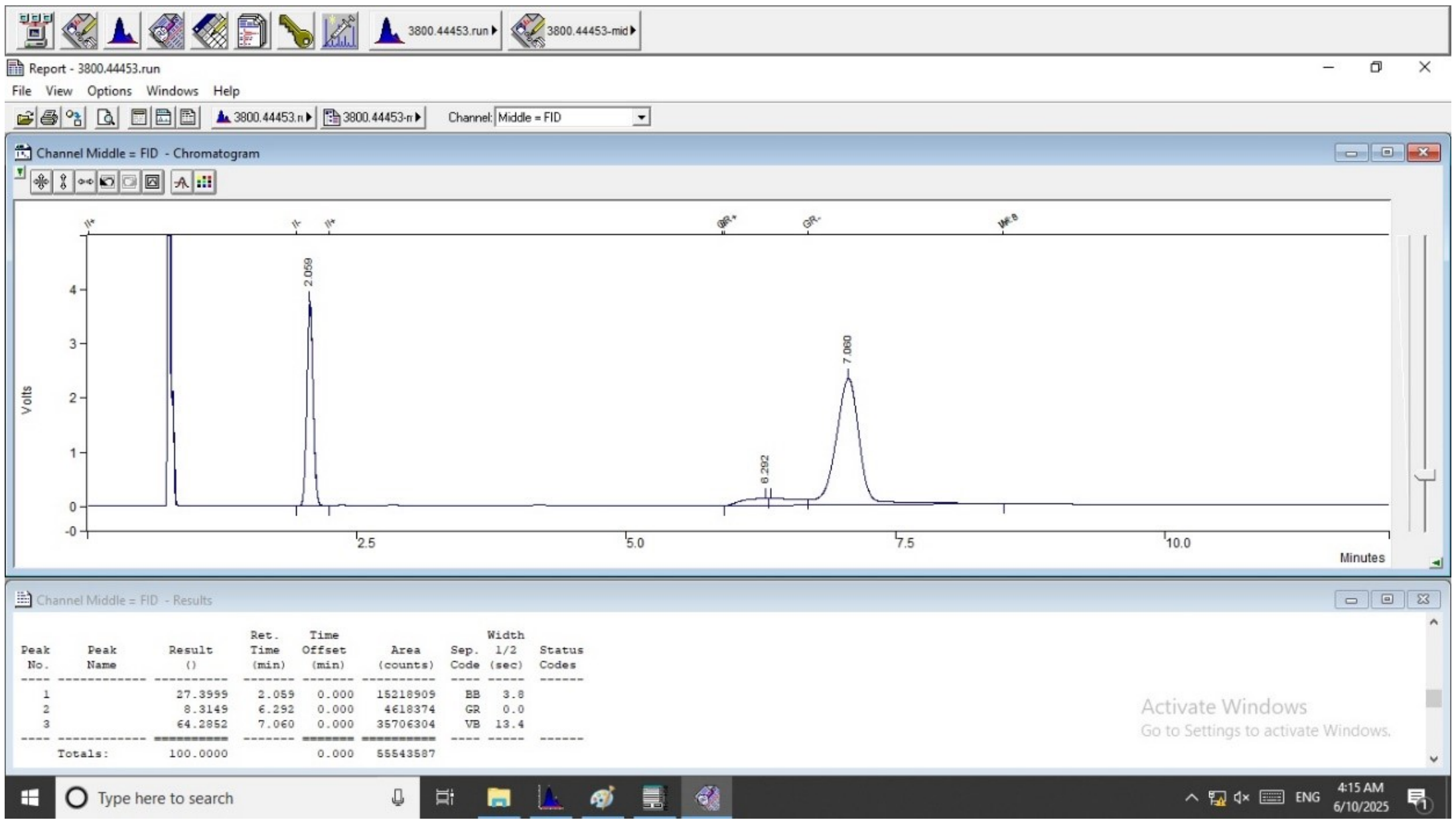

Figure S121. GC Results considering TMB, benzyl acetate and benzyl alcohol peaks after 12 h

References

[1] Karimi, B., Mirzaei, H.M., Mobaraki, A. and Vali, H., Sulfonic acid-functionalized periodic mesoporous organosilicas in esterification and selective acylation reactions. *Catal. Sci. Technol.*, **2015**, *5*(7), 3624-3631.

[2] López, M.I., Esquivel, D., Jiménez-Sanchidrián, C., Romero-Salguero, F.J. and Van Der Voort, P., "one-step" sulfonic acid PMO as a recyclable acid catalyst. *J. Catal.*, **2015**, *326*, 139-148.

[3] Elhamifar, D., Karimi, B., Moradi, A. and Rastegar, J., Synthesis of Sulfonic Acid Containing Ionic-Liquid-Based Periodic Mesoporous Organosilica and Study of Its Catalytic Performance in the Esterification of Carboxylic Acids. *ChemPlusChem*, **2014**, *79*(8), 1147-1152.

[4] Yang, Q., Kapoor, M.P., Inagaki, S., Shirokura, N., Kondo, J.N. and Domen, K., 2005. Catalytic application of sulfonic acid functionalized mesoporous benzene–silica with crystal-like pore wall structure in esterification. *J. Mol. Catal. A: Chem.*, **2005**, *230*(1-2), 85-89.
